# Estimating the effect of HIV on cervical cancer elimination in South Africa: Comparative modelling of the impact of vaccination and screening

**DOI:** 10.1016/j.eclinm.2022.101754

**Published:** 2022-11-17

**Authors:** Marie-Claude Boily, Ruanne V. Barnabas, Minttu M. Rönn, Cara J. Bayer, Cari van Schalkwyk, Nirali Soni, Darcy W. Rao, Lisa Staadegaard, Gui Liu, Romain Silhol, Marc Brisson, Leigh F. Johnson, Paul Bloem, Sami Gottlieb, Nathalie Broutet, Shona Dalal

**Affiliations:** a MRC Centre for Global Infectious Disease Analysis, School of Public Health, Imperial College London, London, United Kingdom; b Division of Infectious Diseases, Massachusetts General Hospital, Harvard Medical School, Boston, MA, USA; c Department of Global Health and Population, Harvard T.H. Chan School of Public Health, Boston, MA, USA; d The South African Department of Science and Innovation/National Research Foundation Centre of Excellence in Epidemiological Modelling and Analysis, Stellenbosch University, Stellenbosch, South Africa; e Departments of Epidemiology and Global Health, University of Washington, Seattle, WA, USA; f Centre de Recherche du CHU de Québec, Québec, Canada; g Département de médecine sociale et préventive, Université Laval, Québec, Canada; h Centre for Infectious Disease Epidemiology and Research, University of Cape Town, Cape Town, South Africa; i Department of Immunization, Vaccines and Biologicals, World Health Organization, Geneva, Switzerland; j Department of Sexual and Reproductive Health and Research, World Health Organization, Geneva, Switzerland; k Department of Global HIV, Hepatitis and STIs Programmes, World Health Organization, Geneva, Switzerland

**Keywords:** Cervical cancer elimination, HPV vaccination, cervical cancer screening, HIV, South Africa

## Abstract

**Background:**

In 2020, the World Health Organization (WHO) launched its initiative to eliminate cervical cancer as a public health problem. To inform global efforts for countries with high HIV and cervical cancer burden, we assessed the impact of human papillomavirus (HPV) vaccination and cervical cancer screening and treatment in South Africa, on cervical cancer and the potential for achieving elimination before 2120, considering faster HPV disease progression and higher cervical cancer risk among women living with HIV(WLHIV) and HIV interventions.

**Methods:**

Three independent transmission-dynamic models simulating HIV and HPV infections and disease progression were used to predict the impact on cervical cancer incidence of three scenarios for all women: 1) girls' vaccination (9–14 years old), 2) girls' vaccination plus 1 lifetime cervical screen (at 35 years), and 3) girls’ vaccination plus 2 lifetime cervical screens (at 35 and 45 years) and three enhanced scenarios for WLHIV: 4) vaccination of young WLHIV aged 15–24 years, 5) three-yearly cervical screening of WLHIV aged 15–49 years, or 6) both. Vaccination assumed 90% coverage and 100% lifetime protection with the nonavalent vaccine (against HPV-16/18/31/33/45/52/58). Cervical cancer screening assumed HPV testing with uptake increasing from 45% (2023), 70% (2030) to 90% (2045+). We also assumed that UNAIDS 90-90-90 HIV treatment and 70% male circumcision targets are reached by 2030. We examined three elimination thresholds: age-standardised cervical cancer incidence rates below 4 or 10 per 100,000 women-years, and >85% reduction in cervical cancer incidence rate. We conducted sensitivity analyses and presented the median age-standardised predictions of outcomes of the three models (minimum–maximum across models).

**Findings:**

Girls' vaccination could reduce age-standardised cervical cancer incidence from a median of 47.6 (40.9–79.2) in 2020 to 4.5 (3.2–6.3) per 100,000 women-years by 2120, averting on average ∼4% and ∼46% of age-standardised cumulative cervical cancer cases over 25 and 100 years, respectively, compared to the *basecase*. Adding 2 lifetime screens helped achieve elimination over the century among all women (2120 cervical cancer incidence: 3.6 (1.9–3.6) per 100,000 women-years), but not among WLHIV (10.8 (5.3–11.6)), and averted more cumulative cancer cases overall (∼45% over 25 years and ∼61% over 100 years compared to *basecase*) than girls' vaccination alone. Adding three-yearly cervical screening among WLHIV (to girls' vaccination and 2 lifetime cervical screens) further reduced age-standardised cervical cancer incidence to 3.3 (1.8–3.6) per 100,000 women-years overall and to 5.2 (3.9–8.5) among WLHIV by 2120 and averted on average 12–13% additional cumulative cancer cases among all women and 21–24% among WLHIV than girls’ vaccination and 2 lifetime cervical screens over 25 years or longer. Long-term vaccine protection and using the nonavalent vaccine was required for elimination.

**Interpretation:**

High HPV vaccination coverage of girls and 2 lifetime cervical screens could eliminate cervical cancer among women overall in South Africa by the end of the century and substantially decrease cases among all women and WLHIV over the short and medium term. Cervical cancer elimination in WLHIV would likely require enhanced prevention strategies for WLHIV. Screening of WLHIV remains an important strategy to reduce incidence and alleviate disparities in cervical cancer burden between women with and without HIV, despite HIV interventions scale-up.

**Funding:**

10.13039/100004423World Health Organization. National Cancer Institute, National Institutes of Health. MRC Centre for Global Infectious Disease Analysis, 10.13039/501100000265UK Medical Research Council. National Institute of Child Health and Human Development research. 10.13039/501100001325Cancer Association of South Africa. 10.13039/501100000024Canadian Institutes of Health Research and the Fonds de recherche du Québec – Santé research.

**Research in context**

**Evidence before this study**

In 2020, the World Health Organization (WHO) launched the Cervical Cancer Elimination Initiative with a global strategy composed of three intervention targets by 2030: HPV vaccination for 90% of girls by the age of 15, 2 lifetime cervical screens with high performance tests for 70% of women by age 45, and treatment of 90% of women with cervical disease. These recommendations were informed by the results of a mathematical model comparison exercise from the cervical cancer elimination-modelling consortium (CCEMC) in 78 low- and middle-income countries.

However, this comparative modelling analysis did not explicitly account for the multiple interactions between HPV and HIV infections and HIV intervention scale-up, which influence HPV natural history and disease progression risk to cervical cancer in WLHIV; this is particularly relevant for regions with high HIV burden, such as southern Africa where more than half of all cervical cancer cases may be attributable to HIV.

**Added value of this study**

As part of the CCEMC, we undertook a novel comparative mathematical modelling analysis (using three independent calibrated transmission dynamic models of co-circulating HIV and HPV) to evaluate the potential impact of WHO triple-intervention elimination strategy and additional enhanced strategies for WLHIV in South Africa, a country highly burdened by HIV and cervical cancer.

Our results suggest that HPV vaccination of 90% of girls with the nonavalent vaccine, lifelong protection, and 2 lifetime cervical screens (WHO's triple-intervention elimination strategy) could reduce cervical cancer in all women below the 4 cases per 100,000 woman-years threshold by 2120 in South Africa but that it would be unlikely with girls' vaccination alone, despite a substantial decrease in cervical cancer incidence. Adding 2 lifetime cervical screens to girls' vaccination accelerated the decrease in cervical cancer incidence compared to girls' vaccination alone and averted more cumulative cervical cancer cases over the next century. Despite substantial decrease in cancer incidence and the sustained scale-up of antiretroviral treatment, WHO's triple-intervention strategy was insufficient to eliminate cervical cancer among WLHIV; adding three-yearly screening among WLHIV was required to approach the 4 cancer cases per 100,000 women-years elimination threshold among WLHIV.

**Implication of all available evidence**

Implementing the WHO's triple intervention elimination strategy for all women (with high coverage of a broad and highly effective HPV vaccine and 2 lifetime cervical screens), with additional efforts to screen WLHIV every three years, as recommended in recent WHO guidelines, could substantially reduce cervical cancer incidence, reduce the stark global disparities seen in cervical cancer burden, and eliminate cervical cancer as a public health problem by the end of the century in a country with high burden of both HIV and cervical cancer. Integrating cervical cancer screening and treatment with HIV services, which are well established and have high coverage, may spur efficiencies and simplify service delivery.

## Introduction

Cervical cancer is the second most frequent cancer among women in low- and middle-income countries (LMICs)^1^, ^2^, ^3^ and the leading cause of cancer deaths among women^4^, ^5^, ^6^ in sub-Saharan Africa. Approximately 20% of the estimated 604,000 new cervical cancer cases and 342,000 deaths worldwide in 2020 occurred in women living in sub-Saharan Africa,^1^ reflecting persisting worldwide inequities in access to and uptake of HPV vaccination and effective screening and treatment interventions, and concomitant high HIV prevalence.^7^ Women living with HIV (WLHIV) are estimated to be six times more likely to develop cervical cancer.^4^^,^^7^, ^8^, ^9^ Given the high HIV prevalence in southern Africa (Female 15+ years old HIV prevalence ∼29.8%), about half of cervical cancer cases in 2018 may be attributable to HIV.^7^^,^^8^

Since 2007, four safe and very efficacious vaccines (efficacy≥93%) have been licensed.^3^^,^^9^, ^10^, ^11^ The bivalent/quadrivalent, and nonavalent vaccines protect against high oncogenic risk HPV types that cause about 70% (HPV-16, 18) and 90% (HPV-16, 18, 31, 33, 45, 52, 58) of cervical cancers, respectively.^9^^,^^12^^,^^13^ Countries that have achieved high vaccination coverage have reported substantial declines in vaccine-type HPV prevalence (∼80%) and in high-grade lesions (CIN2+) (∼45–50%) among women 10 years after routine vaccination programs were introduced.^14^, ^15^, ^16^ All HPV vaccines also elicit a strong immune response against HPV vaccine types among people living with HIV.^10^^,^^17^^,^^18^ Population-based cervical cancer screening programs have also reduced cervical cancer incidence in many high-income countries (HICs).^19^^,^^20^ However, in 2020, only about 30% of countries in Africa had introduced national HPV vaccination programs and cervical cancer screening coverage has been low in sub-Saharan Africa (average of 17% (range: 1–51%) women ever screened).^1^^,^^21^^,^^22^ Mathematical modelling studies suggest that girls-only HPV vaccination and cervical cancer screen-and-treat strategies are cost-effective in LMICs,^23^, ^24^, ^25^, ^26^ which could greatly benefit from vaccination and improvement in cervical cancer screening.

Following the WHO Director-General's call for action, 194 countries adopted WHO's Global strategy for the elimination of cervical cancer as a public health problem in 2020.^27^^,^^28^ The triple strategy defines elimination as reaching and maintaining cervical cancer incidence rates below 4 per 100,000 women-years by 2120 through achieving and maintaining the 90-70-90 targets by 2030: 90% of girls fully vaccinated with HPV vaccine by age 15 years, 70% of women receiving 2 lifetime cervical screens, and 90% of women identified with cervical disease receiving appropriate treatment.^28^ Prior to this, WHO convened a cervical cancer elimination modelling consortium (CCEMC) to undertake a mathematical model comparison exercise and determine if, when, where, and how cervical cancer elimination could be achieved in 78 LMICs.^29^^,^^30^ The CCEMC model predictions, which informed WHO's Global Strategy, suggested that although elimination was possible it would be slower and more difficult to achieve in settings where cervical cancer incidence is high such as in sub-Saharan Africa.^27^ However, none of the models accounted for the effects of HIV infection and HIV interventions, such as HIV treatment and male circumcision, that may directly and/or indirectly (through reducing HIV infection) influence HPV acquisition and/or disease progression.^31^, ^32^, ^33^, ^34^, ^35^, ^36^, ^37^ This brings into question the potential impact of HIV on WHO's elimination strategy recommendations and whether specific vaccination and/or cervical cancer screening programs for WLHIV are needed in high HIV prevalence settings. Since sub-Saharan Africa has the highest burden of cervical cancer incidence and deaths, it is important to assess the robustness of the WHO strategy for cervical cancer elimination in high HIV prevalence settings and explore if focusing on WLHIV can accelerate impact.

The WHO's CCEMC chose South Africa as a priority country to conduct a comparative modelling analysis and determine the potential for cervical cancer elimination using WHO 90-70-90 strategy. Since the country has one of the highest HIV prevalence levels worldwide and an age-standardised cervical cancer incidence rate at least twice the global average,^3^^,^^38^^,^^39^ results from a comparative modelling analysis in South Africa can guide strategies for other high HIV burden countries.

Our specific objectives were to explore the impact of different vaccination and screening scenarios for all women and for WLHIV in particular to: 1) identify prevention strategies that lead to elimination, 2) estimate the timing of elimination using different elimination thresholds, and 3) predict the fraction of cervical cancer cases averted on the path to elimination. We investigated the impact of the different scenarios overall and for WLHIV.

## Methods

### Overview of comparative modelling exercise

The comparative modelling analysis was conducted as follows. First, we searched for dynamic models that explicitly simulated HIV and HPV transmission and disease progression and HIV interventions calibrated to the South African context. At the project start, only three such models were identified and included. Second, we defined relevant scenarios of HPV vaccination and screening strategies, which included the three main scenarios evaluated in the first CCEMC model comparison^29^^,^^30^ and three additional enhanced screening and vaccination scenarios for WLHIV, as well as several sensitivity analysis scenarios (described below). The models were developed and calibrated independently by each team without harmonizing model structures or the parameters governing the setting, disease, and population, in order to fully reflect structural, parameter, and epidemiological data uncertainty. Each calibrated model was used to predict the population-level impact of the different scenarios on cervical cancer incidence over time.

### Model description

Our study included one individual-based model developed in South Africa (*MicroCOSM-HPV*)^40^ and two deterministic models developed in the US (*DRIVE*)^41^ and in the US/UK (*Det_HIV-HPV*).^42^^,^^43^ All three independent models incorporated the key features necessary to assess the population-level effectiveness of HPV and cervical cancer interventions in high HIV prevalence settings. They were transmission-dynamic models of co-circulating HPV and HIV infections and associated diseases that included the following components: demography, sexual behaviour, HPV and HIV transmission, progression through HIV disease stages from acute stage to death, progression through HPV disease stages from infection to cervical cancer via precancerous cervical lesions, multiple HIV and HPV infection/disease associated interactions (which explain heightened cervical cancer risk in WLHIV), HIV prevention interventions (HIV treatment, male circumcision, and condoms) and HPV/cervical cancer interventions (vaccination, screening and diagnosis, and treatment of high grade cervical lesions).

The models represented HPV transmission and natural history of cervical cancer associated with the high-risk HPV types (HR-HPV) included in the nonavalent vaccine (HPV-16,18,31,33,45,52,58) and other non-vaccine HR-HPV types stratified by HIV status. The models simulated HPV transmission in an open and growing heterosexual population stratified by age and sexual behaviour risk groups, and accounted for subgroups’ sexual activity levels, sexual mixing by age and sexual activity levels, and changes in the levels of HIV and HPV/cervical cancer interventions over time since the start of the HIV epidemic. All models represented prophylactic vaccination that protects against infection with the specific high-risk HPV vaccine types (without therapeutic effects) and captured the direct and indirect (herd immunity) effects of vaccination. The models also simulated different cervical screening and treatment programs (e.g., screening age, frequency). Key differences between model structure and demographic, HPV/cervical cancer and HIV biological and epidemiological assumptions and baseline level of interventions are summarised in Table T3, section 2 of the technical appendix.

### Calibration by setting

All models were calibrated to demographic, stratified sexual behaviour and epidemiological HIV and HPV/cervical cancer data over time (e.g., by sex, age, HIV status) specific to South Africa at the national level (*MicroCOSM-HPV, Det_HIV-HPV*) and to Kwazulu-Natal province (KZN) (*DRIVE*) (Section 3 of technical appendix). All models used a Bayesian multidimensional calibration process that identified a posterior subset of demographic, behavioural and biological parameter sets, from pre-specified prior distributions, that generated model predictions reflecting parameter uncertainties and that were consistent with several epidemiological outcomes estimates up to 2020 (Section 3 of technical appendix). The posterior parameter sets that reproduced available data and accounted for pre-existing levels of HIV interventions prior to 2020 were used to simulate the *basecase* scenario. The different intervention scale-up scenarios were introduced from 2020 onward.

South Africa has had a national screening program since 2000, but uptake has remained limited^44^, ^45^, ^46^ and late-stage diagnoses is common.^33^^,^^44^^,^^47^, ^48^, ^49^, ^50^, ^51^, ^52^, ^53^, ^54^, ^55^, ^56^ National HPV vaccination with the bivalent vaccine was introduced in 2014 among 9-year-olds public schoolgirls, which initially achieved a coverage of ∼80% and ∼60% for the 1st and 2nd dose, respectively,^57^^,^^58^ but has decreased subsequently ( ∼40% in 2021).^59^^,^^60^ Given the existence of a national screening program since 2000 in South Africa, the models' *basecase* scenarios assumed some levels of screening prior to 2020 (e.g. <13% of women aged 35-39 year-olds are screened annually while treatment success is low across models) (Table T3 of the technical appendix).^45^ As in the previous CCEMC modelling analysis,^29^ our *basecase* scenario assumed no vaccination prior to 2020. In the intervention scenarios, this assumption has a negligible impact on our post 2020 cervical cancer incidence predictions since the maximum coverage achieved in 9–14 years old girls by 2020 by the national program is below the coverage level simulated in our vaccination of 9–14 year-old girls’ scenario. Additional details on intervention scenarios, model structure and assumptions, calibration, model fits and outcomes are presented in Section 2 of technical appendix.

Models fits to key demographic, HIV, HPV, and cervical cancer outcomes, which highlight key similarities and differences between sites and models are shown in Table T4 of the technical appendix. Briefly, KZN (*DRIVE* model) has a higher overall HIV prevalence than South Africa (∼27% in KZN versus ∼20% nationally among adults aged 15–49 in 2017),^61^ a higher proportion of persons living with HIV virally suppressed (68% in KZN versus 62% nationally in 2017), and similar medical male circumcision coverage (∼30% among men aged >15 years-old in 2017 in KZN and national).^61^ The national models used age-stratified cervical cancer incidence estimates from Globocan (*Det_HIV-HPV*)^39^^,^^62^ or the age stratified pathology-diagnosed incidence in 2017 (*MicroCOSM-HPV*)^63^ for calibration and predicted similar overall age-standardised cervical cancer estimates in 2020 (similar to Globocan 2018 estimates of 43.5 per 100,000 women-years).^39^ As reliable estimates of cervical cancer incidence estimates were not available for KZN, the Globocan national cancer incidence rates were adjusted by age to account for the higher HIV prevalence in KZN, which resulted in higher predicted estimates than the national models.

### Vaccination and screening scale-up scenarios

The three main HPV vaccination and cervical cancer screening scenarios explored were: girls' vaccination of 9–14-year-old girls (Scenario 1), girls' vaccination plus 1 lifetime cervical screen of all women aged 35 years (Scenario 2), and girls’ vaccination plus 2 lifetime cervical screens of all women aged 35 and 45 years (Scenario 3) (Table 1A and Table T1A of the technical appendix). The enhanced scenarios for WLHIV added: multi-age cohort vaccination of young WLHIV aged 15–24 to scenario 1 (Scenario 4), frequent screening of WLHIV aged 25-49 years-old every three years to scenario 3 (Scenario 5), vaccination of young WLHIV and frequent screening of WLHIV to scenario 3 (Scenario 6) (Table 1B, and Table T1B of the technical appendix).

**Table 1:** HPV vaccination & cervical cancer screening scenarios (additional details are provided in Section 1 of technical appendix).

Scenarios	Description
A) Main HPV vaccination & cervical cancer screening scenarios for all women	
Girls' vaccination (**Sc1**):	Routine vaccination of 9–14-year-old girls, with a 90% vaccine coverage
Girls' vaccination & 1 lifetime cervical screen (**Sc2**):	**Sc1** + 1 lifetime cervical screen for women at age 35 years, with a high screening ramp-up (45% in 2023, 70% in 2030, and 90% in 2045)
Girls' vaccination & 2 lifetime cervical screens (**Sc3**)^a^:	**Sc1** + 2 lifetime cervical screens for all women at age 35 and 45 years, with a high screening ramp-up
B) Main enhanced HPV vaccination & cervical screening scenarios for WLHIV	
Vaccination of young WLHIV (**Sc4**):	**Sc1** + multi-age cohort vaccination of WLHIV aged 15–24 years, with 90% vaccine coverage
Frequent cervical screening of WLHIV (**Sc5**):	**Sc3** + three-yearly screening of WLHIV aged 25–49 years old, with a high cervical screening ramp-up
Vaccination and frequent cervical screening of WLHIV (**Sc6**):	**Sc3** +multi-age cohort vaccination of WLHIV aged 15–24 years, with 90% vaccine coverage + three-yearly screening of WLHIV aged 25–49 years old, with a high cervical screening ramp-up

a WHO recommended triple-intervention global cervical cancer elimination strategy.

Analogous to the previous CCEMC model comparison,^29^^,^^30^ we assumed 90% vaccination coverage and that HPV vaccination provided a lifelong 100% efficacy protection against infection with HPV-16,18,31,33,45,52,58. We assumed the same efficacy regardless of HIV status.^10^ All screening scenarios assumed a high screening ramp-up with annual uptake (i.e. proportion of eligible women screened annually) increasing from 45% in 2023, to 70% in 2030, and 90% in 2045. We further assumed the use of HPV testing in screening, with 100% treatment efficacy and 10% lost-to-follow-up for both women living and not living with HIV. We also assumed that the UNAIDS 90-90-90 HIV treatment target, which results in 73% people living with HIV being virally suppressed, and the 70% male circumcision coverage target, would be achieved by 2030 and be maintained thereafter.

Sensitivity analyses were performed to assess the influence of HPV vaccination coverage (80% versus 90%), extending ages of multi-cohort vaccination of WLHIV (15–45 years and all ages versus 15–24 years), HR-HPV types included in the vaccine (HPV-16 and 18 only versus HPV-16,18,31,33,45,52,58), duration of vaccine protection (20 years versus lifelong), and HIV intervention scale-up (remaining at 2020 levels versus achieving UNAIDS 90-90-90 and the 70% male circumcision targets) (Table T2 of the technical appendix).

### Outcomes

As in the previous CCEMC analysis, population-level impact was measured using three main age-standardised outcomes from 2020 to 2120: i) the cervical cancer incidence rate per 100,000 women-years over time, ii) the relative decrease in cervical cancer incidence rate compared to the *basecase* over time, and iii) the cumulative fraction of cervical cancer cases averted over time since 2020 compared to the *basecase*. Each model was used to predict the different age-standardised outcomes at the national level (2 models) and for KZN province (1 model). Outcomes were age-standardised by applying the 2015 world standard age distribution of the female population aged 0–99 years to the predicted age-specific incidence at each time point (details in Section 4 of technical appendix),^64^ for each scenario, women overall, and women stratified by HIV and HIV treatment status. For each model and scenario, we derived the median (and 90% uncertainty interval) of the cancer incidence predictions from the posterior parameters sets. The relative decrease and the cumulative fraction averted reflect the proportional decrease in the relevant median incidence outcome. When combining results from the three models, we presented the median and range (minimum and maximum) across the three models to reflect variations between models and settings.

### Elimination thresholds

We used the main and two alternative elimination thresholds previously defined by WHO and the WHO Global Strategy towards cervical cancer elimination: age-standardised cervical cancer incidence rate below 4/100,000 women-years, or below 10/100,000 women-years, or a ≥85% reduction in age-standardised cervical cancer incidence versus the *basecase*.^29^ The predicted time of elimination was defined as the first year when each threshold was reached.

### Role of the funding source

The research was partly funded by the WHO. WHO contributed to the study design, data interpretation, and writing of the report. Other funders had no role in this work.

## Results

### Impact of HPV vaccination and screening strategies for all women on cervical cancer incidence (Scenarios 1–3)

**Fig. 1:**
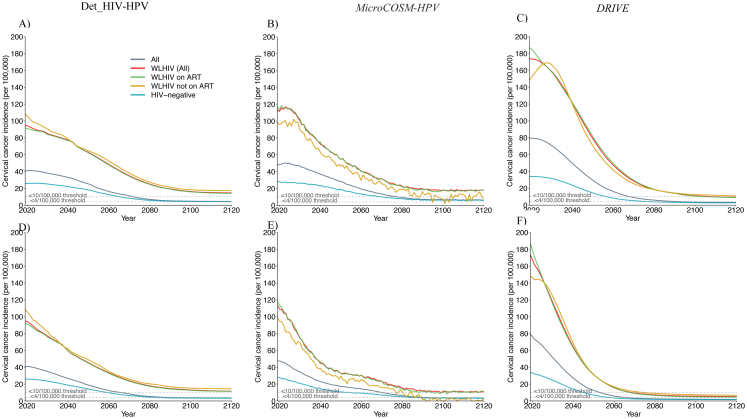
**Temporal dynamics of cervical cancer incidence after HPV vaccination and cervical cancer screening for all women - among all women:** Age-standardised cervical cancer incidence rate per 100,000 women-years among all women after the ramp-up of girls' vaccination only (Sc1), girls' vaccination and 1 lifetime cervical screen (Sc2), and girls' vaccination and 2 lifetime cervical screens (Sc3). Panels show median predictions from the A) *Det_HIV-HPV* and B) *MicroCOSM-HPV* models for South Africa and the C) *DRIVE* model for KZN province. Vaccine coverage = 90%, Vaccine efficacy = 100% against HPV-16/18/31/33/45/52/58, Vaccine duration = Lifetime; Screening = HPV testing, Screening uptake = 45% (2023–2029), 70% (2030–2044), 90% (2045+). Treatment efficacy = 100%, Lost to follow-up = 10%. Scenarios are as described in Table 1.

The predicted temporal dynamics of cervical cancer incidence in the different scenarios were similar across models despite variation in the 2020 age-standardised cervical cancer incidence between the national and KZN models (Fig. 1, Table 2). In all models, HIV prevalence declined over time in the *basecase* scenario due to the expansion of HIV interventions (Fig. T5.1 in technical appendix). However, this alone was insufficient to eliminate cervical cancer, as the predicted age-standardised incidence remained high in 2120 among all women (median: 26.3 per 100,000 women-years and range: 25.3–28.3 across models) and among WLHIV(70.6, 69.4–71.7) (Table 2).

**Table 2:** Main scenarios for all women: Predicted cervical cancer incidence and time of elimination for each threshold among all women, HIV positive and HIV negative women following HPV vaccination and cervical cancer screening.

Scenarios	South Africa (Det_HIV-HPV)					South Africa (MicroCOSM-HPV)					KZN (DRIVE)				
	Age-standardised incidence (per 100,000 women-years) at different time (years)			Year of elimination by thresholds:		Age-standardised incidence (per 100,000 women-years) at different time (years)			Year of elimination by thresholds:		Age-standardised incidence (per 100,000 women-years) at different time (years)			Year of elimination by thresholds:	
	2019^a^ 2045 2060 2120			<4/100,000 <10/100,000 >85% decrease^b^		2019^a^ 2045 2060 2120			<4/100,000 <10/100,000 >85% decrease^b^		2019^a^ 2045 2060 2120			<4/100,000 <10/100,000 >85% decrease^b^	
	ALL	HIV+	HIV-	ALL	HIV+	ALL	HIV+	HIV-	ALL	HIV+	ALL	HIV+	HIV-	ALL	HIV+
Basecase	40.9 [29.6,70.9]	94.7 [66.7,152.6]	25.9 [18.3,47.6]	- [-]	- [-]	47.6 [29.8,71.3]	113.5 [60.2,204.7]	28.0 [13.6,47.4]	- [-]	- [-]	79.2 [55.7,116.3]	173.6 [123.2,245.7]	33.9 [19.4,52.0]	- [-]	- [-]
	34.1 [21.2,60.7]	79.5 [45.1,115.8]	25.1 [17.6,48.4]	- [-]	- [-]	38.5 [23.2,57.0]	75.7 [38.4,126.1]	26.4 [13.9,42.2]	- [-]	- [-]	52.5 [33.1,81.8]	125.5 [77.3,199.2]	29.2 [16.5,48.6]	-[2100,-]	- [-]
	30.1 [18.5,55.8]	72.6 [42.4,110.6]	23.7 [16.4,47.5]	- [-]	- [-]	35.0 [20.9,51.3]	72.6 [35.6,116.1]	27.2 [14.2,41.3]	- [-]	- [-]	39.8 [23.3,64.9]	99.9 [58.8,171.1]	26.0 [14.9,46.5]	- [-]	- [-]
	26.3 [16.0,49.8]	71.7 [40.8,110.8]	22.6 [15.0,45.6]			28.3 [16.2,44.2]	70.6 [34.5,122.5]	27.1 [15.2,43.1]			25.3 [6.0,47.2]	69.4 [19.3,139.8]	20.4 [5.0,40.8]		
Scenario 1:															
Girls' vaccination	40.9 [29.6,70.9]	94.7 [66.7,152.6]	25.9 [18.3,47.6]	- [2081,-]	- [-]	47.6 [29.8,71.3]	113.5 [60.2,204.7]	28.0 [13.6,47.4]	- [2080,-]	- [2083,-]	79.2 [55.7,116.3]	173.6 [123.2,245.7]	33.9 [19.4,52.0]	2095 [2075,-]	- [2104,-]
	29.2 [16.5,49.0]	69.0 [36.9,100.8]	20.1 [13.0,38.2]	2072 [2057,2101]	- [2082,-]	32.4 [20.0,49.5]	63.7 [32.3,107.7]	20.7 [11.1,35.2]	2078 [2062,-]	- [2073,-]	39.0 [23.4,61.2]	99.1 [58.1,159.8]	18.8 [9.9,32.5]	2069 [2058,2092]	2110 [2080,-]
	16.3 [8.4,27.3]	48.4 [22.0,64.8]	12.5 [7.1,22.9]	- [2087,-]	- [2099,-]	19.4 [10.9,30.6]	40.6 [19.9,70.6]	13.6 [6.0,23.3]	- [-]	- [-]	16.2 [8.4,27.9]	47.4 [24.4,87.2]	8.3 [4.4,16.5]	2090 [2076,-]	2098 [2080,-]
	4.5 [2.3,9.6]	14.7 [6.2,27.6]	4.2 [2.1,8.6]			6.3 [2.0,13.0]	17.6 [3.2,43.4]	5.9 [1.7,12.8]			3.2 [0.7,8.4]	9.3 [2.5,27.7]	2.7 [0.6,7.2]		
Scenario 2:															
Girls' vaccination + 1 lifetime screen	40.9 [29.6,70.9]	94.7 [66.7,152.6]	25.9 [18.3,47.6]	2115 [2075,-]	- [-]	47.6 [29.8,71.3]	113.5 [60.2,204.7]	28.0 [13.6,47.4]	- [2077,-]	- [2082,-]	79.2 [55.6,116.1]	173.6 [123.0,245.3]	33.9 [19.3,51.9]	2079 [2066,-]	- [2088,-]
	23.9 [14.1,41.8]	54.2 [29.6,77.6]	17.1 [11.4,33.4]	2068 [2054,2084]	- [2073,-]	28.7 [16.8,43.0]	55.9 [28.0,91.3]	18.0 [8.9,29.5]	2075 [2061,-]	-[2072,-]	28.9 [17.5,44.8]	71.6 [42.4,112.2]	14.8 [7.9,24.9]	2061 [2052, 2073]	2084 [2068,-]
	13.1 [7.1,22.0]	35.8 [15.0,49.6]	10.3 [6.2,18.6]	2119 [2080,-]	- [2087,-]	18.6 [10.7,29.8]	39.4 [20.3,70.4]	12.5 [5.7,21.5]	- [2092,-]	-[2093,-]	10.4 [5.5,17.6]	28.7 [15.3,49.6]	5.7 [3.1,11.0]	2072 [2067,-]	2077 [2069,-]
	3.9 [2.0,8.5]	12.2 [5.4,23.4]	3.5 [1.9,7.6]			5.3 [1.2,10.8]	15.9 [2.7,39.4]	5.1 [1.0,10.7]			2.5 [0.3,6.2]	6.8 [1.0,17.8]	2.1 [0.2,5.3]		
Scenario 3:															
Girls' vaccination + 2 lifetime screens	40.9 [29.6,70.9]	94.7 [66.7,152.6]	25.9 [18.3,47.6]	2094 [2073,-]	- [-]	47.6 [29.8,71.3]	113.5 [60.6,204.7]	28.0 [13.6,47.4]	2093 [2073,-]	- [2078,-]	79.2 [53.6,112.0]	173.6 [119.4,237.6]	33.9 [18.8,50.5]	2067 [2058,-]	- [2076,-]
	23.0 [13.6,40.5]	51.9 [28.4,74.9]	16.6 [11.1,32.5]	2066 [2053,2081]	-[2071,-]	19.5 [11.1,32.3]	39.0 [17.3,70.7]	12.2 [4.9,21.8]	2070 [2050,2088]	- [2069,-]	18.4 [11.4,29.3]	44.5 [27.4,69.3]	10.1 [5.6,17.5]	2054 [2047, 2062]	2069 [2059,-]
	12.3 [6.8,20.7]	33.3 [14.0,46.3]	9.6 [5.9,17.5]	2095 [2078,-]	- [2085,-]	14.3 [7.8,23.8]	30.6 [13.0,56.7]	9.1 [4.0,17.3]	2089 [2083,-]	2099 [2079,-]	5.9 [3.5,10.8]	15.7 [9.2,28.4]	3.5 [1.9,7.3]	2060 [2055, 2066]	2061 [2057,2068]
	3.6 [2.0,8.2]	11.6 [5.3,22.3]	3.2 [1.8,7.3]			3.6 [0.7,8.3]	10.8 [0.6,29.9]	3.3 [0.4,7.9]			1.9 [0.2,5.0]	5.3 [0.5,13.4]	1.6 [0.1,4.4]		

-: Elimination not achieved. The median [and 90% uncertainty interval] of each model is presented.

a Estimates are for the very end of 2019/start of 2020 just before the scale up of intervention starts at the beginning of 2020 – all other estimates are taken at the end of the year.

b Time to reach the 85% incidence reduction determined by the first year when the ratio of the median age-standardised cancer incidence reaches 85%.

**Fig. 2:**
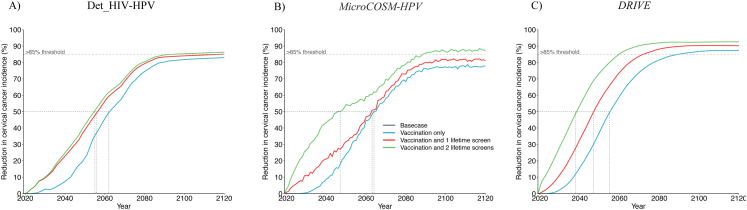
**Relative impact of HPV vaccination and cervical cancer screening for all women – among all women:** Relative decrease in median age standardised cervical cancer incidence among all women over time compared to *basecase* after the ramp-up of girls' vaccination only (Sc1), girls' vaccination and 1 lifetime cervical screen (Sc2), and girls' vaccination and 2 lifetime cervical screens (Sc3). Panels show predictions from the A) *Det_HIV-HPV* and B) *MicroCOSM-HPV* models for South Africa and the C) *DRIVE* model for KZN province. The vertical dotted lines in panels indicate the time when cancer incidence is reduced by 50%. Vaccine coverage = 90%, Vaccine efficacy = 100% against HPV-16/18/31/33/45/52/58, Vaccine duration = Lifetime; Screening = HPV testing, Screening uptake = 45% (2023–2029), 70% (2030–2044), 90% (2045+). Treatment efficacy = 100%, Lost to follow-up = 10%. Scenarios are described in Table 1.

*Among all women*, the models predicted that the median age-standardised cervical cancer incidence would be reduced from 47.6 (40.9–79.2) cases per 100,000 women-years in 2020 to 4.5 (3.2–6.3) in 2120 with girls' vaccination, to 3.9 (2.5–5.3) with vaccination and 1 lifetime cervical screen, and to 3.6 (1.9–3.6) with vaccination and 2 lifetime cervical screens, representing an 83.0% (77.8–87.4%), 85.1% (81.1–90.2%) and 87.3% (86.3–92.6%) decrease in median age standardised incidence by 2120 compared to the *basecase*, respectively (Table 2, Supplement Table S1A, Fig. 1, Fig. 2). However, in the short-term, the decrease in median age-standardised cancer incidence was larger with girls' vaccination and 2 lifetime screens (i.e. WHO's triple-intervention global cervical cancer elimination strategy) than girls' vaccination alone (49.4% (32.6–65.0%) versus 15.8% (14.3–25.8%) by 2045 compared to the *basecase*) (Table 2, Supplement Table S1A, Fig. 2). Compared to the *basecase*, a 50% decrease in median cervical cancer incidence was achieved by 2063 (2056–2064) with girls' vaccination alone, by 2057 (2048–2063) when adding 1 lifetime cervical screen, and by 2046 (2039–2055) when adding 2 lifetime cervical screens (Fig. 2). The uncertainty around the median prediction across models and scenarios is presented in Table 2, Table 3 and Supplement Figs. S1 and S2.

**Table 3:** Enhanced scenarios for WLHIV: Predicted cervical cancer incidence and time of elimination for each threshold among all women, HIV positive and HIV negative women following HPV vaccination and cervical cancer screening.

Scenarios	South Africa (Det_HIV-HPV)					South Africa (MicroCOSM-HPV)					KZN (DRIVE)				
	Age-standardised incidence (per 100,000 women-years) at different time (years)			Year of elimination by thresholds:		Age-standardised incidence (per 100,000 women-years) at different time (years)			Year of elimination by thresholds:		Age-standardised incidence (per 100,000 women-years) at different time (years)			Year of elimination by thresholds:	
	2019^a^ 2045 2060 2120			<4/100,000 <10/100,000 >85% decrease^c^		2019^a^ 2045 2060 2120			<4/100,000 <10/100,000 >85% decrease^c^		2019^a^ 2045 2060 2120			<4/100,000 <10/100,000 >85% decrease^c^	
	ALL	HIV+	HIV-	ALL	HIV+	ALL	HIV+	HIV-	ALL	HIV+	ALL	HIV+	HIV-	ALL	HIV+
Scenario 4:															
Sc1 + vaccination of young WLHIV	40.9 [29.6,70.9]	94.7 [66.7,152.6]	25.9 [18.3,47.6]	- [2080,-]	- [-]	47.6 [29.8,71.3]	113.5 [60.6,204.7]	28.0 [13.6,47.4]	- [2078,-]	- [2083,-]	79.2 [55.7,116.3]	173.6 [123.2,245.7]	33.9 [19.4,52.0]	2093 [2072,-]	- [2101,-]
	28.5 [15.6,46.1]	62.6 [31.7,93.9]	20.0 [12.8,37.5]	2070 [2056,2100]	- [2075,-]	31.4 [18.6,48.9]	60.7 [29.8,103.2]	20.5 [10.9,35.0]	2077 [2060,-]	- [2071,-]	34.5 [20.3,54.1]	84.7 [49.2,135.2]	17.1 [8.8,30.3]	2066 [2055,2090]	2108 [2076,-]
	15.2 [7.8,25.3]	39.4 [17.4,55.2]	12.2 [6.9,22.2]	-[2085,-]	- [2094,-]	18.6 [9.8,30.5]	35.9 [16.8,66.6]	13.4 [5.4,23.5]	- [-]	- [-]	13.6 [7.1,23.9]	37.1 [19.5,68.7]	7.5 [4.0,15.2]	2085 [2071,-]	2094 [2074,-]
	4.5 [2.3,9.6]	14.6 [6.1,27.5]	4.2 [2.1,8.6]			6.6 [2.2,13.2]	17.5 [3.1,43.6]	6.3 [1.8,13.0]			3.2 [0.7,8.4]	9.3 [2.4,27.7]	2.7 [0.6,7.2]		
Scenario 5:															
Sc3 + frequent screening of WLHIV	40.6^b^ [29.3,70.2]	93.8^b^ [65.8,150.5]	25.9 [18.3,47.6]	2089 [2071,-]	- [2106,-]	47.6 [29.8,71.3]	113.5 [60.6,204.7]	28.0 [13.6,47.4]	2092 [2072,-]	- [2077,-]	79.2 [50.7,105.2]	173.6 [113.4,223.6]	33.9 [18.8,50.5]	2062 [2044,-]	2115 [2063,-]
	19.0 [11.8,34.4]	30.3 [14.9,48.4]	16.4 [11.0,31.6]	2062 [2050, 2078]	2096 [2053,-]	16.3 [8.6,27.1]	23.8 [10.8,46.6]	12.1 [5.0,22.7]	2069 [2040,2088]	2080 [2046,-]	13.6 [8.5,23.0]	26.6 [17.1,42.0]	9.5 [5.3,16.7]	2050 [2044,2058]	2059 [2052,-]
	10.8 [6.2,18.4]	20.4 [7.5,30.8]	9.5 [5.8,16.9]	2088 [2076,-]	2092 [2058,-]	12.5 [6.4,21.5]	20.2 [9.6,40.2]	8.8 [3.6,17.1]	2087 [2081,-]	2079 [2075,2094]	4.4 [2.4,8.8]	9.1 [4.9,17.9]	3.2 [1.7,6.9]	2055 [2050,2063]	2051 [2046,2059]
	3.3 [1.9,7.9]	8.5 [3.8,17.0]	3.2 [1.8,7.3]			3.6 [0.5,8.5]	5.2 [0.5,20.2]	3.3 [0.4,8.5]			1.8 [0.1,4.7]	3.9 [0.3, 10.0]	1.6 [0.1,4.3]		
Scenario 6:															
S3 + vaccination of young WLHIV + frequent screening of WLHIV	40.6^b^ [29.3,70.2]	93.8^b^ [65.8,150.5]	25.9 [18.3,47.6]	2087 [2070,-]	- [2102,-]	47.6 [29.8,71.3]	113.5 [60.6,204.7]	28.0 [13.6,47.4]	2091 [2070,-]	- [2075,-]	79.2 [50.7,105.2]	173.6 [113.4,223.6]	33.9 [18.8,50.5]	2060 [2052,-]	2115 [2061,-]
	18.2 [11.3,32.7]	26.9 [13.7,41.9]	16.2 [10.8,30.9]	2061 [2049,2077]	2087 [2051,-]	16.4 [8.5,26.3]	23.4 [9.8,49.0]	12.1 [5.1,21.2]	2068 [2040,2085]	2077 [2045,-]	11.7 [7.2,20.5]	22.4 [14.3,36.0]	8.3 [4.5,15.3]	2047 [2042,2056]	2056 [2049,-]
	10.2 [5.8,17.3]	16.8 [6.9,27.1]	9.3 [5.7,16.4]	2087 [2075,-]	2083 [2056,-]	12.2 [6.1,20.6]	18.4 [8.4,36.4]	8.9 [3.7,17.1]	2087 [2079,-]	2076 [2068,2094]	3.8 [2.1,7.8]	7.5 [4.2,14.7]	2.9 [1.5,6.3]	2052 [2047,2059]	2050 [2044,2055]
	3.3 [1.9,7.9]	8.4 [3.7,16.9]	3.2 [1.8,7.3]			3.6 [0.6,8.3]	5.9 [0.0,20.3]	3.4 [0.5,8.3]			1.8 [0.1,4.7]	3.9 [0.3,10.0]	1.6 [0.1,4.3]		

-: Elimination not achieved.

The median [and 90% uncertainty interval] of each model is presented. The *basecase* scenario is the same as in Table 2.

a Estimates are for the very end of 2019/start of 2020 just before the scale up of intervention starts at the beginning of 2020 – all other estimates are taken at the end of the year.

b The 2020 values are slightly different than for the other scenarios because screening uptake is assumed to increase linearly between 2017 and 2023 to meet the desired targets in 2023 among WLHIV. This does not occur in the other scenarios (e.g. 2 lifetime screening of all women) because uptake of screening is already higher in the *basecase* scenario in 2023 than the desired target for 2023; in this instance screening uptake rate are maintained at their *basecase* values until the date of the next higher screening target is reached.

c Time to reach the 85% incidence reduction determined by the first year when the ratio of the median age-standardised cancer incidence reaches 85%.

Vaccination and 2 lifetime cervical screens was predicted by all models to achieve elimination below the WHO elimination threshold of <4 cases per 100,000 women-years by 2093 (2067–2094) but elimination was not predicted unanimously by all models with girls' vaccination alone, highlighting the importance of cervical cancer screening (Table 2, Fig. 1). Similar results were obtained across models with the alternative >85% incidence decrease threshold. However, cervical cancer incidence could decline to <10/100,000 women-years by 2072 (2069–2078) with girls’ vaccination only, by 2068 (2061–2075) with vaccination and 1 lifetime cervical screen, and by 2066 (2054–2070) with vaccination and 2 lifetime cervical screens.

**Fig. 3:**
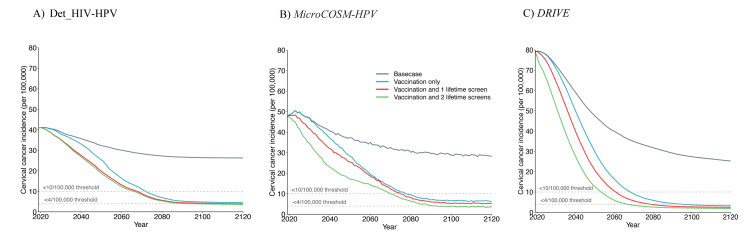
**Temporal dynamics of cervical cancer incidence after HPV vaccination and cervical cancer screening for all women - by HIV and treatment status:** Age standardised cervical cancer incidence per 100,000 women-years among women stratified by HIV and treatment status after HPV girls' vaccination only (Sc1) (A,B,C) and girls' vaccination and 2 lifetime cervical screens (Sc3) (D,E,F). Panels show median predictions from the *Det_HIV-HPV* (A, D) and *MicroCOSM-HPV* (B,E) models of South Africa and the *DRIVE* model of KZN (C,F). Vaccine coverage = 90%, Vaccine efficacy = 100% against HPV-16/18/31/33/45/52/58, Vaccine duration = Lifetime; Screening = HPV testing, Screening uptake = 45% (2023–2029), 70% (2030–2044), 90% (2045+). Treatment efficacy = 100%, Lost to follow-up = 10%. Scenarios are described in Table 1.

*Among WLHIV*, all models predicted substantial declines in median age-standardised cervical cancer incidence from 113.5 (94.7–173.6) per 100,000 women-years in 2020 to 69.0 (63.7–99.1) in 2045 and 14.7 (9.3–17.6) in 2120 with girls' vaccination only and to 44.5 (39.0–51.9) and 10.8 (5.3–11.6) with vaccination and 2 lifetime cervical screens (Fig. 3, Table 2, Supplement Fig. S3). Interestingly, the predicted time trend in cervical cancer incidence among women without HIV in the *basecase* scenario was similar across models. Although WLHIV benefited from similar substantial decreases in median cervical cancer incidence by 2120 (75–92% compared to *basecase*) as HIV negative women (78–92%) across the three main scenarios, none of the models predicted a decline in cervical cancer incidence among WLHIV below <4/100,000 women-years within 100 years (only one model predicted a decline below 10/100,000) with girls’ vaccination and 2 lifetime cervical screens (Supplement Table S1). Differences in cancer incidence between women with and without HIV remained despite HIV treatment and HPV prevention scale-up (Fig. 3, Supplement Table S1, Supplement Fig. S3).

### Impact of enhanced HPV vaccination and screening for WLHIV on cervical cancer incidence (Scenarios 4–6)

**Fig. 4:**
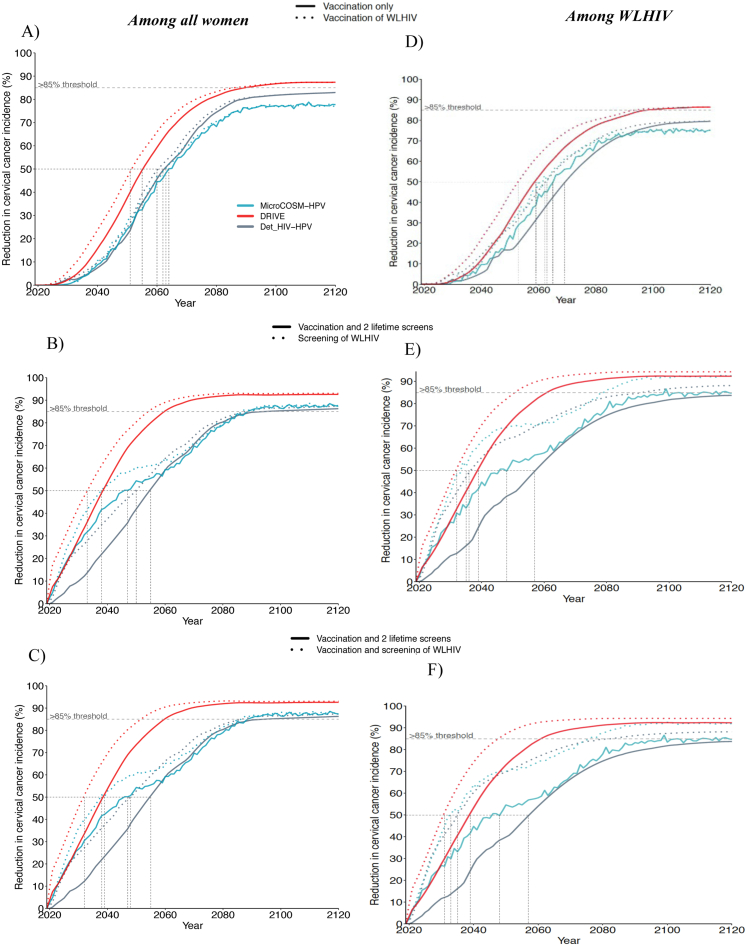
**Incremental impact of enhanced HPV vaccination and cervical cancer screening scenarios for WLHIV – among all women and among WLHIV:** Relative decrease in median age-standardised cervical cancer incidence over time compared to *basecase.* Panels A and D compare girls' vaccination only (Sc1) and girls' vaccination + vaccination of young WLHIV (Sc4). Panels B and E compare girls' vaccination +2 lifetime cervical screens for all women (Sc3) and Sc3+3 yearly screening of WLHIV (Sc5). Panels C and F compare girls' vaccination +2 lifetime cervical screens for all women (Sc3) and scenario 3+vaccination and 3-yearly screening of young WLHIV(Sc6). Panels show predictions from each model among all women (A–C) and among WLHIV (D–F). The vertical dotted lines indicate when cancer incidence is reduced by 50%. Vaccine coverage = 90%, Vaccine efficacy = 100% against HPV-16/18/31/33/45/52/58, Vaccine duration = Lifetime; Screening = HPV testing, Screening uptake = 45% (2023–2029), 70% (2030–2044), 90% (2045+). Treatment efficacy = 100%, Lost to follow-up = 10%. Scenarios are described in Table 1.

*Among all women,* extending vaccination of young WLHIV (aged 15–24 years) to girls' vaccination (aged 9–14 years) was slightly more impactful in the shorter than longer-term (Fig. 4, Table 3, Supplement Fig. S4), decreasing overall median cervical cancer incidence by 3.2% (2.3–11.5%), 6.9% (4.4–16.4%), and 0% (-4.6-0.0%) in 2045, 2060, and 2120 respectively compared to girls' vaccination alone. It did not accelerate elimination because it did not substantially increase long-term overall vaccination coverage compared to girls' vaccination alone (Fig. T5.2 of technical appendix). Adding more frequent cervical screening of WLHIV (Scenario 5) to girls' vaccination and 2 lifetime cervical screens had a more substantial impact, decreasing overall median cervical cancer incidence by 17.3% (16.4–25.9%), 12.6% (12.5–25.6%), and 6.3% (1.0–8.1%) in 2045, 2060, 2120, respectively, compared to girls’ vaccination and 2 lifetime cervical screens. It also accelerated the time to elimination (<4 and < 10 cases per 100,000 women-years) by 7 years or less across models (Table 3, Fig. 4B, Supplement Fig. S4).

Among *WLHIV,* the incremental impact of vaccinating young WLHIV in addition to vaccinating girls was larger than for women overall but still did not increase the likelihood of elimination by 2120. It reduced median cervical cancer incidence in WLHIV to 62.6(60.7–84.7), 37.1(35.9–39.4), and 14.6 (9.3–17.5) per 100,000 women-years by 2045, 2060, and 2120, respectively, corresponding to a 9.3% (4.7–14.5%), 18.7% (11.4–21.7%), and 0.2% (0.0–0.7%) decrease, compared to girls’ vaccination alone (Fig. 4D, Table 3, Supplement Table S1A).

Adding more frequent screening of WLHIV to girls' vaccination and 2 lifetime cervical screens reduced cervical cancer incidence among WLHIV to 26.6(23.8–30.3), 20.2(9.1–20.4) and 5.2 (3.9–8.5) cancer cases per 100,000 women-years in 2045, 2060, and 2120, respectively, corresponding to a 40.1% (38.9–41.7%), 38.9% (34.0–42.0%), and 27.2% (25.8–52.0%) decrease compared to girls' vaccination and 2 lifetime cervical screens (Fig. 4E, Table 3, Supplement Table S1A). This made the elimination to <10/100,000 and the incidence decrease to >85% among WLHIV more likely or earlier (by ∼10–20 years across models) and helped further reduce disparities between women with and without HIV (from an incidence rate ratio (IRR) of 3.3–3.6 with girls’ vaccination and 2 lifetime cervical screens, to 1.7–2.6 in 2120 with more frequent screening of WLHIV) (Supplement Table S1A).

### Cumulative cervical cancer cases averted – scenarios for all women

**Fig. 5:**
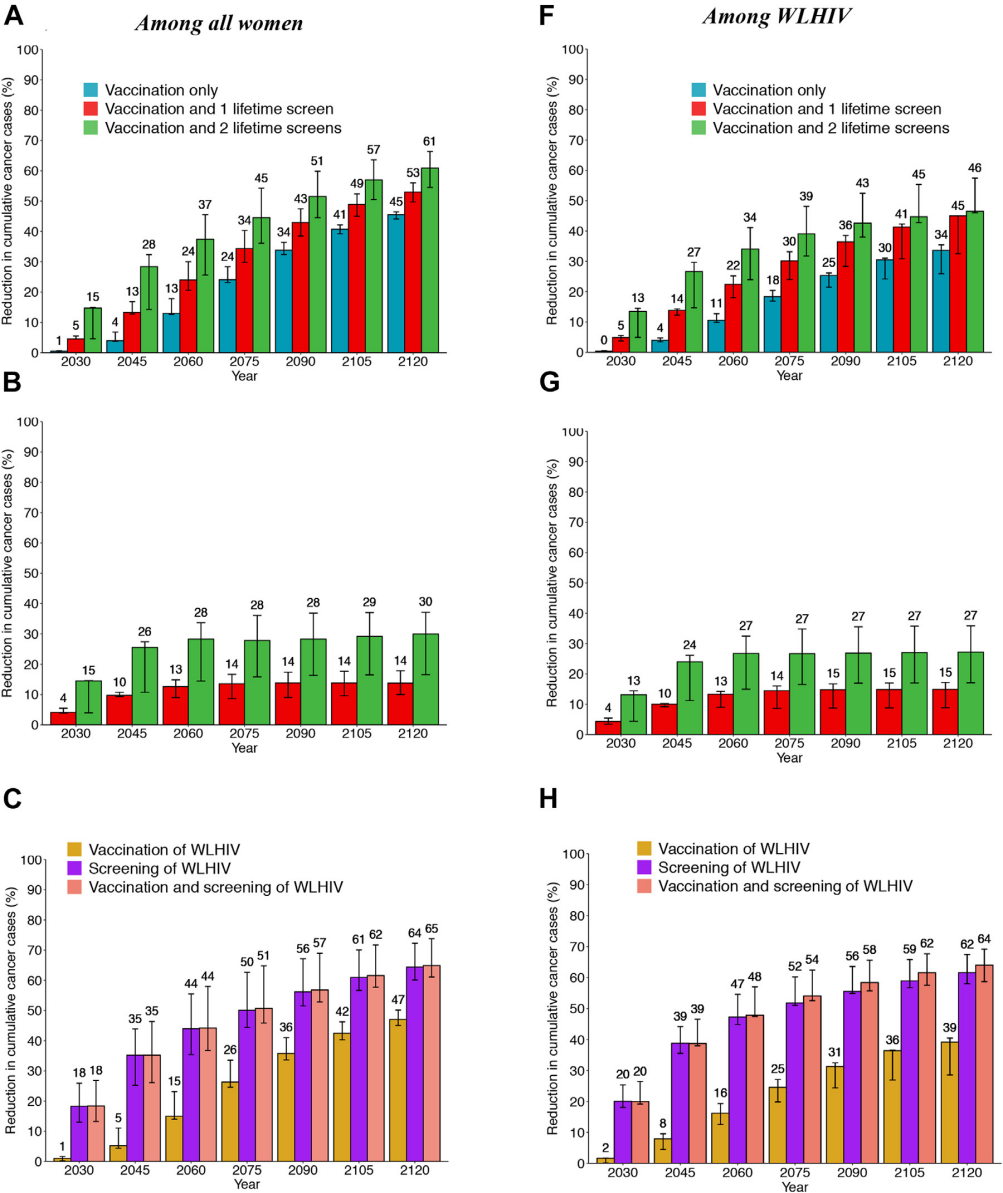

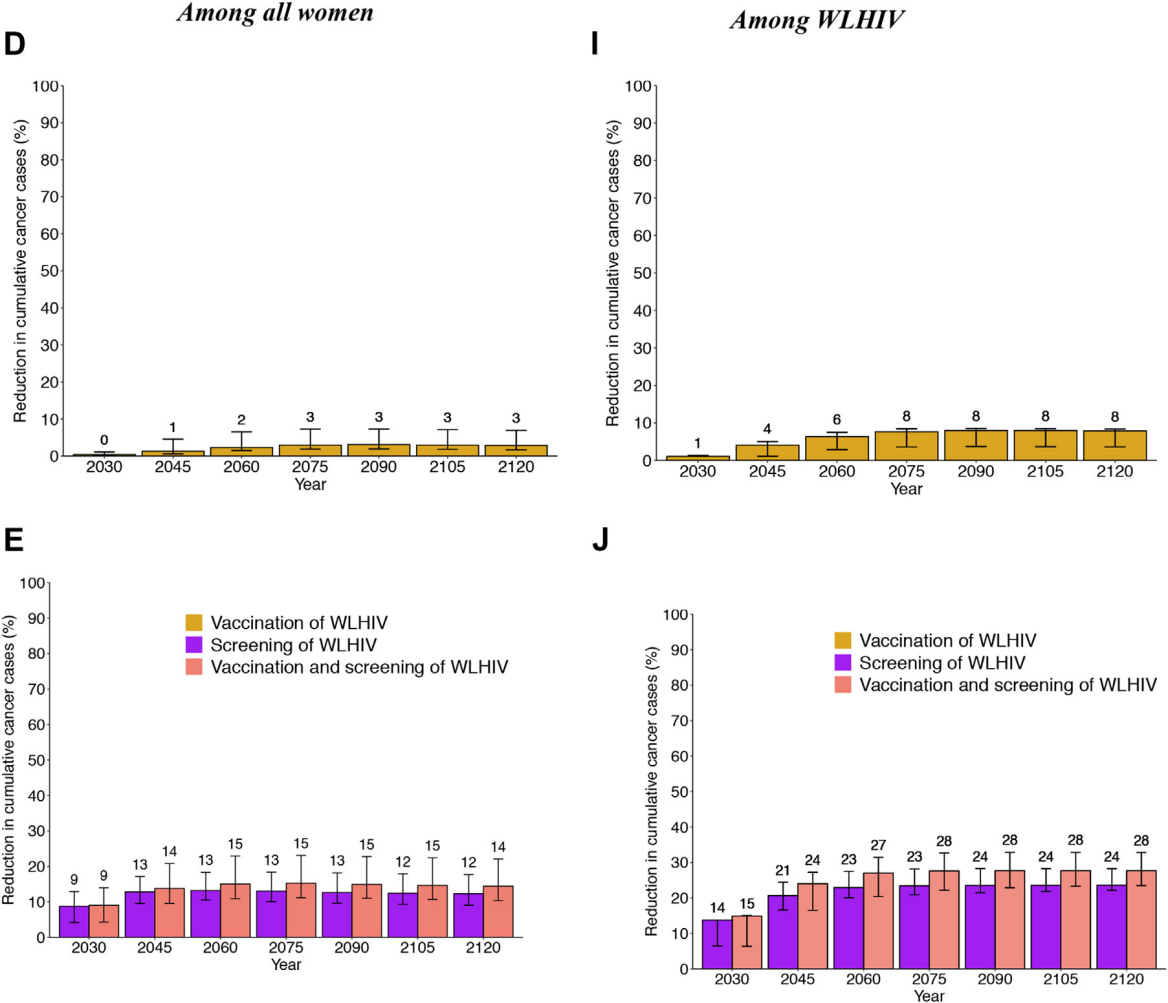
**Cumulative fraction of cervical cancer cases averted after HPV vaccination and cervical cancer screening – among all women and among WLHIV:** Age-standardized fraction of cumulative cervical cancer cases averted over time since 2020 following girls' vaccination only (Sc1), girls' vaccination + 1 lifetime cervical screen (Sc2), girls' vaccination + 2 lifetime cervical screen (Sc3) in panels A–B, F-G, and Sc1 + vaccination of young WLHIV (Sc4), Sc3+ 3-yearly screening of WLHIV (Sc4), or Sc3 + vaccination and 3-yearly screening of WLHIV (sc6) in panels C–E, H-J. Results are presented among all women (A–E) and among WLHIV (F–J). The relevant scenarios are compared to *basecase* (A, C, F, H), girls' vaccination alone (B, D, G, I), and girls' vaccination plus 2 lifetime cervical screens (E, J). The numbers represent the median of the three models. The error bars represent the minimum and maximum of the three models. Vaccine coverage = 90%, Vaccine efficacy = 100% against HPV-16/18/31/33/45/52/58, Vaccine duration = Lifetime; Screening = HPV testing, Screening uptake = 45% (2023–2029), 70% (2030–2044), 90% (2045+). Treatment efficacy = 100%, Lost to follow-up = 10%. Scenarios are as described in Table 1.

*Among all women,* girls' vaccination alone would avert 4.0% (3.8–6.8%), 13.0% (12.7–17.8%), and 45.5% (44.1–46.4%) of age-standardised cumulative cervical cancer cases over 25, 40 and 100 years compared to *basecase,* respectively, whereas girls’ vaccination and 2 lifetime cervical screens would avert 28.4% (14.3–32.3%), 37.4% (25.6–45.5%), and 60.9% (54.5–66.3%) (Fig. 5A).

*Among WLHIV*, the fraction of cumulative cervical cancer cases averted was lower across the three main scenarios than among all women (Fig. 5F). Girls' vaccination alone would avert 4.0% (3.4–4.8%), 10.5% (9.9–12.8%), 33.6% (25.9–35.4%) of cumulative cervical cancer cases over 25, 40 and 100 years compared to *basecase,* respectively. Girls’ vaccination and 2 lifetime cervical screens would avert 26.6% (14.7–29.7%), 34.0% (24.0–41.1%), and 46.5% (46.0–57.4%) of cumulative cervical cancer cases.

Hence, compared to girls’ vaccination alone, adding 2 lifetime cervical screens prevented approximately 25% more cancer cases among women overall and also among WLHIV over 25–100 years (Fig. 5B and G).

### Cumulative cervical cancer cases averted – enhanced scenarios for WLHIV

Compared to girls' vaccination alone (aged 9–14 years), also vaccinating young WLHIV (age 15–24 years) averted a median of 1.3–2.9% additional cumulative cervical cancer cases over 25–100 years among women overall, respectively, and 4.1%–7.9% among WLHIV (Fig. 5D and I). However, screening WLHIV more frequently (3-yearly) in addition to girls’ vaccination and 2 lifetime cervical averted a median of 12-13% additional cancer cases cumulatively over 25 or more years among women overall, and about twice this among WLHIV (21-24%); the incremental impact of also adding young WLHIV vaccination to 3-yearly cervical screening of WLHIV was very modest (∼2% more cumulative cases averted) (Fig. 5E and J).

### Sensitivity analysis

We assessed the sensitivity of model predictions to vaccination coverage, duration of vaccine protection, type of vaccine, additional multi-age cohort vaccination of WLHIV and scale-up of HIV interventions (Supplement Table S1, supplement Figs. S5–S9).

The duration of vaccine protection and number of HPV types included in the vaccine were the two factors explored that most substantially reduced the impact of vaccination and jeopardised the likelihood of reaching elimination within 100 years by any of the three thresholds, even with girls' vaccination and 2 lifetime cervical screens. For example, girls’ vaccination (90% coverage) and 2 lifetime cervical screens decreased median cervical cancer incidence by 57.1% (40.9–84.7%) by 2120 compared to *basecase* if the vaccine protection lasted 20 years (explored by 3 models) and by 61.6–77.7% (explored by 2 models) if the vaccine protected against HPV16 and 18 only for life compared to 87.3% (86.3–92.6%) with the nonavalent vaccine with lifelong protection (Supplement Figs. S6 and S7, Supplement Table S1C and D).

Although reducing vaccination coverage from 90% to 80% had a limited impact on the decrease in cervical cancer incidence in the shorter-term, it could still prevent reaching the 4/100,000 women-years elimination threshold by 2120 with girls’ vaccination and 2 lifetime cervical screens (4.7 (1.9–5.0) per 100,000 woman-years with 80% coverage) (Supplement Fig. S5, Supplement Table S1A and B).

Extending the age of vaccination for WLHIV up to 45 years (explored by 2 models) had a more sizeable short-to medium-term impact than vaccination of WLHIV up to 24 years, decreasing cervical cancer incidence by 7.8–13.3% overall, and by 14.9–18.0% among WLHIV compared to vaccination of young WLHIV up to 24 years (Supplement Fig. S8, Supplement Table 2A and E). Adding vaccination of WLHIV up to age 45 years averted on average 6.1–13.4% and 8.9–15.5% more cumulative cervical cancer cases over a 40-year period or longer than girls’ vaccination alone among women overall and WLHIV, respectively (Supplement Fig. S8C and F).

The influence of not reaching the 90-90-90 UNAIDS and 70% male circumcision targets (by remaining at the 2020 coverage levels) was modest, due to relatively high HIV intervention coverage achieved by 2020, but nevertheless was sufficient to prevent reaching the <4 cases per 100,000 women-years threshold after 100 years with girls’ vaccination and 2 lifetime cervical screens (overall 2120 cancer incidence: 4.8 (3.2–5.1) cases per 100, 000 women-year). However, screening WLHIV every 3 years helped reduce overall cancer incidence below 4/100,000 by 2120 among women overall but not among WLHIV (Supplement Fig. S9, Supplement Table S1G).

## Discussion

Three independent models predicted a substantial decline in age-standardised cervical cancer incidence over time across all scenarios investigated and the achievement of elimination (<4/100,000 women-year) among all women within 100 years in South Africa and in KZN province using the proposed WHO elimination strategy for vaccination, 2 lifetime cervical screens and high treatment coverage.^65^ Although the elimination threshold was approached, it was not met among WLHIV. Extending vaccination for WLHIV up to age 24 and cervical screening for WLHIV every 3 years further reduced incidence in this group (5.9 (3.9–8.4) cases per 100,000 woman-years) closer to the elimination threshold, mainly due to screening WLHIV more frequently. The WHO triple elimination strategy could also have a substantial impact in the short-term, averting approximately one third of cumulative cervical cancer cases among both women overall and WLHIV over the first 25 years and about half or more over 100 years.

Girls' vaccination was necessary but insufficient to achieve elimination. Alone it was predicted to reduce cervical cancer incidence to 4.5 (3.2–6.3) cases per 100,000 women-years overall in 2120, an average 83% decrease compared to the *basecase*. Increasing cervical screening coverage for all women and for WLHIV was more impactful in the short/medium-than longer-term compared to vaccination alone. For example, girls' vaccination alone, girls' vaccination and 2 lifetime cervical screens, and 3-yearly screening of WLHIV in addition to WHO's triple-intervention elimination strategy decreased overall age-standardised cervical cancer incidence on average by 16%, 49%, and 58%, respectively, after 25 years and by 83%, 87%, and 87% after 100 years compared to the *basecase*.

Achieving cervical cancer elimination in a setting such as South Africa, where the predicted 2020 age-standardised cervical cancer incidence per 100,000 woman-years was 40.9–47.6 at the national level and 79.2 in KZN, would be a remarkable achievement. Countries with a high burden of HIV are often those with the highest incidence of cervical cancer, and therefore have much further to go to reach elimination.^1^ However, our results suggest that achieving elimination among WLHIV will be challenging. Despite achieving a >∼85% reduction in cervical cancer incidence in 2120 compared to *basecase,* the WHO triple-intervention strategy did not reduce incidence in WLHIV below the 10 cases/100,000 woman-years threshold. To cross this threshold and reduce disparities in cervical cancer burden between women with and without HIV, more frequent screening of WLHIV will remain critically important. WHO's latest guidelines suggest using a strategy of starting cervical screening at the age of 25 years among WLHIV, with regular screening every 3–5 years using HPV DNA detection for cervical cancer prevention.^65^ Our models predicted that following the WHO evidence-based guidelines,^65^ by adding 3-yearly screening of WLHIV aged 25–49, could reduce incidence to 5.2 (3.9–8.5) cases per 100,000 woman-years by 2120 and avert on average 13% more cumulative cancer cases among women overall and 21% among WLHIV over 25 years compared to the WHO triple-intervention elimination strategy.

The impact of vaccinating WLHIV up to age 24 years was modest, averting less than 8% additional cumulative cancer cases among women overall and among WLHIV, compared to only vaccinating girls 9–14 years of age. This is mainly due to the marginal increase in overall vaccine coverage in the long-term, since most young WLHIV would have been vaccinated through routine vaccination especially when coverage is high, and to some extent previous exposure to HPV types. This is similar to previous studies showing modest long term impact of catch-up vaccination since routine vaccination ultimately determines overall vaccination coverage at equilibrium.^66^^,^^67^ Further extending the vaccination age of WLHIV up to 45 years almost doubled this impact on cumulative cancer cases averted over 40 years or more, providing greater benefits at population-level in the short to medium-term (25–60 years). Even if WLHIV could benefit from vaccination,^10^ the population-level impact of increased age of vaccination for WLHIV faded in the longer-term and did not influence elimination because over time the majority of women would be vaccinated at a young age through routine vaccination before acquiring HIV. However, the incremental population-level benefits of vaccinating WLHIV are expected to be smaller when added to WHO's triple-intervention strategy and frequent screening of WLHIV.

Our results are consistent with the previous CCEMC model comparison study in 78 LMIC, which concluded that elimination of cervical cancer below the 4/100,000 women-years threshold was not possible with girls’ vaccination alone in countries with age-standardised cancer incidence above 25 cases per 100,000 women-years, such as in South Africa, and required 2 lifetime cervical screens.^29^ Even if HPV vaccination eradicated HPV types 16, 18, 31, 33, 45, 52, and 58, this would not totally eliminate cervical cancer risk since at least 10% of cervical cancers cases are due to HPV types that are not prevented by the available HPV vaccines and because antiretroviral therapy does not completely eliminate the excess cancer risk associated with HIV.^32^^,^^33^ Similar to the previous CCEMC model results of LMICs with the highest cervical cancer incidence (e.g., Uganda), our sensitivity analysis also suggested that vaccine protection lasting at least 20 years and broad-spectrum protection against HPV types 16, 18, 31, 33, 45, 52, 58 will be needed to achieve elimination in South Africa. The age-standardised cervical cancer incidence remained above the 10/100,000 women-years threshold when the duration of protection was reduced from lifelong to 20 years (2 out of 3 models) and above the 4/100,000 women-years threshold for a vaccine that only protected against HPV-16 and HPV-18 (2 out of 2 models).

Although our impact predictions were less sensitive to a 10% drop in vaccine coverage (from 90% to 80%) or failure to reach UNAIDS 90-90-90 targets and 70% male circumcision coverage, these could still jeopardise the chance of reaching the <4 cases per 100,000 women-years threshold (but not the <10 cases per 100,000 threshold) with the WHO triple-intervention elimination strategy among all women. This suggests that frequent screening of WLHIV will be even more important to reach cervical cancer elimination if UNAIDS 90-90-90 targets are not reached or, conversely, that scaling-up HIV treatment and VMMC may, to a certain extent, help reduce cervical cancer and reach elimination, especially in countries where HIV intervention coverage is low.

Our analysis has some limitations. As we predicted over a time horizon of 100 years, potential demographic, behavioural and or epidemiological changes and technological developments could impact future cervical cancer incidence trends and impact estimates. Our predictions may be conservative since improvements in vaccines (e.g. with broader HPV type protection and/or cross-protection) and/or screening and treatment modalities (e.g. therapeutic vaccines)^68^, ^69^, ^70^ or improvements in HIV prevention would help accelerate elimination. Furthermore, there was uncertainty in predictions of each model due to parameter and structural assumptions, which is partly reflected by variation across models.

The use of age-standardised incidence should limit the influence of demographic changes unless the age distribution of cervical cancer is more substantially altered than assumed. Our predictions may also be optimistic given the ambitious assumptions on HPV vaccination and cervical cancer screening and treatment scale-up, efficacy, and coverage, which will be challenging to achieve in reality. While slower scale-up would postpone the time of elimination and reduce the fraction of cervical cancer cases and deaths averted, achieving lower coverage of vaccination, screening and HIV interventions would also reduce the likelihood of elimination. The impact of cervical screening could be lower than predicted if treatment success was lower in WLHIV than women without HIV.^65^^,^^71^^,^^72^

The overall impact of vaccination could be lower than predicted if HPV vaccine efficacy and duration of protection, or if rollout and coverage achieved among WLHIV, was lower than for women not living with HIV. Although no large randomized efficacy trials of HPV vaccination have been done among people living with HIV, our recent comprehensive systematic reviews of the evidence from 18 safety and immunogenicity studies consistently demonstrated a good immune response against all HPV vaccine types among people with HIV of all genders and ages, which support the assumption that they may be effective at preventing infections and disease among people with HIV.^10^ A pragmatic relative evaluation of the effectiveness of South Africa national vaccination programs (HOPE) that is currently ongoing will provide important additional data on the effectiveness of vaccinating young WLHIV with single and 2 HPV vaccine doses.^73^ The overall impact of vaccination in South Africa could be larger than predicted by the model with a vaccine that only protect against HPV-16 and HPV-18 (without cross protection) since the bivalent vaccine currently used in South Africa national HPV vaccination program (Cervarix), provide some levels of cross protection against additional vaccine types.^57^^,^^74^ Nevertheless, the impact of WHO's triple-intervention elimination strategy, and the likelihood of reaching elimination will be reduced using the bivalent vaccine.

Finally, our results may also depend on the limited estimates of cervical cancer incidence, especially for KZN, which could have been over- or underestimated. The two South African models were calibrated to either plausible Globocan cervical cancer estimates^,^ (extrapolated from recent trends in incidence obtained from national or subnational population-based cancer registries)^39^^,^^62^ or to cervical cancer diagnosis,^40^^,^^63^ which produced similar age standardised cervical cancer incidence predictions. Given the lack of data, the KZN model estimated incidence from Globocan estimates^39^^,^^62^ information on cervical cancer risk by HIV status, and relative HIV prevalence, which produced cervical cancer incidence estimates similar to those assumed for Eswatini in the previous CCEMC model comparison.^29^ However, this is unlikely to affect our conclusions since results were consistent across the three models despite large variation in initial cervical cancer incidence and HIV prevalence in 2020. Nevertheless, population-based cancer surveillance in many sub-Saharan African countries should be strengthened to improve cervical cancer incidence estimates overall and by HIV status to better inform local cancer control strategies and to monitor progress toward cervical cancer elimination.

Our study also has several strengths. By explicitly simulating co-circulating and interacting HIV and HPV infections and diseases, and HIV intervention scale-up, we addressed one important limitation of the previous CCEMC model comparison.^29^ We were able to assess the impact of the same three main cervical cancer prevention scenarios for all women as well as new enhanced strategies for WLHIV on cervical cancer incidence among women overall and among WLHIV. These highlighted the importance of frequent screening of WLHIV in addition to the proposed WHO triple-intervention elimination strategy to help control cervical cancer overall and to reduce disparities between WLHIV and women without HIV. Current empirical evidence suggests that HIV treatment does not completely eliminate the excess HPV-related disease progression and cervical cancer risk associated with HIV, and that it depends on CD4 at initiation and duration on ART.^32^ Nevertheless, as HIV treatment is scaled-up, a further decline in HIV prevalence is expected over time, indirectly contributing to cervical cancer prevention.

By comparing three independent models calibrated to South African data at the national level and KZN province, we produced predictions that accounted for variation in parameter and structural assumptions as well as varied epidemiological contexts, allowing to generalize results to other high HIV prevalence settings in sub-Saharan Africa if WHO's and UNAIDS targets are achieved. However, results may vary across settings depending on the success of scale up of both cervical cancer and HIV prevention interventions in real life. It would therefore be useful to replicate the model comparison analysis in additional settings. Of note, despite additional model complexity and some model differences in parameters associated with HIV infection and treatment and its interactions with HPV infections and disease progression, the three models produced consistent impact results and conclusions and predicted similar trends in age-standardised cervical cancer incidence among women without HIV over time. By providing more nuanced results by HIV status, our study helps to support and validate the WHO triple-intervention global cervical cancer strategy needed to achieve elimination of cervical cancer as a public health problem globally, and for countries highly burdened by HIV in particular.^28^

In conclusion, our comparative modelling analysis suggests that cervical cancer elimination as a public health problem is possible in South Africa by the end of the century. To achieve elimination under the proposed threshold of four or fewer cancer cases per 100,000 women-years, in South Africa and other high HIV prevalence settings, both high HPV vaccination coverage and screening uptake will also be necessary. Importantly, more frequent screening of WLHIV as recommended in WHO's most recent guidelines on screening and treatment for the prevention of cervical cancer will be necessary to reduce cervical cancer incidence below 10 cancer cases per 100,000 women-years and decrease the large disparities in cervical cancer burden between women with and without HIV. Considerable international commitment will be required to achieve WHO's triple-intervention targets by 2030, particularly in countries with the highest burden of both cervical cancer and of HIV, where scale-up of vaccination and cervical screening resources are most urgently needed.

## Contributors

MCB, RVB, MMR, CJB, DWR, MB, and WHO co-authors (SD, NB, SG, PB) contributed to the study design. MMR, CJB, CV carried out the modelling analysis. NS, DWR, LS, GL, RS helped with secondary data analysis and literature reviews to inform the model. NS, MMR, CJB, CV, MCB analysed the model results and generated figures and tables. MCB wrote the first version of the manuscript with the contribution from RVB and SD. MCB, RVB, SD, MMR, CV, CJB, NS had full access to all the data in the study, interpreted results, and revised the first manuscript draft. All co-authors critically revised the versions of the manuscript and help interpret results. MCB, RVB and SD had final responsibility for the decision to submit for publication.

## Data sharing statement

Further information on the data is available upon request from the first author (MCB).

## Declaration of interests

RVB reports grants from the Bill and Melinda Gates Foundation (BMGF), the 10.13039/100000002National Institutes of Health (10.13039/100000002NIH), and manuscript and abstract writing support from Regeneron Pharmaceuticals outside the submitted work. MMR reports funding from Harvard Data Science Institute and travel support to attend meetings for cervical cancer elimination from the WHO and Canadian Institute of Health Research, all outside of the submitted work. CJB did a Graduate Research Assistantship with Merck & Co., Inc. after and outside of the submitted work. GL started working from Merck in March 2022 and detains Merck stock/stock options. MB received funding from the 10.13039/100012016Institut National de Santé Publique du Québec for other work. DWR reports additional NIH, USAID and WHO salary support for unrelated work. LS received funding from 10.13039/100014588Sanofi Pasteur for projects outside the scope of this manuscript. PB, SG, NS, RS, LFJ, SG, and NB declare no competing interests.

## Appendix A Supplementary data

Supplement Tables S1

Technical Appendix

Model specific _Technical Appendix _T1

Model specific _Technical Appendix _T2

Model specific _Technical Appendix _T3

Captions for Supplement material
