# Supplementary material for: Estimating the effect of HIV on cervical cancer elimination in South Africa: Comparative modelling of the impact of vaccination and screening
Source: eClinicalMedicine. 2022 Nov 17;54:101754. doi: 10.1016/j.eclinm.2022.101754 (PMC9793279; doi:10.1016/j.eclinm.2022.101754)

Supplement figure S1. Uncertainty in model predictions of the impact of HPV vaccination and cervical screening for all women - among all women: Predicted age-standardised cervical cancer incidence over time among all women for the *basecase* scenario and for A) girls' vaccination (Sc1) and B) girls' vaccination and 2 lifetime cervical screens (Sc3) introduced in 2020. Solid line and shaded area represent the median and 90% uncertainty interval for each model, respectively. *Det_HIV-HPV* and *MicroCOSM-HPV* are models of South Africa and the *DRIVE* model if for KZN.

Vaccine coverage=90%, Vaccine efficacy=100% against HPV16/18/31/33/45/52/58, Vaccine duration=Lifetime; Screening=HPV testing, Screening uptake= 45% (2023-2029), 70% (2030-2044), 90% (2045+). Treatment efficacy=100%, Lost to follow-up=10%.

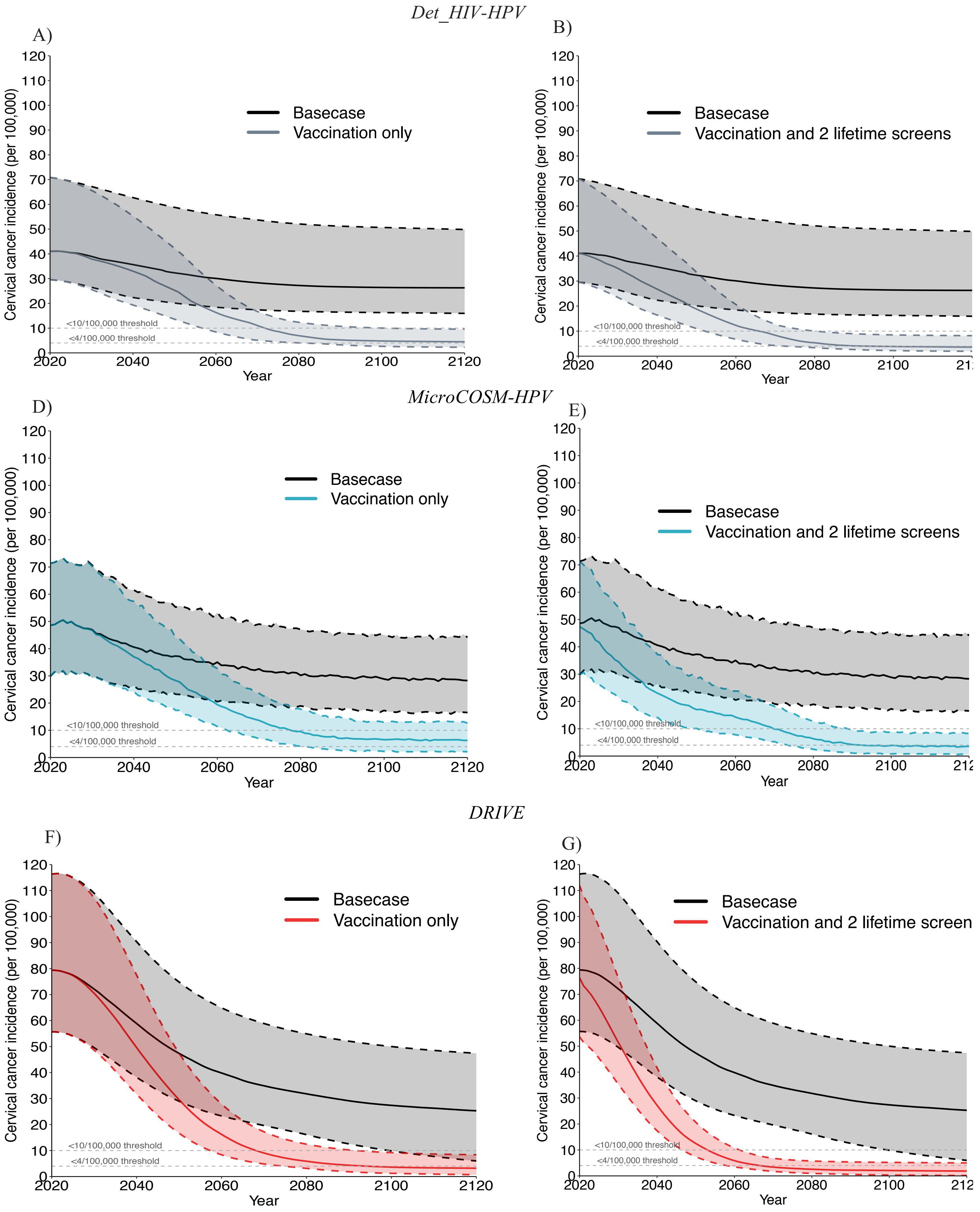

Supplement: Supplement Figure S1 [file mmc1.pdf]
