# Supplementary material for: Estimating the effect of HIV on cervical cancer elimination in South Africa: Comparative modelling of the impact of vaccination and screening
Source: eClinicalMedicine. 2022 Nov 17;54:101754. doi: 10.1016/j.eclinm.2022.101754 (PMC9793279; doi:10.1016/j.eclinm.2022.101754)

Supplement figure S3. Relative impact of HPV vaccination and cervical screening for all women - by HIV and treatment status: Predicted relative decrease in median age-standardised cervical cancer incidence compared to *basecase* stratified by HIV and treatment status after girls' vaccination (Sc1) (A,B,C), and girl's vaccination and 2 lifetime cervical screens (Sc3) (D,E,F) introduced in 2020. Panels show median predictions from the *Det_HIV-HPV* (A,D) and *MicroCOSM-HPV* (B,E) models and the *DRIVE* model (C,F).

Vaccine coverage=90%, Vaccine efficacy=100% against HPV16/18/31/33/45/52/58, Vaccine duration=Lifetime; Screening=HPV testing, Screening uptake= 45% (2023-2029), 70% (2030-2044), 90% (2045+). Treatment efficacy=100%, Lost to follow-up=10%.

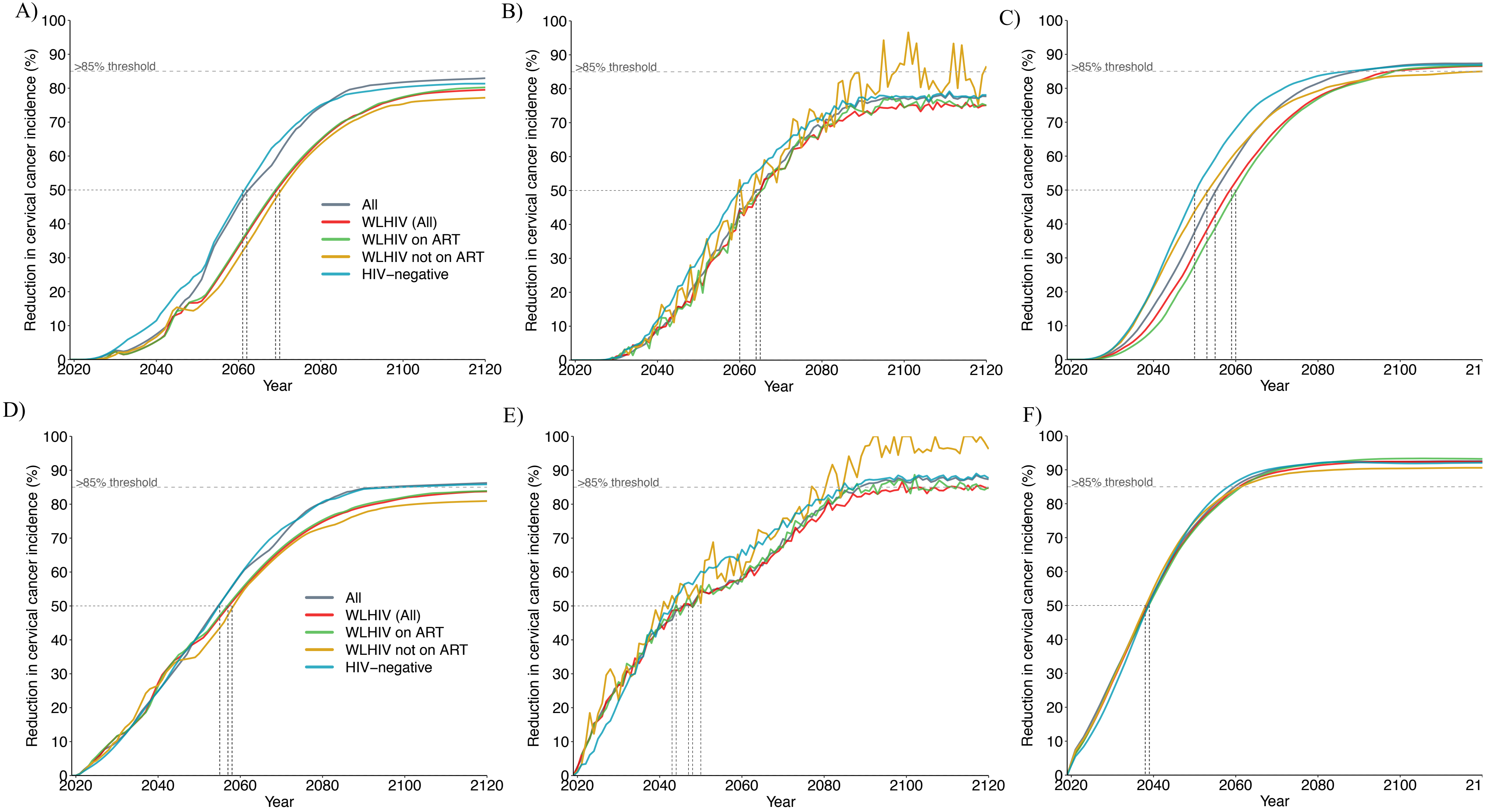

Supplement figure S4. **Temporal dynamics and relative impact of HPV vaccination and cervical screening for all women and for WLHIV – among all women:** Predicted age-standardised cervical cancer incidence per 100,000 women-years (A,B,C) among all women in the *basecase* scenarios and after girls vaccination (Sc1), vaccination and 2 lifetime screens (Sc3), vaccination and 2 lifetime screens + vaccination of young WLHIV (Sc 4), or + frequent screening of WLHIV (Sc5), or + vaccination of young WLHIV and frequent screening of WLHIV (Sc 6) introduced in 2020. Relative decrease in median age-standardised cervical cancer incidence among all women compared to *basecase* (D,E,F) after HPV vaccination and screening ramp-up introduced in 2020 (Scenarios Sc1, Sc3-6). Panels show median predictions from the *Det_HIV-HPV* (A,D) and *MicroCOSM-HPV* (B,E) models and the *DRIVE* model (C,F).

Vaccine coverage=90%, Vaccine efficacy=100% against HPV16/18/31/33/45/52/58, Vaccine duration=Lifetime; Screening=HPV testing, Screening uptake= 45% (2023-2029), 70% (2030-2044), 90% (2045+). Treatment efficacy=100%, Lost to follow-up=10%.

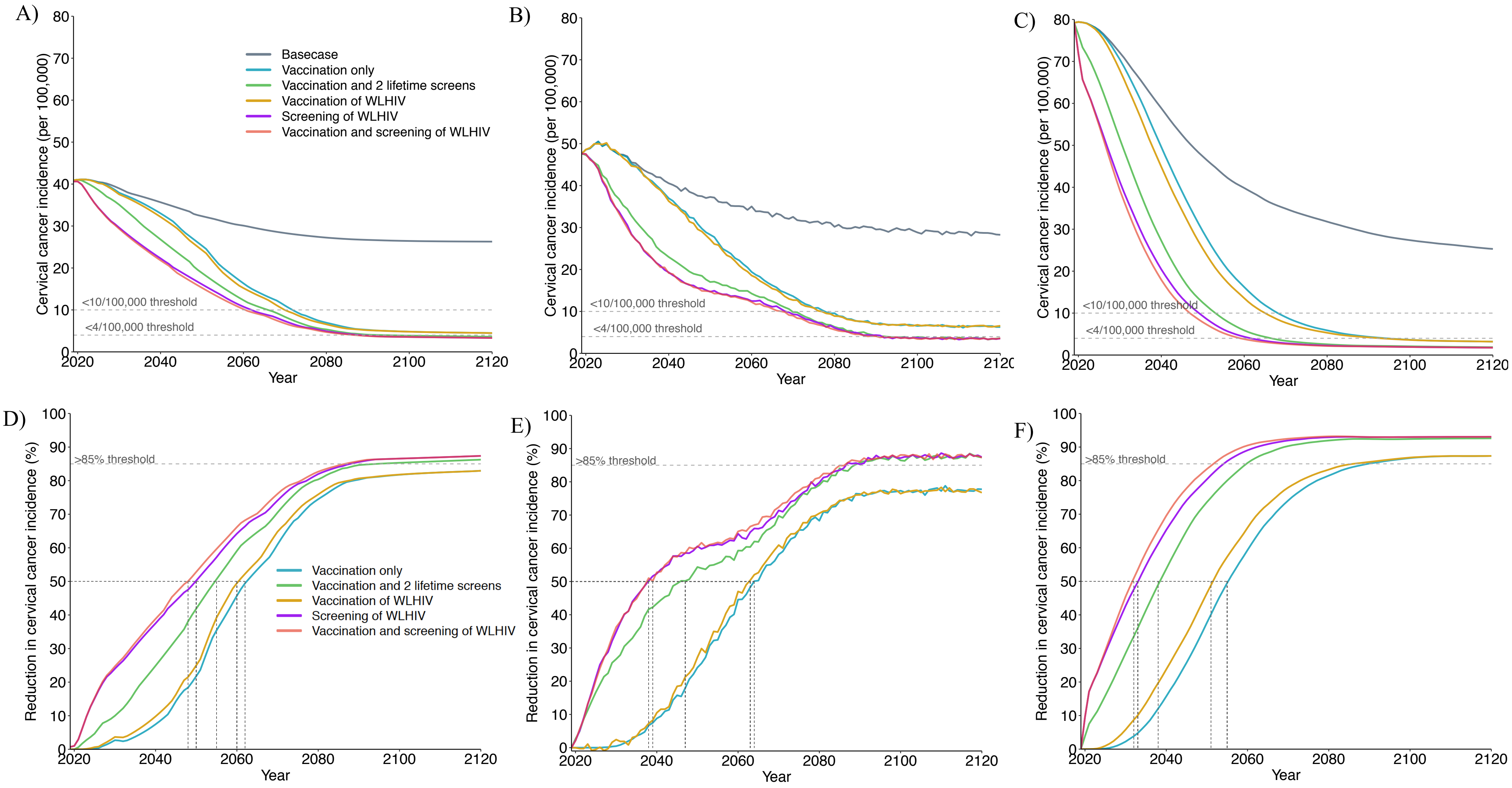

Supplement: Supplement Figures S3-S4 [file mmc3.pdf]
