# Supplementary material for: Estimating the effect of HIV on cervical cancer elimination in South Africa: Comparative modelling of the impact of vaccination and screening
Source: eClinicalMedicine. 2022 Nov 17;54:101754. doi: 10.1016/j.eclinm.2022.101754 (PMC9793279; doi:10.1016/j.eclinm.2022.101754)

Supplement figures S5. Sensitivity analysis to HPV vaccination coverage (80% versus 90%) - among all women: Predicted age-standardised cervical cancer incidence per 100,000 women-years (A-C) among all women and relative decrease in median age-standardised cervical cancer incidence compared to *basecase* (D-F) following the introduction of girls' vaccination (Sc1), girls' vaccination and 2 lifetime cervical screens (Sc3), and girls' vaccination and vaccination of young WLHIV (Sc4). Panels show median prediction from the *Det_HIV-HPV* (A,D) and *MicroCOSM-HPV* (B,E) models and the *DRIVE* model (C,F).

Vaccine coverage=90% or 80%, Vaccine efficacy=100% against HPV16/18/31/33/45/52/58, Vaccine duration=Lifetime.

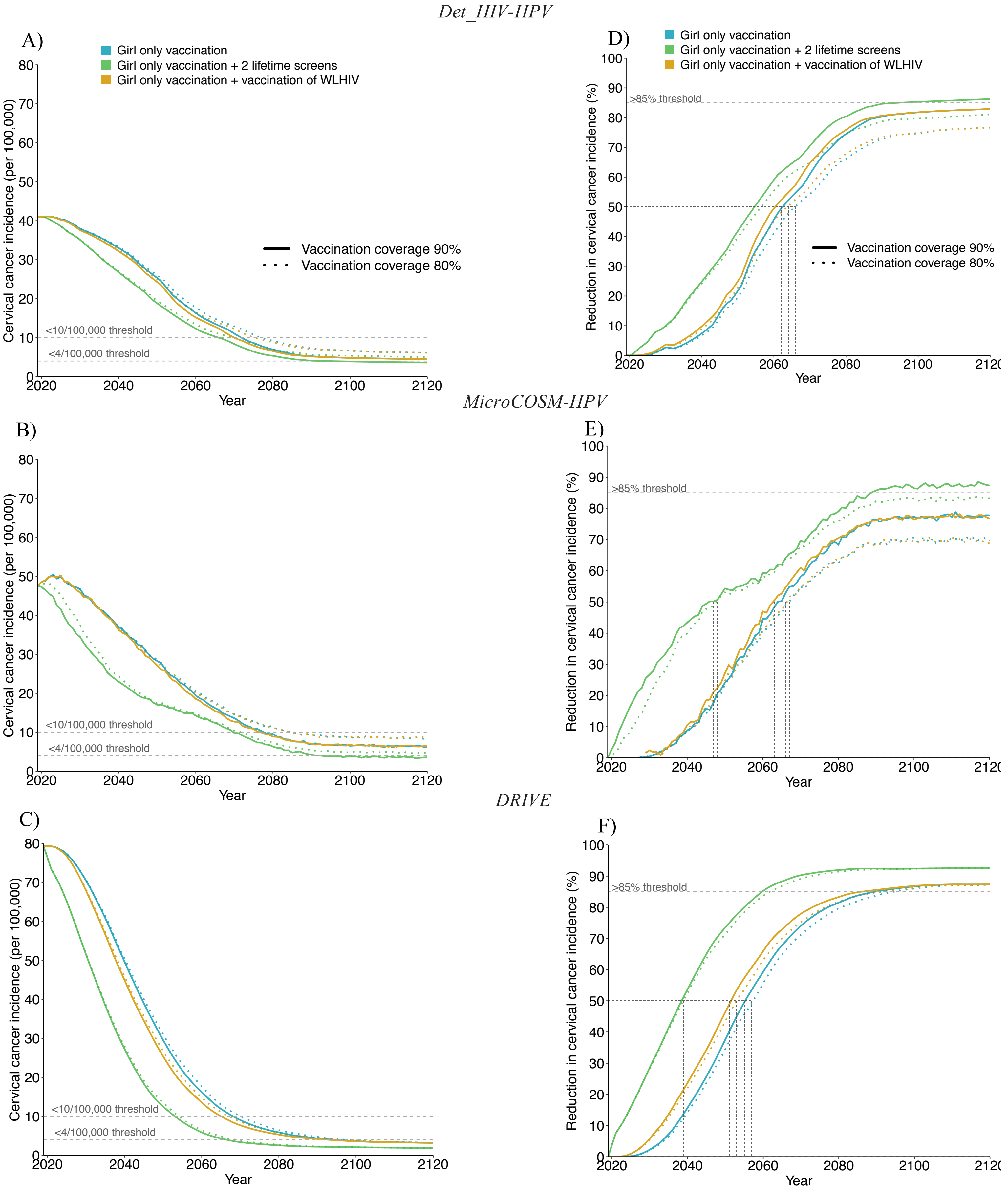

Supplement figures S6. **Sensitivity analysis to the duration of vaccine protection (20 years versus lifelong) – among all women**: Predicted age-standardised cervical cancer incidence per 100,000 women-years (A-C) among all women in the *basecase* scenario and following the introduction of girls' vaccination (Sc1) and girls' vaccination and 2 lifetime cervical screens (Sc3). Relative decrease in median age-standardised cervical cancer incidence compared to *basecase* (D-F) following the introduction of girls' vaccination (Sc1) and girls' vaccination and 2-lifetime cervical screens (Sc3). Plain and dotted lines assume lifelong and 20-years vaccine protection, respectively. Panels show median predictions from the *Det_HIV-HPV* (A,D) and *MicroCOSM-HPV* (B,E) models and the *DRIVE* model (C,F).
Vaccine coverage=90%, Vaccine efficacy=100% against HPV16/18/31/33/45/52/58, Vaccine duration=Lifetime or 20 years.

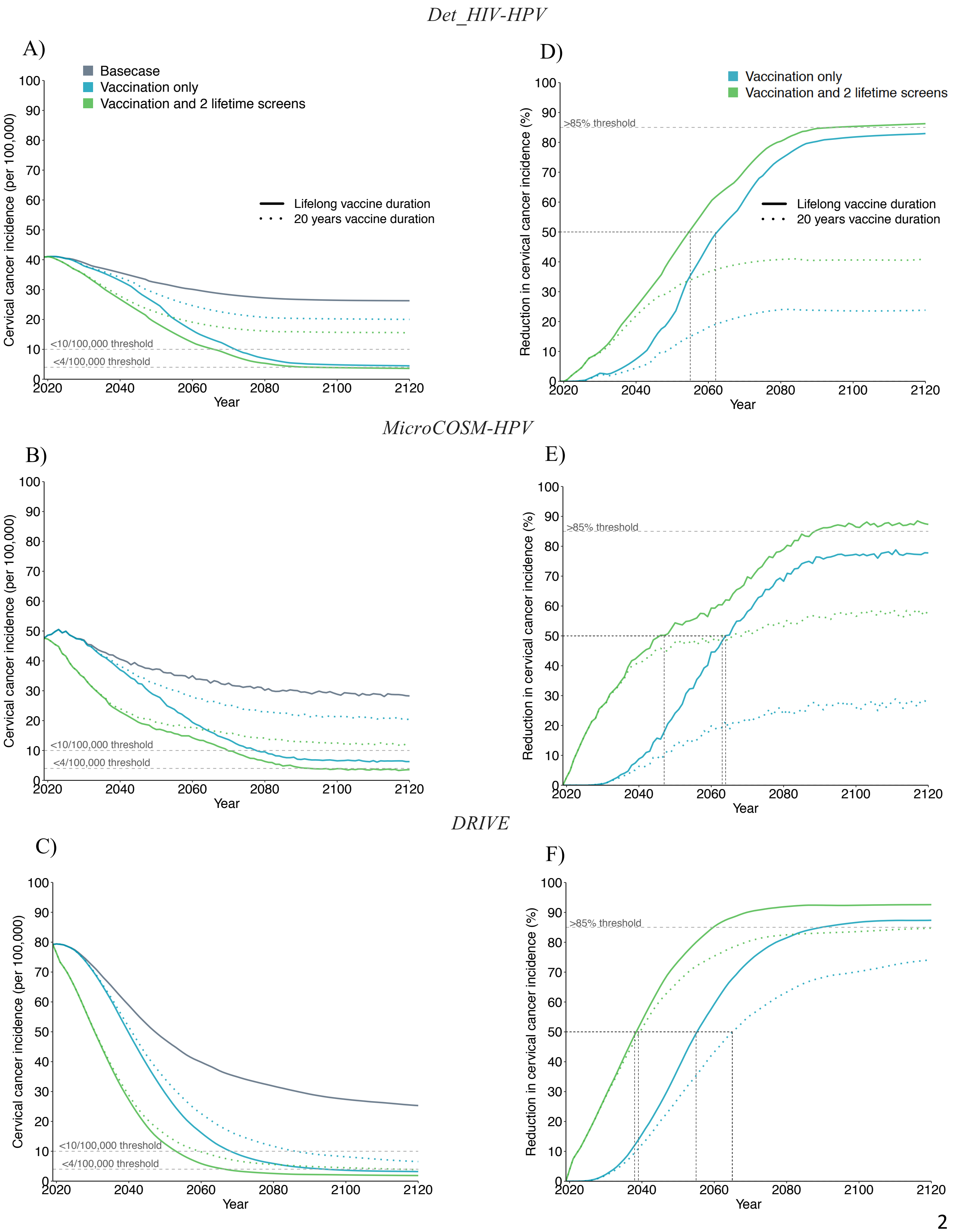

Supplement figures S7. Sensitivity analysis to the vaccine type (nonavalent versus bivalent) – among all women: Predicted age-standardised cervical cancer incidence per 100,000 women-years (A,B) among all women and relative decrease in median age-standardised cervical cancer incidence compared to *basecase* (C,D) following the introduction of girls' vaccination (Sc1) and girls' vaccination and 2 lifetime cervical screens (Sc3). Panels show median predictions from the two models (Det_HIV-HPV (A,C), MicroCOSM-HPV (B,D)) that could model bivalent vaccination.
Vaccine coverage=90%, Vaccine efficacy=100% against HPV16/18/31/33/45/52/58 or against 16/18 only, Vaccine duration=Lifetime.

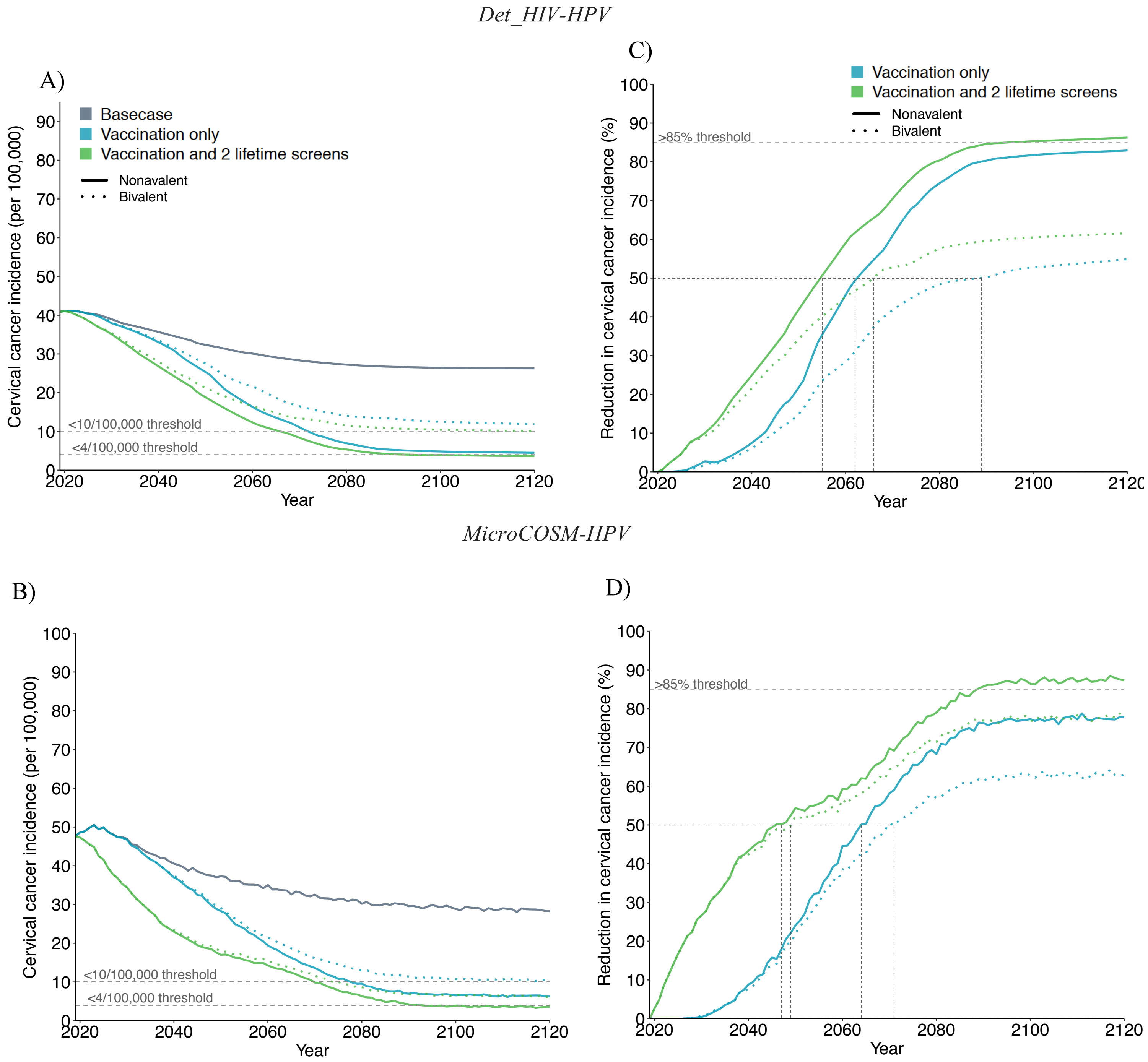

Supplement figure S8. Sensitivity analysis to the age eligibility of vaccination for WLHIV (15-24 years, 15-45 years old, All) – impact among all women: Predicted age-standardised cervical cancer incidence per 100,000 women-years of girls' vaccination and vaccination of WLHIV among all women (A,D), relative decrease in median age-standardised cervical cancer incidence compared to *basecase* (B,E), and age-standardised fraction of cumulative cervical cancer cases averted since 2020 compared to girls' vaccination alone (C,F). Panels show median predictions from the two models that could represent these scenarios (MicroCOSM-HPV (A-C), DRIVE (D-F)).
Vaccine coverage=90%, Vaccine efficacy=100% against HPV16/18/31/33/45/52/58, Vaccine duration=Lifetime.

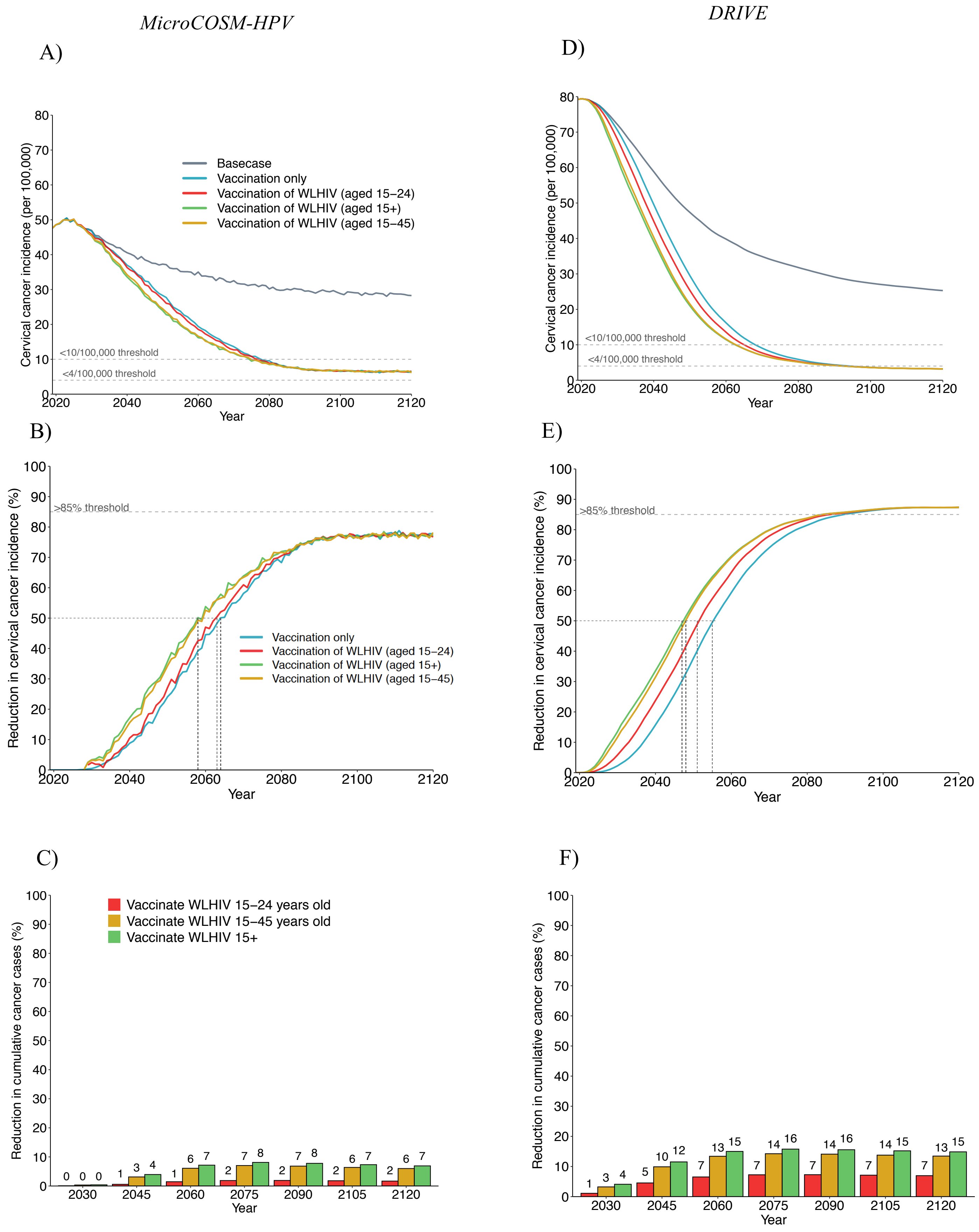

Supplement figure S8 (continue). Sensitivity analysis to the age eligibility of vaccination for WLHIV (15-24 years old, 15-45 years old, All) – impact among WLHIV: Median predicted age-standardised cervical cancer incidence per 100,000 women-years among WLHIV (G,J), relative decrease in median age-standardised cervical cancer incidence compared to *basecase* (H,K), and age-standardised fraction of cumulative cervical cancer cases averted since 2020 compared to girls' vaccination (Sc1) (I,L). Panels show median predictions from the two models that could represent these scenarios (MicroCOSM-HPV (G-I), DRIVE (J-L)).

Vaccine coverage=90%, Vaccine efficacy=100% against HPV16/18/31/33/45/52/58, Vaccine duration=Lifetime.

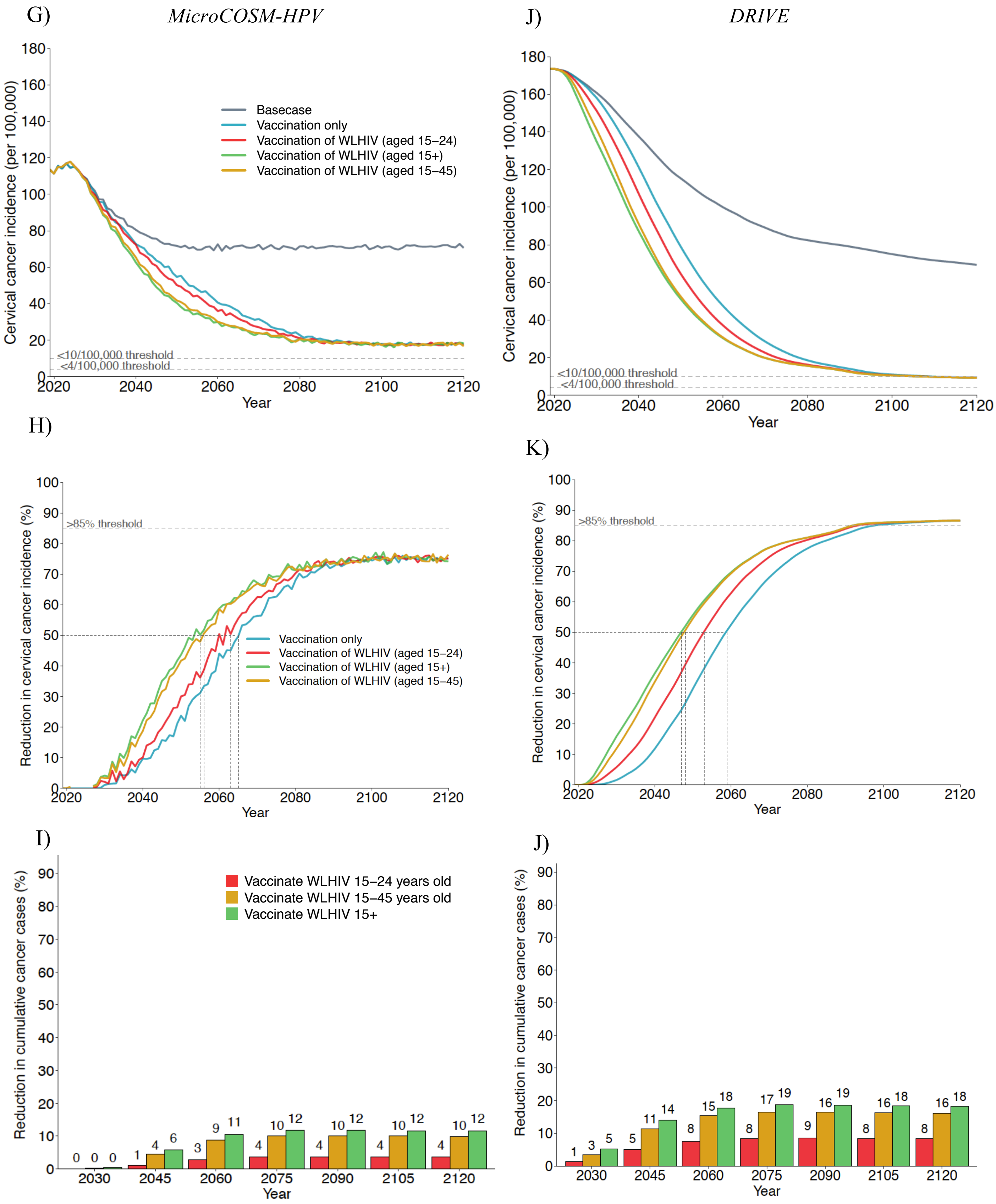

Supplement figures S9. Sensitivity analysis to ART scale-up (reaching UNAIDS 90-90-90 and 70% male circumcision targets versus not reaching it) – among all women: Predicted age-standardized cervical cancer incidence per 100,000 women-years in all women (A-C) and relative decrease in median age-standardised cervical cancer incidence compared to *basecase* when UNAIDS 90-90-90 targets are not reached (D-F). Median predictions are presented for each model ((Det_HIV-HPV (A,D), MicroCOSM-HPV (B,E), DRIVE (C,F)).

Vaccine coverage=90%, Vaccine efficacy=100% against HPV16/18/31/33/45/52/58, Vaccine duration=Lifetime, UNAIDS 90-90-90 HIV care and treatment and 70% male circumcision reached by 2030 or when not reach HIV treatment and male circumcision remain at 2020 levels.

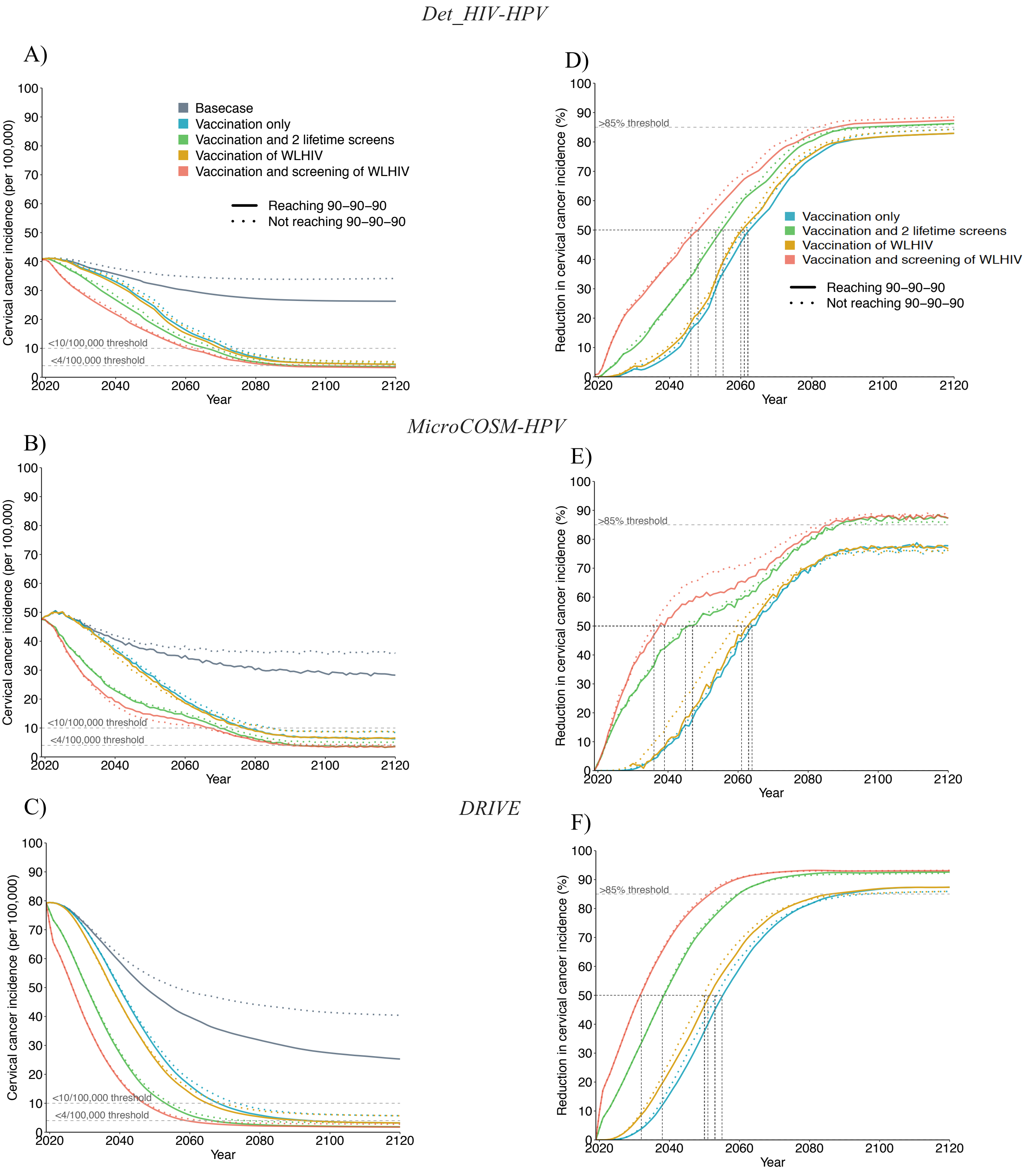

Supplement: Supplement Figures S4-S9 [file mmc4.pdf]
