# Supplementary material for: Estimating the effect of HIV on cervical cancer elimination in South Africa: Comparative modelling of the impact of vaccination and screening
Source: eClinicalMedicine. 2022 Nov 17;54:101754. doi: 10.1016/j.eclinm.2022.101754 (PMC9793279; doi:10.1016/j.eclinm.2022.101754)
Supplement: Supplement Tables S1 [file mmc5.docx]

**Supplement table S1**. **Sensitivity analysis**: Cervical cancer incidence among all women and women stratified by HIV status following HPV vaccination and cervical cancer screening and predicted time of elimination for two thresholds. The median prediction of each model is presented.

|  | **South Africa (Det_HIV-HPV)** | | | | | | **South Africa (MicroCOSM-HPV)** | | | | | | **KZN (DRIVE)** | | | | | |
| --- | --- | --- | --- | --- | --- | --- | --- | --- | --- | --- | --- | --- | --- | --- | --- | --- | --- | --- |
|  | Age standardised CC incidence  (per 100,000 women) | | | | Year of elimination  by threshold | | Age standardised CC incidence  (per 100,000 women) | | | | Year of elimination  by threshold | | Age standardised CC incidence  (per 100,000 women) | | | | Year of elimination  by threshold | |
| Scenarios | Time:  2019/2020^”^  2045  2060  2120 | | | | <4/100,000  <10/100,000 | | Time:  2019/2020^”^  2045  2060  2120 | | | | <4/100,000  <10/100,000 | | Time:  2019/2020^”^  2045  2060  2120 | | | | <4/100,000  <10/100,000 | |
|  | HIV+ | HIV - | ALL | IRR | HIV+ | ALL | HIV+ | HIV - | ALL | IRR | HIV+ | ALL | HIV+ | HIV - | ALL | IRR | HIV+ | ALL |
| A) Vaccination coverage - 90% (lifetime duration of vaccine protection, nonavalent vaccine) | | | | | | | | | | | | | | | | | | |
| *Basecase* | 94.7  79.5  72.6  71.7 | 25.9  25.1  23.7  22.6 | 40.9  34.1  30.1  26.3 | 3.7  3.2  3.1  3.2 | -  - | -  - | 113.5  75.7  72.6  70.6 | 28.0  26.4  27.2  27.1 | 47.6  38.5  35.0  28.3 | 4.1  2.9  2.7  2.6 | -  - | -  - | 173.6  125.5  99.9  69.4 | 33.9  29.2  26.0  20.4 | 79.2  52.5  39.8  25.3 | 5.1  4.3  3.8  3.4 | -  - | -  - |
| Sc1:  Girls’ vaccination | 94.7  69.0  48.4  14.7 | 25.9  20.1  12.5  4.2 | 40.9  29.2  16.3  4.5 | 3.7  3.4  3.9  3.5 | -  - | -  2072 | 113.5  63.7  40.6  17.6 | 28.0  20.7  13.6  5.9 | 47.6  32.4  19.4  6.3 | 4.1  3.1  3.0  3.0 | -  - | -  2078 | 173.6  99.1  47.4  9.3 | 33.9  18.8  8.3  2.7 | 79.2  39.0  16.2  3.2 | 5.1  5.3  5.7  3.4 | -  2110 | 2095  2069 |
| Relative decrease^*^ S1 vs *basecase* | 0%  13%  33%  80% | 0%  20%  47%  81% | 0%  14%  46%  83% | na | na | na | 0%  16%  44%  75% | 0%  22%  50%  78% | 0%  16%  45%  78% | na | na | na | 0%  21%  53%  87% | 0%  36%  68%  87% | 0%  26%  59%  87% | na | na | na |
| Sc2 : Girls’ vaccination + 1 lifetime screen | 94.7  54.2  35.8  12.2 | 25.9  17.1  10.3  3.5 | 40.9  23.9  13.1  3.9 | 3.7  3.2  3.5  3.5 | -  - | 2115  2068 | 113.5  55.9  39.4  15.9 | 28.0  18.0  12.5  5.1 | 47.6  28.7  18.6  5.3 | 4.1  3.1  3.2  3.1 | -  - | -  2075 | 173.6  71.6  28.7  6.8 | 33.9  14.8  5.7  2.1 | 79.2  28.9  10.4  2.5 | 5.1  4.8  5.0  3.2 | -  2084 | 2079  2061 |
| Relative decrease^*^ Sc2 vs *basecase* | 0%  32%  51%  83% | 0%  32%  57%  85% | 0%  30%  56%  85% | na | na | na | 0%  26%  46%  78% | 0%  32%  54%  81% | 0%  25%  47%  81% | na | na | na | 0%  43%  71%  90% | 0%  49%  78%  90% | 0%  45%  74%  90% | na | na | na |
| Sc3: Sc1+ 2 lifetime screens | 94.7  51.9  33.3  11.6 | 25.9  16.6  9.6  3.2 | 40.9  23.0  12.3  3.6 | 3.7  3.1  3.5  3.6 | -  - | 2094  2066 | 113.5  39.0  30.6  10.8 | 28.0  12.2  9.1  3.3 | 47.6  19.5  14.3  3.6 | 4.1  3.2  3.4  3.3 | -  - | 2093  2070 | 173.6  44.5  15.7  5.3 | 33.9  10.1  3.5  1.6 | 79.2  18.4  5.9  1.9 | 5.1  4.4  4.5  3.3 | -  2069 | 2067  2054 |
| Relative decrease^*^ Sc3 vs *basecase* | 0%  35%  54%  84% | 0%  34%  59%  86% | 0%  33%  59%  86% | na | na | na | 0%  48%  58%  85% | 0%  54%  67%  88% | 0%  49%  59%  87% | na | na | na | 0% 65%  84%  92% | 0%  65%  86%  92% | 0%  65%  85%  93% | na | na | na |
| Sc4: Sc1+ vaccination of young WLHIV | 94.7  62.6  39.4  14.6 | 25.9  20.0  12.2  4.2 | 40.9  28.5  15.2  4.5 | 3.7  3.1  3.2  3.5 | -  - | -  2070 | 113.5  60.7  35.9  17.5 | 28.0  20.5  13.4  6.3 | 47.6  31.4  18.6  6.6 | 4.1  3.0  2.7  2.8 | -  - | -  2077 | 173.6  84.7  37.1  9.3 | 33.9  17.1  7.5  2.7 | 79.2  34.5  13.6  3.2 | 5.1  5.0  4.9  3.4 | -  2108 | 2093  2066 |
| Relative decrease^*^ Sc4 vs *basecase* | 0%  21%  46%  80% | 0%  20%  49%  81% | 0%  16%  49%  83% | na | na | na | 0%  20%  50%  75% | 0%  22%  51%  77% | 0%  18%  47%  77% | na | na | na | 0%  32%  63%  87% | 0%  42%  71%  87% | 0%  34%  66%  87% | na | na | na |
| Sc5: Sc3 + frequent screening of WLHIV | 93.8^^^  30.3  20.4  8.5 | 25.9  16.4  9.5  3.2 | 40.6^^^  19.0  10.8  3.3 | 3.6  1.8  2.1  2.7 | -  2096 | 2089  2062 | 113.5  23.8  20.2  5.2 | 28.0  12.1  8.8  3.3 | 47.6  16.3  12.5  3.6 | 4.1  2.0  2.3  1.6 | -  2080 | 2092  2069 | 173.6  26.6  9.1  3.9 | 33.9  9.5  3.2  1.6 | 79.2  13.6  4.4  1.8 | 5.1  2.8  2.8  2.4 | 2115  2059 | 2062  2050 |
| Relative decrease^*^ Sc5 vs *basecase* | 1%  62%  72%  88% | 0%  34%  60%  86% | 1%  44%  64%  87% | na | na | na | 0%  69%  72%  93% | 0%  54%  68%  88% | 0%  58%  64%  87% | na | na | na | 0%  79%  91%  94% | 0%  68%  88%  92% | 0%  74%  89%  93% | na | na | na |
| Sc6: S3 + vaccination of young WLHIV + frequent screening of WLHIV | 93.8^^^  26.9  16.8  8.4 | 25.9  16.2  9.3  3.2 | 40.6^^^  18.2  10.2  3.3 | 3.6  1.7  1.8  2.6 | -  2087 | 2087  2061 | 113.5  23.4  18.4  5.9 | 28.0  12.1  8.9  3.4 | 47.6  16.4  12.2  3.6 | 4.1  1.9  2.1  1.7 | -  2077 | 2091  2068 | 173.6  22.4  7.5  3.9 | 33.9  8.3  2.9  1.6 | 79.2  11.7  3.8  1.8 | 5.1  2.7  2.6  2.4 | 2115  2056 | 2060  2047 |
| Relative decrease^*^ Sc6 vs *basecase* | 1%  66%  77%  88% | 0%  35%  61%  86% | 1%  47%  66%  87% | na | na | na | 0%  69%  75%  92% | 0%  54%  67%  87% | 0%  57%  65%  87% | na | na | na | 0%  82%  92%  94% | 0%  71%  89%  92% | 0%  78%  90%  93% | na | na | na |
| B) Vaccination coverage - 80% (lifetime duration of vaccine protection, nonavalent vaccine) | | | | | | | | | | | | | | | | | | |
| Sc1: Girls’ vaccination | 94.7  70.0  51.7  21.9 | 25.9  20.6  13.7  5.7 | 40.9  29.7  17.7  6.1 | 3.7  3.4  3.8  3.8 | -  - | -  2077 | 113.5  63.6  43.3  23.6 | 28.0  21.1  14.3  8.1 | 47.6  33.0  20.6  8.6 | 4.1  3.0  3.0  2.9 | -  NA | -  2085 | 173.6  101.2  50.7  9.9 | 33.9  19.7  9.3  2.7 | 79.2  40.1  17.7  3.2 | 5.1  5.1  5.5  3.7 | -  2120 | 2098  2071 |
| Relative decrease^*^ Sc1 vs *basecase* | 0%  12%  29%  69% | 0%  18%  42%  75% | 0%  13%  41%  77% | na | na | na | 0%  16%  40%  67% | 0%  20%  47%  70% | 0%  14%  41%  70% | na | na | na | 0%  19%  49%  86% | 0%  33%  64%  87% | 0%  24%  56%  87% | na | na | na |
| Sc3: Sc1+ 2 lifetime screens | 94.7  52.8  35.9  17.7 | 25.9  16.9  10.6  4.3 | 40.9  23.3  13.4  5.0 | 3.7  3.1  3.4  4.1 | -  - | -  2070 | 113.5  39.3  30.0  14.3 | 28.0  12.3  9.4  4.5 | 47.6  20.1  14.6  4.7 | 4.1  3.2  3.2  3.2 | -  - | -  2072 | 173.6  46.2  17.1  5.4 | 33.9  10.7  4.0  1.6 | 79.2  19.1  6.5  1.9 | 5.1  4.3  4.3  3.4 | -  2072 | 2069  2055 |
| Relative decrease^*^ Sc3 vs *basecase* | 0%  34%  51%  75% | 0%  33%  55%  81% | 0%  32%  55%  81% | na | na | na | 0%  48%  59%  80% | 0%  53%  65%  84% | 0%  48%  58%  83% | na | na | na | 0%  63%  83%  92% | 0%  63%  85%  92% | 0%  64%  84%  93% | na | na | na |
| Sc4: Sc1+ vaccination of young WLHIV | 94.7  64.6  43.3  21.9 | 25.9  20.4  13.4  5.7 | 40.9  29.1  16.5  6.1 | 3.7  3.2  3.2  3.8 | -  - | -  2076 | 113.5  61.6  39.8  23.5 | 28.0  21.0  14.5  8.4 | 47.6  32.1  19.7  8.8 | 4.1  2.9  2.7  2.8 | -  - | -  2085 | 173.6  87.3  39.9  9.8 | 33.9  18.0  8.3  2.7 | 79.2  35.6  14.7  3.2 | 5.1  4.9  4.8  3.6 | 2096  2067 | -  2118 |
| Relative decrease^*^ Sc4 vs *basecase* | 0%  19%  40%  69% | 0%  19%  43%  75% | 0%  15%  45%  77% | na | na | na | 0%  19%  45%  67% | 0%  20%  46%  69% | 0%  17%  44%  69% | na | na | na | 0%  30%  60%  86% | 0%  38%  68%  87% | 0%  32%  63%  87% | na | na | na |
| C) Duration of vaccine protection – 20 years (90% coverage, nonavalent vaccine) | | | | | | | | | | | | | | | | | | |
| Sc1: Girls’ vaccination | 94.7  74.9  65.1  62.0 | 25.9  22.1  19.7  17.9 | 40.9  31.4  24.6  20.0 | 3.7  3.4  3.3  3.5 | -  - | -  - | 113.5  67.2  58.3  57.7 | 28.0  22.5  20.9  19.7 | 47.6  35.8  28.1  20.5 | 4.1  3.0  2.8  2.9 | -  - | -  - | 173.6  105.0  62.4  18.4 | 33.9  21.1  13.0  5.2 | 79.2  42.0  22.6  6.5 | 5.1  5.0  4.8  3.5 | -  - | -  2087 |
| Relative decrease^*^ Sc1 vs *basecase* | 0%  6%  10%  13% | 0%  12%  17%  21% | 0%  8%  18%  24% | na | na | na | 0%  11%  20%  18% | 0%  15%  23%  27% | 0%  10%  20%  27% | na | na | na | 0%  16%  38%  73% | 0%  28%  50%  75% | 0%  20%  43%  74% | na | na | na |
| Sc3: Sc1+ 2 lifetime screens | 94.7  55.7  48.3  46.4 | 25.9  18.5  15.3  13.8 | 40.9  24.8  19.1  15.5 | 3.7  3.0  3.2  3.4 | -  - | -  - | 113.5  42.0  36.4  35.5 | 28.0  13.4  11.8  11.4 | 47.6  21.0  17.4  12.1 | 4.1  3.1  3.1  3.1 | -  - | -  - | 173.6  49.5  23.7  8.5 | 33.9  11.7  6.0  3.1 | 79.2  20.8  9.8  3.9 | 5.1  4.2  4.0  2.7 | -  2102 | 2115  2060 |
| Relative decrease^*^ Sc3 vs *basecase* | 0%  30%  33%  35% | 0%  26%  35%  39% | 0%  27%  37%  41% | na | na | na | 0%  44%  50%  50% | 0%  49%  57%  58% | 0%  45%  50%  57% | na | na | na | 0%  61%  76%  88% | 0%  60%  77%  85% | 0%  60%  75%  85% | na | na | na |
| Sc4: Sc1+ vaccination of young WLHIV | 94.7  72.2  62.2  62.0 | 25.9  22.0  19.7  17.9 | 40.9  31.0  24.2  20.0 | 3.7  3.3  3.2  3.5 | -  - | -  - | 113.5  67.7  57.9  56.5 | 28.0  22.5  20.9  19.6 | 47.6  34.8  27.9  20.5 | 4.1  3.0  2.8  2.9 | -  - | -  - | 173.6  88.8  45.8  13.6 | 33.9  19.1  10.6  3.8 | 79.2  37.0  17.7  5.0 | 5.1  4.6  4.3  3.6 | -  - | -  2075 |
| Relative decrease^*^ Sc4 vs *basecase* | 0%  9%  14%  13% | 0%  12%  17%  21% | 0%  9%  20%  24% | na | na | na | 0%  10%  20%  20% | 0%  15%  23%  28% | 0%  10%  20%  27% | na | na | na | 0%  29%  54%  80% | 0%  35%  59%  81% | 0%  29%  55%  80% | na | na | na |
| Sc5: Sc3 + frequent screening of WLHIV | 93.8^^^  35.2  31.1  32.5 | 25.9  18.4  15.2  13.6 | 40.6^^^  20.9  16.9  14.7 | 3.6  1.9  2.0  2.4 | -  - | -  - | 113.5  24.0  21.3  20.4 | 28.0  13.4  12.3  11.4 | 47.6  17.8  15.8  11.7 | 4.1  1.8  1.7  1.8 | -  - | -  - | 173.6  30.5  15.1  6.4 | 33.9  10.9  5.5  2.7 | 79.2  15.7  7.4  3.1 | 5.1  2.8  2.7  2.4 | -  2076 | 2093  2053 |
| Relative decrease^*^ Sc5 vs *basecase* | 1%  56%  57%  55% | 0%  27%  36%  40% | 1%  39%  44%  44% | na | na | na | 0%  68%  71%  71% | 0%  49%  55%  58% | 0%  54%  55%  59% | na | na | na | 0%  76%  85%  91% | 0%  63%  79%  87% | 0%  70%  81%  88% | na | na | na |
| Sc6: S3 + vaccination of young WLHIV + frequent screening of WLHIV | 93.8^^^  33.5  30.2  32.5 | 25.9  18.3  15.2  13.6 | 40.6^^^  20.7  16.8  14.7 | 3.6  1.8  2.0  2.4 | -  - | -  - | 113.5  24.0  21.0  19.9 | 28.0  13.4  12.2  11.1 | 47.6  17.7  15.6  11.4 | 4.1  1.8  1.7  1.8 | -  - | -  - | 173.6  24.9  10.7  5.4 | 33.9  9.4  4.3  2.0 | 79.2  13.2  5.6  2.5 | 5.1  2.6  2.5  2.7 | -  2062 | 2072  2050 |
| Relative decrease^*^ Sc6 vs *basecase* | 1%  58%  58%  55% | 0%  27%  36%  40% | 1%  39%  44%  44% | na | na | na | 0%  68%  71%  72% | 0%  49%  55%  59% | 0%  54%  55%  60% | na | na | na | 0%  80%  89%  92% | 0%  68%  82%  90% | 0%  75%  86%  90% | na | na | na |
| D) Type of vaccine – bivalent (Duration of vaccine protection – lifelong, 90% coverage) | | | | | | | | | | | | | | | | | | |
| Sc1: Girls’ vaccination | 94.7  71.7  53.4  33.0 | 25.9  21.8  16.4  11.0 | 40.9  30.6  21.5  11.9 | 3.7  3.3  3.3  3.0 | -  - | -  - | 113.5  64.5  45.1  29.8 | 28.0  21.4  15.1  10.0 | 47.6  33.2  21.5  10.5 | 4.1  3.0  3.0  3.0 | -  - | -  - | RNA | RNA | RNA | RNA | RNA | RNA |
| Relative decrease^*^ Sc1 vs *basecase* | 0%  10%  26%  54% | 0%  13%  31%  51% | 0%  10%  28%  55% | na | na | na | 0%  15%  38%  58% | 0%  19%  44%  63% | 0%  14%  39%  63% | na | na | na | RNA | RNA | RNA | na | RNA | RNA |
| Sc3: Sc1+ 2 lifetime screens | 94.7  53.3  40.8  28.1 | 25.9  18.2  13.0  9.3 | 40.9  24.5  16.5  10.1 | 3.7  2.9  3.1  3.0 | -  - | -  - | 113.5  39.9  33.0  18.7 | 28.0  12.6  9.9  6.1 | 47.6  20.1  15.2  6.3 | 4.1  3.2  3.3  3.1 | -  - | -  2076 | RNA | RNA | RNA | RNA | RNA | RNA |
| Relative decrease^*^ Sc3 vs *basecase* | 0%  33%  44% 61% | 0%  27%  45%  59% | 0%  28%  45%  62% | na | na | na | 0%  47%  55%  74% | 0%  52%  63%  78% | 0%  48%  57%  78% | na | na | na | RNA | RNA | RNA | na | RNA | RNA |
| Sc4: Sc1+ vaccination of young WLHIV | 94.7  67.0  49.5  33.0 | 25.9  21.6  16.3  11.0 | 40.9  29.7  20.5  11.9 | 3.7  3.1  3.0  3.0 | -  - | -  - | 113.5  52.9  38.0  29.1 | 28.0  21.5  14.8  10.2 | 47.6  29.7  19.6  10.5 | 4.1  2.5  2.6  2.9 | -  - | -  - | RNA | RNA | RNA | RNA | RNA | RNA |
| Relative decrease^*^ Sc4 vs *basecase* | 0%  16%  32%  54% | 0%  14%  32%  51% | 0%  13%  32%  55% | na | na | na | 0%  30%  48%  59% | 0%  19%  45%  63% | 0%  23%  44%  63% | na | na | na | RNA | RNA | RNA | na | RNA | RNA |
| Sc5: Sc3 + frequent screening of WLHIV | 93.8^^^  33.6  26.8  21.7 | 25.9  18.1  13.0  9.3 | 40.6^^^  20.1  14.5  9.8 | 3.6  1.9  2.1  2.3 | -  - | -  2108 | 113.5  24.2  20.9  9.5 | 28.0  12.4  10.2  5.9 | 47.6  16.8  13.9  6.0 | 4.1  2.0  2.0  1.6 | -  2087 | -  2073 | RNA | RNA | RNA | RNA | RNA | RNA |
| Relative decrease^*^ Sc5 vs *basecase* | 1%  58%  63%  70% | 0%  28%  45%  59% | 1%  41%  52%  63% | na | na | na | 0%  68%  71%  87% | 0%  53%  62%  78% | 0%  56%  60%  79% | na | na | na | RNA | RNA | RNA | na | RNA | RNA |
| Sc6: S3 + vaccination of young WLHIV + frequent screening of WLHIV | 93.8^^^  32.0  25.1  21.7 | 25.9  17.9  12.8  9.3 | 40.6^^^  19.8  14.3  9.8 | 3.6  1.8  2.0  2.3 | -  - | -  2108 | 113.5  19.1  13.9  8.7 | 28.0  12.4  10.0  5.8 | 47.6  14.9  12.0  6.0 | 4.1  1.5  1.4  1.5 | NA  2083 | -  2069 | RNA | RNA | RNA | RNA | RNA | RNA |
| Relative decrease^*^ Sc6 vs *basecase* | 1%  60%  65%  70% | 0%  28%  46%  59% | 1%  42%  52%  63% | na | na | na | 0%  75%  81%  88% | 0%  53%  63%  79% | 0%  61%  66%  79% | na | na | na | RNA | RNA | RNA | na | RNA | RNA |
| E) Age at vaccination of WLHIV – 15-45 years old (Duration of vaccine protection – lifelong, 90% coverage) | | | | | | | | | | | | | | | | | | |
| Sc4: Sc1+ vaccination of young WLHIV | RNA | RNA | RNA | RNA | RNA | RNA | 113.5  51.7  30.0  16.7 | 28.0  20.5  13.1  5.9 | 47.6  29.0  16.6  6.2 | 4.1  2.5  2.3  2.8 | NA  NA | NA  2075 | 173.6  69.5  30.7  9.3 | 33.9  15.6  6.8  2.7 | 79.2  29.9  11.6  3.2 | 5.1  4.5  4.5  3.4 | -  2108 | 2093  2063 |
| Relative decrease^*^ Sc4 vs *basecase* | RNA | RNA | RNA | na | RNA | RNA | 0%  32%  59%  76% | 0%  22%  52%  78% | 0%  25%  53%  78% | na | na | na | 0%  45%  69%  87% | 0%  47%  74%  87% | 0%  43%  71%  87% | na | na | na |
| F) Age at vaccination of WLHIV – 15+ years old (Duration of vaccine protection – lifelong, 90% coverage) | | | | | | | | | | | | | | | | | | |
| Sc4: Sc1+ vaccination of young WLHIV | RNA | RNA | RNA | RNA | RNA | RNA | 113.5  49.1  29.9  18.2 | 28.0  20.1  13.0  6.1 | 47.6  28.2  16.2  6.4 | 4.1  2.4  2.3  3.0 | -  - | -  2075 | 173.6  67.2  30.3  9.3 | 33.9  15.3  6.7  2.7 | 79.2  28.9  11.5  3.2 | 5.1  4.4  4.5  3.4 | -  2107 | 2093  2063 |
| Relative decrease^*^ Sc4 vs *basecase* | RNA | RNA | RNA | na | RNA | RNA | 0%  35%  59%  74% | 0%  24%  52%  77% | 0%  27%  54%  77% | na | na | na | 0%  46%  70%  87% | 0%  48%  74%  87% | 0%  45%  71%  87% | na | na | na |
| G) Not reaching UNAIDS targets of 90-90-90 and 70% male circumcision by 2030 | | | | | | | | | | | | | | | | | | |
| Base case | 94.7  81.4  77.4  75.4 | 25.9  24.9  24.1  24.0 | 40.9  36.7  34.8  34.1 | 3.7  3.3  3.2  3.1 | -  - | -  - | 113.5  75.8  70.7  69.5 | 28.0  26.4  27.2  27.1 | 47.6  40.1  38.4  35.6 | 4.1  2.9  2.6  2.6 | -  - | -  - | 173.6  128.1  112.0  95.1 | 33.9  30.2  28.9  26.3 | 79.2  56.7  48.6  40.4 | 5.1  4.2  3.9  3.6 | -  - | -  - |
| Sc1: Girls’ vaccination | 94.7  69.1  43.7  15.1 | 25.9  19.9  12.1  4.5 | 40.9  31.0  18.0  5.4 | 3.7  3.5  3.6  3.4 | -  - | -  2075 | 113.5  62.4  40.2  17.3 | 28.0  20.7  13.4  6.0 | 47.6  33.6  21.1  8.4 | 4.1  3.0  3.0  2.9 | -  - | -  2085 | 173.6  96.2  46.9  13.6 | 33.9  19.1  9.0  3.9 | 79.2  39.9  18.1  5.7 | 5.1  5.0  5.2  3.5 | -  - | -  2074 |
| Relative decrease^*^ Sc1 vs *basecase* | 0%  15%  44%  80% | 0%  20%  50%  81% | 0%  16%  48%  84% | na | na | na | 0%  18%  43%  75% | 0%  22%  51%  78% | 0%  16%  45%  77% | na | na | na | 0%  25%  58%  86% | 0%  37%  69%  85% | 0%  30%  63%  86% | na | na | na |
| Sc3: Sc1+ 2 lifetime screens | 94.7  50.7  30.8  12.4 | 25.9  16.5  9.5  3.4 | 40.9  24.6  13.7  4.8 | 3.7  3.1  3.2  3.6 | -  - | -  2069 | 113.5  38.3  29.7  10.5 | 28.0  12.1  9.1  3.3 | 47.6  20.2  15.3  5.1 | 4.1  3.2  3.3  3.2 | -  - | -  2073 | 173.6  44.4  16.6  7.5 | 33.9  10.5  4.1  2.3 | 79.2  19.3  7.0  3.2 | 5.1  4.2  4.0  3.3 | -  2073 | 2076  2055 |
| Relative decrease^*^ Sc3 vs *basecase* | 0%  38%  60%  84% | 0%  34%  61%  86% | 0%  33%  61%  86% | na | na | na | 0%  49%  58%  85% | 0%  54%  66%  88% | 0%  50%  60%  86% | na | na | na | 0%  65%  85%  92% | 0%  65%  86%  91% | 0%  66%  85%  92% | na | na | na |
| Sc4: Sc1+ vaccination of young WLHIV | 94.8  63.2  38.1  15.1 | 25.9  19.8  11.8  4.5 | 40.9  30.1  17.2  5.4 | 3.7  3.2  3.2  3.4 | -  - | -  2073 | 113.5  51.2  33.2  17.4 | 28.0  20.8  13.0  6.1 | 47.6  29.7  18.4  8.5 | 4.1  2.5  2.6  2.9 | -  - | -  - | 173.6  81.8  36.6  13.6 | 33.9  17.3  8.0  3.9 | 79.2  34.4  15.1  5.7 | 5.1  4.7  4.6  3.5 | -  2073 | -  2070 |
| Relative decrease^*^ Sc4 vs *basecase* | 0%  22%  51%  80% | 0%  21%  51%  81% | 0%  18%  50%  84% | na | na | na | 0%  32%  53%  75% | 0%  21%  52%  77% | 0%  26%  52%  76% |  | na | na | 0%  36%  67%  86% | 0%  43%  72%  85% | 0%  39%  69%  86% | na | na | na |
| Sc5: Sc3 + frequent screening of WLHIV | 93.9^^^  31.9  19.5  8.9 | 25.9  16.3  9.3  3.4 | 40.7^^^  19.9  11.3  3.9 | 3.6  2.0  2.1  2.6 | -  2096 | 2115  2065 | 113.5  23.5  20.3  5.2 | 28.0  12.0  8.8  3.2 | 47.6  16.3  12.9  4.0 | 4.1  2.0  2.3  1.6 | -  2080 | 2112  2070 | 173.6  28.0  10.4  5.7 | 33.9  9.9  3.8  2.2 | 79.2  14.6  5.2  2.8 | 5.1  2.8  2.7  2.6 | -  2061 | 2067  2051 |
| Relative decrease^*^ Sc5 vs *basecase* | 1%  61%  75%  88% | 0%  35%  61%  86% | 1%  46%  67%  89% | na | na | na | 0%  69%  71%  93% | 0%  55%  68%  88% | 0%  59%  66%  89% | na | na | na | 0%  78%  91%  94% | 0%  67%  87%  92% | 0%  74%  89%  93% | na | na | na |
| Sc6: S3 + vaccination of young WLHIV + frequent screening of WLHIV | 93.9^^^  29.0  18.0  8.9 | 25.9  16.0  9.1  3.4 | 40.7^^^  18.9  10.9  3.9 | 3.6  1.8  2.0  2.6 | -  2089 | 2115  2064 | 113.5  18.9  14.5  5.3 | 28.0  11.9  8.9  3.6 | 47.6  14.5  11.1  4.3 | 4.1  1.6  1.6  1.5 | -  2077 | -  2065 | 173.6  23.1  8.6  5.7 | 33.9  8.7  3.4  2.2 | 79.2  12.1  4.5  2.8 | 5.1  2.7  2.5  2.6 | -  2057 | 2064  2048 |
| Relative decrease^*^ Sc6 vs *basecase* | 1%  64%  77%  88% | 0%  36%  62%  86% | 1%  48%  69%  89% | na | na | na | 0%  75%  79%  92% | 0%  55%  67%  87% | 0%  64%  71%  88% | na | na | na | 0%  82%  92%  94% | 0%  71%  88%  92% | 0%  79%  91%  93% | na | na | na |

IRR: Incidence rate ration between HIV+ and HIV-; - : Elimination not achieved; na: not applicable; RNA: Results for this scenario not available from this model; ^*^: Relative decrease is based on the ratio of the median age-standardised incidence rate of relevant scenarios; ^”^ 2019 estimates are for the very end of 2019/start of 2020 - just before the scale up of intervention starts at the beginning of 2020 – all other estimates are for the end of the year; ^^^ The 2019 values are slightly different than for the other scenarios because screening uptake is assumed to increase linearly between 2017 and 2023 to meet the desired targets in 2023 among WLHIV. This does not occur in the other scenarios because (e.g. 2 lifetime screening of all women) because uptake of screening is already higher in the *basecase* scenario in 2023 than the desired target for 2023; in this instance screening uptake rate are maintained at their *basecase* values until the date of the next higher screening target is reached.
