# Supplementary material for: Estimating the effect of HIV on cervical cancer elimination in South Africa: Comparative modelling of the impact of vaccination and screening
Source: eClinicalMedicine. 2022 Nov 17;54:101754. doi: 10.1016/j.eclinm.2022.101754 (PMC9793279; doi:10.1016/j.eclinm.2022.101754)
Supplement: Technical Appendix [file mmc6.docx]

**­­­Technical appendix**

**Cervical cancer elimination in South Africa: Comparative modelling of the impact of vaccination and screening**

Prof Marie-Claude Boily, PhD^1†^, Prof Ruanne Barnabas MD PhD^2†^, Minttu M Rönn PhD^3¶^, Cara J Bayer^2¶^, Cari van Schalkwyk PhD^4¶^, Nirali Soni^1^, Darcy W Rao^5^, Lisa Staadegaard^1^, Gui Liu^3^, Romain Silhol PhD^1^, Prof Marc Brisson PhD^6,7^, Leigh F Johnson PhD^8^, Paul Bloem MBA^9^, Sami Gottlieb MD^10^, Nathalie Broutet MD, PhD^10^, Shona Dalal PhD^11^

† Joint first authors

^¶^ Contributed equally

TABLE OF CONTENT

**1) Modelled scenarios** ................................................................................................................... Page 1

**2) Overview of each model** .................... .................................................................................... Page 3

Summary of model structure .................... ............................................... ..................................... Page 3

Summary of key differences between models ............................................... ................................Page 6

**3) Calibration – and key setting differences** .................................................................... ..........Page 8

**4) Model outcomes and standardisation**......................................................................................Page 12

**5) Selected model outcomes – post 2020**......................................................................................Page 12

**6) References**…................................................ .............................................................................Page 15

**1) Modelled scenarios**

Table T1: Description of the main vaccination and screening and treat scenarios modelled

| **Scenarios** | **Vaccination**^**^ | | | | | | | **Screening** | | | | | | **Number of models** |
| --- | --- | --- | --- | --- | --- | --- | --- | --- | --- | --- | --- | --- | --- | --- |
|  | **Routine**  **(All)** | | | | **Catch-up**  **(HIV+)** | | | **HIV negative** | | | **HIV-positive** | | |  |
|  | **Vaccine** | **Sex** | **Age (yrs)** | **Cov** | **Sex** | **Age**  **(yrs)** | **Cov** | **Nb of screens** | **Age**  **(yrs)** | **Screening uptake**  **(2023/2030/2045)** | **Nb of screens** | **Age**  **(yrs)** | **Screening uptake***  **(2023/2030/2045)** |  |
| *Basecase* | - | - | - | 0% | - | - | 0% | Baseline^1^ | Baseline^1^ | Baseline^1^ | Baseline^1^ | Baseline^1^ | Baseline^1^ | 3 |
| 1. ***Phase I: 3 main* vaccination and screening scenarios** | | | | | | | | | | | | | |  |
| Sc1 - Vaccination only | HPV9 | F | 9-14 | 80% | - | - | - | Baseline | Baseline | Baseline | Baseline | Baseline | Baseline | 3 |
|  | HPV9 | F | 9-14 | 90% | - | - | - | Baseline | Baseline | Baseline | Baseline | Baseline | Baseline | 3 |
| Sc2 - Vaccination + 1 lifetime screen | HPV9 | F | 9-14 | 80% | - | - | - | 1 | 35 | 45, 70, 90% | 1 | 35 | 45, 70, 90% | 3 |
|  | HPV9 | F | 9-14 | 90% | - | - | - | 1 | 35 | 45, 70, 90% | 1 | 35 | 45, 70, 90% | 3 |
| Sc3 - Vaccination + 2 lifetime screens | HPV9 | F | 9-14 | 80% | - | - | - | 2 | 35, 45 | 45, 70, 90% | 2 | 35, 45 | 45, 70, 90% | 3 |
|  | HPV9 | F | 9-14 | 90% | - | - | - | 2 | 35, 45 | 45, 70, 90% | 2 | 35, 45 | 45, 70, 90% | 3 |
| 1. ***Phase II: Adding vaccination and screening focused on WLHIV*** | | | | | | | | | | | | | | |
| Sc1+ vaccinating young WLHIV | HPV9 | F | 9-14 | 80% | F | 15-24 | 50% | Baseline | Baseline | Baseline | Baseline | Baseline | Baseline | 3 |
|  | HPV9 | F | 9-14 | 80% | F | 15-24 | 80% | Baseline | Baseline | Baseline | Baseline | Baseline | Baseline | 3 |
|  | HPV9 | F | 9-14 | 90% | F | 15-24 | 50% | Baseline | Baseline | Baseline | Baseline | Baseline | Baseline | 3 |
|  | HPV9 | F | 9-14 | 90% | F | 15-24 | 90% | Baseline | Baseline | Baseline | Baseline | Baseline | Baseline | 3 |
| Sc3 + frequently screening WLHIV | HPV9 | F | 9-14 | 90% | F | - | - | 2 | 35, 45 | 45, 70, 90% | Every 3years | 25-49 | 45, 70, 90% | 3 |
| Sc3 + vaccinating young WLHIV + frequently screening WLHIV | HPV9 | F | 9-14 | 90% | F | 15-24 | 50% | 2 | 35, 45 | 45, 70, 90% | Every 3years | 25-49 | 45, 70, 90% | 3 |
|  | HPV9 | F | 9-14 | 90% | F | 15-24 | 90% | 2 | 35, 45 | 45, 70, 90% | Every 3years | 25-49 | 45, 70, 90% | 3 |

F: female; Nb: Nombre; Cov: Coverage defined as proportion in the given age cohorts vaccinated; HPV9: nonavalent vaccine against 7 high oncogenic risk HPV (HR-HPV16/18/31/33/45/52/58); Uptake: Proportion of women of the eligible age cohorts screened;

^1^Baseline screening can differ across models – see details in Table T3;

*If screening level has already reached or exceeded the 2023 target before 2023, we assumed that it stayed constant until the date of the next target;

** In all scenarios, including *basecase*, vaccination starts in 2020. We did not account for vaccination of 9 year-olds with the bivalent vaccine that has been implemented in South Africa in 2014 (with estimated coverage 1-2 doses of 60-80%)(1) to ensure our results are comparable with the previous model comparison analysis of the Cervical Cancer Elimination Initiative (CCEMC) for 78 LMICs(2), which also assumes no vaccination in *basecase*. This is not expected to influence estimates of cervical cancer incidence and the likelihood of elimination since this corresponds to a small fraction of the 9-74 year-olds in 2020 and the same or a larger fraction of 14 year-olds are vaccinated in our different scenarios in 2020. The impact of vaccination with a bivalent vaccine is assessed in sensitivity analysis (Table T2).

Additional assumptions^^^ – unless specified differently in sensitivity analysis

| Additional assumptions on vaccine efficacy (VE):   - 100% VE - Lifelong protection - Same for HIV positive and negative | Additional assumptions for cervical cancer screening and treatment:   - Sensitivity of screening: 90% for CIN2, 94% for CIN3, 94% for cervical cancer - 90% of women diagnosed with CIN2+ treated - No triage - 100% Treatment efficacy - 10% loss to follow-up | Additional assumptions on HIV interventions:   - UNAIDS 90-90-90 HIV treatment targets achieved by 2030 - Male circumcision coverage reaches 70% by 2030 |
| --- | --- | --- |
| ^^^As in main CCEMC analysis(2) | | |

Table T2: Scenarios modelled in the sensitivity analysis

| **Scenarios** | **Vaccination**^**^ | | | | | | | **Screening** | | | | | | | | **Number of models** |
| --- | --- | --- | --- | --- | --- | --- | --- | --- | --- | --- | --- | --- | --- | --- | --- | --- |
|  | **Routine**  **(All)** | | | | **Catch-up**  **(HIV+)** | | | **HIV negative** | | | | | **HIV-positive** | | |  |
|  | **Vaccine** | **Sex** | **Age (yrs)** | **Cov** | **Sex** | **Age**  **(yrs)** | **Cov** | **Nb of screens** | **Age**  **(yrs)** | | | **Screening uptake**  **(2023/2030/2045)** | **Nb of screens** | **Age**  **(yrs)** | **Screening uptake***  **(2023/2030/2045)** |  |
| ***Phase III): 3 main* vaccination and screening scenarios** | | | | | | | | | | | | | | | | |
| *A)* Assuming the % on ART/virally suppressed and % VMMC and % condom use plateaus at 2020 levels (instead of reaching UNAIDS 90-90-90 and 70% male circumcision targets by 2030) | | | | | | | | | | | | | | | | |
| *Base case* – under low ART | - | - | - | 0% | - | - | - | Baseline^1^ | Baseline^1^ | | Baseline^1^ | | Baseline^1^ | Baseline^1^ | Baseline^1^ | 3 |
| Vaccination only | HPV9 | F | 9-14 | 90% | - | - | - | Baseline | Baseline | | Baseline | | Baseline | Baseline | Baseline | 3 |
| Vaccination + 2 lifetime screens | HPV9 | F | 9-14 | 90% | - | - | - | 2 | 35, 45 | | 45, 70, 90% | | 2 | 35, 45 | 45, 70, 90% | 3 |
| *B) Assumes nonavalent vaccine protection lasts 20 years only (instead of lifelong)* | | | | | | | | | | | | | | | | |
| Vaccination only - 20 years duration | HPV9 | F | 9-14 | 90% | - | - | - | Baseline | Baseline | Baseline | | | Baseline | Baseline | Baseline | 3 |
| Vaccination – 20 years + 2 lifetime screens | HPV9 | F | 9-14 | 90% | - | - | - | 2 | 35, 45 | 45, 70, 90% | | | 2 | 35, 45 | 45, 70, 90% | 3 |
| ***C)*** *With the bivalent vaccine*** (instead of nonavalent)* | | | | | | | | | | | | | | | | |
| Vaccination only | HPV9 | F | 9-14 | 90% | - | - | - | Baseline | Baseline | Baseline | | | Baseline | Baseline | Baseline | 2 |
| Vaccination + 2 lifetime screens | HPV9 | F | 9-14 | 90% | - | - | - | 2 | 35, 45 | 45, 70, 90% | | | 2 | 35, 45 | 45, 70, 90% | 2 |
| ***Phase IV):***  ***Adding vaccination and screening focused on WLHIV*** | | | | | | | | | | | | | | | | |
| *A)* Assuming the % on ART/virally suppressed and % VMMC and % condom use plateaus at 2020 levels (instead of reaching UNAIDS 90-90-90 and 70% male circumcision targets by 2030) | | | | | | | | | | | | | | | |  |
| Sc1+ vaccinating young WLHIV | HPV9 | F | 9-14 | 90% | F | 15-24 | 90% | Baseline | Baseline | Baseline | | | Baseline | Baseline | Baseline | 3 |
| Sc3 + frequently screening WLHIV | HPV9 | F | 9-14 | 90% | F | - | - | 2 | 35, 45 | 45, 70, 90% | | | Every 3years | 25-49 | 45, 70, 90% | 3 |
| Sc3 + vaccinating young WLHIV + frequently screening WLHIV | HPV9 | F | 9-14 | 90% | F | 15-24 | 90% | 2 | 35, 45 | 45, 70, 90% | | | Every 3years | 25-49 | 45, 70, 90% | 3 |
| *B) Assumes nonavalent vaccine protection lasts 20 years only (instead of lifelong)* | | | | | | | | | | | | | | | | |
| Sc1+ vaccinating young WLHIV – 20 years duration | HPV9 | F | 9-14 | 90% | F | 15-24 | 90% | Baseline | Baseline | Baseline | | | Baseline | Baseline | Baseline | 3 |
| Sc3 – 20 years + frequently screening WLHIV | HPV9 | F | 9-14 | 90% | F | - | - | 2 | 35, 45 | 45, 70, 90% | | | Every 3years | 25-49 | 45, 70, 90% | 3 |
| Sc3 + vaccinating young WLHIV – 20 years + frequently screening WLHIV | HPV9 | F | 9-14 | 90% | F | 15-24 | 90% | 2 | 35, 45 | 45, 70, 90% | | | Every 3years | 25-49 | 45, 70, 90% | 3 |
| ***C)*** *With the bivalent vaccine (instead of nonavalent)* | | | | | | | | | | | | | | | | |
| Sc1 + vaccinating young WLHIV | HPV2 | F | 9-14 | 90% | F | 15-24 | 90% | Baseline | Baseline | Baseline | | | Baseline | Baseline | Baseline | 2 |
| Sc3 + frequently screening WLHIV | HPV2 | F | 9-14 | 90% | F | - | - | 2 | 35, 45 | 45, 70, 90% | | | Every 3years | 25-49 | 45, 70, 90% | 2 |
| Sc3 + vaccinating young WLHIV + frequently screening WLHIV | HPV2 | F | 9-14 | 90% | F | 15-24 | 90% | 2 | 35, 45 | 45, 70, 90% | | | Every 3years | 25-49 | 45, 70, 90% | 2 |
| D) Extended age at vaccination for WLHIV | | | | | | | | | | | | | | | | |
| Sc1+ vaccinating WLHIV - 15-45 years old | HPV9 | F | 9-14 | 90% | F | 15-45 | 90% | Baseline | Baseline | Baseline | | | Baseline | Baseline | Baseline | 2 |
| Sc1+ vaccinating WLHIV – All ages | HPV9 | F | 9-14 | 90% | F | All | 90% | Baseline | Baseline | Baseline | | | Baseline | Baseline | Baseline | 2 |

F: female; Nb: number; Cov: Coverage defined as proportion of the given age cohorts vaccinated; HPV9: nonavalent vaccine against 7 high oncogenic risk HPV (HR-HPV16/18/31/33/45/52/58), HPV2: a vaccine that only protects against HR-HPV16/18, without cross protection as in the CCEMC analysis(2); Uptake: Proportion of women of the eligible age screened annually;

^1^Baseline screening can differ across models – see details in Table T3;

*If screening level has already reached or exceeded the 2023 target before 2023, we assumed that it stayed constant until the date for the next target;

** In all scenarios, including *basecase*, vaccination starts in 2020. We ignored vaccination of 9 year-olds with the bivalent that has been implemented in South Africa in 2014 (with estimated coverage 1-2 doses of 60-80%)(1) to ensure our results are comparable with the previous model comparison analysis of the Cervical Cancer Elimination Initiative (CCEMC) for 78 LMICs(2). This is not expected to influence estimates of cervical cancer incidence and the likelihood of elimination since this corresponds to a small fraction of the 9-74 year-olds in 2020 and the same or a larger fraction of 14 year-olds are vaccinated in our different scenarios in 2020

**2) Overview of each model**

Three transmission dynamic models *Det_HIV-HIV* (developed by Harvard Chan School of Public Health and Imperial College London team), *MicroCOSM-HPV* (developed by SACEMA and CIDER (Centre for Infectious Disease Epidemiology and Research, University of Cape Town, Cape Town, South Africa) team), and *DRIVE* (developed by the University of Washington) calibrated to the South African context were identified, adapted, and used to assess the population-level impact of HPV vaccination and cervical cancer (CC) screening, in a country with a high burden of HIV and high HIV interventions scale-up, which are known to modify HPV disease progression and cervical cancer risks. The models were designed to explicitly represent the transmission dynamics of co-circulating HIV and HPV infections and disease progression, and the scale-up of relevant HIV and HPV/CC interventions at the national level (for 2 models: *Det_HIV-HIV* and *MicroCOSM-HPV*) and in KwaZulu-Natal province (for 1 model: *DRIVE*). Each model is structured to reflect five main domains: 1) demography, 2) sexual behaviour and transmission, 3) natural history of infections and disease progression, 4) multiple HIV and HPV interactions, and 5) interventions. Each calibrated model (i.e., the *basecase* scenario) is used to simulate different vaccination and screening and treatment scenarios described in Tables T1-T2. The summary of each model is as follows.

*Summary of model structure*

| *Box 1: Summary of each model* |
| --- |
| *i) Det_HPV-HIV (Harvard Chan School of Public Health/Imperial College London) – South Africa* |
| *Det_HPV-HIV* is a model for South Africa at the national level*.*  Briefly, *Det_HPV-HIV* is a deterministic transmission dynamic model that simulates HIV and 3 groups of high-risk HPV genotypes (HPV16/18, HPV 31/33/45/52/58, and non-vaccine high-risk HPV types), HIV disease progression, and HPV-induced cervical carcinogenesis associated with each HR-HPV group type in a heterosexual population stratified by sex, 5-years age groups, and 3 sexual activity levels (with different rate of partner acquisition). Each HPV related health state represents the underlying true health state of the simulated individuals (as opposed to a diagnosed state) such as infection and natural immunity status, high-grade cervical intraepithelial neoplasia (CIN2+), and cervical cancer (see model flowchart below). The model assumes that HIV increases the risk of HPV acquisition and progression to CIN2+, reduces clearance of infection, regression from CIN2+, and duration of immunity. It also assumed that HPV infection increases the risk of HIV acquisition. Women living with HIV (WLHIV) who receive HIV treatment (i.e. on ART) have a partially reduced risk of HPV and associated disease progression compared to WLHIV not on ART, but they have a higher risk compared to HIV negative individuals. HIV treatment (ART) reduces HPV disease progression (compared to people living with HIV [PLHIV] not on ART), but male circumcision and condom use are not protective against HPV acquisition in the model (Table T3).  The model represents evolution in the levels of HIV interventions such as condom use, male circumcision and HIV treatment (ART) of PLHIV by sex, age, sexual activity level over time since the beginning of the HIV epidemic in 1985. The model represents a baseline level of cervical cancer screening and treatment (starting in 2012).  The per capita risk of infection among individuals susceptible to the infection (HIV, or one of the three grouped HPV types) depends on the individuals’ sexual activity, number and types of sex acts, sexual mixing patterns, the prevalence of infection among partners, HIV/ART or HPV status, cofactors effect between HIV and HPV infections, and levels and efficacy of interventions. The transition rates between health states depend on sex, age, HPV group type, and HIV/ART status.  Additional details of the *Det_HPV-HIV* model and parameter used can be found in model-specific technical appendix T1. |
| *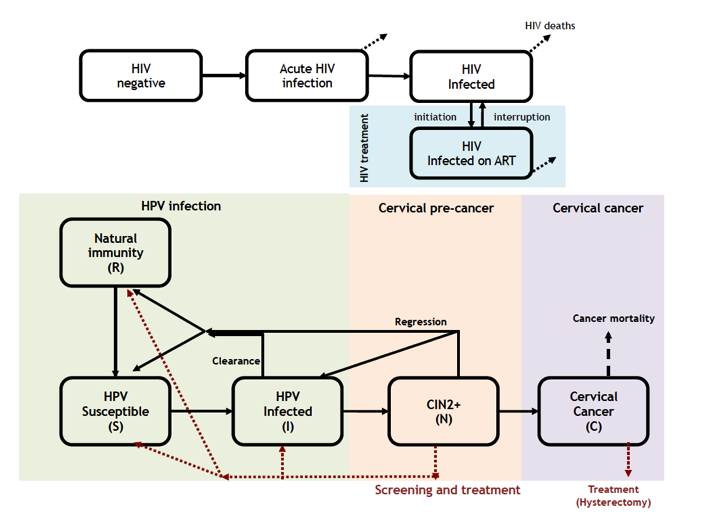*  *Flowchart of the model representing the HIV and HPV/CC health states represented in the model. Arrows represent flows between health states* |
| *ii) MicroCOSM-HPV (SACEMA) – South Africa* |
| *MicroCOSM-HPV* is also a model for South Africa at the national level. Briefly, *MicroCOSM-HPV* is an individual-based stochastic model representing 1 million individuals in 1985. The model simulates HIV and HPV transmission (13 independent high-risk types: vaccine types 16, 18, 31, 33, 45, 52, 58, and non-vaccine types: 35, 39, 51, 56, 59, 68) and HPV-induced cervical carcinogenesis associated with each type among heterosexual individuals by sex, single age cohorts, and sexual risk. Each health state represents the underlying true health state of each simulated individual (as opposed to a diagnosed state) such as active infection, latent infection and natural immunity status, cervical intraepithelial neoplasia (CIN1, 2, 3), and 8 cancer stages (4 stages undiagnosed and 4 diagnosed) (see model flowchart below). The model assumes that HIV increases the risk of persistent HPV infections and disease progression to cervical cancer among WLHIV, whereas ART reduces the risk of HPV and disease progression compared to WLHIV not on ART but not completely compared to HIV negative women. Condom use and ART are also effective against HPV infections and/or disease progression. Male circumcision is assumed to have no effect on HPV acquisition for men or transmission from men to women. HIV infection does not influence the duration of natural immunity, but reactivation of a latent infection depends on the stage of HIV infection, and ART status. No effect of HPV on HIV is assumed (Table T3).  The model accounts for changes in the levels of HIV interventions such as condom use and HIV treatment over time since the beginning of the HIV epidemic in 1990. The model also represents baseline cervical cancer screening and treatment through a detailed screening and diagnosis algorithm (starting in 2000).  Model transitions between health states are governed by probabilities that depend on sex, age, HPV type, and HIV/ART status. The probabilities of HPV or HIV transmission among susceptible individuals depend on their sexual activity, the prevalence of infection among partners, and level of interventions (condom use, ART status). The per sex-act transmission probability for HPV is independent of HIV status (of the individual or their partner), and vice versa.  Additional details of the *MicroCOSM-HPV* model and parameters used can be found in model-specific technical appendix T2. |
| *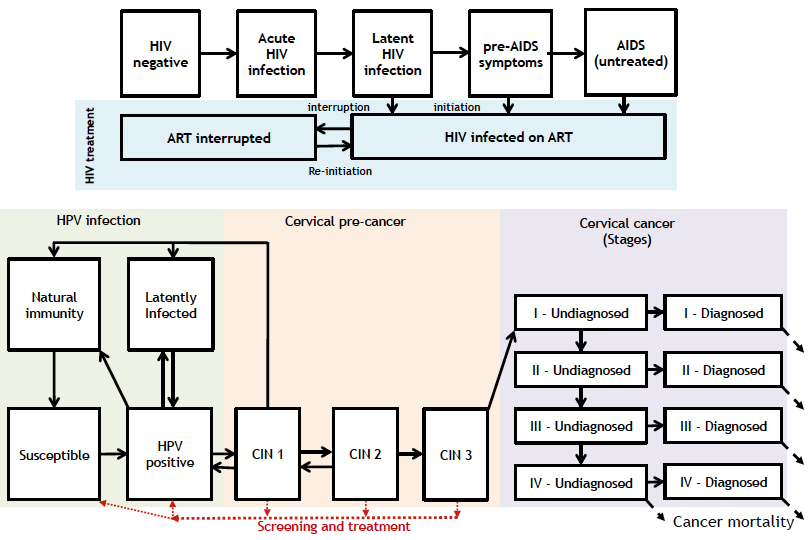*  *Flowchart of the model representing the HIV and HPV/CC health states represented in the model. Arrows represent flows between health states* |
| iii) *DRIVE (Data-driven Recommendations for Interventions against Viral infEction) (University of Washington) – KwaZulu-Natal* |
| *DRIVE* is a model for the province of KwaZuluNatal. Briefly, DRIVE is a deterministic transmission dynamic model that simulates HIV and HPV transmission (2 groups of high-risk genotypes: HPV16/18/31/33/45/52/58, non-vaccine high-risk HPV types), HIV disease progression and HPV-induced cervical carcinogenesis associated with each type group in a heterosexual population that is stratified by sex, 5-year age groups, and sexual activity. Each health state aims to represent the underlying true health state of the simulated individuals (as opposed to a diagnosed state) such as infection status, grade of cervical intraepithelial neoplasia (CIN1, 2, 3), and cancer stage (see flowchart below). The model takes into account the multiple interactions between HIV and HPV infection and disease progression, namely it is assumed that HIV increases the risk of HPV acquisition and disease progression to cervical cancer. WLHIV on ART and virally suppressed are assumed to experience rates of HPV acquisition and disease progression comparable to HIV negative women, but their HPV clearance rate, disease regression rates, and cervical cancer-associated mortality are only partially restored (and are assumed similar to untreated WLHIV with high (>500) CD4 count). The model also assumes that condom use is effective against HPV acquisition. Male circumcision is assumed to have no effect on HPV (details in table T3 below).  The model represents variation in the levels of HIV interventions such as condom use, male circumcision and HIV treatment (ART) of people living with HIV (PLHIV) over time since the beginning of the HIV epidemic in 1980. The model assumes that all individuals treated with ART are virally suppressed. The model also represents HPV vaccination (starting in 2020) and baseline cervical cancer screening and treatment (starting in 2000).  The risk of HPV acquisition among susceptible individuals depends on their sexual activity, the prevalence of infection among partners, HIV and ART status, and level of condom use and HPV vaccination interventions. The transition rates between HPV health states depend on sex, age, HPV type group, and HIV/ART status. The risk of HIV acquisition depends on sexual activity, the prevalence of infection and viral load among partners, and level of HIV interventions. The transition rates between HIV health states depend on sex and age.  Additional details of the *DRIVE* model and parameter used can be found in model-specific technical appendix T3.  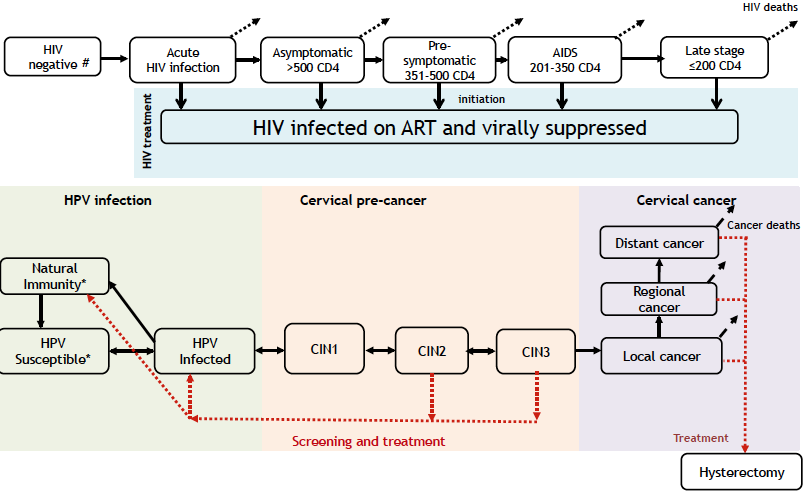  *Flowchart of the model representing the HIV and HPV/CC health states represented in the model. Arrows represent flows between health states. ^*^HPV vaccination is effective only for individuals in these stages. Once received, vaccination status is tracked through subsequent transitions. ^#^HIV susceptible men are stratified by circumcision status, with circumcision decreasing the risk of HIV acquisition.* |

*Summary of key assumptions between models*

Key differences between models included the mathematical formalism (deterministic versus individual based), number of age groups, number of high-risk (HR)-HPV group types modelled, baseline HIV prevalence, baseline cervical cancer incidence and HIV intervention levels (particularly between the South Africa and KwaZulu-Natal models), baseline cervical cancer screening, specific quantitative assumptions on the magnitude of the association between HIV at different stages and HPV disease progression, number of stages between HPV infections and cervical cancer incidence, and duration of natural immunity (Table T3). Additional details on each model can be found in model-specific technical appendix T1-T3.

Table T3: Summary of key assumptions across models (Additional details are provided for each model in model-specific technical appendix T1-T3).

|  | ***Det_HPV-HIV*** | ***MicroCOSM-HPV*** | ***DRIVE*** | |
| --- | --- | --- | --- | --- |
| **Setting** | - South Africa | - South Africa | | - KwaZulu-Natal, South Africa |
| **Age groups** | - 9-74 (13 x 5-year age groups) | - 0-99+ (single year) | | - 0-79 (16 x 5-year age groups) |
| **HPV types** | - 3 groups HR types: - HPV16/18 - HPV31/33/45/52/58 - Other HR HPV | - Multiple single HR types: - HPV16/18/31/33/45/52/58 individually & other HR HPV | | - 2 groups HR types: - HPV16/18/31/33/45/52/58 - Other HR HPV |
| **Natural immunity following HPV infection in women** | - 45-75% develop it (100% protection) - Duration: ~5 years to lifelong | - 30-60% develop it (100% protection) - Duration: 16-19 years | | - 100% develop it (0-50% protection) - Duration: 41 years |
| **HIV/ HPV interactions** | - HIV increases susceptibility to HPV infection - HIV reduces clearance of HPV - HIV increases disease progression (to CIN2+) - HIV reduces regression from CIN2+ - HIV increases waning of HPV natural immunity (1.5-2.0 x shorter in PLHIV) - Magnitude of interactions are independent of HIV disease stage - HPV increases HIV susceptibility | - HIV increases the risk of persistent HPV infection - HIV increases disease progression to CC - HIV increases reactivation of latent infections – varies by stage of HIV infection - HIV does not influence natural immunity against HPV - Magnitude of interactions are dependent of HIV disease stage - HPV does not influence HIV susceptibility | | - HIV increases susceptibility to HPV infection - HIV reduces clearance of HPV - HIV increases disease progression to CC - HIV reduces disease regression from CIN2 or CIN3 - Magnitude of interactions are dependent of CD4 levels - HIV increases waning of HPV natural immunity (1.4-2.8 x shorter in PLHIV - HIV increases cervical cancer-associated mortality - HPV does not influence HIV susceptibility |
| **Effect of ART on HPV/CC** | - ART partially reduces HPV infection and disease progression risk in WLHIV compared to WLHIV not on ART but not completely compared to HIV negative | - ART partially reduces HPV infection and disease risk in WLHIV compared to WLHIV not on ART but not completely compared to HIV negatives - ART reduces reactivation of latent infections compared to WLHIV not on ART | | - ART reduces HPV infection risk of virally suppressed WLHIV to the same risk level as HIV negatives - ART partially reduces HPV clearance, natural immunity waning, disease progression rates, disease regression rates, and cervical cancer-associated mortality of virally suppressed WLHIV to the same risk level asuntreated WLHIV with >500 CD4 count |
| **Effects of HIV interventions on HPV infection** | - Condom use does not reduce HPV acquisition risk for women or men - Male circumcision does not reduce acquisition risk for men | - Condom use partially reduces HPV acquisition risk - Male circumcision does not affect HPV acquisition risk | | - Condom use partially reduces HPV acquisition risk for women and men - Male circumcision does not affect HPV acquisition risk |
| **Intervention levels in *basecase* scenario** | - ART coverage scaled up to reach 90-90-90 in 2030 - Male circumcision coverage reaches 70% in 2030 - No vaccination - Baseline CC screening* | - ART scaled up to reach 90-90-90 in 2030. - Male circumcision coverage reaches 70% in 2030. - No vaccination - Baseline CC screening* | | - ART scaled up to reach 90-90-90 in 2030 - Male circumcision coverage reaches 70% in 2030 No vaccination - Baseline CC screening* |
| **Baseline vaccination** | - None (see section 3) | - None (see section 3) | | - None (see section 3) |
| **Baseline CC screening (*basecase*)** | - Screen for CIN2+ - 20%, 25%, 25% of 25, 35 and 45 years old screened respectively in 2012 linearly increasing to 40%, 55%, 55% by 2017^1, &^ - Same for HIV+/and HIV- - Additional details provided in section 4 of model-specific technical appendix T1 | - Screen for CIN1+^2^ - Annual probability of entering screening programme depend on age, ART status and time; informed by Western Cape individual-level screening data^4,&^. - See section 4 of model-specific technical appendix T2 for details on various parameters used | | - Screen for CIN2+ - Screen additional women each year to have screened 0%, 18% & 48% of 35-39 year-old women once in a lifetime by 2000, 2003 and 2016 (linearly increasing)^3^. - Same for HIV+ and HIV- - Additional details provided in section 4 of model-specific technical appendix T3 |
| **Proportion of women in relevant age groups screened annually** | - See figure T5.3 and T5.4 | - See figure T5.3 and T5.4 | | - See figure T5.3 and T5.4 |
| ***Basecase* treatment efficacy** | - 91% for CIN2+ for HIV- women - 77% for CIN2+ for WLHIV - 58% for CC for HIV- women - 58% for CC for WLHIV | - 75% for CIN1+ - 40% for CIN1+ for WLHIV | | - 91% for CIN2+ - 77% for CIN2+ for WLHIV |
| **Probability of being treated at the end of cascade for women who are screened^++^** | - HIV- : 16% - HIV+: 13% | - HIV- : 11% - HIV+: 5%   (but women can return following inadequate screen) | | - HIV- : 19% - HIV+: 16% |

***Pre-existing level of screening assumed before 2020 for each model (in *Basecase* scenario)- assumptions across model differ due to different data used, model structure and setting – we did not standardise baseline levels of screening across models as this reflected uncertainty in data and their interpretation.

^&^ Two models (Det_HPV-HIV, MicroCOSM-HPV) explicitly specififed the uptake, defined as the fraction of women of the given age screened each year. ^1^ Based on Health Barometer data (3–5) and secondary analysis of 2012 SABSS data(6)_._

^2^Two consecutive LSIL cytology results are referred to colposcopy; HSIL+ immediately.

^3^One model (DRIVE) screened additional women of the given age group each year to reach a defined coverage level based on 2016 SADHS data(7). Given the model’s 5-year age groups, screening every 5 years is modelled in scenarios with enhanced screening for women with HIV(details in model-specific technical appendix T3)

^4^Western Cape data(8)

**^++^** The probability that a woman who has been screened that year will be adequately treated depends on the test adequacy, the sensitivity of the screening test, the probability to follow-up for colposcopy/biopsy, the probability of a positive diagnosis following colposcopy, the proportion who are treated and the probability that the treatment is successful (additional details on the cascade are presented in model-specific technical appendix T1-T3).

**3) Calibration & key setting differences**

All models were parameterised and fitted to stratified sexual behaviour and epidemiological data over time (e.g., by sex, age, HIV status) specific to South Africa at the national level (*MicroCOSM-HPV,* *Det_HIV-HPV*) and to KwaZulu-Natal province (KZN) (*DRIVE*). All models used a Bayesian multidimensional calibration process to identify posterior sets of demographic, behavioural and biological parameters, from pre-specified prior distributions. These posterior parameter sets generated predictions consistent with several epidemiological outcomes over time and allowed propagating parameter uncertainties in model predictions. Typical outcomes fitted included: HIV prevalence by age and sex, HPV infections and HPV disease stage prevalence by age, sex and HIV status, HPV type distribution, and cervical cancer incidence. For each model, the posterior parameter sets that reproduce available data while accounting for pre-existing levels of interventions were used to simulate the *basecase* scenario. In absence of intervention scale-up, the calibrated *basecase* scenario assumed the HPV and cervcal cancer intervention efforts remained constant at their 2020 pre-existing values until 2120 (whereas HIV intervention increased to meet UNAIDS targets). Scenarios with scale-up of HPV vaccination and cervical screening and treatment interventions are introduced in 2020. The specific demography and epidemiology of HIV and HPV/cervical cancer of each setting (i.e., national or KwaZulu-Natal province) was taken into account as relevant during modeling and calibration.

Given the existence of a national screening program since 2000 in South Africa, the models’ *basecase* scenarios assumed some levels of screening prior to 2020 (e.g , <12% of women age 35-39 are screened each year across models)(5,9) (Figure T5.3-5.4). The South Africa national vaccination program for 9-year-old schoolgirls that started in 2014 (with the bivalent vaccine) achieved ~60% coverage of the second dose(1,10). However, to be consistent with the previous model comparison analysis of the Cervical Cancer Elimination Initiative (CCEMC) for 78 LMICs(2), our *basecase* scenario assumes no vaccination prior to 2020. In addition, since the coverage achieved by the national programme among 9-14 year-olds and among all women by 2020 is below what is achieved in our girls' vaccination scenario (which vaccinates 90% of 9-14 years old girls), this has a negligible impact on our post 2020 model predictions of cervical cancer incidence and time to elimination. In addition, as very few women between ages 9 and 14 are expected to get infected with HPV and/or develop cancer by 2020, this also does not influence calibration.

The data sources used for calibration could vary across models reflecting differences in model structure, settings modelled, and interpretation on the relevance and quality of available data. For example, the *Det_HPV-HIV* model used Globocan national age-specific cervical cancer incidence estimates for 2018 for calibration(11), whereas the *MicroCOSM-HPV* model was fitted to age-specific pathology diagnosed cancer incidence as reported by the National Cancer Registry between 2000 and 2016(12) and cross-validated with Globocan estimates(11). As reliable estimates of cervical cancer incidence were not available for KwaZulu-Natal, the national Globocan 2018 cancer incidence rates by age were adjusted to take into account higher HIV prevalence in KwaZulu-Natal. Assuming that WLHIV have four times increased risk of cervical cancer(13), the overall cervical cancer incidence rate was reweighted according to the HIV prevalence in KwaZulu-Natal compared to South Africa nationally.

Models also used different sources of demographic projections of population growth and/or age distribution for 2020 onward to be replicated by the model. *Det_HPV-HIV* model was based on United Nations population estimates(14). *MicroCOSM-HPV* assumed that rates of fertility and mortality will decline to minimum levels (based on the American Census Bureau’s International Data Base projections)(15) and that net international migration will decline to zero(16). *DRIVE* model assumed a 50% linear decline in fertility rates from 2020 to 2035 to match projected United Nations Population Division estimates for population size, age distribution, and total fertility(14,17). Additional details of each model structure, data used for calibration, model fits, and vaccination coverage are presented in model-specific technical appendix T1-T3.

Table T4 show models fits to key demographic, HIV, HPV, and cervical cancer outcomes. Additional fit results are shown in model-specific technical appendix T1-T3. Briefly, KwaZulu-Natal has a higher HIV prevalence than South Africa overall (27% in KwaZulu-Natal vs. 20% nationally among adults aged 15-49 in 2017)(18), a higher proportion of persons living with HIV with viral suppression (68% in KwaZulu-Natal vs. 62% nationally among all PLHIV in 2017)(18), and similar male medical circumcision coverage (31% in KwaZulu-Natal vs. 32% nationally among men aged 15 and older in 2017)(18).

| Table T4: Summary of key calibration & cross validation results in *basecase* scenario for each model | | | |
| --- | --- | --- | --- |
|  | **Det_HPV-HIV model** | **MicroCOSM-HPV model** | **DRIVE model**** |
| **A) Age distribution in 2019** | 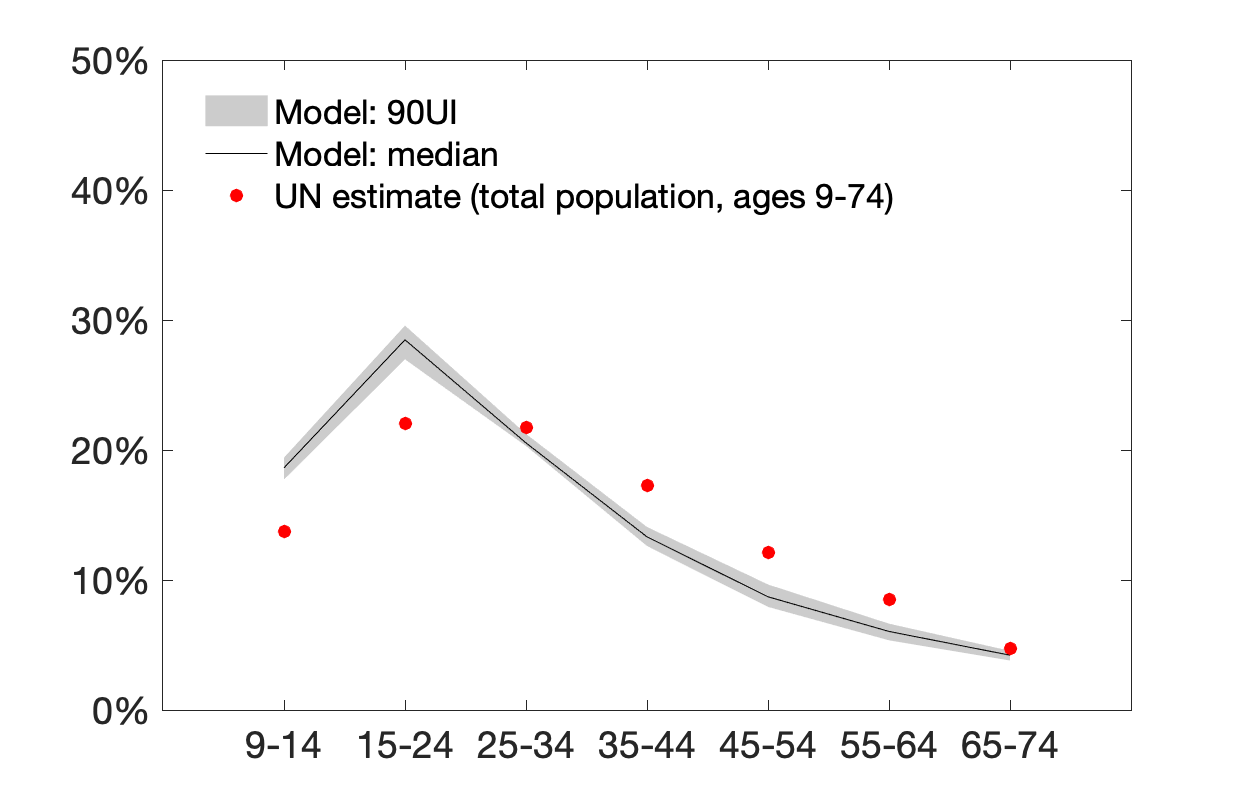 | 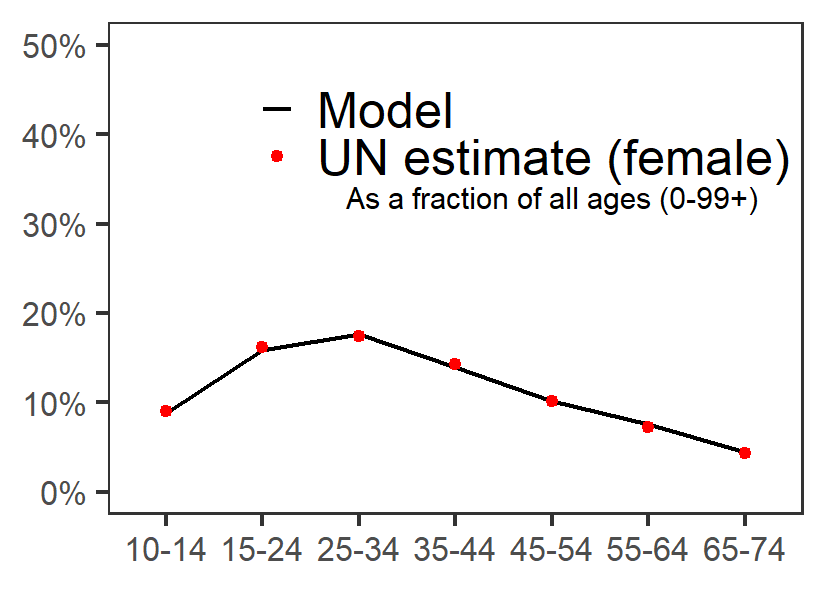 | **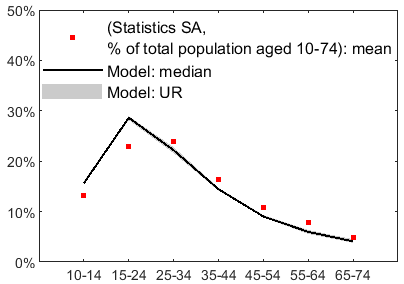** |
| **B) HIV prevalence over time in women (15-49 years old)** | **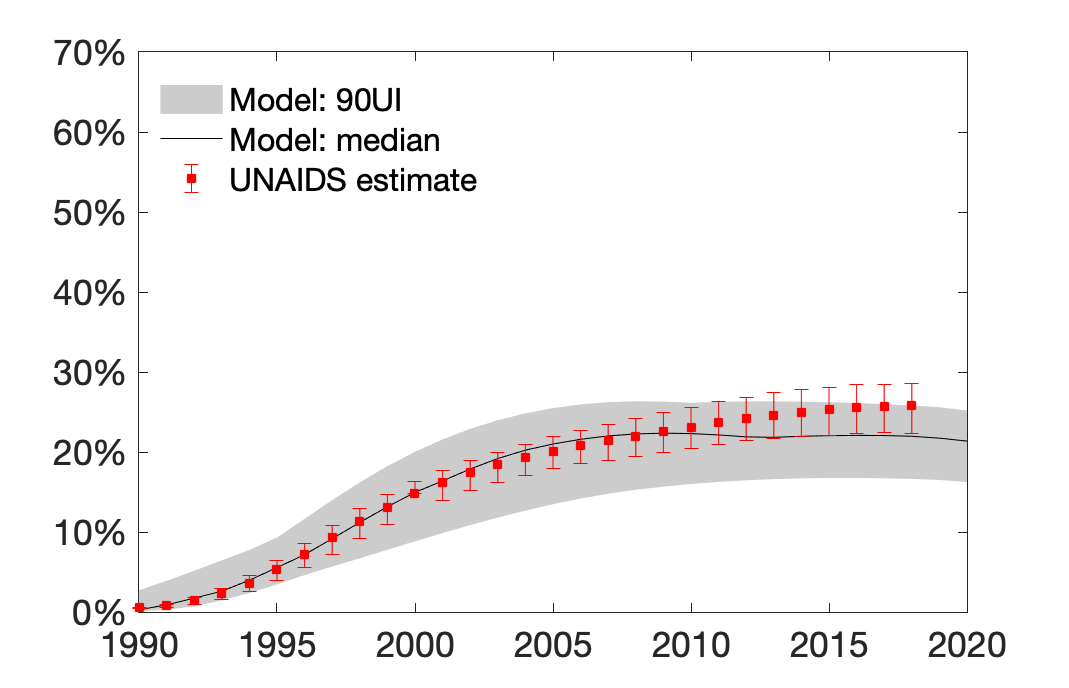** | 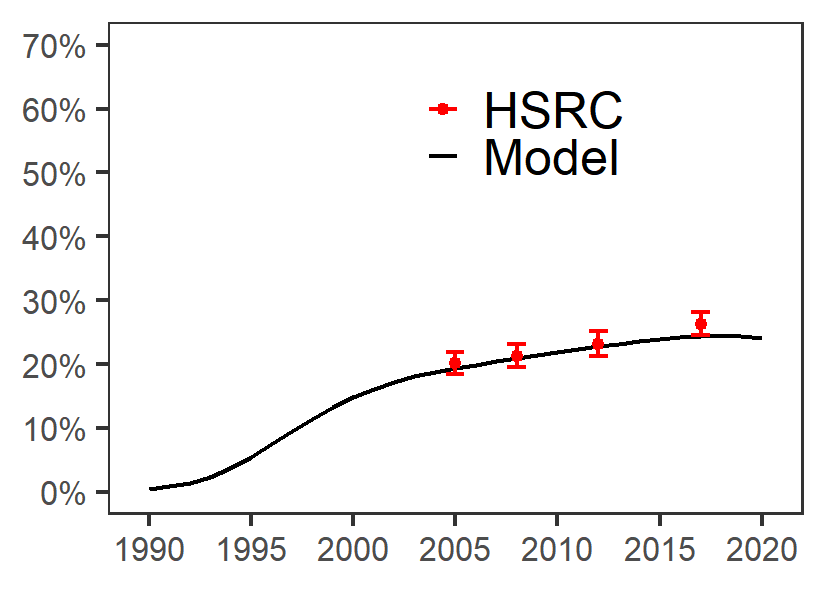 | **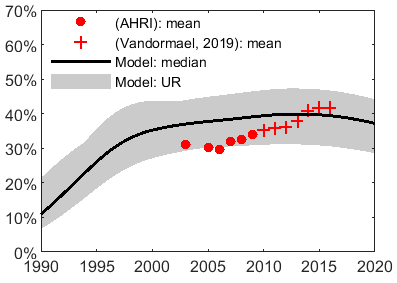** |
| **C) HIV prevalence by age in women** | **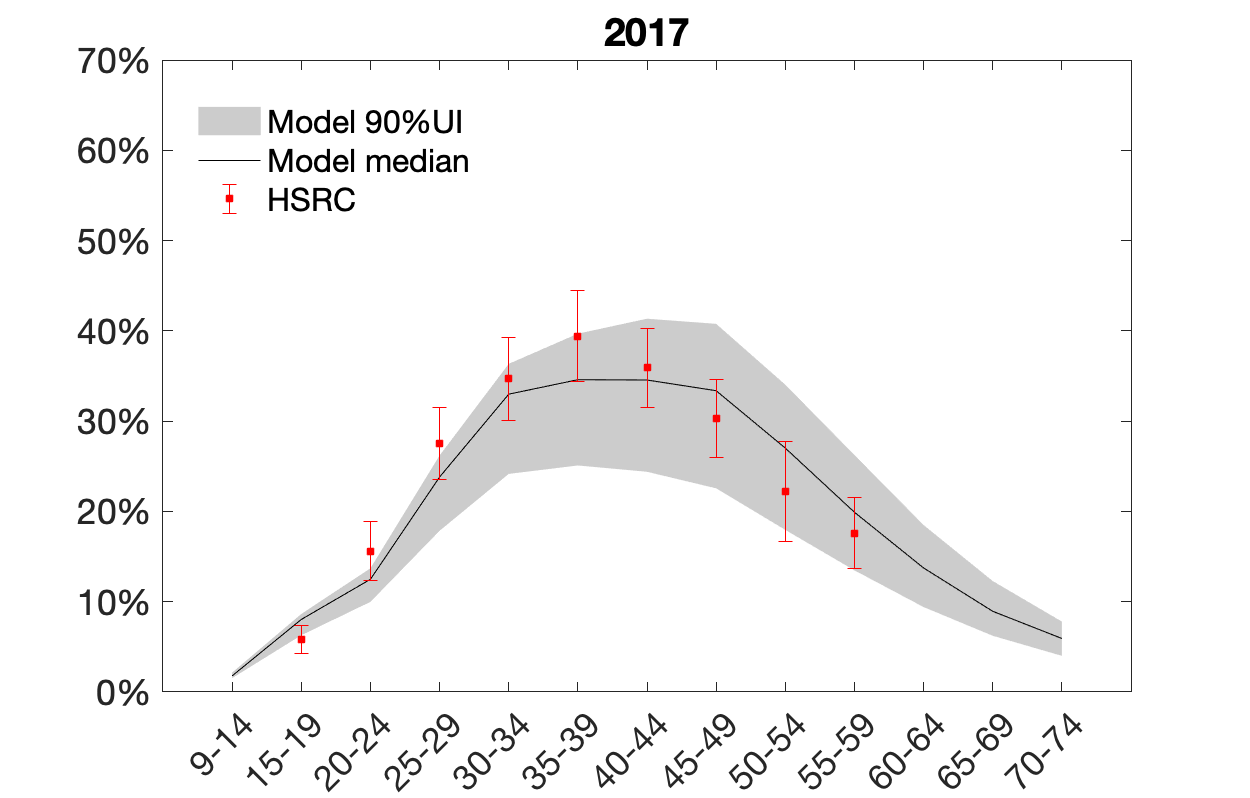** | 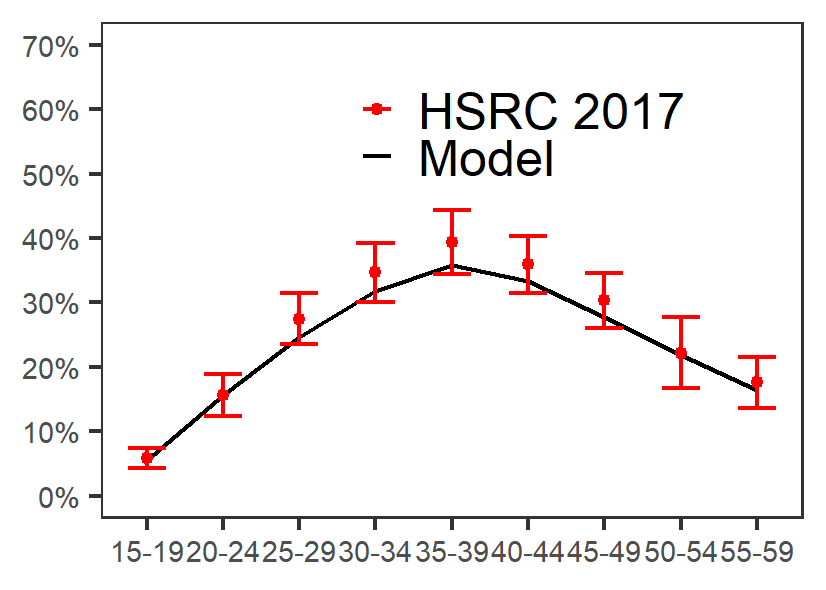 | **2016**  **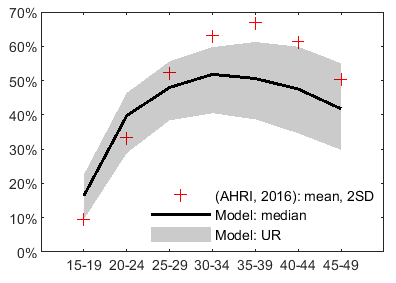** |
| **D) ART coverage in women over time***** | **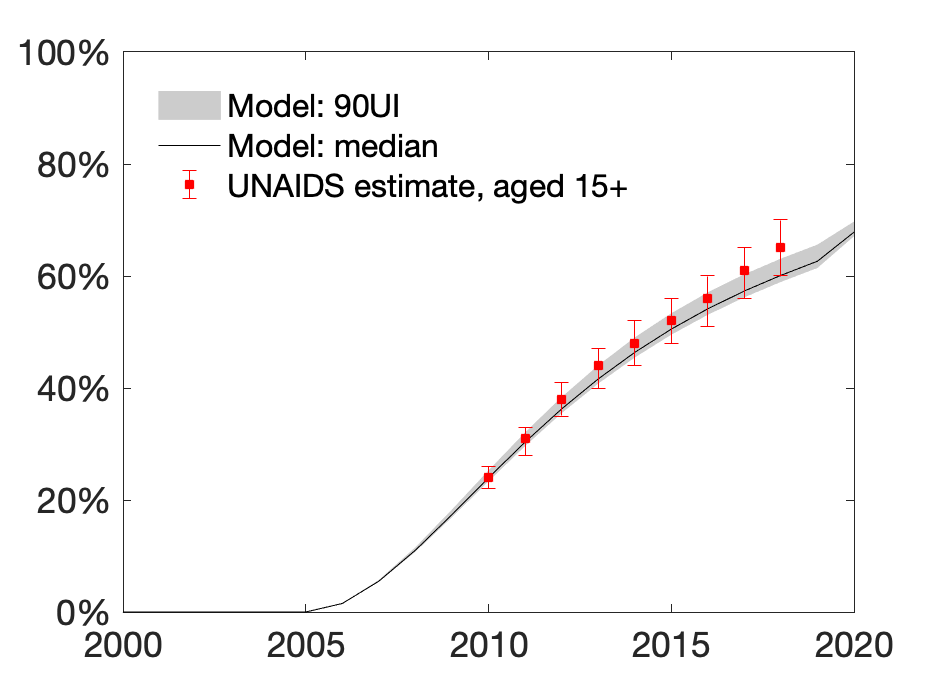** | 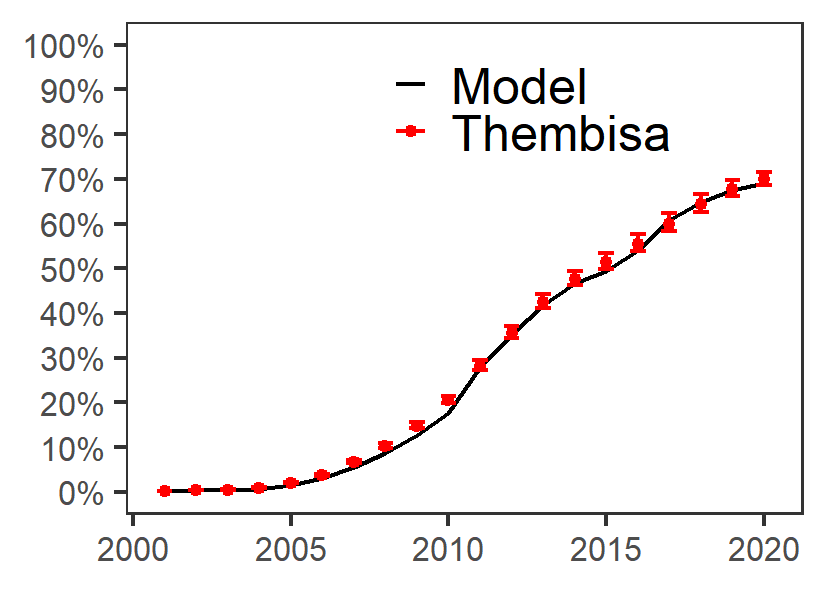 | **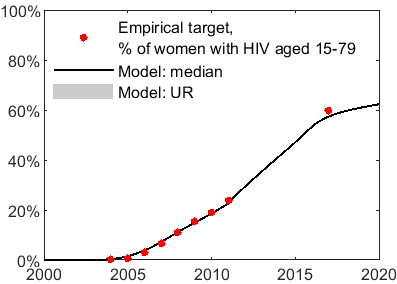** |
| **E) Cervical cancer incidence in 2018** | **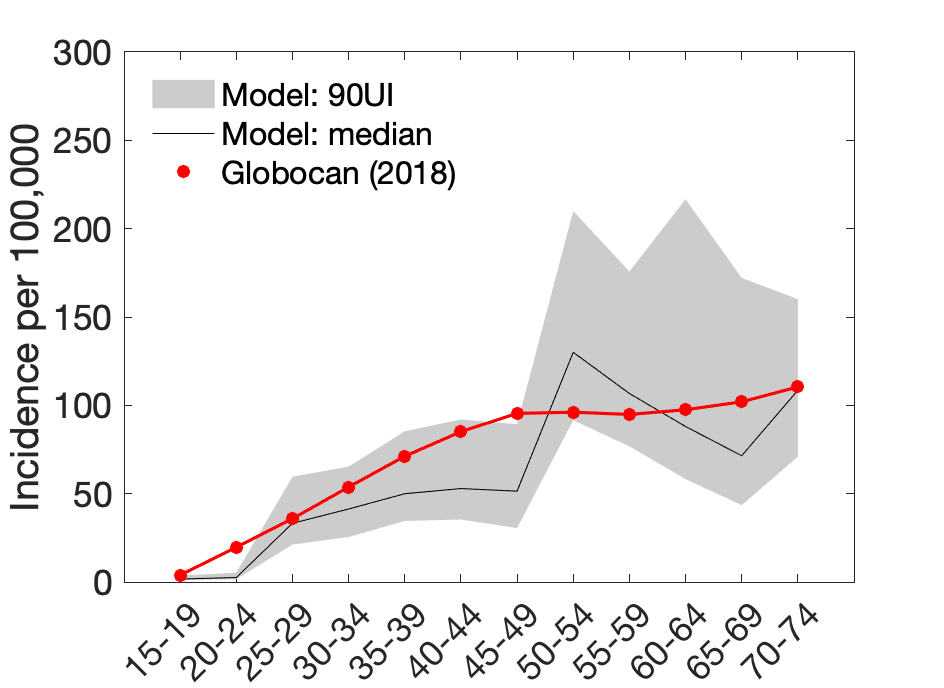** | 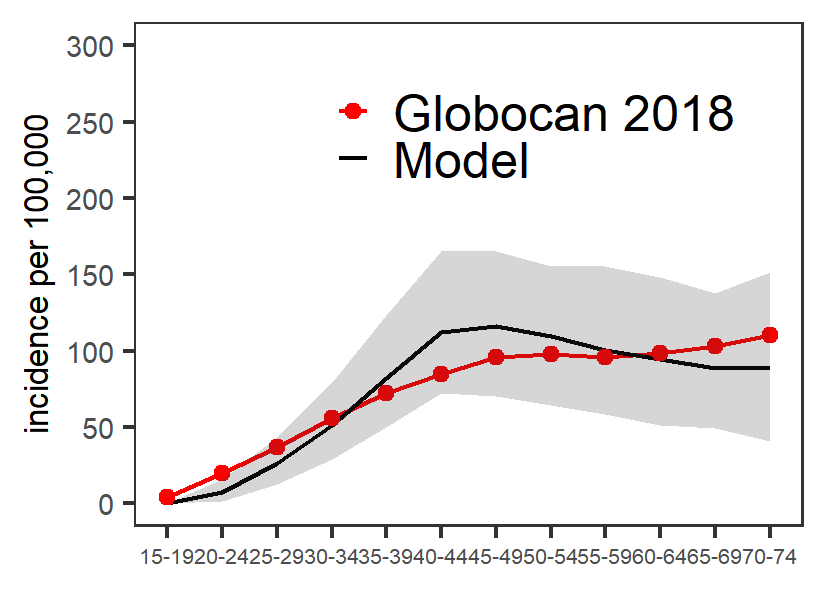 | **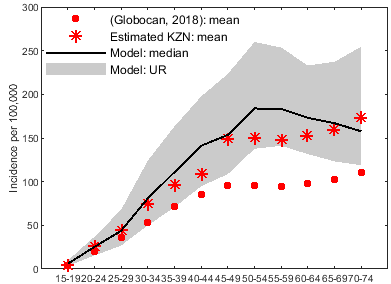** |
| **F) HR-HPV type distribution in women (2019-2020)*** | 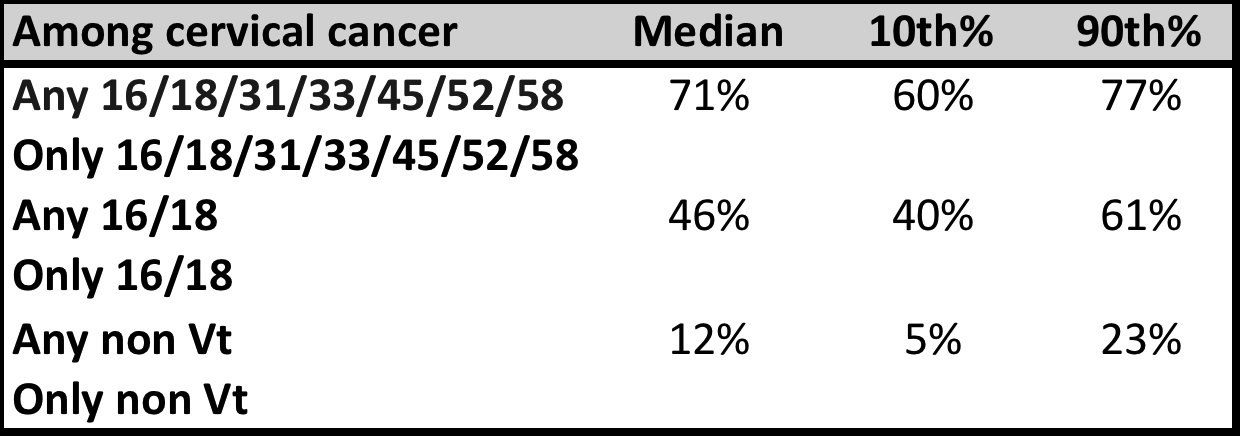 | 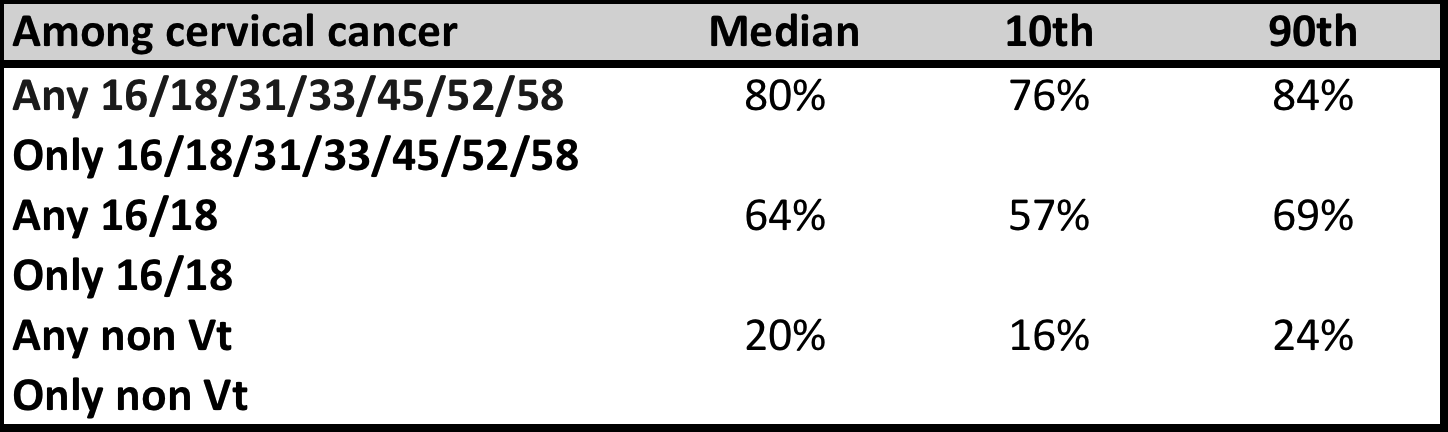 | 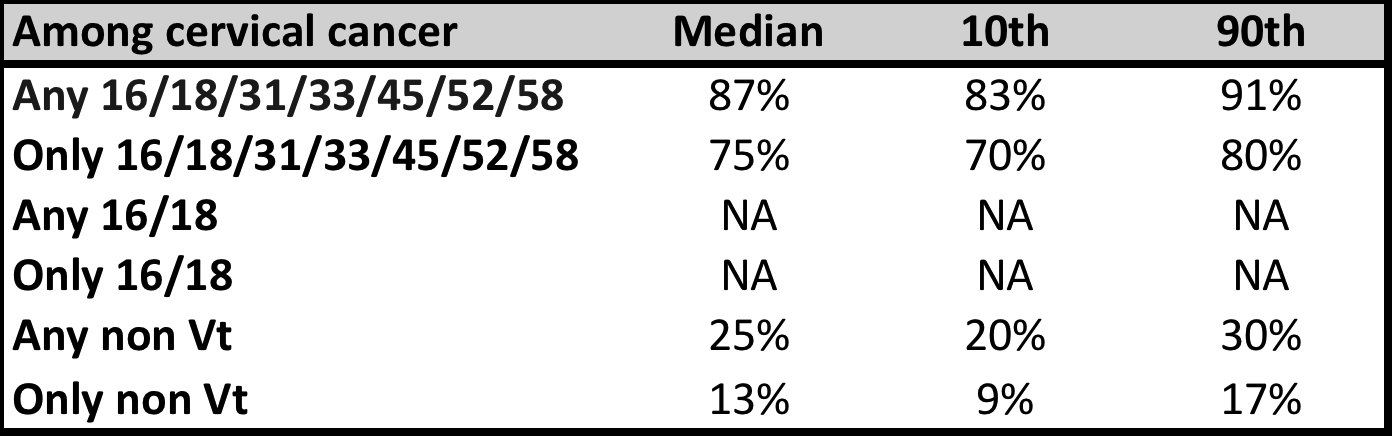  NA: 16/18 not modelled |

* See estimates Box2 for comparison; HR: High-risk

** For DRIVE model, red circles represent calibration data, red asterisks (Globocan, 2018) represent calibration data adjusted for KwaZulu-Natal, and red crosses represent cross-validation data (not used to fit the model but check model predictions afterwards.

*** For DRIVE, only model the effect of ART among individuals who achieve viral suppression: persons with HIV who initiate treatment without achieving viral suppression are assumed to have no benefit from treatment and are not tracked in the model.

**4) Model outcomes and standardisation**

Each calibrated model (based on the posterior parameter sets identified at the calibration stage) was used to predict different demographic, epidemiological, and intervention coverage and impact outcomes (e.g., population size, age distribution, prevalence/incidence of infections and disease, ART or vaccination intervention coverage) over time in the *basecase* and the scaled-up scenarios.

The population-level impact of each scenario was measured using three main age-standardised outcomes: i) the cervical cancer incidence over time, ii) the relative reduction in cervical cancer incidence compared to the *basecase* at each time point, and iii) the cumulative fraction of cervical cancer cases averted over different time periods since 2020 compared to the *basecase* scenario.

- Outcomes were age-standardised by applying the age distribution of the 2015 world female population aged 0-99 years(19) to the relevant median age-stratified outcome predicted by the model at each time point. For example, the standard age distribution was applied to the median age-stratified cancer incidence predicted by the model (IR(a,t)) to obtain the median age-standardised incidence (ASIR(t)).
- The relative reduction in age-standardised cervical cancer incidence at a given time point (RR_ASIR(t)) was estimated by comparing the predicted median age-standardised incidence rate in the intervention (ASIR_sc_(t)) and the *basecase* (ASIR_bc_(t)) scenarios at each time point as follows: RR_ASIR(t)= ((ASIR_bc_(t)-ASIR_sc_(t)) /ASIR_bc_(t)).
- The age-standardised fraction of cumulative cases averted over time (AF-ASCI(t)) was obtained by calculating the relative difference between the median age-standardised cumulative number of incidence cases in the *basecase* (ASCI_bc_(t_2020,_ t_2_)) and the interventions (ASCI_sc_(t_2020,_t_2_)) scenarios from 2020 onward up to the relevant time point (t_2_) as follows: (AF-ASCI(t) = ((ASCI_bc_(t_2020,_t_2_)-ASCI_sc_(t_2020,_t_2_))/ASCI_bc_(t_2020_)). The age-standardised cumulative number of cases from 2020 up to time t_2_ was obtained by applying the standard age distribution to the product of the predicted median age-specific cervical cancer incidence rates, $IR\left( a,t \right)$, and female population size, $N\left( a,t \right)$, and summing over age and time.

**5) Selected model outcomes – post 2020**

**Figure T5.1:** Median age standardised HIV prevalence over time across models in the *basecase* scenario that assumes that the UNAIDS 90-90-90 targets and 70% male circumcision are achieved by 2030.


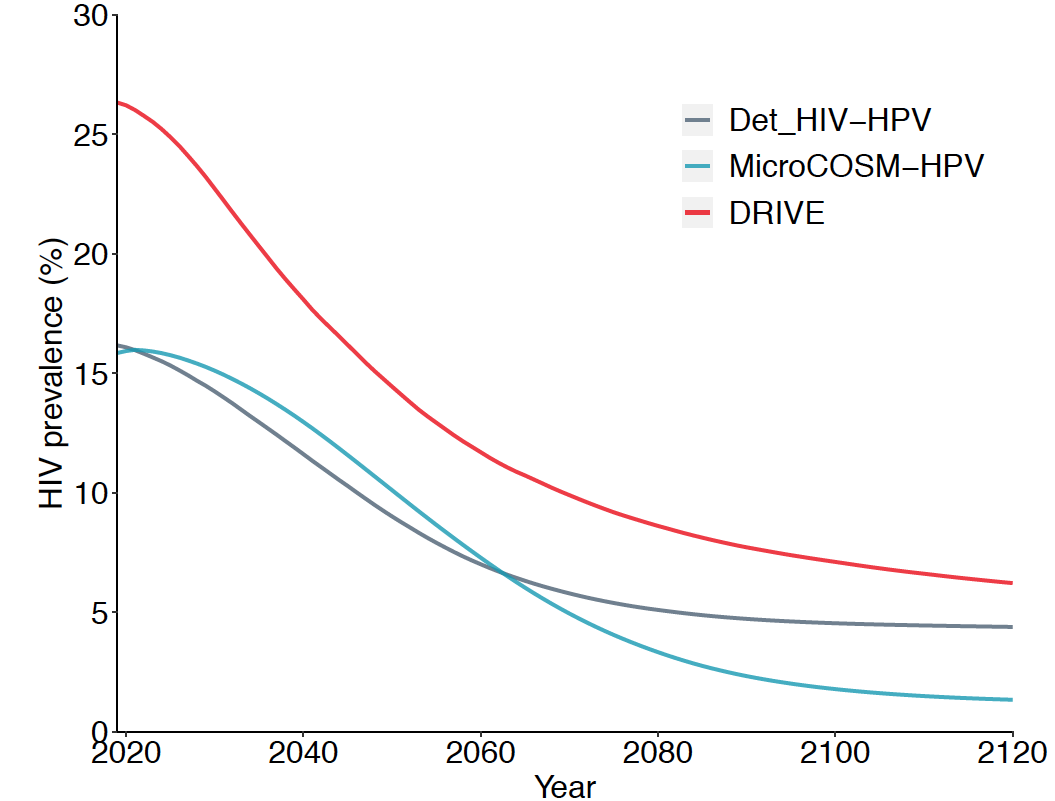


**Figure T5.2**: Overall age-standardised vaccination coverage over time among women aged 0-99 for each model for: girls’ vaccination (aged 9-14 years old, 90% coverage, Scenario 1) (black line), vaccination of young WLHIV (scenario 4: Scenario 1+ vaccination of WLHIV aged 15-24 years old, 90% coverage, dotted line). Results are presented for WLHIV (red) and HIV negative women (blue).


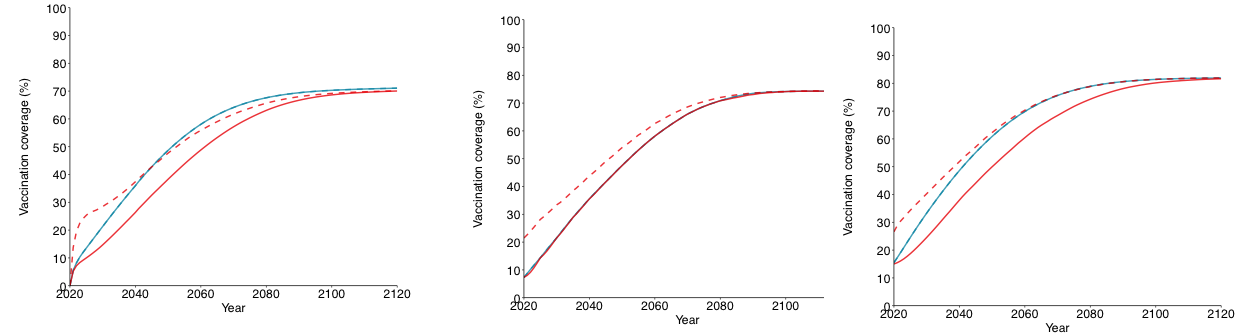


A) *Det_HIV-HPV model*

B) *MicroCOSM-HPV model*

C) *DRIVE* model

**Figure T5.3**: Comparison of the proportion of women aged 35-39 years that are screened annually (uptake) in the *basecase* scenario across the three models


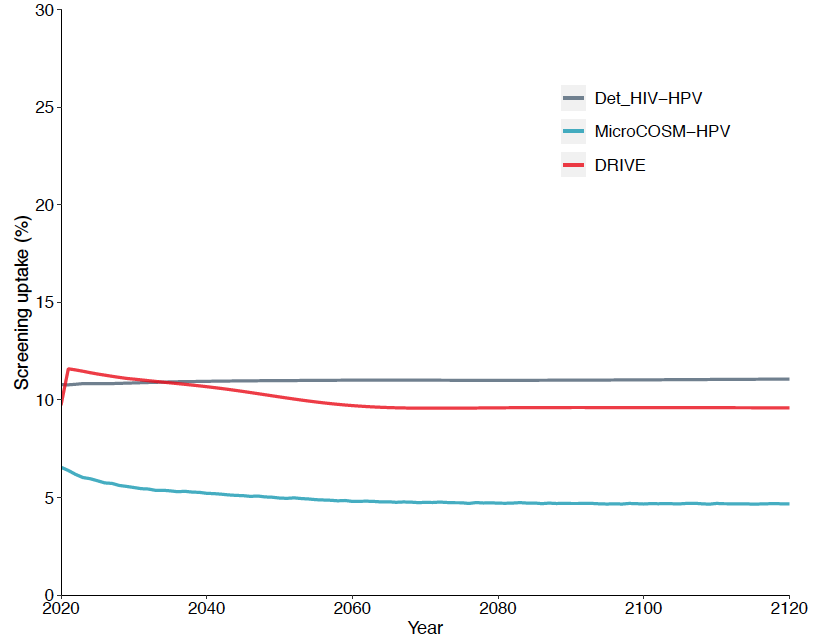


**Figure T5.4**: Proportion of women that are screened annually (uptake) per age group in the *basecase* scenario for the A) *Det_HIV-HPV* model (the 35-39 and 45-49 lines overlap), B) *MicroCOSM-HPV* model, and C) the *DRIVE* model

**A)** *Det_HIV-HPV B) MicroCOSM-HPV C) DRIVE*

**
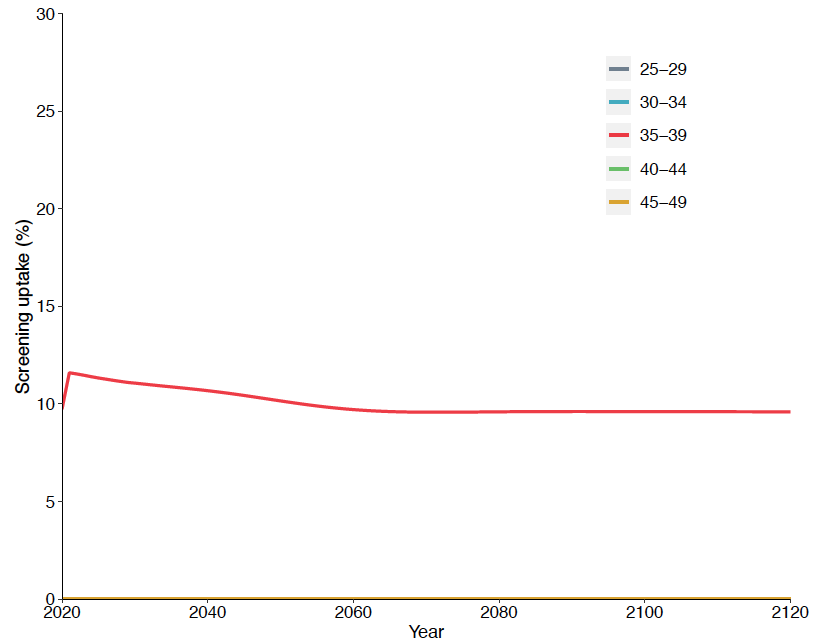
**
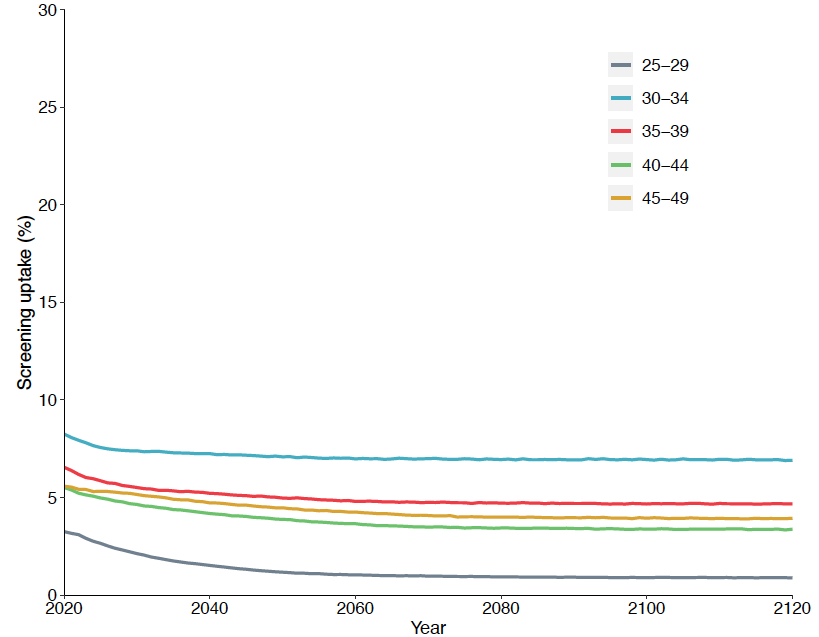

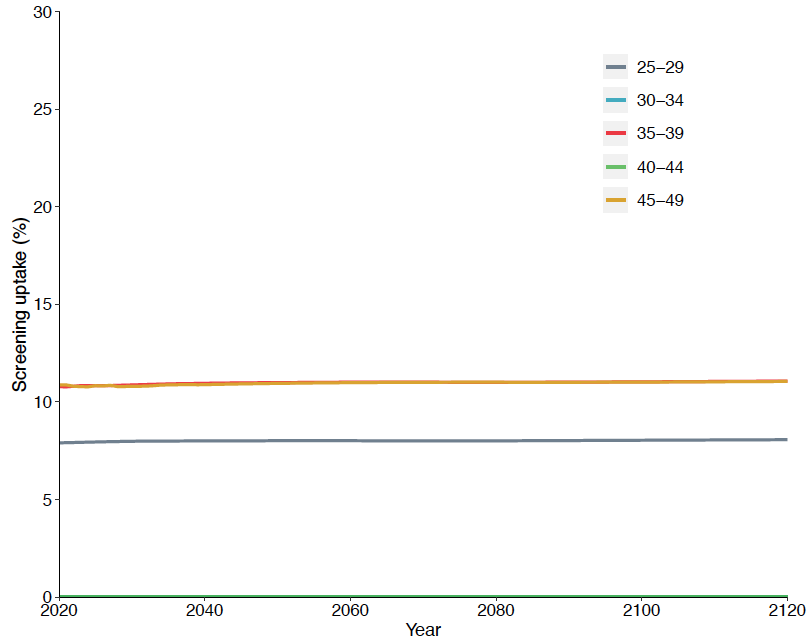


**6) References**

1. Delany-Moretlwe S, Kelley KF, James S, Scorgie F, Subedar H, Dlamini NR, et al. Human Papillomavirus Vaccine Introduction in South Africa: Implementation Lessons From an Evaluation of the National School-Based Vaccination Campaign. Glob Health Sci Pract. 2018 Oct 3;6(3):425–38.

2. Brisson M, Kim JJ, Canfell K, Drolet M, Gingras G, Burger EA, et al. Impact of HPV vaccination and cervical screening on cervical cancer elimination: a comparative modelling analysis in 78 low-income and lower-middle-income countries. The Lancet. 2020 Feb 22;395(10224):575–90.

3. District Health Barometer [Internet]. Health Systems Trust. Available from: https://www.hst.org.za/publications/Pages/HSTDistrictHealthBarometer.aspx

4. District Health Barometer [2012-2018]. Durban: Health Systems Trust; 2012 2018.

5. Makura CBT, Schnippel K, Michelow P, Chibwesha CJ, Goeieman B, Jordaan S, et al. Choropleth Mapping of Cervical Cancer Screening in South Africa Using Healthcare Facility-level Data from the National Laboratory Network. AIMS Public Health. 2016;3(4):849–62.

6. Shisana O, Rehle T, Simbayi L, Zuma K, Jooste S. South African National HIV Prevalence, Incidence and Behaviour Survey, 2012 [Internet]. HSRC Press; 2014 Jun [cited 2020 Jul 2]. Available from: https://www.hsrcpress.ac.za/books/south-african-national-hiv-prevalence-incidence-and-behaviour-survey-2012

7. South Africa Demographic and Health Survey 2016 [Internet]. Pretoria, South Africa, and Rockville, Maryland, USA: National Department of Health (NDoH) SSASS, South African Medical Research, Council (SAMRC); Available from: https://dhsprogram.com/pubs/pdf/FR337/FR337.pdf

8. van Schalkwyk C, Moodley J, Welte A, Johnson LF. Modelling the impact of prevention strategies on cervical cancer incidence in South Africa. Int J Cancer. 2021 Oct 15;149(8):1564–75.

9. Massyn N, Padarath A, Peer N, Day C. District Health Barometer 2016/17 [Internet]. Durban: Health Systems Trust; 2017 [cited 2021 Dec 8]. Available from: https://www.hst.org.za:443/publications/Pages/District-Health-Barometer-201617.aspx

10. Kelley K, James S, Delaney-Moretlwe S. Report of the HPV Vaccination Inter-Campaign Assessment. Findings and Recommendations. WITS Reproductive Health & HIV Institute; 2014.

11. Bruni L, Albero G, Serrano B, Mena M, Collado J, Gómez D. ICO/IARC Information Centre on HPV and Cancer (HPV Information Centre). Human Papillomavirus and Related Diseases in South Africa. Summary Report 22 [Internet]. 2021. Available from: https://hpvcentre.net/statistics/reports/ZAF.pdf

12. National Institute for Occupational Health, National Health Laboratory Service. Cancer in South Africa 2017 Full Report [Internet]. National Cancer Registry; 2020. Available from: https://www.nicd.ac.za/centres/national-cancer-registry/

13. Liu G, Sharma M, Tan N, Barnabas RV. HIV-positive women have higher risk of human papilloma virus infection, precancerous lesions, and cervical cancer. AIDS Lond Engl. 2018 Mar 27;32(6):795–808.

14. UN Department of Economic and Social Affairs, World Population Prospects: The 2019 Revision [Internet]. Available from: https://population.un.org/wpp/Download/Standard/Population/

15. United States Census Bureau International Database [Internet]. [cited 2022 Mar 20]. Available from: https://www.census.gov/data-tools/demo/idb/#/country?COUNTRY_YEAR=2022&COUNTRY_YR_ANIM=2022&FIPS_SINGLE=SF

16. Johnson L, Dorrington R. Thembisa 4.3: A model for evaluating the impact of HIV / AIDS in South Africa. 2020; Available from: www.thembisa.org

17. Mid-year population estimates 2019. Statistical Release P0302 [Internet]. Pretoria, South Africa; 2019. Available from: https://www.statssa.gov.za/publications/P0302/P03022019.pdf

18. Simbayi L, Zuma K, Zungu N, Moyo S, Miranda E, Jooste S. South African National HIV Prevalence, Incidence, Behaviour and Communication Survey, 2017 [Internet]. HSRC Press; 2017. Available from: http://www.hsrc.ac.za/en/research-outputs/view/10185

19. United Nations, Department of Economic and Social Affairs, Division P. World Population Prospects: The 2017 Revision [Internet]. [cited 2018 Oct 17]. Available from: https://esa.un.org/unpd/wpp/dataquery/
