# Supplementary material for: Estimating the effect of HIV on cervical cancer elimination in South Africa: Comparative modelling of the impact of vaccination and screening
Source: eClinicalMedicine. 2022 Nov 17;54:101754. doi: 10.1016/j.eclinm.2022.101754 (PMC9793279; doi:10.1016/j.eclinm.2022.101754)
Supplement: Model specific _Technical Appendix _T1 [file mmc7.docx]

**Model-Specific Technical Appendix T1:** Technical material for the *Det_HPV-HIV* model

*(National level)*

**Table of content**

Model overview………………………………………………………………………….…………….Page 1

Technical description of the *Det_HPV-HIV* model (South Africa) ……………………………… …..Page2

1. Demography, sexual behaviour and transmission…………………………………………………Page 2
2. Natural history of infection and disease progression…………………………………………….. Page 6
3. Baseline interventions – for HIV……………………………………………………………….....Page 8
4. Baseline interventions - for HPV and Cervical cancer…………………………………………... Page 9
5. Model calibration page……………………………………………………………………………Page 10
6. Parameter estimates and fitting/validations outcomes and data sources……………………….....Page 11
7. Description of analysis……………………………………………………………………………Page 28
8. Fitting and validation results……………………………………………………………………...Page 28
9. Model equations…………………………………………………………………………………..Page 41
10. References ………………………………………………………………………………………..Page 42

**Model overview**

The *Det_HPV-HIV model* is a model for South Africa at the national level. Briefly, the *Det_HPV-HIV* model is a deterministic transmission dynamic model that simulates heterosexual HIV and HPV transmission (3 groups of high-risk genotypes: HPV16/18, HPV 31/33/45/52/58, and high-risk HPV types not included in the nonavalent vaccine), HIV disease progression and HPV-induced cervical carcinogenesis associated with each type group in a heterosexual population that is stratified by sex, 5-year age groups, and 3 sexual activity levels. Each HPV related health state represents the underlying true health state of the simulated individuals (as opposed to a diagnosed state) such as infection and natural immunity status, high-grade cervical intraepithelial neoplasia (CIN2+), and cervical cancer. The model takes into account that HIV increases the risk of HPV infections and disease progression to cervical cancer. Persons living with HIV (PLHIV) who are on ART have a reduced risk of HPV and associated disease progression compared to PLHIV not on ART, but they have a higher risk compared to HIV negative individuals. We also allow prevalent HPV infection to increase HIV acquisition risk.

The model represents variation in the levels of HIV interventions such as condom use, male circumcision, and HIV treatment (ART) of people living with HIV (PLHIV) by sex, age, and sexual activity level over time since the beginning of the HIV epidemic in 1985. The model represents cervical cancer screening and treatment (starting in 2017) in the base case scenario. HPV vaccination is not included in the basecase scenario, and it is introduced in 2020 in the interventions including vaccination.

The risk of infection among individuals susceptible to the infection (HIV, or one of the three grouped HPV types) depends on individuals’ sexual activity, condom use (women and men), the prevalence of infection among partners, and HIV/ART or HPV status, number of unprotected sex acts, and level of interventions. The transition rates between health states depend on sex, age, HPV type group, and HIV/ART status.

The model was parameterized and fitted using multivariate calibration process based on detailed data on sexual behaviour, HIV and HPV infections and cervical cancer epidemiology over time representative of the South African context. The data sources used at this stage are described below.

**Technical description of the Det_HPV-HIV** **model (South Africa)**

1. **Demography, sexual behavior, and transmission**

The compartmental model simulates an open and growing heterosexual population aged 9-74 representative of the population of South Africa. The model is parameterized to represent heterosexual women and men, stratified in five-year age groups (first age group is 9-14 years, and five-year age groups up to age 74). Individuals can leave the population either through background mortality, or HIV and cervical cancer associated mortality. Population growth and population demography are calibrated to reflect UN estimates of the South African population between 1950-2100(1).

The population is also stratified by sex and sexual activity. Each sex has three 3 sexual activity classes (low medium and high), defined by number and type of partners (partner change rate), which also varies by age. The highest activity level represents female sex workers (FSW) and clients of FSWs for women and men, respectively. The model is parameterized to reflect South African population with sexual activity parameterized to reflect the proportion of individuals who have ever had sex. Sexual activity levels vary by sex and age and are informed by South African Bio-Behavioural Surveillance Survey (SABBSM) data-analysis using the 2005 survey(2,3). The prior range for the proportion of women who are commercial sex workers (FSW), and their average number of male clients were sourced from a review of FSW in the South African population(4). There are two types of partnerships: each sexually active class can have non-commercial sex partnerships, and FSW and clients can also have commercial partnerships. There is a number of sex acts per partnership(3). As non-commercial sex partnerships are assumed to last longer than commercial partnerships, they have more sex acts than commercial partnership. Similarly, higher activity levels have more partnerships than low activity level, and they are assumed to be of shorter duration and to have fewer sex acts. To derive the number of sex acts per non-commercial partnerships, we divide the sampled number of acts per partnership by the group’s respective yearly partnership rate, which assumes that people with very few partners have a longer partnership duration and their number of sex acts per partnership is higher. In commercial partnerships, the number of sex acts did not dependent on the number of partners the FSW or clients.

We distinguished between vaginal (VI) and anal intercourse (AI), with associated proportion of acts that are of each type. We model condom use over time, with partial efficacy, which varies by activity level, whether the partnership is commercial or non-commercial, type of sexual act (AI, VI) and by age. Sexual mixing in non-commercial partnerships is defined by activity level and by age (Figure 1, equations below). The sexual mixing matrix is defined by age, and within the age by sexual activity level. Prior ranges for the parameters governing age-mixing were informed by SABBSM 2005 survey(2,3) secondary data-analysis for the population aged 15 and older, abased on the following age categories: 9-14, 15-24, 25-34, 35-49, 50-74(5).

In the model, women can have partners within their own age group or older age groups, and men can have partners of their own age group or within younger age groups. For disassortative age mixing, we set the upper values of the ranges for the proportion of partners with the next age group to the maximum possible proportion of each age group reporting partners who were at least 5 years older (for women), and younger (for men) (Figure 1A) to include full uncertainty in age mixing while conforming to age patterns reported in SABBSM. Reflecting data, we also allowed for a small proportion of partnerships to be with partners who are significantly older (or younger). For the population aged 9-14, we use HPTN068 data (data are for 13-14 year-olds) to inform the proportion of the age group who are sexually active and their age-mixing patterns (Pettifor, personal communication; further details in Table 6.1 ).

$$m_{jlmn}=\varepsilon_{jm}^{1}*\left( \varepsilon^{2}d_{ln}+(1-\varepsilon^{2})\frac{c_{mn}N_{mn}}{\sum_{n} c_{mn}N_{mn}} \right)$$

Mixing matrix in non-commercial relationships ($m_{jlmn}$) is defined by mixing by age ($\varepsilon_{jm}^{1})$, and within age, mixing by sexual activity level ($\varepsilon^{2})$. With people of age *j*, and sexual activity *l*, partnering with opposite sex people of age *m*, and of sexual activity level *n*. Where $c_{mn}$ is the partner change rate, and $N_{mn}$ is the number of people in the respective age-activity category. The Kronecker delta for the sexual activity category (δ_ln_=1 if l*=n* and δ_ln_=0 if *l≠n*). The number of partnerships between men and women who have non-commercial partnerships are balanced at each time step using the Garnett-Anderson method, with balancing factor ($K)$ set to 0.5 (equal balance by both sexes).(6)

$$c_{jlmn}^{*}=c_{jl}\left( \frac{c_{mn}m_{mnjl}N_{mn}}{c_{jl}m_{jlmn}N_{jl}} \right)^{K}$$

$$c_{mnjl}^{*}=c_{mn}\left( \frac{c_{mn}m_{mnjl}N_{mn}}{c_{jl}m_{jlmn}N_{jl}} \right)^{-(1-K)}$$

FSW and clients can have non-commercial partners in addition to commercial partners. Non-commercial partnerships are governed by the same age-mixing principles as the other non-commercial partnerships. Number of commercial partners between FSW and clients is dependent on availability of FSW commercial partnerships (i.e. annual number of assigned clients and size of FSWs population), because we believe that the self-reported data on the number of clients of FSW is less biased than the number of commercial partners that clients have(4). Given there are substantially larger number of clients than FSW in the model, the clients have fewer commercial partners than the FSW. Age mixing in commercial partnerships is independent of age. Thus we defined prior ranges for the annual number of clients by age, and sizes of the FSW and clients population. In commercial partnerships, the number of partnerships available from FSW determines the number of commercial partners clients can have, for

$$c_{n}^{*}=\frac{c_{l}N_{l}}{N_{n}}$$

**
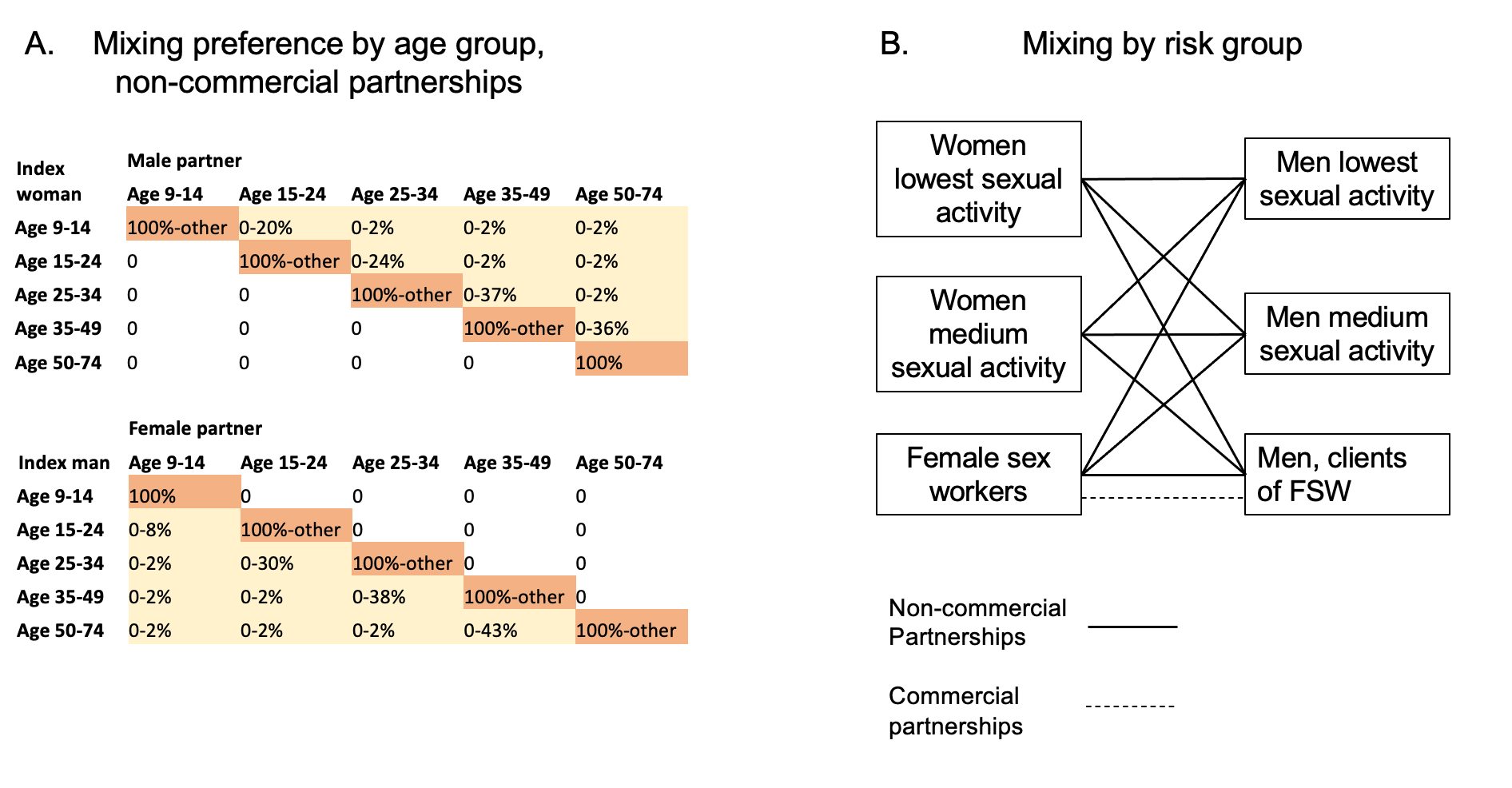
**

**Figure 1.1 A).** Sexual mixing prior parameter ranges are defined for the age groups: 9-14, 15-24, 25-34, 35-49 and 50-74 (A), where the row values sum to 100% with preference for age-assortative mixing, and within age groups mixing is defined by sexual activity level (B). Age mixing parameter corresponds to $\varepsilon_{jm}^{1}$while risk mixing ($\varepsilon^{2}$) within age group is allowed to vary from assortative to proportionate mixing between activity levels. Mixing for commercial partners is independent of age and determined by number of partnerships available from the FSW.


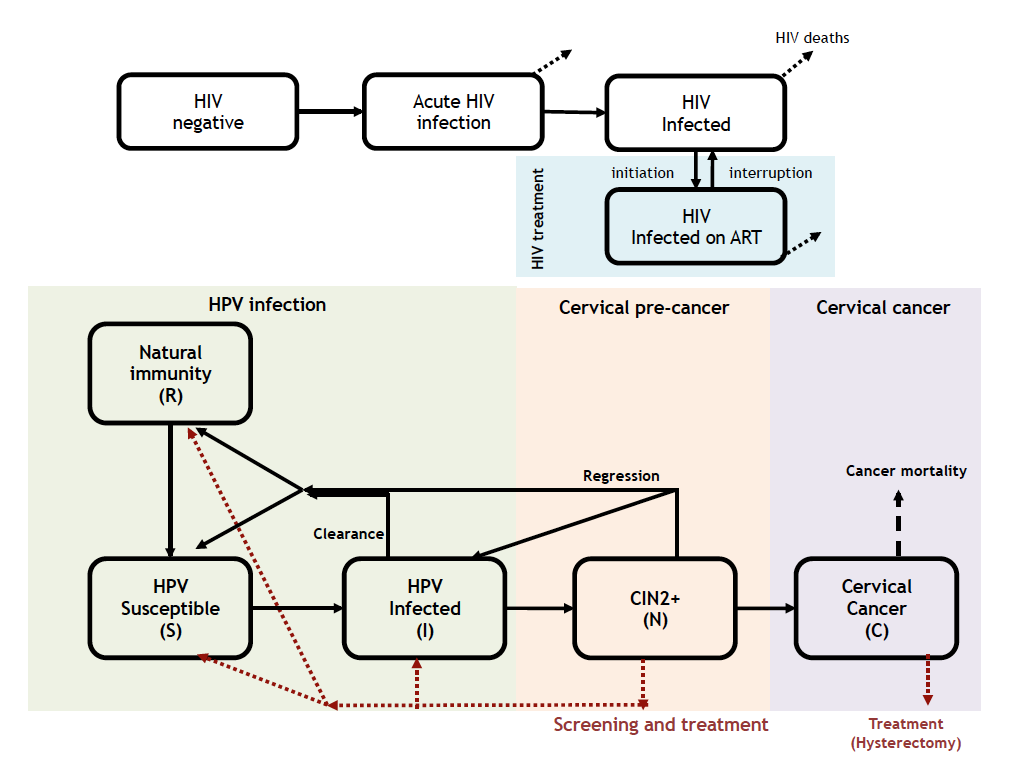


**Figure 1.1 B.** Flowchart of the natural history of HIV and HPV infections and associated disease progression shown in black arrows. Cervical cancer screening and treatment are shown in red dashed arrows. The model includes co-infection between HIV and HPV, and between different grouped HPV types, background mortality and new recruits to the sexually active population (not represented on the graph). Interactions considered among PLHIV include: 1) an increase in HPV susceptibility (HPV acquisition risk), 2) a longer persistence of HPV infection (reduced clearance rate), 3) faster rate of progression from the CIN2+ state to cancer, 4) slower rate of natural regression from CIN2+, and 5) shorter duration of natural immunity among PLHIV. We also assumed an increased HIV acquisition risk in presence of HPV infection

**HIV force of infection***:* The per capita HIV force of infection of a susceptible individual of a given sex, sexual activity level and age (i.e. the per capita rate of infection) is a time varying function of its sexual partner change rate, number and type of sex acts, transmission probability per type of sex act, cofactors effect due to HPV infection, mixing matrix (by sex, age, and activity level), the prevalence of infection in their partner pool and level of intervention. The element of the mixing matrix determines the probability that an individual of sex k, age a and sexual activity class s choose a non-commercial partners of opposite sex and age a’ and activity class. The force of infection can be simplified as:

$$\kappa_{h,s,r,a}=c_{jlmn}^{*}m_{jlmn}\sum_{i',m,n} \beta_{h,i^{'}, s,r,a}\frac{Q_{i'mn}^{'}}{N_{mn}^{'}}$$

Where$\beta_{h,i^{'},s,r,a}$ is the transmission probability, and $i^{'}$ is the HIV infection state of the opposite sex partner, and HPV infection status (h), sex (s), sexual activity level (r) and age (a) of the susceptible person also impact transmission probability. Q’ is the HIV infected health states in the opposite sex population (N), of age m and risk category n. For FSW and clients, the force of infection from commercial and non-commercial partnerships is additive.

Transmission probability depends on the type of sex acts (VI and AI), the HIV stage of the partner, number of sex acts per partnership, and whether the act is protected by condoms or not (with associated condom use efficacy by type of act). The transmission probability per sex act is higher from male to female than from female to male and if the HIV susceptible individual is co-infected with HPV infection. Per partnership transmission probability is defined as a Bernoulli equation, where number of acts are modeled by the proportion of acts that are vaginal (1-$p_{r}$, t=1) vs anal ($p_{r}$, t=2), and within the act type, proportion of acts that are protected by condoms ($u_{t,r,a}$):

$\beta_{h,i^{'},s,r,a}= 1-[$ …

$$\left( 1-\boldsymbol{\beta}_{\boldsymbol{h,i' ,s,t=1}}^{\boldsymbol{HIV}} \right)^{n_{ra}\left( 1-u_{t=1,r,a} \right)\left( 1-p_{r} \right)}\left( 1-\boldsymbol{\beta}_{\boldsymbol{h,i',s,t=1}}^{\boldsymbol{HIV}}\left( 1-\iota_{t=1} \right) \right)^{n_{ra}\left( u_{t=1,r,a} \right){(1-p}_{r})}$$

$$\left( 1-\boldsymbol{\beta}_{\boldsymbol{h,i',s,t=2}}^{\boldsymbol{HIV}} \right)^{n_{ra}\left( 1-u_{t=2,r,a} \right)p_{r}}{(1-\boldsymbol{\beta}_{\boldsymbol{h,i',s,t=2}}^{\boldsymbol{HIV}}(1-\iota_{t=2}))}^{n_{ra}\left( u_{t=2,r,a} \right)p_{r}} ]$$

We model time-varying changes in condom use and VMMC coverage. We account for voluntary medical male circumcision (VMMC) in the model as an average reduction in transmission probability in the HIV acquisition probability among men, which was modeled as the proportion of adult men circumcised and efficacy of VMMC against HIV acquisition.

**HPV force of infection (**$\lambda_{isra})$**:** The per capita time varying HPV force of infection is a time-varying function of a person’s partner change rate, age and sexual activity mixing matrix, transmission probability and the prevalence of infection in their partner pool ( $\frac{\sum_{h} P_{hmn}^{'}}{N_{mn}^{'}}$ , where $\sum_{h} P_{hmn}^{'}$ reflects the sum of HPV disease stages (h) where HPV is infectious). HPV transmission is modelled as transmission probability per partnership ($\beta_{h,i}^{HPV}$) given the high infectivity of HPV infection, which means that transmission is less influenced by the type or frequency of sexual acts and can explain low impact of imperfect condom use against HPV. Transmission probability remains constant across HPV infection stages but differs by HPV types: HPV-16/18 have the highest transmission probability and HPV-31/33/45/52/58 and nvt-HR HPV have transmission lower probabilities relative to HPV-16/18. As the data on the efficacy of condom-use against HPV acquisition is inconsistent and very low, we did not include protective effect(7,8). We also assumed male circumcision was not protective against HPV acquisition for men. We modelled an increased transmission probability for HPV among the HIV infected persons exposed to HPV.

$$\lambda_{isra}=c_{jlmn}^{*}m_{jlmn}\beta_{h,i}^{HPV}\sum_{m,n} \frac{\sum_{h} P_{hmn}^{'}}{N_{mn}^{'}}$$

1. **Natural history of infection and disease progression**

The model represents disease acquisition and progression of HIV and three grouped HPV types: i) HPV- 16/18, ii) HPV- 31/33/45/52/58 and iii) other non-vaccine type high-risk HPV types, and co-infections between these groups. The discrete health states include susceptible, infected, partial natural immunity, as well as the development of cervical intraepithelial neoplasia (CIN2+), which can develop to cervical cancer. Natural history of HIV represents HIV disease progression from susceptible to infection to treatment, transitioning through phases of varying infectivity when on and off ART, and associated deaths.

**HIV natural history***:* HIV is introduced in the model in 1985 with the initial number (i.e. epidemic seed) of people living with HIV varied in the calibration. Differential equations for HIV progression below, parameter values specific for HIV in table 6.2. HIV disease progression beings by force of infection ($\kappa_{h,s,r,a}$ ) moving those who acquire infection from susceptible (Z) to acute infection (Y), and from acute infection to post-acute HIV infection, at rate $\xi$, where people are not on ART (K), or on ART (T) with associated ART-initiation rate ($\tau$) and rate of stopping ART ($o_{s,r,a}).$ People can move back and forth between the ‘on ART’ and ‘not on ART’ compartments reflecting ART initiation and discontinuation, which depend on age and sex of the individual. FSW have higher rates of ART discontinuation. As proportion of people living with HIV on ART are virally suppressed; transmission probability in this health state is a weighted average of virally suppressed and non-suppressed. People on ART have a reduced HIV transmission probability and HIV-associated mortality rate ($\eta_{i})$, compared to people not on ART. There is also background mortality ($\mu_{s,a})$. The differential equations show the HIV natural history, and the equations do not show the underlying HPV natural history which is noted in the equations with subscript h (to describe the three grouped HPV types and their health states). Additionally, we allow a proportion of children entering the model age group 9-14 to be living with HIV to account for perinatally infected children who have survived until age 9, from 1998 onward as informed by the literature(9), these are omitted from the equations.

$$\frac{d\boldsymbol{Z}_{h,s,r,a}}{dt}= -(\kappa_{h,s,r,a}+\mu_{s,a})Z_{h,s,r,a}$$

$$\frac{d\boldsymbol{Y}_{h,s,r,a}}{dt}= \kappa_{h,s,r,a}Z_{h,s,r,a}-(\eta_{i}+\mu_{s,a}+\xi)Y_{h,s,r,a}$$

$$\frac{d\boldsymbol{K}_{h,s,r,a}}{dt}= \xi Y_{h,s,r,a}-\tau K_{h,s,r,a}+ o_{s,r,a}T_{h,s,r,a}-(\eta_{i}+\mu_{s,a})K_{h,s,r,a}$$

$$\frac{d\boldsymbol{T}_{h,s,r,a}}{dt}= \tau K_{h,s,r,a}-o_{s,r,a}T_{h,s,r,a} -(\eta_{i}+\mu_{s,a})T_{h,s,r,a}$$

**HPV and cervical cancer natural history:** We model high-risk HPV in the following grouped HPV types, and co-infection between the different types: i) HPV- 16/18, ii) HPV- 31/33/45/52/58 and iii) other non-vaccine type high-risk HPV types, and explicitly represent all plausible co-infections between HPV types and HPV and HIV. Following infection with a HR-HPV type, susceptible women transition to the infected stage (I), from which they can naturally clear the infection, at rate $\sigma_{h,i,s}$ , or progress to CIN2+ health state (N), at rate $\psi_{h,i,s}$ , with rate dependent on the grouped HPV type and HIV status. CIN2+ can clear through natural regression or through successful cervical cancer screening and treatment ($\theta_{i,s,a})$. Natural regression rate ($\omega_{h,i,s,a}$) is age dependent. A proportion ($q$) of CIN2+ who regress also clear their infection. CIN2+ can progress to cervical cancer (C) at a rate $\pi_{s,a}$which is age dependent, and there is no natural regression from the cervical cancer health state. People with cervical cancer have cervical cancer -associated mortality ($\upsilon_{s})$, and they may get diagnosed with cervical cancer. If they are successfully treated for cancer (via hysterectomy), they are no longer assumed to be at risk of developing cervical cancer and they are removed from the model. Natural immunity (R) can develop in a proportion (m) of individuals who clear the infection. Natural immunity is complete but wanes over time at rate $\delta_{h,i}$, and waning depends on the grouped HPV type, can be slower or faster between grouped types (10), and on HIV status.

We assume that HPV-16/18 transmission probability is higher than for other HPV grouped types, and clearance from HPV-16/18 pre-CIN2+ stage is faster than for other types to reflect the grouped nature modeled (with more HPV types in the other grouped categories) Progression to CIN2+ is faster and regression from CIN2+ is slower for HPV 16/18 than for the other grouped HPV types.(10–12) Cervical cancer progression rate from CIN2+ is independent of HPV types. We calibrated the model to reflect the type distribution at different stages of cervical cancer development (HPV, CIN2+ and cervical cancer; figure 7.5). Below are the differential equations for women who are susceptible for HPV- 31/33/45/52/58 (S^2^) and susceptible for other non-vaccine type high-risk HPV types (S^3^) but are in different health states regarding HPV-16/18 infection status (S^1^, I^1^, R^1^, N^1^, C^1^). Rate $\alpha_{a}$ represents the aging process.

$$\frac{d{\boldsymbol{S}^{\boldsymbol{1}}\boldsymbol{S}^{\boldsymbol{2}}\boldsymbol{S}^{\boldsymbol{3}}}_{i,s,r,a}}{dt}=\alpha_{a-1}{S^{1}S^{2}S^{3}}_{i,s,r,a-1}- {(\alpha}_{a}{{+\lambda_{i,s,r,a}^{1}+\lambda_{i,s,r,a}^{2}+\lambda_{i,s,r,a}^{3})S}^{1}S^{2}S^{3}}_{i,s,r,a}+{\left( 1-m \right)\sigma}_{h,i,s}{I^{1}S^{2}S^{3}}_{i,s,r,a}+(1-m)\left( q\omega_{h,i,s,a}+{z\theta}_{h,s,a} \right){N^{1}S^{2}S^{3}}_{i,s,r,a}+\delta_{h,i}{R^{1}S^{2}S^{3}}_{i,s,r,a}$$

$$\frac{d{\boldsymbol{I}^{\boldsymbol{1}}\boldsymbol{S}^{\boldsymbol{2}}\boldsymbol{S}^{\boldsymbol{3}}}_{i,s,r,a}}{dt}=\alpha_{a-1}{I^{1}S^{2}S^{3}}_{i,v,s,r,a-1}- {(\alpha}_{a}{{+\lambda_{i,s,r,a}^{2}+\lambda_{i,s,r,a}^{3}+\sigma_{h,i,s}+\psi_{h,i,s})I}^{1}S^{2}S^{3}}_{i,s,r,a}+\lambda_{i,s,r,a}^{1}{S^{1}S^{2}S^{3}}_{i,s,r,a}+(1-z)\theta_{s,a}{N^{1}S^{2}S^{3}}_{i,s,r,a}+{(1-q)\omega}_{h,i,s,a}{N^{1}S^{2}S^{3}}_{i,s,r,a}$$

$$\frac{d{\boldsymbol{R}^{\boldsymbol{1}}\boldsymbol{S}^{\boldsymbol{2}}\boldsymbol{S}^{\boldsymbol{3}}}_{i,s,r,a}}{dt}=\alpha_{a-1}{R^{1}S^{2}S^{3}}_{i,s,r,a-1}- {(\alpha}_{a}{{+\lambda_{i,s,r,a}^{2}+\lambda_{i,s,r,a}^{3}+\delta_{h,i})R}^{1}S^{2}S^{3}}_{i,s,r,a}+{m\sigma}_{h,i,s}{I^{1}S^{2}S^{3}}_{i,s,r,a}+m\left( q\omega_{h,i,s,a}+{z\theta}_{h,s,a} \right){N^{1}S^{2}S^{3}}_{i,s,r,a}$$

$$\frac{d{\boldsymbol{N}^{\boldsymbol{1}}\boldsymbol{S}^{\boldsymbol{2}}\boldsymbol{S}^{\boldsymbol{3}}}_{i,s,r,a}}{dt} =\alpha_{a-1}{N^{1}S^{2}S^{3}}_{i,s,r,a-1}-{(\alpha}_{a}{{+\lambda_{i,s,r,a}^{2}+\lambda_{i,s,r,a}^{3}+\omega_{h,i,s,a}+\theta_{i,s,a}+\pi_{s,a})N}^{1}S^{2}S^{3}}_{i,s,r,a}+\psi_{h,i,s} {I^{1}S^{2}S^{3}}_{i,s,r,a}$$

$$\frac{d{\boldsymbol{C}^{\boldsymbol{1}}\boldsymbol{S}^{\boldsymbol{2}}\boldsymbol{S}^{\boldsymbol{3}}}_{i,s,r,a}}{dt}=\alpha_{a-1}{C^{1}S^{2}S^{3}}_{i,s,r,a-1}- {(\alpha}_{a}+\upsilon_{s}+\lambda_{i,s,r,a}^{2}+\lambda_{i,s,r,a}^{3}+\theta_{i,s,a}){C^{1}S^{2}S^{3}}_{i,s,r,a}+\pi_{s,a}{N^{1}S^{2}S^{3}}_{i,s,r,a}$$

**Co-infections and effect of ART on HPV and diseases:** No interactions are assumed between the HPV types (e.g. no cross-protection or changes in susceptibility or natural history in presence of another HPV type). A number of interactions are assumed between HPV and HIV. The interactions of HIV on HPV have the same relative magnitude of effect for each grouped HPV type (i.e. the relative increase in HPV acquisition risk in PLHIV is the same irrespective of HPV type) and are independent of the number of co-infections with different types (i.e. HPV impact on HIV is not larger if an individual is infected by more than one grouped HPV type present).

Based on by systematic reviews and meta-analyses (13–15) the different interactions considered includes : 1) an increase in HPV susceptibility (i.e HPV acquisition risk) and 2)a longer persistence of HPV infection (reduced clearance rate) , 3) faster rate of progression from the CIN2+ state to cancer , 4) slower rate of natural regression from CIN2+, and 5) shorter duration of natural immunity among PLHIV, than in HIV-negative individuals^.^

Finally, we also assumed an increased HIV acquisition risk in presence of HPV infection(15).

We assume that HIV-HPV interactions are partially reduced in PLHIV who are on ART compared to PLHIV,(13) as not on ART as current evidence from systematic reviews suggest that they still remain at slightly higher risk compared to HIV negative individuals (i.e. HPV infection and disease progression rates among PLHIV do not return to the rates for HIV-negative individuals). Specifically we assume that:

i) being on ART partially reduces the risk of HR HPV acquisition

ii) being on ART partially reduces the risk of disease progression in HPV infected individuals

iii) being on ART partially increases the chance of regression

iv) being on ART partially increases duration of natural immunity

1. **Baseline interventions – for HIV**

**Condom use:** Condom use reduces the risk of HIV acquisition(16). We do not model the protective effect of condom use against HPV, for which the evidence is less clear(8). We initially represent low level of condom use which increases over time. We vary the timing of changes in condom use to reflect uncertainty in condom use trends prior to the years 1995 to 1998, followed by a linear increase until 2005-2010, after which condom use stays stable at achieved levels. This reflects the condom use campaigns in South Africa where condom use promotion programs began in the 1985-1998(17). Coverage varies by sexual activity level, but all groups experience the same time trend for increasing coverage. Condom use coverage varies by commercial and non-commercial partnerships, by age, and by act type (VI, AI). We assume the condom efficacy is the same for women and men. Condom use is modeled the same way in the base case and counterfactual analyses.

**Male circumcision**: Voluntary medical male circumcision reduces the risk of HIV infection in the model(18) The model reflects changes in the levels of male circumcision over time in adult men overall. We include an increase in VMMC coverage between 2007-2030 reflecting the impact of a national VMMC program. In 2007 WHO recommended VMMC as a key component of HIV prevention. In 2010 in South Africa, VMMC program was initiated(19). We include a low level of VMMC prior to 2007 to reflect low coverage of traditional circumcision(20,21). VMMC increases to 70% in 2030 and stays stable thereafter. The impact of VMMC is modelled as an average reduction in HIV susceptibility in the male population which is calculated as a reduction in transmission relative to the proportion of adult men who have been circumcised and efficacy of VMMC against HIV acquisition.

**HIV treatment:** HIV treatment increases survival in PLHIV and reduces infectivity of HIV. In 2004 a national ART program was initiated in South Africa, and prior to this ART was only made available through demonstration projects. We model the increase in ART coverage at population-level from 2004 onwards and assumed no ART coverage prior to this to reflect low coverage (22–24). ART scale up is parameterized to reflect UNAIDS estimates of ART coverage in women and men between 2010-2018, and to meet the 90-90-90 targets by 2030 for each sex, after which the coverage remains stable in the main analyses. As validation, we compared the age specific coverage estimated in the model and observed in data.

People can move between the ‘not on ART’ and ‘on ART’ compartments reflecting discontinuation of ART use. Rate of stopping ART varies by sex and age, and is higher for FSW than women who are not FSW. All age groups in the model are eligible for ART initiation. Efficacy of ART accounts for a proportion on ART being virally suppressed with efficacy of 100%, while non-virally suppressed persons have a reduced but non-zero infectivity. People on ART have a reduced mortality compared to PLHIV not on ART, but they remain at higher risk of death compared to HIV- individuals.

**4) Baseline interventions - for HPV and Cervical cancer**

**Cervical cancer screening:** Cervical cancer screening in South Africa has been offered in the form of cervical cytology smears as part of the national cervical cancer prevention program since 2000(25) but screening coverage remained low for a decade (median = 33% in 2013-2014) increasing to about 50% in recent years among eligible women above 30 years-old(26).

In the model basecase (in absence of counterfactual interventions), we model cervical screening programs as linear increase, so that prior to 2012, there is not screening, and between 2012 and 2017 screening increases from 20% of annual screening uptake among 25 years old, 25% uptake of screening among of 35 and 45 years old to uptake of 40%, 55% and 55%, respectively (27). After 2017, the screening coverage stays stable (unless it is increased as part of a modelled intervention scenario). We assumed similar uptake between women living with and without HIV, given limited differences in the proportion ever screened between HIV positive and negative women in 2012 SABBSM survey(28). We incorporated a low screening uptake in the younger ages (at age 24), reflecting the reported proportion of women younger than 35 who reported having ever had a PAP smear in their life (8.7% of 15-24 years old, and 34% of 25-34 years old) in the SABBS 2012 survey.

We also accounted for the treatment cascade following a screening based available data on reported practices in South Africa in the literature.(10,29–31) Probability of screening uptake multiplied by the screening cascade will result in the probability of successful clearing of the CIN2+. In the model this is operationalized with the parameter $\theta_{i}$(successful treatment given uptake of screening, detection, follow-up, and treatment success (the full cascade following screening uptake is outlined in table 3.1). We assume that the screening cascade depends on HPV disease stage (CIN2+ or CC), and on HIV status, but not on HPV types present.

People who are successfully treated can either clear infection (parameter *z* varied between 60-80%): (32)

A proportion of people who clear the infection will develop temporary, fully protective immunity against reinfection with the HPV grouped types they cleared. Women who clear the infection but do not develop protective immunity return to the susceptible compartment. Successful treatment of cervical cancer is incorporated in the form of hysterectomy(32). People who receive hysterectomy, and who are successfully treated are removed from the model.

In the counterfactual intervention scenarios (with increased screening), screening uptake targets were reached by 2030, and 2045, and these were modeled as linear increase between 2017-2030 and 2030-2045, so that the target was reached by the desired year. Screening uptake was increased if the intervention target was higher than the base case screening uptake, otherwise it was kept at basecase level. The intervention scenarios model an improved (more effective) treatment cascade (table 3.1). We assume that the improved treatment cascade was available for everyone who takes up CC screening (and not only those who are now screened more frequently compared to basecase).

**Table 3.1**. Treatment cascade for women with CIN2+ or CC following uptake of screening in the baseline model as described in the text above.

| **Cascade** | **Basecase scenario** | | | | | **Intervention scenarios** | |
| --- | --- | --- | --- | --- | --- | --- | --- |
|  | **CIN2+** | **CIN2+** | **CC** | **CC** | **References** |  |  |
|  | HIV- | HIV+ | HIV- | HIV+ |  |  |  |
| **Quality of test/adequacy** | 80% | 80% | 80% | 80% | (33) |  | CIN2+ and CC: 100% |
| **Probability of detecting lesion/Sensitivity** | 65% | 65% | 65% | 65% | (34–37) |  | CIN2+ 92%  CC 94% |
| **Follow-up for colposcopy / biopsy** | 72% | 72% | 90% | 90% | (29) |  | CIN2+ and CC: 90% |
| **Probability of diagnosis following colposcopy** | 90% | 90% | 90% | 90% | (10) |  | CIN2+ and CC: 100% |
| **Proportion treated** | 51% | 51% | 40% | 40% | (29,30) |  | CIN2+ 90%  CC 100% |
| **Treatment success** | 91% | 77% | 30-85%  (used 57.5%) | 30-85%  (used 57.5%) | (31) |  | CIN2+ and CC: 100% |
| **People who clear the infection as a result of successful treatment for CIN2+ (Uniform distribution)** | 0.6-0.8 | 0.6-0.8 | NA | NA | (32) |  | CIN2+ 0.6-0.8  CC NA |

**HPV vaccination:** Although South Africa has rolled-out a national vaccination programme in 2014, with consistent 2-dose coverage of around 60% among 9-year-old girls, our basecase assumption for this analysis is that there is no vaccination programme in place. This is to be consistent with previous analyses of the Cervical Cancer Elimination Modelling Consortium. We model HVP vaccination (with 9-valent vaccine) in the main scenarios (and bivalent vaccine in sensitivity analysis). We only introduce vaccination in 2020 in the intervention scenarios and no vaccination in the basecase scenario. Vaccination moves people from unvaccinated to vaccinated health states at per capita rates among the eligible population which is defined to achieve the desired vaccine coverage and relevant age groups. We assume lifelong 100% efficacy against all HR-HPV types in the 9-valent vaccine, and no cross-protection for nvt-HPV. Vaccine protection is assumed to be the same by HIV status. In the main scenarios, vaccine-induced immunity is lifelong but can also represent shorter duration of vaccine protection, as assumed in sensitivity analysis. Waning of vaccine immunity, is implemented by applying a constant rate to the vaccinated compartments. The inverse of the waning rate represents the average duration of vaccine protection. People losing their immunity move to HPV susceptible non-vaccinated health states and are fully susceptible to vt-HPV infections again. As part of sensitivity analysis, we also incorporated bivalent vaccine instead of 9-valent vaccine. In these scenarios, the vaccine only provided protection against HPV 16/18 and there was not cross-protection against the other grouped types.

**5) Model calibration**

We calibrated the model in 4 phases in Bayesian framework, that was operationalized in order:

1. Demography (by age, year)
2. HIV and sexual behaviors (by sex, age, year)
3. HPV and CIN2+ (by HIV, sex, age, year)
4. Cervical cancer incidence (by age)

For each phase, we varied the parameters governing the processes; for phase i) we varied the population growth and mortality, for phase ii) we varied parameters in tables 6.1 and 6.2, and for phase iii) we varied parameters from table 6.3 (with the exception of CC related parameters), and in phase iv) we only varied the CC related parameters.

For each phase we, draw a random sample from the prior distribution of the parameters varied in that phase, run the model with a combination of the parameters to be varied, and select runs that fit within the targets set for the phase (e.g. HIV prevalence targets by sex and age for phase 2). Once we have calibrated a phase, we use the model fits in the next phase. We sample from the joint posterior parameter distribution of the calibrated model at draw level (e.g. randomly select a simulation from the accepted runs, and us the parameters of the simulation), we then repeat the calibration sampling from the joint posterior distribution of the previous phases, and the random sample of the parameters varied in the current phase. The approach allows varying a smaller number of parameters at a time, while calibrating to multiple targets.

**6) Parameter estimates and fitting/validations outcomes and data sources**

Table 6.1. Ranges used for the sexual behavior parameters

| **Parameter** | **Symbol** | **Distribution** | **Min** | **Max** | | **Description, references** | |
| --- | --- | --- | --- | --- | --- | --- | --- |
| **Proportion of people in different sexual activity groups (i), by sex (s), constant over time** | | | | | | | |
| Men, lowest activity |  | Uniform | 0.77 | 0.86 | | Lowest activity = 1-medium activity – high activity | |
| Men, medium activity |  | Uniform | 0.07 | 0.11 | | Approximately 10% of the population (aged 15-34) reported 3 or more partners in the past year, which was defined as our cut-off for medium risk. SABBS 2012 (data-analysis of SABBS) (28) | |
| Men, FSW clients |  | Uniform | 0.07 | 0.12 | | There is uncertainty on the proportion of population who are FSW clients: upper end of self-reports ~10%. Self-reports likely biased downwards.(3,4,38) | |
| Women, lowest activity |  | Uniform | 0.91 | 0.965 | | 1-medium activity – high activity | |
| Women, medium activity |  | Uniform | 0.03 | 0.07 | | Approximately 5% of the population (aged 15-34) reported 3 or more partners in the past year SABBSM 2012 (data-analysis) (28) | |
| Women, FSW |  | Uniform | 0.005 | 0.02 | | Proportion estimated to be FSW (0.69-0.96% of the adult female population).  (4,38–40) | |
| **Partner change rates (PCR) per year.** | | | | | | | |
| **9-14 years old PCR per year for non-commercial partnerships** | | | | | | | |
| **Annual partner change rate** | $\boldsymbol{c}_{\boldsymbol{ra}}$ |  |  |  | | Varied by sex, activity level and age, constant over time, adjusted as part of balancing of partnerships | |
| Women, lowest activity |  | Uniform | 0 | 0.01 | | Between 5-7.6% of women reported having had sex before age 15 (Shisana 2017). In YRBS, 20-30% of <15 yos report ever having had sex.(41,42). We define the range of sexual activity in the lowest activity level from 0 to 0.01 per year to account for uncertainty in the proportion sexually active before age 15. | |
| Women, medium activity |  | Uniform | 0.1 | 0.42 | | Women in the 2 highest risk groups consist of ~6% of population, they are assigned a low level of sexual activity before age 15. In HPTN068 7.2% of 13-14 yos girls reported sexual partners in the past 12 months, and approximately 1.25 partners per sexually active person per year (ages 13-14). We define upper end as 1.25/2 to account for 9-10 years olds. (Pettifor 13-14 years old in HPTN068, personal communication) | |
| Women, highest activity, non-commercial |  | Uniform | 0.1 | 0.42 | | As above. | |
| Men, lowest activity |  | Uniform | 0 | 0.01 | | For 2002-2017, between 11.3-19.5% of men reported having had sex before age 15. In absence of further data for 9-14 years old boys, we assume the same priors as for girls. (41,42)﻿ | |
| Men, medium activity |  | Uniform | 0 | 0.6 | | Given uncertainty for the boys (and higher proportion reporting sexual activity), we assigned wider priors for medium-activity boys. | |
| Men, highest activity, non-commercial |  | Uniform | 0 | 0.6 | | Given uncertainty for the boys (and higher proportion reporting sexual activity), we assigned wider priors for medium-activity boys. | |
| **15-24 years old PCR per year for non-commercial partnerships:** We define PCR for the ages 25-34 (age that has the highest PCR in SABBSM 2012 data(28)), and the other age groups (15+) are defined relative to 25-34 using a risk ratio (RR) | | | | | | | |
| Women, lowest activity (RR) |  | Uniform | 0.56 | 0.58 | | RR to 25-34 low activity women. Ever had sex: 57.3% (54.3-60.3%), Average number of partners last year: 0.573 (0.542-0.603), (SABBSM 2012 data-analysis) | |
| Women, medium activity (RR) |  | Uniform | 1.25 | 1.35 | | RR to 25-34 medium activity women. Ever had sex 100%, average number of partners last year: 3.43 (2.61-4.24), which was higher than for women25-34. '' | |
| Women, highest activity (RR) |  | Uniform | 0.34 | 1 | | For FSW non-commercial partners, the data are often not age stratified: 5.6-29.2% of FSW reported casual partners in the last 6 months and they reported 1-9 (for range of IQR across cities) casual partners in the previous 6 months (SAHMS 2014). In the Port Elisabeth study, 12-19% of FSW had casual partners in the last year and they reported a mean of 1.38 (0.34-2.41) casual partners in the past year. (PEFSW 2015, Rao, Baral et al. 2016). We assume that women aged 25-34 have the highest PCR and 15-24 have lower or similar PCR. (43–45) | |
| Men, lowest activity (RR) |  | Uniform | 0.58 | 0.59 | | RR to 25-34 low activity men. Ever had sex: 49.3 (46.4-52.1), Average number of partners last year: 0.606 (0.563-0.649). (SABBSM 2012 data-analysis) | |
| Men, medium activity (RR) |  | Uniform | 0.64 | 0.7 | | RR to 25-34 medium activity men. Ever had sex 100%, average number of partners last year: 5.13 (4.1-6.16). (SABBSM 2012 data-analysis) | |
| Men, highest activity (RR) |  | Uniform | 0.64 | 0.7 | | Stone et al. study described a wide range of non-commercial partners for FSW clients with 36-76% of clients reporting casual partners with 1.1-15.1 partners per year resulting in clients having 0.4-11.5 casual partners.(4). In the Port Elisabeth client survey, 57.6% (95%CI: 53.4-61.7%) of clients reported having a casual female partner in the last 3 months. In absence of more accurate data, we have assumed that the clients have similar range of non-commercial partners to medium activity men including the age pattern. | |
| **25-34 years old PCR per year for non-commercial partnerships:** We define PCR for the ages 25-34 (age that has the highest PCR in SABBSM 2012 data(28)), and the other age groups (15+) are defined relative to 25-34 using RR | | | | | | | |
| Women, lowest activity |  | Uniform | 0.96 | 0.98 | | Ever had sex: 97.4 (96.3-98.5), Average number of partners last year: 0.971 (0.96-0.982). (SABBSM 2012 data-analysis) | |
| Women, medium activity |  | Uniform | 2.5 | 2.99 | | Ever had sex 100%, Average number of partners last year: 2.57 (2.14-2.99)  (SABBSM 2012 data-analysis) | |
| Women, highest activity |  | Uniform | 2 | 2.41 | | For FSW non-commercial partners, the data are often not age stratified: 5.6-29.2% of FSW reported casual partners in the last 6 months and they reported 1-9 (for range of IQR across cities) casual partners in the previous 6 months (SAHMS 2014). In the Port Elisabeth study, 12-19% of FSW had casual partners in the last year and they reported a mean of 1.38 (0.34-2.41) casual partners in the past year. (PEFSW 2015, Rao, Baral et al. 2016). We kept the prior range similar to women who are medium activity. | |
| Men, lowest activity |  | Uniform | 1.03 | 1.11 | | Ever had sex: 93.5 (91.4-95.6), Average number of partners last year: 1.07 (1.03-1.11)  (SABBSM 2012 data-analysis) | |
| Men, medium activity |  | Uniform | 4.56 | 9.66 | | Ever had sex 100%, Average number of partners last year: 7.11 (4.56-9.66)  (SABBSM 2012 data-analysis) | |
| Men, highest activity |  | Uniform | 4.56 | 9.66 | | Same partner number as for medium activity men | |
| **35-49 years old PCR per year for non-commercial partnerships:** We define PCR for the ages 25-34 (age that has the highest PCR in SABBSM 2012 data(28)), and the other age groups (15+) are defined relative to 25-34 using RR | | | | | | | |
| Women, lowest activity (RR) |  | Uniform | 0.97 | 1 | | RR to 25-39 low activity women. Ever had sex: 98.6 (97.5-99.7), Average number of partners last year: 0.984 (0.973-0.995). (SABBSM 2012 data-analysis) | |
| Women, medium activity (RR) |  | Uniform | 0.65 | 0.68 | | RR to 25-34 medium activity women. Ever had sex 100%, Average number of partner last year: 1.75 (1.39-2.11). (SABBSM 2012 data-analysis) | |
| Women, highest activity (RR) |  | Uniform | 0.34 | 1 | | RR to 25-34 highest activity women. Applied same RR priors for 35-49 years olds as for 15-24 years olds. | |
| Men, lowest activity (RR) |  | Uniform | 0.95 | 0.97 | | RR to 25-39 low activity men. Ever had sex: 96.4 (94.7-98.2), Average number of partners last year: 1.02 (0.995-1.05). (SABBSM 2012 data-analysis) | |
| Men, medium activity (RR) |  | Uniform | 0.44 | 0.47 | | RR to 25-34 medium activity men. Ever had sex 100%. Average number of partners last year: 3.27 (2.0-4.54). (SABBSM 2012 data-analysis) | |
| Men, highest activity, (RR) |  | Uniform | 0.44 | 0.47 | | RR to 25-34 highest activity men. Used the same prior ranges for non-commercial partners of clients. | |
| **50-74 years old PCR per year for non-commercial partnerships:** We define PCR for the ages 25-34 (age that has the highest PCR in SABBSM 2012 data(28)), and the other age groups (15+) are defined relative to 25-34 using RR. For people 50+ assumed their partner change rate is significantly reduced, and that ongoing partnerships are long term. | | | | | | | |
| Women, lowest activity (RR) |  | Uniform | 0 | 0.05 | | RR to 25-39 low activity women. Ever had sex: 97.3 (96.0-98.5), Average number of partners last year: 0.969 (0.955-0.982). (SABBSM 2012 data-analysis) | |
| Women, medium activity (RR) |  | Uniform | 0 | 0.1 | | RR to 25-34 medium activity women. Ever had sex 100%, Average number of partners last year: 1.27 (0.805-1.73). (SABBSM 2012 data-analysis) | |
| Women, highest activity (RR) |  | Uniform | 0 | 0.5 | | RR to 25-34 highest activity women. | |
| Men, lowest activity (RR) |  | Uniform | 0 | 0.05 | | RR to 25-39 low activity men. Ever had sex: 97.6 (96.2-99.0), Average number of partners last year: 1.01 (0.988-1.04). To account for long-term partners (crudely), the UL was divided by 4  (SABBSM 2012 data-analysis) | |
| Men, medium activity (RR) |  | Uniform | 0 | 0.1 | | RR to 25-34 medium activity men. Ever had sex 100%, Average number of partners last year: 3.16 (1.79-4.53). (SABBSM 2012 data-analysis) | |
| Men, highest activity, non-commercial (RR) |  | Uniform | 0 | 0.5 | | RR to 25-34 highest activity men. | |
| **Commercial partners (PCR per year) for FSW** | | | | | | | |
| FSW aged 15-24 |  | Uniform | 50 | 200 | | Wide range for clients reported with 100-1000 per year estimated in Stone et al. As there were more studies for FSW than for clients, the FSW partner numbers were used to determine the client partner numbers. We apply the same number of clients per year for 25-34, and 35-49 years old FSW. Most women enter sex work at age 21+ (SAHMS), and the client number is halved for 15-24 yos (more accurate could be 1/3 times OCR)  (43) | |
| FSW aged 25-34 |  | Uniform | 500 | 1000 | | As above | |
| FSW aged 35-49 |  | Uniform | 100 | 1000 | | As above | |
| FSW aged 50-74 |  | Uniform | 1 | 10 | | We assume FSW who are aged 50+ have substantially fewer clients. Data on FSW older than 50 is limited. | |
| **Commercial partners (PCR per year) for male clients of FSW** | | | | | | | |
| Number of commercial FSW partners clients have |  | Adjusted to FSW | NA | NA | | Large variation and fairly small partner numbers reported (from 2 py to up to 4 per 3 months). We used FSW data to determine the client partner change rate. (3,4,38) | |
| **Number of sexual acts per partnerships** | | | | | | | |
| **Acts in a partnerships** | $\boldsymbol{n}_{\boldsymbol{ra}}$ |  |  |  | |  | |
| Acts in a partnership, lowest activity |  | Uniform | 50 | 64.8 | | Frequency of sex in last 30 days among those with regular partners was (4.6-5.9) for male and (3.7-4.6) for female in the 2005 HSRC survey (Shisana, Rehle et al. 2005). In the Ekurhuleni study (Quaife, Eakle et al. 2016), males and females reported 3.8-5.7 and 3.5-5.1 sex acts in the previous month, respectively. Average is 3.9-5.4 per month. (3,46)  We sample from the priors for each age, activity, and sex category, and divide the acts by their partner change rates. This follows the assumption that the more partners someone has, the shorter the partnerships and/or the fewer acts per partnership.  For ages 9-14, the acts are divided by 2, despite the low partner change rate in this age group based on 13-14 yos sexually active girls reporting relatively few acts relative to number of partners (Pettifor 13-14 years old in HPTN068, personal communication) | |
| Acts in a partnership, medium activity |  | Uniform | 50 | 64.8 | | Assume the same prior for acts for HR partnerships. The number of acts are divided by the age specific partner change rates for non-commercial partnerships in HR for ages >15+ | |
| Acts in a partnership, highest activity, non-commercial partnerships |  | Uniform | 50 | 64.8 | | Assume the same prior for acts for HR partnerships. The number of acts are divided by the age specific partner change rates for FSW/client non-com for ages 15 and over, for the younger age group (9-14) the number is divided by 2 (assumption) | |
| Acts in a partnership – commercial partnerships |  | Uniform | 1 | 3 | | Assumption. | |
| **Vaginal vs anal intercourse (VI and AI)** | | | | | | | |
| **Proportion of sex acts that are AI by sexual activity group** | $\boldsymbol{p}_{\boldsymbol{r}}$ |  |  |  | |  | |
| Proportion of sex acts that are AI in low-activity people |  | Uniform | 0.001 | 0.009735 | | In Owen et al. 2017, pooled lifetime prevalence estimate for AI (any partner) among general-population was 19.3% (2.2-36.3%) while in the past 12 months prevalence is 5.4% (4.9-5.9%; 2 studies only). Proportion of acts among those practicing AI was 0.6-16.5% in the general population. We use the LL and UL of 12 month prevalence of AI * proportion of acts that are AI. (47) | |
| Proportion of sex acts that are AI for medium activity people (RR compared to low-activity) |  | Uniform | 1.1 | 1.3 | | Among higher-risk participants 23.2% (0.0-47.4%) reported ever having had AI The lifetime AI prevalence is 1-1.3 times higher than for the general population, and we applied this as an RR to estimate of the low activity people.(47) | |
| Proportion of sex acts that are AI for FSW, or clients with their non-commercial partners |  | Uniform | 0.001 | | 0.063 | | Of non-commercial partners of FSW,16.5% (11.4-21.6%) report having had AI in the past month (Owen 2019). We assume the same proportion of acts as for HR people (0.6-29.2%) (Owen 2017), and multiply the LL and UL of prevalence and proportion of acts (47,48) |
| Proportion of sex acts that are AI for FSW-client commercial relationships |  | Uniform | 0.024 | | 0.159 | | Owen (2017) found no large differences between FSW and the general population in South Africa for AI prevalence. Owen (2019) found that among FSW in the African region 15.1% (8.8–21.4) report ever having had AI, and 20.4% (10.1-31.8%) report AI in the past month. The percentage of all intercourse acts that were AI ranged from 2.4 to 15.9% in the six studies that reported it across the whole sample. Among those practicing AI 17% of acts were AI. Here, used the 2.4-15.9% over all acts as the most applicable measure. (47,48) |
| **Sexual mixing preference parameters by age and risk** | | | | | | | |
| **Age mixing of female index case with a male partner** | $\boldsymbol{\varepsilon}_{\boldsymbol{jm}}^{\boldsymbol{1}}$ |  |  | |  | |  |
| Preference of women aged 9-14 choosing a partner aged 15-24 |  | Uniform | 0 | | 0.20 | | Women can have partners with their own age group or from older age groups (see figure 1.1.). Age mixing data are defined based on proportion of age groups reporting partners 5 years older. We define the maximum possible proportion that could have partners from the next age group. For 9-14 yos, we use HPTN068 (13-14 yos data). For 15+, we used SABBSM 2005 data.(3) |
| Women 15-24 yos with preference for same partner aged 25-39 |  | Uniform | 0 | | 0.24 | | 24% report partners 5 years older. (3) |
| Women 25-34 yos with preference for partner aged 35-49 |  | Uniform | 0 | | 0.37 | | 37% report partners 5 years older. (3), |
| Women 35-49 yos with preference for partner 50-74 |  | Uniform | 0 | | 0.36 | | 36% report partners 5 years older.(3) |
| Women 50-74 yos with preference for same age partners |  | Fixed | 1 | | 1 | | Women can only have the same age partners in the oldest age group. |
| Women with preference for much older age groups |  | Uniform | 0 | | 0.02 | | The proportion of partnerships had with 2 age groups apart, assumed the same prior across age groups to reduce the number of parameters. |
| **Age mixing of male index case with a female partner** | $\boldsymbol{\varepsilon}_{\boldsymbol{mj}}^{\boldsymbol{1}}$ |  |  | |  | |  |
| Men 9-14 yos with preference for same age partners |  | Fixed | 1 | | 1 | | Men can have partners with their own age group or one age group younger.(3) |
| Men 15-24 yos with preference for partners aged 9-14 |  | Uniform | 0 | | 0.08 | | 8% report partners 5 years younger(3) |
| Men 25-34 yos with preference for partners aged 15-24 |  | Uniform | 0 | | 0.36 | | 32% report partners 5 years younger(3) |
| Men 35-49 yos with preference for partners aged 25-34 |  | Uniform | 0 | | 0.42 | | 42% report partners 5 years younger(3) |
| Men 50-74 yos with preference for partners aged 35-49 |  | Uniform | 0 | | 0.49 | | 49% report partners 5 years younger(3) |
| Men’s preference for age groups that are significantly younger |  | Uniform | 0 | | 0.02 | | The proportion of partnerships allowed age groups that are the furthest from your own age group (see figure 1.1 schematic). |
| **Sexual mixing by activity level** | $\boldsymbol{\varepsilon}^{\boldsymbol{2}}$ | Uniform | 0 | | 1 | | Mixing by activity level is operationalized within age group. Sexual mixing is varied from assortative to proportionate for non-commercial partnerships, see figure 1.1. |

Table 6.2. Epidemiological parameters for HIV

| **Parameter** | **Symbol** | **Distribution** | **Min** | **Max** | **Description, references** |
| --- | --- | --- | --- | --- | --- |
| **HIV transmission probability in absence of preventative measures depends on HPV status (h), sex of the PLHIV (s), stage of HIV (i), and act type (t); reduction in transmission probability by** | | | | | |
| **Transmission probability per act for HIV** | $\boldsymbol{\beta}_{\boldsymbol{h,s, i}}^{\boldsymbol{HIV}}$ |  |  |  |  |
| Baseline HIV transmission probability per vaginal intercourse (VI) sex act |  | Uniform | 0.00053 | 0.00097 | Meta-analysis of 6 studies in HIV asymptomatic stage, not stratified by sex. The asymptomatic stage is selected as the baseline as the rest are RR to this.(49) |
| RR: HIV transmission probability per sex act female vs male (VI) |  | Uniform | 1.5 | 2 | RR to baseline HIV VI (49) |
| Baseline HIV transmission probability per sex act (AI) female to male (insertive) |  | Uniform | 0.001 | 0.0026 | Meta-analysis of 3 studies, used 95% CI, mixture of pre-ART and early-ART; all MSM (50) |
| RR: HIV transmission probability female vs male (female receptive; AI) |  | Uniform | 10 | 22 | RR to baseline HIV (AI)  Meta-analysis of 4 studies, 95% confidence interval of 0.0055-0.0223, which is 10-22 times that of baseline VI probability. (50) |
| RRRR: infectivity in acute state vs baseline |  | Uniform | 10 | 19 | RR= 9.17 (95% CI 4.47-18.81), given the wide CI, we used the point estimate and upper limit.(49) |
| RR: Susceptibility if HPV |  | Uniform | 1 | 5 | RR per sex act. We used a wider range than in the meta-analysis as the RR is applied at act level (while the studies measure it at individual-level).(15) |
| **HIV introduction and health state transitions** | | | | | |
| Year HIV is introduced in the model |  | Fixed | 1985 |  | (51) |
| Number of initial HIV infections seeded in the model population |  | Uniform | 100 | 1000 | We fixed the year of HIV introduction but vary the initial population size with HIV. |
| Annual rate from acute phase to HIV infection without ART | $\boldsymbol{\xi}$ | Uniform | 3 | 8 | Duration in acute phase is estimated at 0.24 (95% CI 0.10-0.50) years. Estimate based on serodiscordant couples study in Rakai, Uganda.(52) |
| **Annual HIV death rate** | $\boldsymbol{\eta}_{\boldsymbol{i}}$ |  |  |  |  |
| Annual HIV related death rate among those not on ART |  | Uniform | 0.071 | 0.1 | Morghan 2002 estimated 9.8 years survival from seroconversion, allowed for longer survival ranges(53) |
| Annual HIV related deaths if on ART (RR to HIV deaths not on ART) |  | Uniform | 0.05 | 0.2 | In a study by HIV CASUAL collaboration(54)the mortality hazard ratio was 0.48 (95%CI: 0.41, 0.57) for ART initiation versus no initiation. In Mahou 2017 1/RR of 2-3 was used as a prior. (54–56) Given improved survival and increased proportion who are virally suppressed, we chose a lower bound. |
| **Perinatally infected children (9-14) are modeled as a proportion of model population entering the age category 9-14 as living with HIV.** | | | | | |
| Proportion of 9-14 years old entering the model as perinatally infected from epidemic start to 1997.5 |  | Fixed | 0 |  | No perinatally infected children 9-14 years of age prior to 1997.5 (9) |
| Proportion of 9-14 years old entering the model as perinatally infected between 1997.5-2004 |  | Uniform | 0.003 | 0.005 | HIV prevalence among 10-year-olds was less than 0.2% in 2000 due to low survival. Children enter the model in non-ART non-acute HIV state until 2004, after 2004, the perinatally infected children enter the model in ART state.(9) |
| Proportion of 9-14 years old entering the model as perinatally infected between 2004-2013 |  | Uniform | 0.02 | 0.03 | HIV prevalence among 10-year-olds was estimated at approximately 2.1% in 2008 in absence of PMTCT (Ferrand 2012). Johnson (2016) estimates a stable and high MtoCT rate until 2002-2005 after which it starts declining (a child born in 2003 would be 9 2012). We assume that up to 2013 children entering the model at 9 years would have 1.5-3% probability of being HIV infected.(9,57) |
| RR: Proportion of 9-14 years old entering the model as perinatally infected between 2013-2020 modeled as RR to 2004-2013 value |  | Uniform | 0.60 | 0.70 | HIV prevalence among 10-year-olds is estimated at approximately 3.3% in 2020 in absence of PMTCT. PMTCT coverage was 57% in 2007 (reducing MTCT probability to 2% from 35%). Ferrand (2012) estimates HIV related mortality will be halved in 5-year-olds by 2005 and in 16-year-olds by 2020 in presence of PMTCT at coverage levels corresponding to South Africa. Johnson (2016) estimated the rate of mother to child transmission to be 9.1% in 2011-12 and 5.2% in 2019-20. We modeled this as: between 2014-2020 children entering the model at 9 years would have 60-70% reduction in perinatal infection compared to value between 2004-2013. (9,57) |
| RR: Proportion of 9-14 years old entering the model as of perinatally from 2020 onwards modeled as RR to 2013-2020 estimate |  | Uniform | 0.6 | 0.7 | South Africa is aiming for universal PMTCT coverage. Johnson (2016) estimated the rate of mother to child transmission to be 5.2% in 2019-20 and remain relatively stable after in presence of declining background HIV incidence in the population. We allow for low level of perinatal HIV in the model with the assumption that mother to child transmission would not be fully eliminated. For simplicity, we assume a stable level of HIV-prevalence in 9-year-olds after 2020 implemented as a further 60-70% reduction in 2014-2020.(57). Stable thereafter. |
| **ART coverage is modeled as time-varying increase in initiation from 2004 to 2030, and stable coverage thereafter; ART coverage is a result of modeled initiation of ART and stopping ART use.** | | | | | |
| Efficacy of ART among those on ART |  | Uniform | 0.92 | 0.99 | Being on ART reduced transmission by 92% among serodiscordant couples (mixed sample regarding CD4 and viral load status). We set this as the LL. For UL, we take the weighted average of virally suppressed and those not virally suppressed. In South Africa, 87.5% of those on ART are virally suppressed (Among women 89.9% and among men 82.1%). (58,59) |
| **ART initiation** | $\boldsymbol{\tau}$ |  |  |  |  |
| Year ART use begins |  | Fixed | 2004 |  | No ART use before 2004 when national ART program was started (22–24) |
| Year ART use acceleration ends |  | Fixed | 2030 |  | Rate increase to stop after 2030 |
| Annual rate of initiation of ART, maximum initiation |  | Fixed | 0.9 |  | Linear increase in ART initiation from annual rate from 0 to 0.9, set so that the resulting coverage estimates reflect data. |
| **Rate of stopping ART** | $\boldsymbol{o}_{\boldsymbol{s}\mathbf{,}\boldsymbol{r}\mathbf{,}\boldsymbol{a}}$ |  |  |  |  |
| Annual rate of stopping ART in the general population women |  | Uniform | 0.14 | 0.16 | In Rosen, ART initiation within 3 months of study start at 10 months LTFU was 7.9%; LTFU per year is 11.6% assuming people started on average 1.5 months after study start; 95% CI 5.8-17.6%. Similar range found in a cohort study (Mberi 2015) following general population: 10.9 per 100 person-years (95%CI: 9.2-12.8).(60,61) |
| RR of stopping ART in the general population men (vs women) |  | Uniform | 1.5 | 2.2 | RR to women. Men have a lower ART coverage, and we reproduced the trend by assigning higher stopping rate for men. |
| Annual rate of stopping ART among FSW |  | Uniform | 0.20 | 0.35 | Among women offered early ART, 30.2% (22.7-38.6%) were LTFU in the first 12 months. This does not account for women who should be on ART based on guidelines and may be an overestimate of loss to follow-up (LTFU) in the total FSW population. Rate per year calculated from the 95% CI of 12 month proportion, and the range minimum is divided by 2 to allow for more uncertainty in the LTFU in FSW. (62) |
| RR of stopping ART in people aged 9-14, RR to 50-74 |  | Uniform | 1.8 | 2.1 | Independent of sex, to replicate age trends of ART coverage in the general population.  Reference category is 50-74 years old who have the highest ART coverage based on data. |
| RR of stopping ART in people aged 15-24, RR to 50-74 |  | Uniform | 2 | 3 | As above |
| RR of stopping ART in people aged 25-34, RR to 50-74 |  | Uniform | 1.1 | 1.8 | As above |
| RR of stopping ART in people aged 35-49, RR to 50-74 |  | Fixed | 1.1 | 1.1 | As above |
| RR of stopping ART in people aged 50-74 (reference) |  | Fixed | 1 | 1 | Reference category |
| **Voluntary medical male circumcision (VMMC) coverage increases from 2007 to 2030 and stable thereafter; VMMC coverage is modeled as an average reduction in HIV susceptibility among men.** | | | | | |
| Reduced susceptibility if circumcised |  | Uniform | 0.38 | 0.66 | Medical male circumcision reduces the acquisition of HIV by heterosexual men by between 38% and 66% based on Cochrane review.(18) |
| Year when VMMC coverage starts increasing |  | Fixed | 2007 |  | In 2007 WHO recommended VMMC as a key component of HIV prevention. In 2010 in South Africa, VMMC program was initiated. We assume VMMC scale up starts in 2007 in South Africa |
| Year when VMMC cvoerage stops increasing |  | Fixed | 2030 |  | Assume remains stable after 2017 at levels 55-70%. |
| Proportion of men with VMMC at before 2007 |  | Uniform | 0.16 | 0.32 | Male circumcision prevalence was 38.0% (32.4-42.0%) in 2002 (Shisana, Simbayi et al. 2002). We assume the lower bound as the upper bound of baseline level of circumcision (larger trials were done in late 2000s and here we assume these would not have impacted men's circumcision status prior to 2002).(20,21) |
| Proportion of men with VMMC by 2030 and thereafter |  | Fixed | 0.70 |  | Assume VMMC coverage reaches 70% by 2030 to reflect HIV prevention goals.(41) |
| **Condom use, condom use increases linearly between start and stop year, before and after condom use stays stable** | | | | | |
| **Efficacy of condom use** | $\boldsymbol{\iota}_{\boldsymbol{t}}$ |  |  |  | Depends on type of act (t) |
| Efficacy of condoms for HIV, VI |  | Uniform | 0.75 | 0.90 | Effectiveness is approximately 80.2% but could be as low as 35.4% and as high as 94.2%, we defined a range around 80.2% (16) |
| **Proportion using condoms** | $\boldsymbol{u}_{\boldsymbol{tra}}$ |  |  |  | Depends on type of act (t), sexual activity level (commercial, non-commercial), and age. Time-varying. |
| Year condom use starts increasing |  | Uniform | 1985 | 1998 | Condom use promotion programs started in the 1990s. |
| Year condom use stops increasing |  | Uniform | 2005 | 2010 | Condom use increase stops |
| Condom use coverage (proportion, per act), pre-increase, non-commercial, reference category (15-24 years) |  | Uniform | 0.01 | 0.10 | Probability of condom use per act for non-commercial partners is assumed to be the same by sex and age and activity. In 2000 condom use on average 15.1% (across all partner types). Condom use during AI was similar to that for VI. Among general-risk populations, the fraction of AI and VI acts that were unprotected was. 27.0–53.6% and 26.9–57.0%, respectively, and it was assume that the same condom use probability applied for VI and AI(47). We assume that condom use before 2000 was lower than 15% |
| Condom use coverage (proportion, per act), final, non-commercial, reference category (15-24 years |  | Uniform | 0.48 | 0.71 | Probability of condom use per act for non-commercial partners is assumed to be the same by sex and age, and AI, VI. For age-specific assumptions we use RR, and the reference group is 15-25 years old whose condom used at last sex: 51.9% (47.7-56.0%) for women and 66.6% (61.9-71.2%) for men, in 2005 (SABBSM 2005 data analysis), we used the lowest and highest uncertainty limits to define ranges. |
| Condom use coverage (proportion, per act), pre-increase, commercial |  | Uniform | 0.05 | 0.15 | Probability of condom use pe act in commercial FSW-client interactions individuals (same by sex and age). Condom use of FSW with main partners was very low in 2000 (5-15%) (RHRU 2000/01), with another survey having 66% of FSW reporting not using a condom with the last non-paying partner in 2001/02 (Peltzer, Seoka et al. 2004). RHRU references from (4). |
| Condom use coverage (proportion, per act),, final. Commercial, VI |  | Uniform | 0.5 | 0.86 | Probability of condom use pe act in commercial FSW-client interactions individuals (same by sex and age). Clients report 64.7% (38.3-85.8%) and FSW 38.5% ( 20.2-59.4%) condom use with the last client in Shisana 2008. Condom use in FSW-client relationships is assumed to not vary by age. (4,44,45) |
| RR: Condom use, coverage for AI (RR to VI) |  | Uniform | 0.5 | 1 | Pooled estimates of the prevalence of unprotected AI among those reporting AI were higher than unprotected VI among those reporting VI although 95% CIs overlap. We use RR of 0.5-0.8 to capture the potentially lower usage of condoms for AI (47,48) |
| RR of condom use by age group, age 9-14 vs 15-24 |  | Uniform | 0.5 | 0.8 | Based on HPTN068 there were approximately 66 VI acts in the past 3 months of which 39 were reported protect (56%). (Pettifor 13-14 years old in HPTN068, personal communication). In absence of further data, we assume condom use to be lower among 9–14-year-olds compared to 15-24 year-olds. |
| RR of condom use by age group, age 15-24 |  | Fixed | 1 |  | In SABBSM 2005, highest condom use (at last sex) is reported among 15–24-year-olds, which defined our reference. We apply RR based on the LL and UL of reported estimates to reflect the condom use trends by age. |
| RR of condom use by age group, age 25-34 vs 15-24 |  | Uniform | 0.60 | 1 | SABBSM 2005, condom use 34.1% (30.1-38.1%) for women, and 45.7% (37.3-54.1%) for men. In SABBSM 2012 Women 38.8% (34.7-42.8), Men 45.5% (41.0-50.0). LL informed by 2005 and UL 2012. |
| RR of condom use by age group, age 35-49 vs 15-24 |  | Uniform | 0.40 | 1 | SABBSM 2005, condom use 20.2% (17.4-23.0%) for women 23.0% (18.9-27.1%) for men. SABBSM 2012, Women 26.7% (23.9-29.6), Men 28.4% (24.3-32.6). LL informed by 2005 UL 2012  0.56 |
| RR of condom use by age group, age 50-74 vs 15-24 |  | Uniform | 0.15 | 1 | SABBSM 2005, condom use 2.6% (1.45-3.75%) for women 7.27% (4.99-9.55%) for men. In SABBSM 2012, Women 9.56% (6.97-12.2), Men 15.1% (11.6-18.6). LL informed by 2005 UL 2012. |

Table 6.3. Epidemiological parameters for HPV

| **Parameter** | **Symbol** | **Distribution** | **Min** | **Max** | **Description, references** |
| --- | --- | --- | --- | --- | --- |
| **HPV transmission, transmission depends on HPV type (h), and HIV (i) (see section on interactions)** | | | | | |
| **Per person HPV transmission probability** | $\boldsymbol{\beta}_{\boldsymbol{h,i}}^{\boldsymbol{HPV}}$ |  |  |  |  |
| Per person HPV transmission probability for HPV 16/18 |  | Uniform | 0.3 | 0.8 | In Van der Valde varied from 0.05-1. Median 0.5 (IQR 0.3-0.8), used this as a per person transmission probability (10) |
| RR- HPV transmission probability for HPV 31/33/45/52/58 vs HPV 16/18 |  | Uniform | 0.5 | 1 | RR to HPV 16/18. (10) |
| Per person HPV transmission probability (nvtHPV) (RR to HPV 16/18) |  | Uniform | 0.5 | 1 | RR to HPV 16/18. (10) |
| **HPV clearance, depends on HPV type (h), HIV (i) (see section on interactions) and sex (s)** | | | | | |
| **Annual clearance rate for HPV (pre-CIN2+ stages)** | $\boldsymbol{\sigma}_{\boldsymbol{h,i,s}}$ |  |  |  |  |
| Annual clearance rate for HPV 16/18 |  | Uniform | 0.7 | 3 | Based on model posterior estimates estimated in Van de Velde (10,11) |
| RR: Clearance rate for HPV 31/33/45/52/58 vs HPV 16/18 |  | Uniform | 0.2 | 1 | RR to 16/18 clearance. |
| RR: Clearance rate for nvt HR-HPV vs HPV 16/18 |  | Uniform | 0.2 | 1 | RR to 16/18 clearance. |
| RR: Clearance rate for men vs women’s rate |  | Uniform | 0.6 | 1 | Slower clearance in men.(10) |
| **HPV natural immunity, depends on HPV type (h) and HIV (i) (see section on interactions)** | | | | | |
| Proportion who develop immunity when clearing infection? | m | Uniform | 0.4 | 0.8 | Unknown, uninformative prior - in Van de Velde posterior estimates broad with median ~0.5 and IQR ~0.3-0.8 (for lifelong natural immunity) (10,63) |
| **Annual rate of waning natural immunity for HPV infection** | $\boldsymbol{\delta}_{\boldsymbol{h,i,s}}$ |  |  |  |  |
| Annual rate of waning natural immunity HPV- 16/18 |  | Uniform | 0 | 0.2 | Unknown. 1 year to lifetime immunity (10,63) |
| RR: Waning natural immunity for HPV 31/33/45/52/58 vs HPV 16/18) |  | Uniform | 0.8 | 1.2 | Unknown. RR to HPV 16/18 immunity, assumed similar across HPV types. (10,63) |
| Waning natural immunity - nvtHPV (RR to HPV 16/18) |  | Uniform | 0.80 | 1.20 | Unknown. RR to HPV 16/18 immunity, assumed similar across HPV types. (10,63) |
| **CIN2+ health state transitions for women, progression depends on progression HPV type (h) and HIV (i), regression depends on HPV type (h), HIV (i), and age (a). (For HIV, see section on interactions)** | | | | | |
| **Annual rate of progression to CIN2+** | $\boldsymbol{\psi}_{\boldsymbol{h,i,s}}$ |  |  |  |  |
| Annual rate of progression to CIN2+ HPV 16/18 |  | Uniform | 0.05 | 0.20 | In Campos progression to CIN2+ between 0.003 and 0.02 per month for HPV 16 and HPV18 depending on time from infection. In HPV Advise from infected to CIN1 0.25-1.33, CIN1 to CIN2 0.07-3.84, and from CIN2 to CIN3 0.43-4.27 per year for HPV 16. Here from infected/CIN1 to CIN2+, we take half of upper bound of 0.07-3.84. (10–12) |
| RR: Progression to CIN2+ (31/33/45/52/58) (RR to HPV 16/18) |  | Uniform | 0.10 | 0.50 | RR to HPV 16/18. Progression tends to be slower. As in Campos , Van de Velde and others. In HPV Advise the relative rate is between 0.5-1.5 (cross-protective types) and 0.25-1 for other HPV types. |
| RR: Progression to CIN2+ (nvtHPV) (RR to HPV 16/18) |  | Uniform | 0.05 | 0.20 | '' |
| **Annual regression rate from CIN2+** | $\boldsymbol{\omega}_{\boldsymbol{h,i}\mathbf{,s,}\boldsymbol{a}}$ |  |  |  |  |
| Annual regression rate from CIN2+, HPV 16/18 |  | Uniform | 0.08 | 1.24 | RR to HPV 16/18. In Campos regression of CIN3 is 0.00435 per month and for CIN2 0.0087 per month. We take the average of these two as the lower bound. In HPV Advise, regression from CIN1 is 0=3.62 and CIN2 to CIN1 0-2.48 per year for HPV 16. We take half of CIN2 to CIN1 upper bound as the upper limit.(10,12) |
| RR: Regression from CIN2+ for HPV-31/33/45/52/58 vs HPV 16/18 |  | Uniform | 1.50 | 2.50 | RR to HPV 16/18. In Campos regression is the same across HPV types. In HPV Advise, relative regression is 1-2 for other HPV HPV. Here allow for higher upper bound due to grouped type behaving differently to individual types (10,12) |
| RR: Regression from CIN2+ for nvtHPV vs HPV 16/18 |  | Uniform | 1.50 | 3.00 | '' |
| RR: Regression among 25-34 years old, 1/RR to baseline regression |  | Uniform | 1.00 | 1.50 | Given the simplified natural history structure, we model age-dependent cervical cancer development by reducing regression in older women. This approximates the persistent infection in a subset of women |
| Regression among 35-49 years, 1/RR to baseline regression |  | Uniform | 10.00 | 15.00 | ’’ |
| Regression among 60+ yos, 1/RR to baseline regression |  | Uniform | 50.00 | 75.00 | ‘’ |
| Proportion of people who clear their infection during natural regression from CIN2+/CIN3 | q | Uniform | 0.6 | 0.8 | Assumption |
| Proportion of people who are successfully treated as a result of CC screening and who clear their infection during regression from CIN2+/CIN3 | z | Uniform | 0.6 | 0.8 | Assumption |
| Screening and treatment |  |  |  |  | See section on CC screening cascade |
| **Cervical cancer development in women (s) depends on age (a)** | | | | | |
| **Annual rate of CC development** | $\boldsymbol{\pi}_{\boldsymbol{s,a}}$ |  |  |  |  |
| Annual rate of CC development all types (baseline) |  | Uniform | 0.006 | 0.01 | Same development probability assumed in Campos across HPV types (0.000012-0.00742 pm). In HPV Advise progression from CIN3 to CC1 0.03-0.07. (10,12) |
| RR: Rate of CC development in individuals aged <25 years (RR to baseline progression) |  | Fixed | 1/10 |  | Lower development risk from CIN2+ to cervical cancer in younger ages. |
| RR: Rate of CC development in older individuals 50+ yos (RR to baseline) |  | Uniform | 10 | 20 | Transition towards cervical cancer is set to vary by age. This approximates the longer duration with pre-cancer in older ages (12) |
| RR:Rate of CC development in older individuals 60+ yos (RR to baseline) |  | Uniform | 30 | 45 | ‘’ |
| **Annual rate of CC death across all types** | $\boldsymbol{\upsilon}_{\boldsymbol{s}}$ | Uniform | 0.03 | 0.05 | In Campos, invasive cancer mortality is between 0.0016-0.03 per month depending on CC stage and length of illness. In HPV Advise, mortality rates vary between 0.018-0.354 depending on CC stage. These are the same rate estimates. We use the range as a prior. (10,12). Varied by age as described below. |
| **HPV-HIV interactions.** Same interactions applied across HPV types | | | | | |
| RR susceptibility if HIV (any HPV) |  | Uniform | 1 | 2.6 | Based on meta-analyses. HPV transmission probability is capped at 1.  Any HPV 1.60 (95%CI 1.33, 1.93)  HR HPV 2.36 (95%CI 2.11, 2.64) (15) |
| RR: Reduced clearance of HPV in PLHIV vs HIV- |  | Uniform | 0.4 | 0.7 | Same reduction for all HPV types  Any 0.53 (95%CI 0.42, 0.67)  HR 0.63 (95%CI 0.55, 0.74) (15) |
| RR: rate of waning immunity if HIV+ vs HIV- |  | Uniform | 1.5 | 2.0 | Unknown, for some HPV types evidence that re-infection risk is higher for HIV+ than HIV- people. (64) HIV assumed to influence natural immunity. RR among HIV+ vs HIV-, so that immunity wanes faster among PLHIV. |
| RR: Regression from CIN2+ if HIV+, RR to HIV- |  | Uniform | 0.3 | 0.6 | Slower regression among HIV+ observed compared to HIV-  (13,14) |
| RR: Progression to CIN2+ if HIV positive |  | Uniform | 1 | 3 | Incidence of CIN is higher in HIV+(14) |
| ART efficacy in reducing the HPV cofactor effects, after 2010. |  | Uniform | 0.1 | 0.5 | Impact of ART on HPV (susceptibility, clearance, waning of immunity, progression and regression). Assumed to operate in the same quantity when on ART to reduce the impact of HIV on HPV (susceptibility, clearance, waning of immunity).(13,63) |
| ART efficacy in reducing the HPV cofactor effects, before 2010. |  | Uniform | 0.1 | 0.3 | Reduction in ART efficacy on reducing HPV cofactor effect prior to 2010 is lower to approximate duration on ART.(13,63) |

Table 6.4. Outcomes used for model fitting and validation

| Outcomes | Years used in the calibration /validation | Sources |
| --- | --- | --- |
| Demography | | |
| Total Population size aged 9-74  Calibration  Validation | 1990, 2005, 2019  1950-2100 | (1)  (1) |
| Age distribution: ages 9-14, 15-24, 25-34, 35-49, 50-74  Calibration  Validation | 1990, 2005, 2019  2100 | (1)  (1) |
| HIV epidemiology | | |
| HIV prevalence  By sex and by age  Calibration  Validation | 2012  1990-2017 | (2,3,65)  (66,67) |
| HIV prevalence among FSW aged 15 and older  Validation | 1996-2015 | (68–73) |
| ART coverage by sex, aged over 15  Validation | 2010-2017 | (66) |
| ART coverage by age (both sexes combined)  Validation | 2017 | (67) |
| HPV/CC epidemiology | | |
| Any HR-HPV by age and HIV status in women (prevalence of HPV regardless of stage of disease)  Calibration  Validation | 2001  1998-2015 | (74)  (75–88) |
| Any HR-HPV by age and HIV status in men  Validation | 2008 | (75) |
| HPV-16/18 prevalence by age and HIV status in women (prevalence of HPV regardless of stage of disease)  Validation | 1998-2015 | (76–78,83,85,87,89–94) |
| HPV-16/18 prevalence by age and HIV status in men (prevalence of HPV regardless of stage of disease)  Validation | 2003-2012 | (95–97) |
| Nonavalent HPV prevalence by sex and age (prevalence of HPV regardless of stage of disease)  Validation | 2008 | (75,98) |
| CIN2+ prevalence in women:  By age and HIV status  Calibration | 2001 | (99,100) |
| CC infection incidence by age in women  Calibration  CC infection incidence, overall rate in women  Validation | 2018  2002-2018 | (103)  (101,102) |
| Total number of reported CC diagnoses in women  Validation | 2002-2010 | (104) |
| Type distribution in cervical cancer  Proportion of CC 16/18  Proportion of CC nvt HR-HPV | 2018  2018 | (105)  (105) |

**7) Description of analysis**

We estimated age-standardised cervical cancer incidence rate between 2020-2120, assuming that South Africa achieves UNAIDS 90-90-90 targets by 2030, and male circumcision increases to 70% by 2030, using the simulations from the 27 posterior parameter sets identified at the calibration stage to provide estimates for the basecase and the different intervention scenarios where vaccination and cervical screening is scaled up from 2020 onward. The different interventions scenarios are listed in Table 1 of the main technical appendix.

For the counterfactual scenarios, we simulated vaccination by scaling-up of vaccine coverage from 0 to target level among 9-14 years old during the first year of intervention (2020), from thereon the vaccine coverage is kept at target level in the age groups targeted by the intervention. In the counterfactual intervention scenarios (with increased screening), screening uptake targets were reached by 2030, and 2045, and these were modeled as linear increase between 2017-2030 and 2030-2045, so that the target was reached by the desired year. Screening uptake was increased if the intervention target was higher than the base case screening uptake, otherwise it was kept at basecase level. The intervention scenarios model an improved (more effective) treatment cascade (table 3.1). We assume that the improved treatment cascade was available for everyone who takes up CC screening (and not only those who are now screened more frequently compared to basecase). When an age standardised result was calculated, we age-standardised results at draw level and compute the median across age-standardised model estimates.

**8) Fitting and validation results**

The following figures shows the results of the model fits compared to data used for fitting the model at the calibration stage. And additional data not used to fit the model but to validate model predictions from the basecase scenario. Results show the full range of predicted model outcomes across the 27 parameter sets (i.e. 100% range) in gray and the median of model predictions. Data are shown in red. Black vertical lines represent the prior calibration range informed by the different data sources.

8.1) Demography


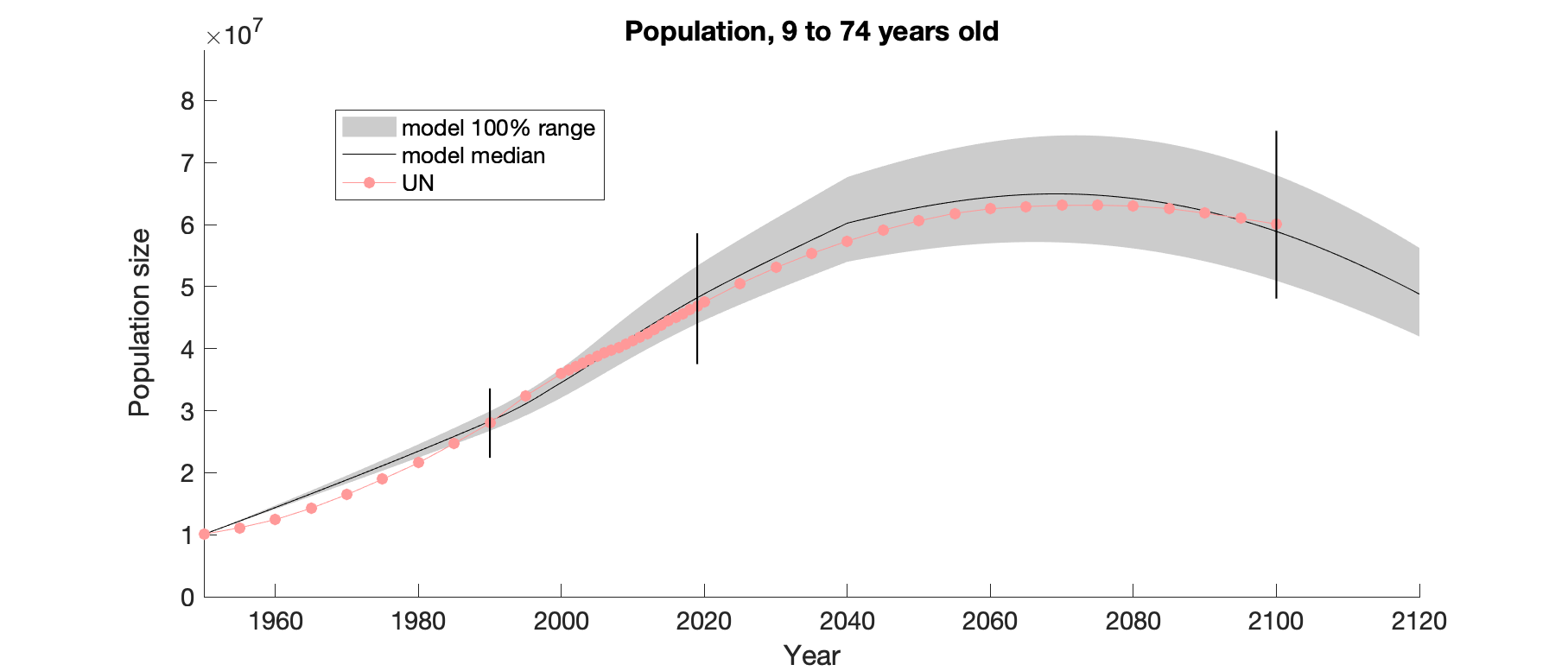


Figure 8.1 A) Modeled size of the total population over time compared to UN data (1)


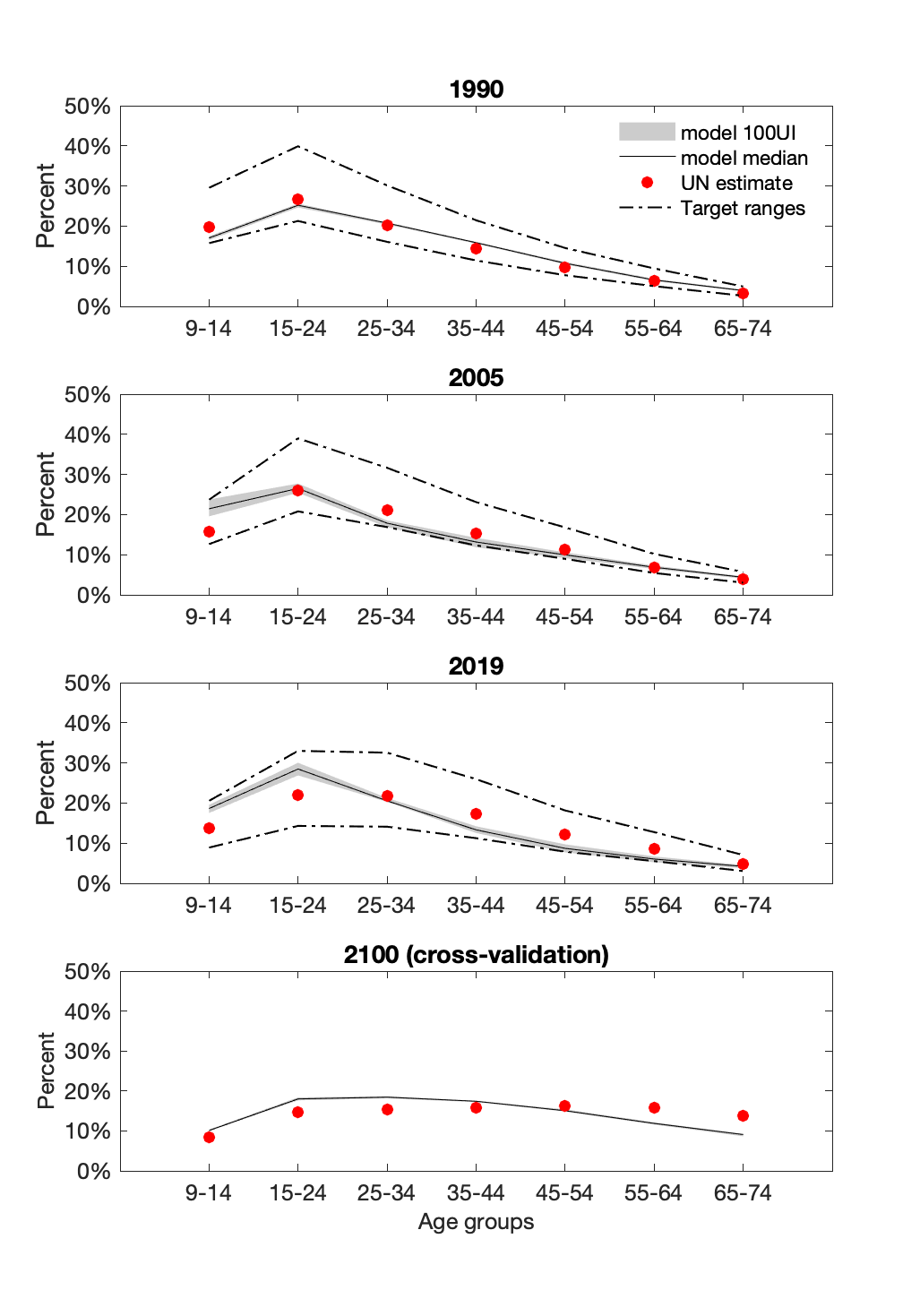


Figure 8.1B) Predicted Age distribution of the total population at different time compared to UN population estimates (1) Target ranges are shown in dashed lines. Last estimate for predicted population distribution in 2100 was used for cross-validation.

8.2) HIV epidemiology


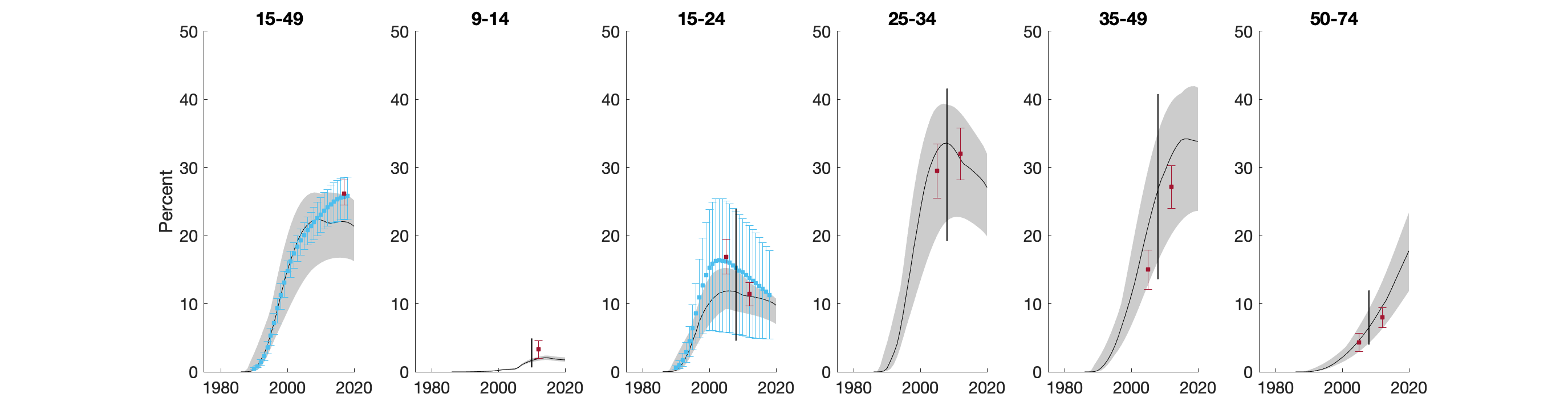
HIV prevalence

Figure 8.2 A) Modeled HIV prevalence in women, by age for1985-2020. Presented as the 100% model uncertainty range (grey) and median (black line). Model is calibrated by age (9-15, 15-25, 25-34, 35-49, 50-74) with the vertical black bars showing the calibration target (year 2012). Modeled estimates are compared against the overall adult aged 15-49 data. Calibration data used was SABBSM(3,65) in red, and model estimates were validated against UNAIDS estimates (aged 15-49, and 15-24)(24) **in blue.**


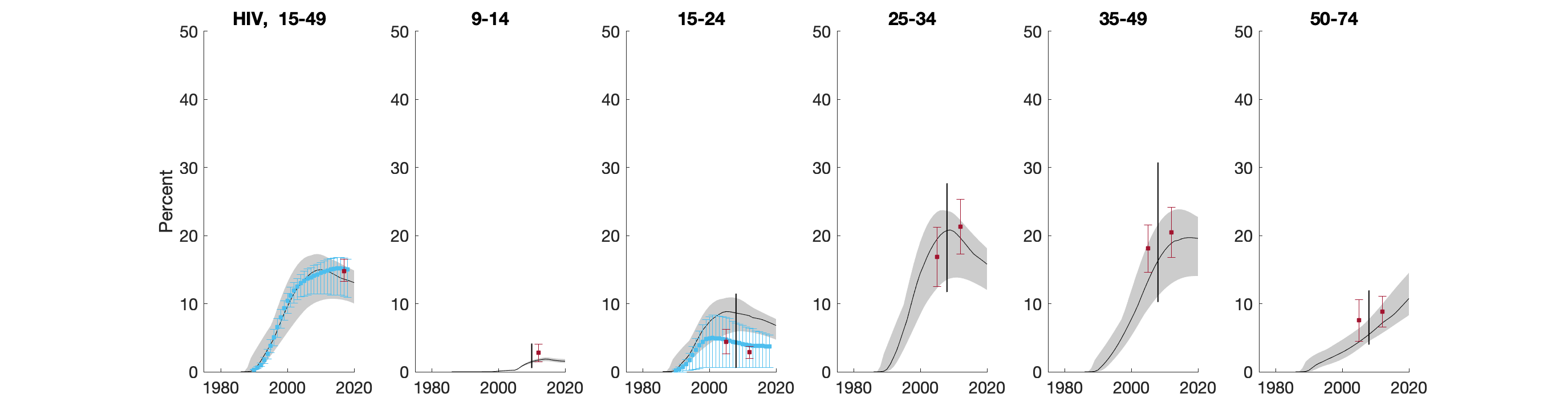


Figure 8.2 B) Modeled HIV prevalence in men, by age for1985-2020. Presented as the 100% model uncertainty range (grey) and median (black line). Model is calibrated by age (9-15, 15-25, 25-34, 35-49, 50-74) with the vertical black bars showing the calibration target (year 2012). Modeled estimates are compared against the overall adult aged 15-49 data. Calibration data used was SABBSM **in red** (3,65), and model estimates were validated against UNAIDS estimates **in blue** (aged 15-49, and 15-24)(24).


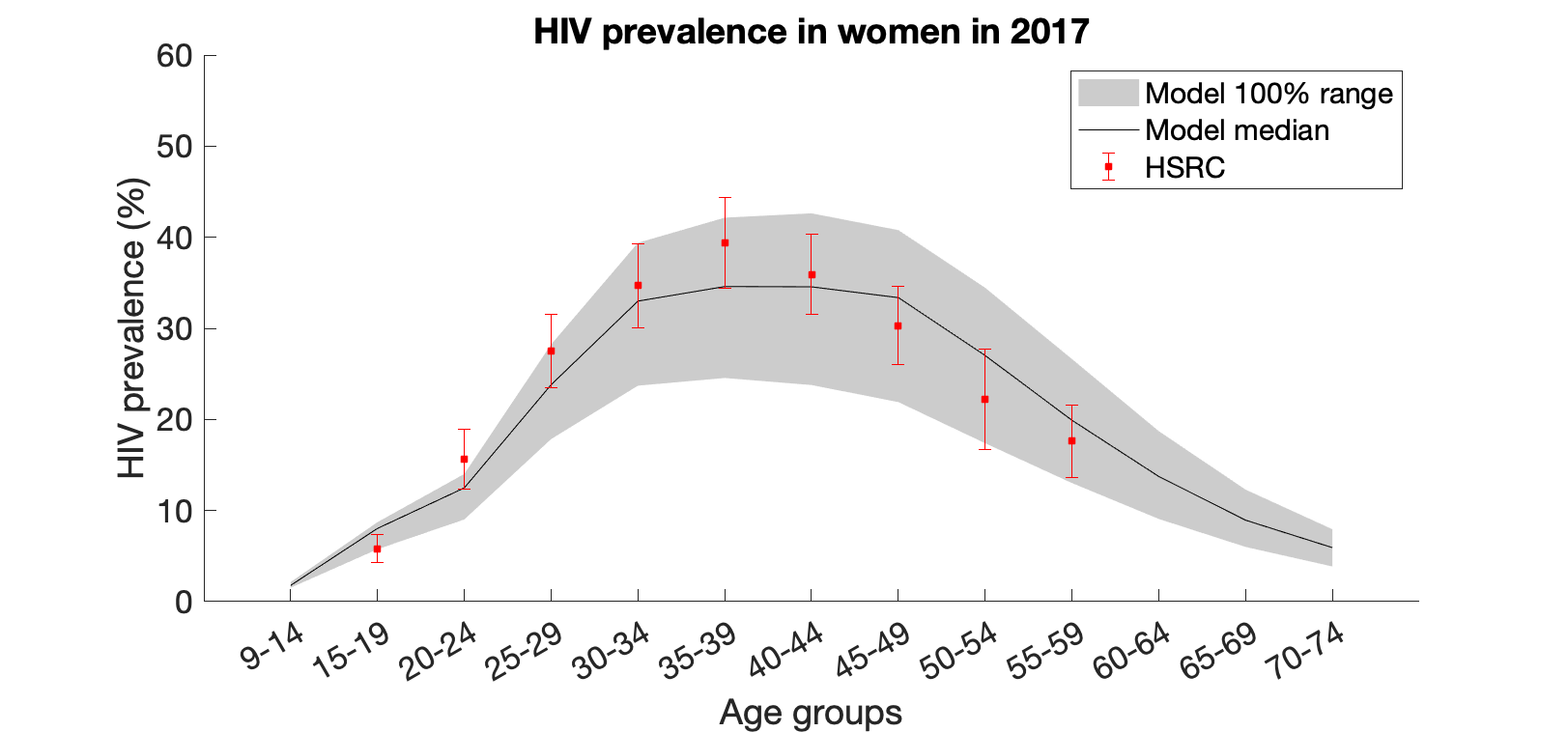


Figure 8.2 C) Modeled HIV prevalence in women by age compared to data in 2017.(67) Used for model validation.


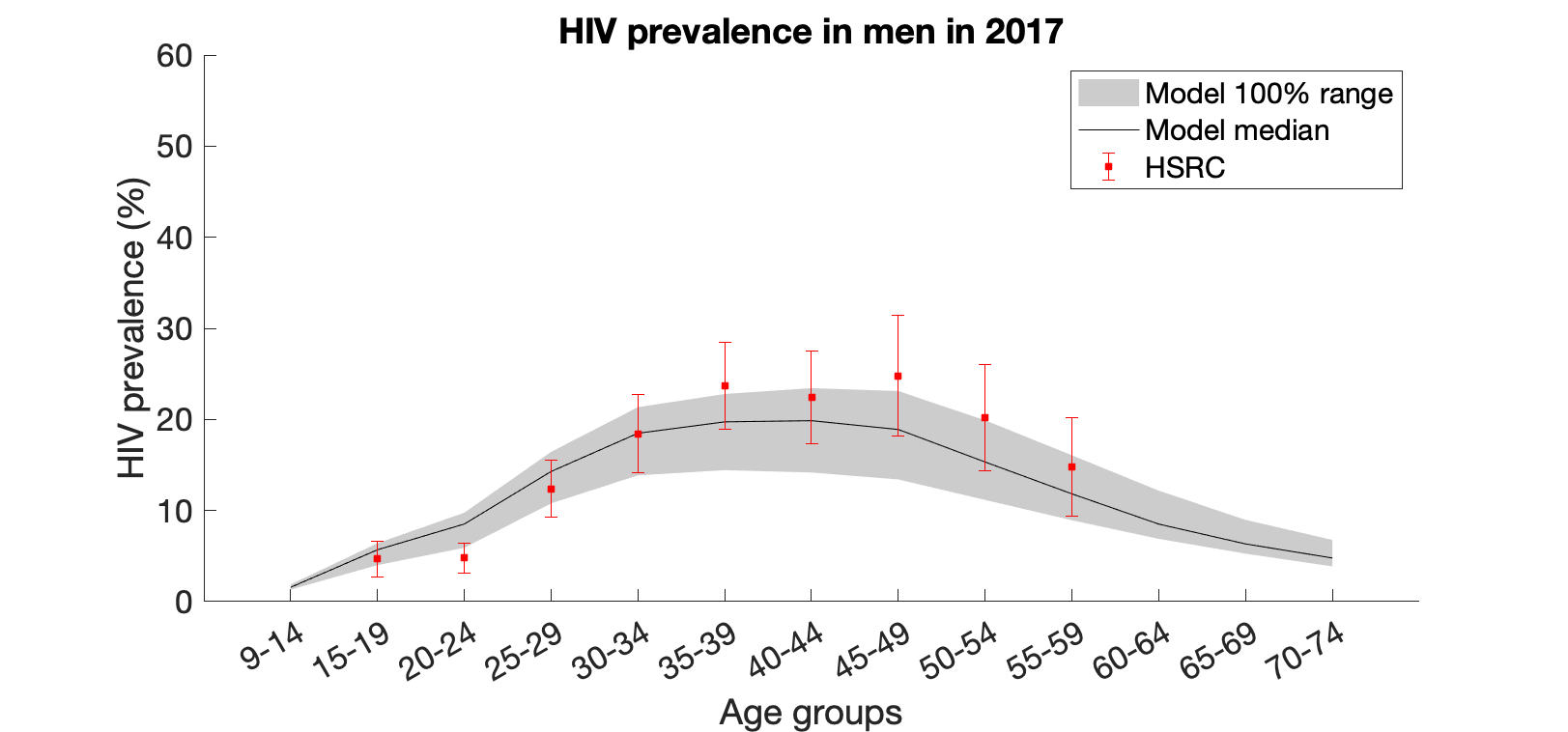


Figure 8.2 D) Modeled HIV prevalence in men by age compared to data in 2017.(67) Used for model validation.


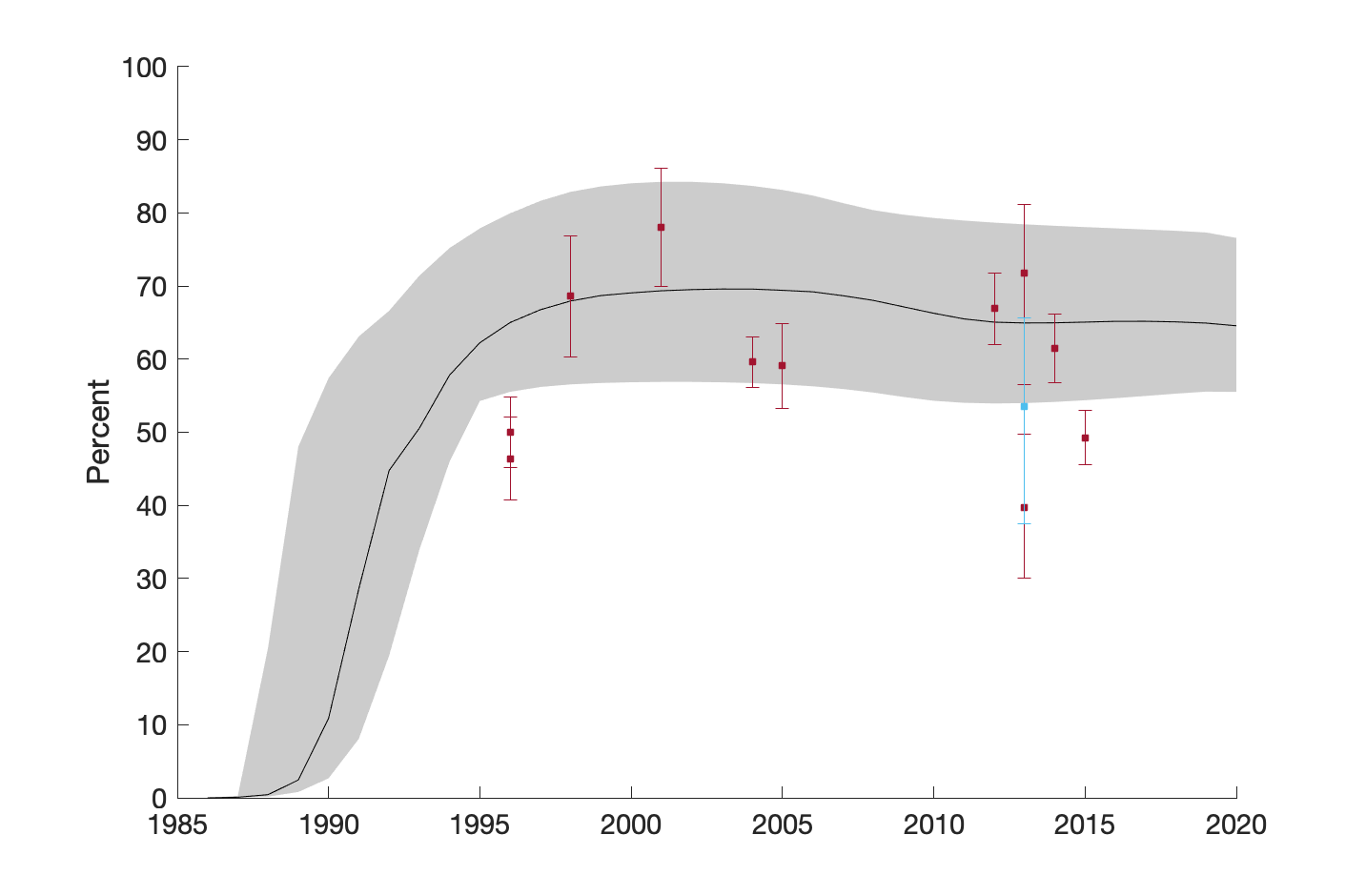


Figure 8.2 E) Modeled HIV prevalence among FSW who are aged 15 and older. Presented as the 100% model uncertainty range (grey) and median (black line). Validation against data. (68–73)

ART coverage


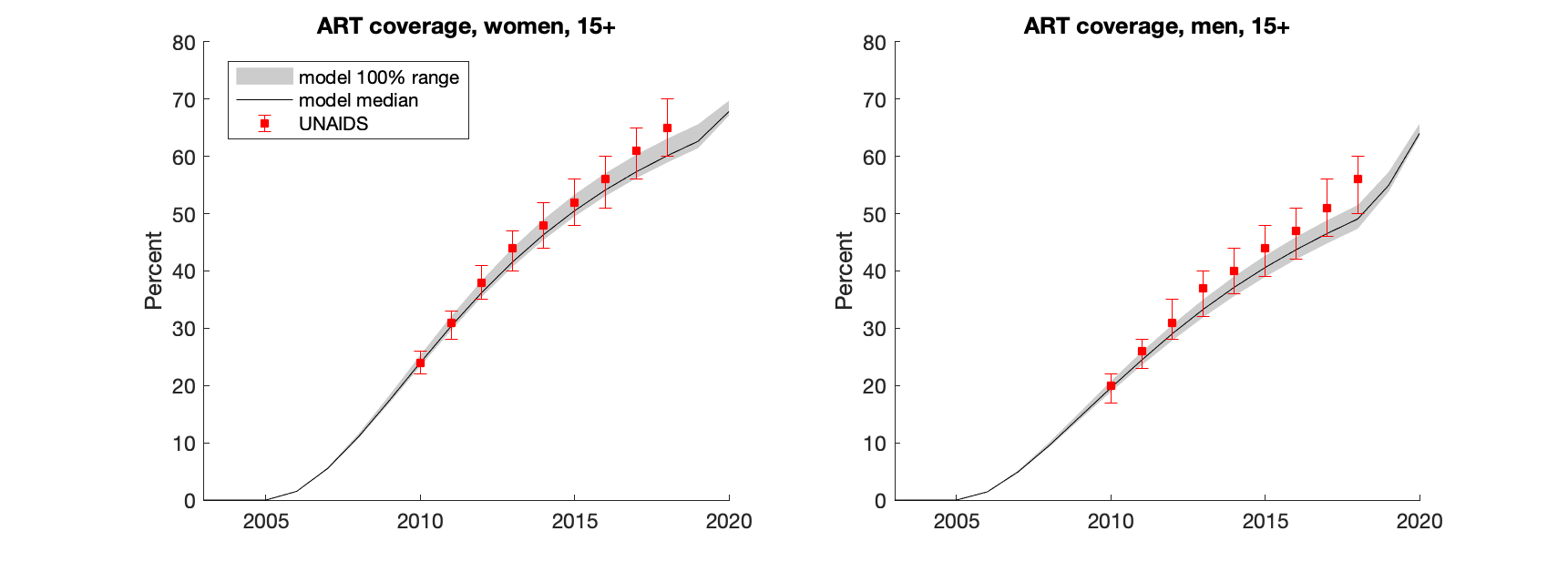


Figure 8.2 F) Modeled ART coverage among people living with HIV, over time by sex. Model input parameters set so that the desired increase in ART coverage is achieved, and the outputs are validated against UNAIDS estimates (66).


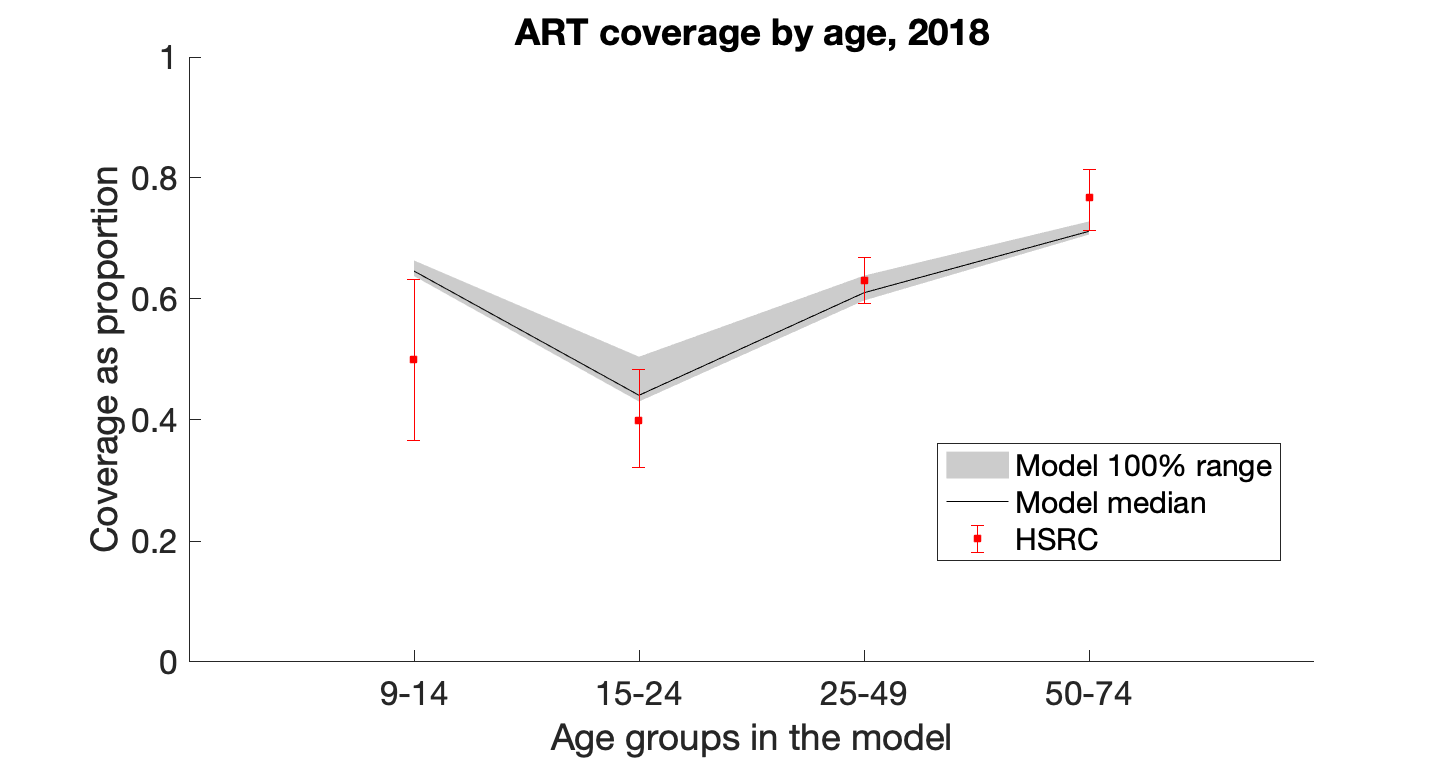


Figure 8.2 G) Modeled ART coverage among people living with HIV, both sexes combined. Model input parameters set so that the desired increase in ART coverage by age is achieved, and the outputs are validated against HSRC 2017 estimates(67).

N.B. The first age group in the HSRC is 0-14 years old, which is compared against 9-14 model output.

8.3) HPV epidemiology

HPV prevalence

Main calibration and validation figures


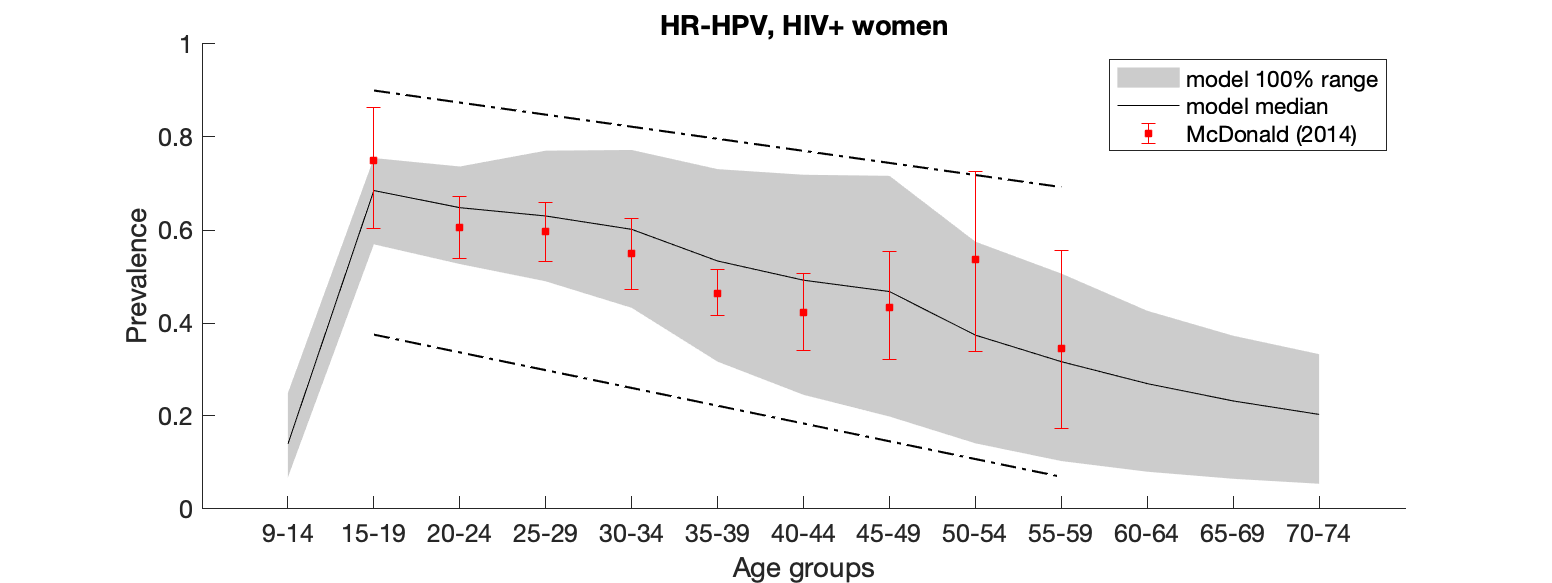


Figure 8.3 A) Modeled HR-HPV prevalence in women living with HIV by age in 2001(74) calibration. Dashed lines represent calibration target ranges.

N.B. First age group in McDonald is 17-19 years old, and we allowed the lower minimum prevalence range for 15-19 age group to reflect 15-16 years potentially having a lower prevalence.


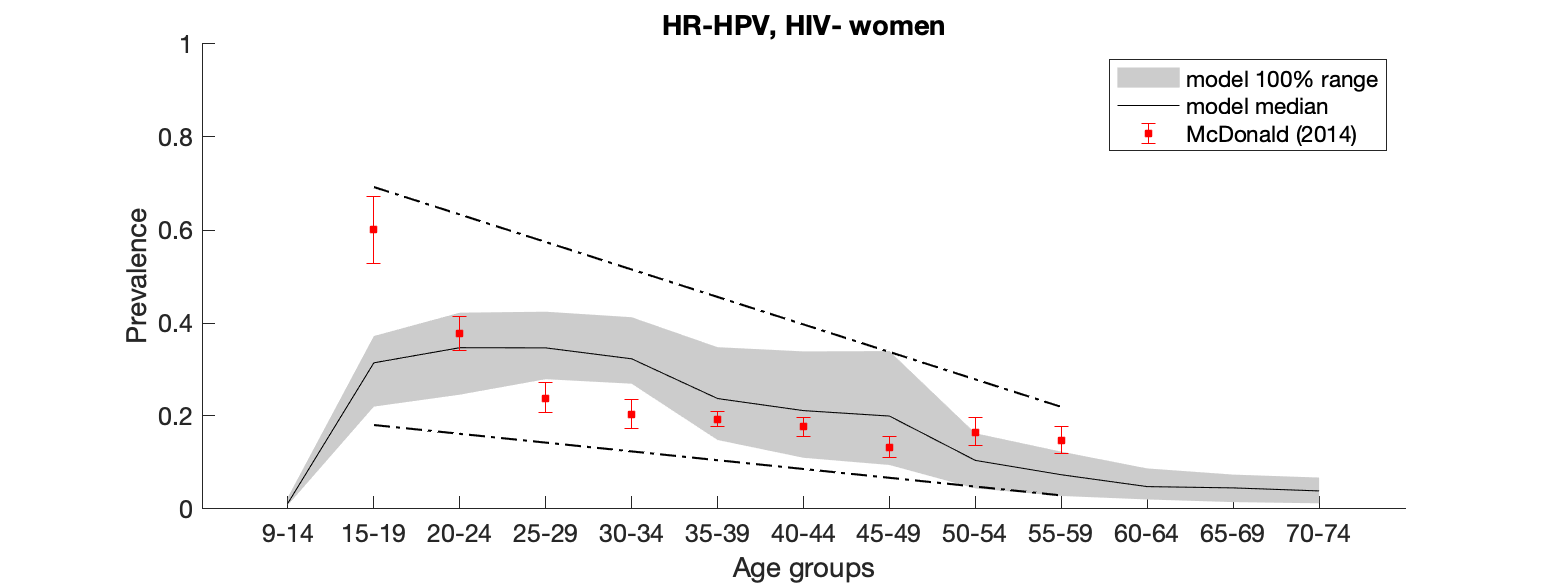


Figure 8.3 B) Modeled HR-HPV prevalence in HIV-negative women by age in 2001(74) calibration. Dashed lines represent calibration target ranges.

N.B. First age group in McDonald is 17-19 years old, and we allowed the lower minimum prevalence range for 15-19 age group to reflect 15-16 years potentially having a lower prevalence.


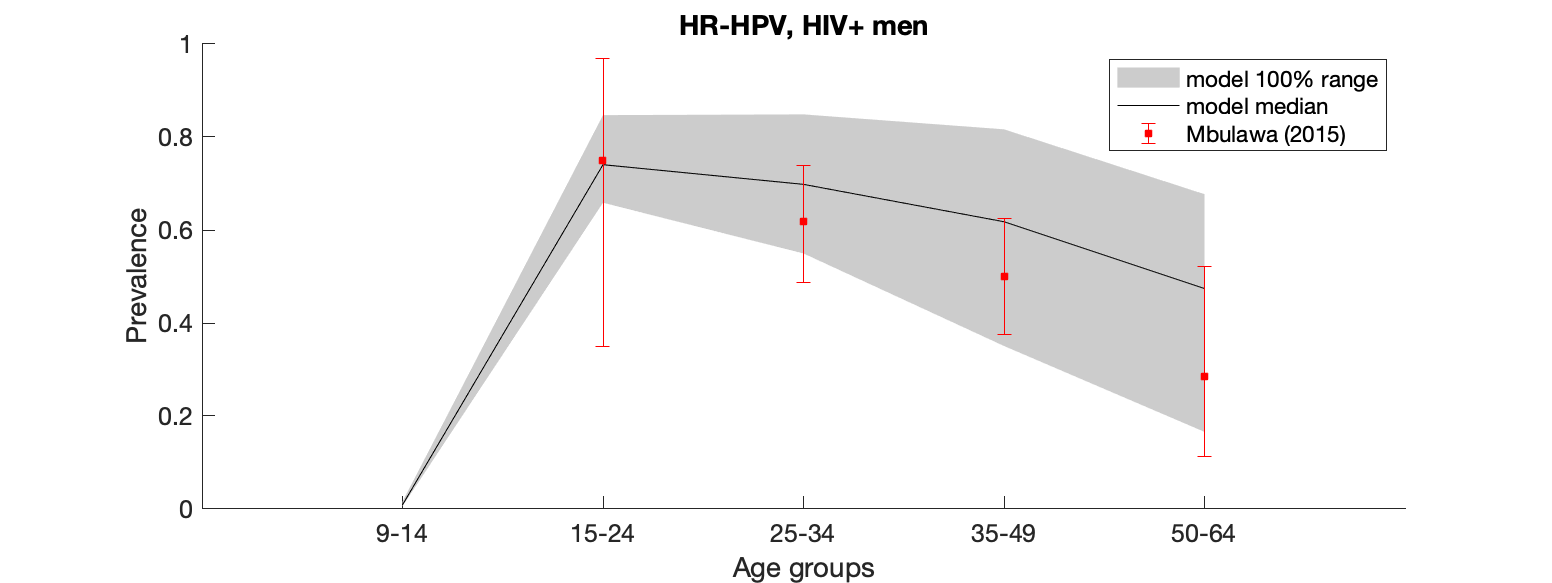


Figure 8.3 C) Modeled HR-HPV prevalence in men living with HIV by age in 2008. Model outputs validated against data (75).

N.B. Mbulawa (2015) is from sexually active population only, used for validation.


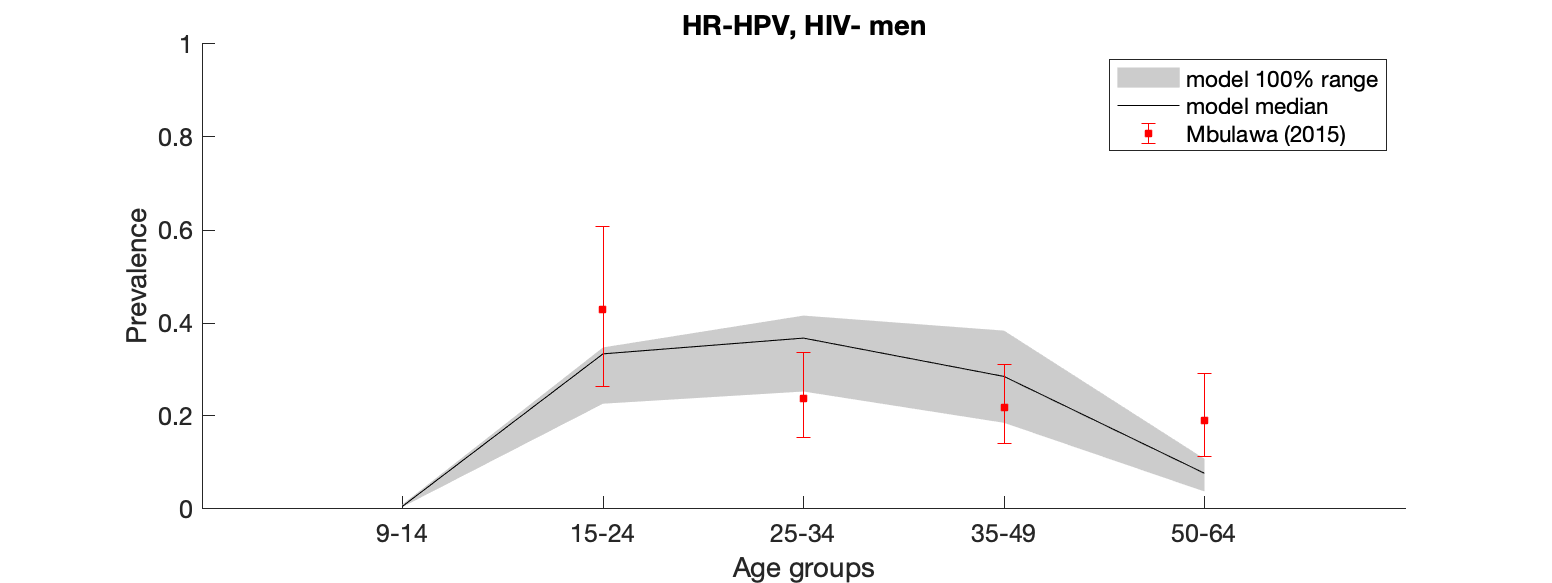


Figure 8.3 D) Modeled HR-HPV prevalence in HIV-negative by age in 2008. Model outputs validated against data (75).

N.B. Mbulawa (2015) is from sexually active population only, used for validation.


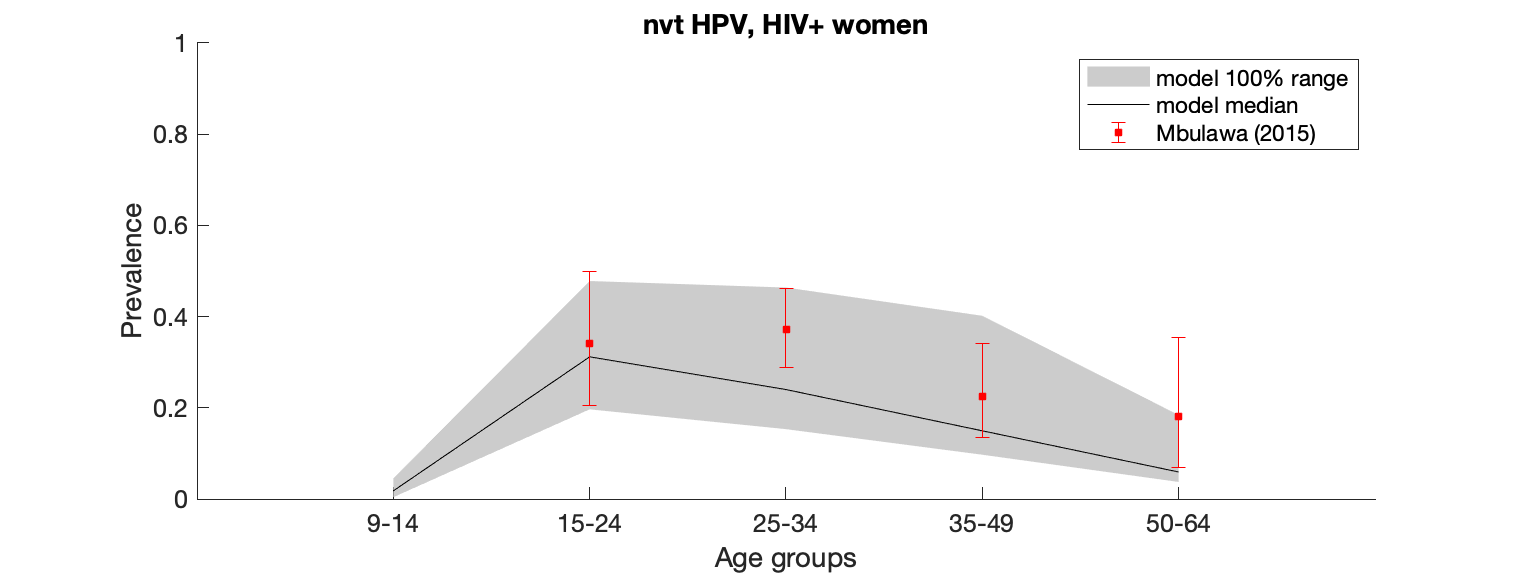


Figure 8.3 E) Modeled nvt-HPV prevalence in women living with HIV by age in 2008. Model outputs validated against data (75).

N.B. Mbulawa (2015) is from sexually active population only, used for validation.


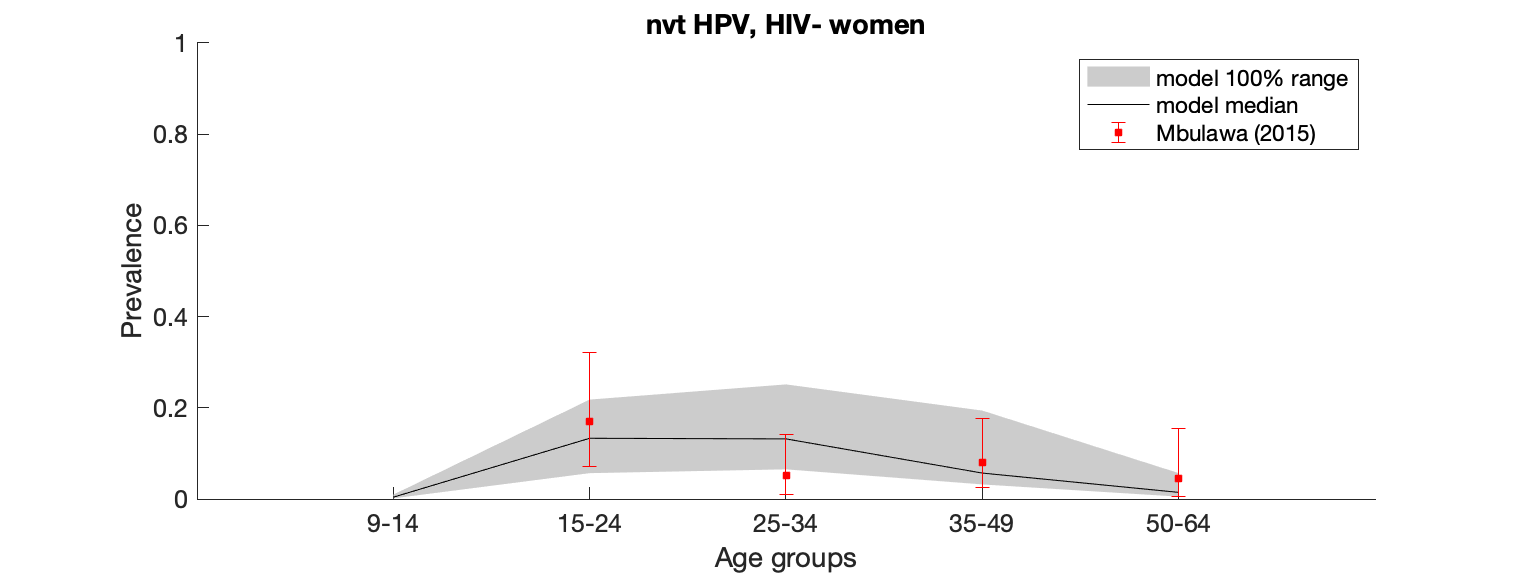


Figure 8.3 F) Modeled HR-HPV prevalence in HIV-negative women by age in 2008. Model outputs validated against data (75).

N.B. Mbulawa (2015) is from sexually active population only, used for validation.


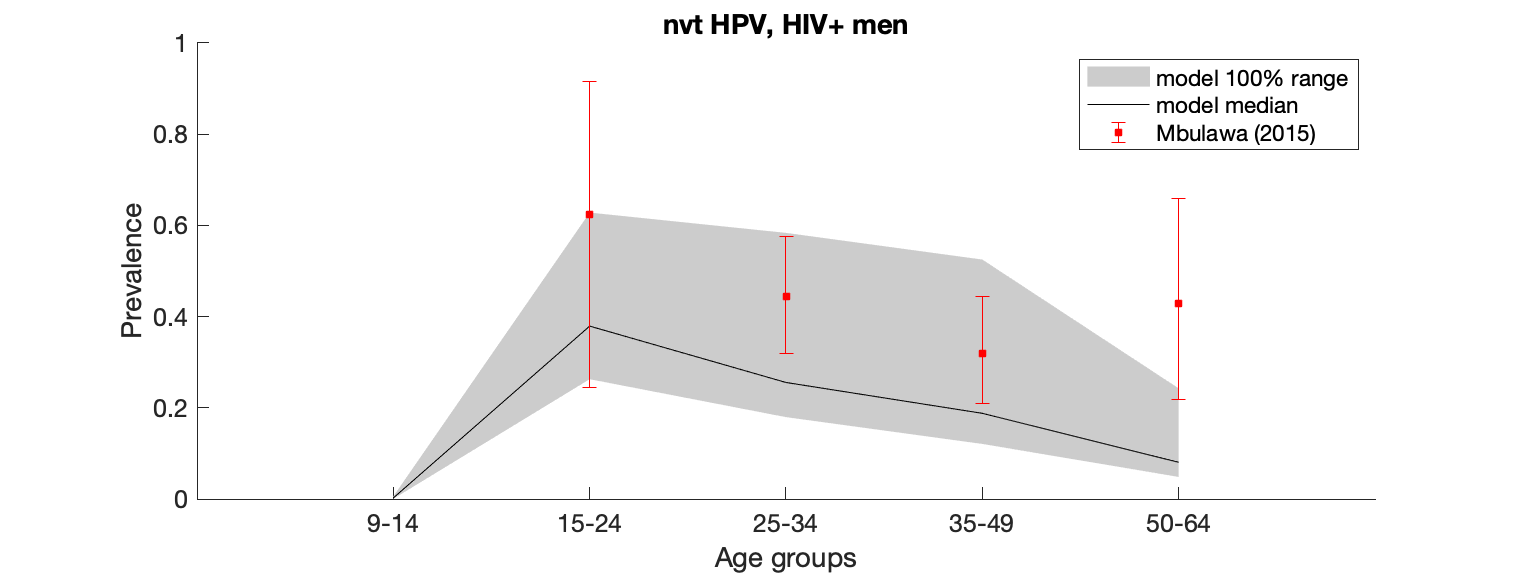


Figure 8.3 G) Modeled nvt-HPV prevalence in men living with HIV by age in 2008. Model outputs validated against data (75).

N.B. Mbulawa (2015) is from sexually active population only, used for validation.


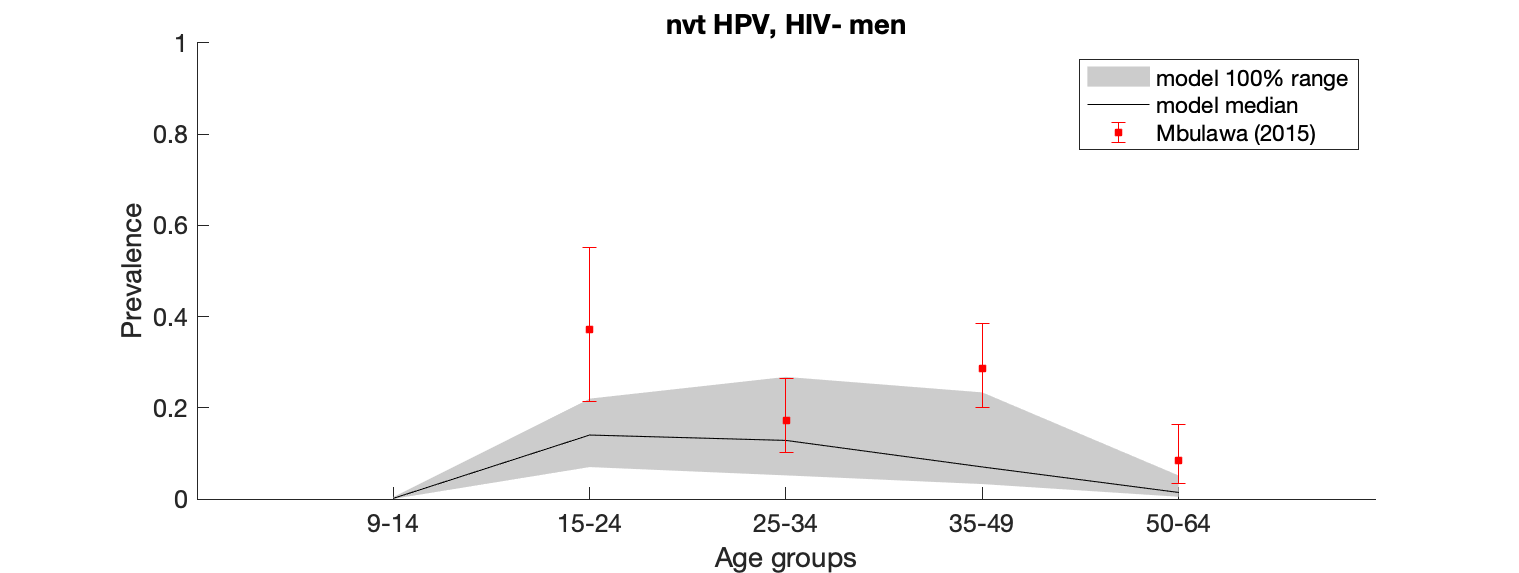


Figure 8.3 H) Modeled HR-HPV prevalence in HIV-negative men by age in 2008. Model outputs validated against data (75).

N.B. Mbulawa (2015) is from sexually active population only, used for validation

Additional validation figures which show HPV prevalence over time using mixed sample data.


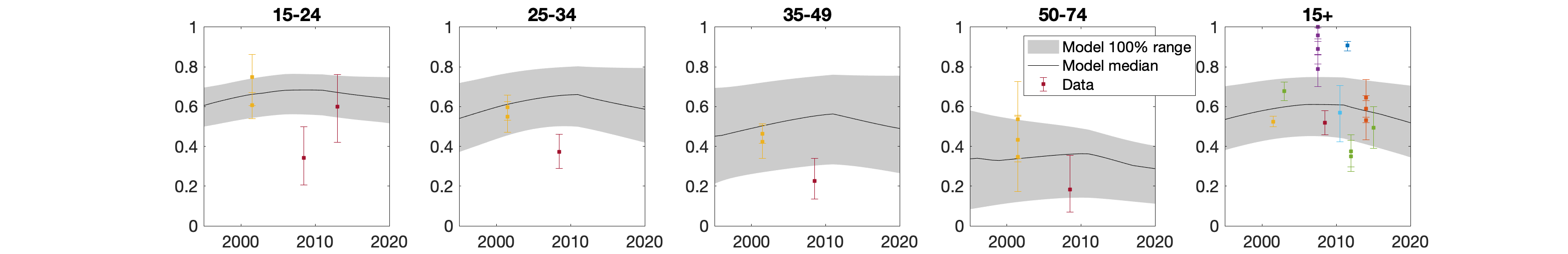


Figure 8.3 I) Modeled HR-HPV prevalence in women living with HIV over time. Model outputs validated against varied data. (75,77–81,88)


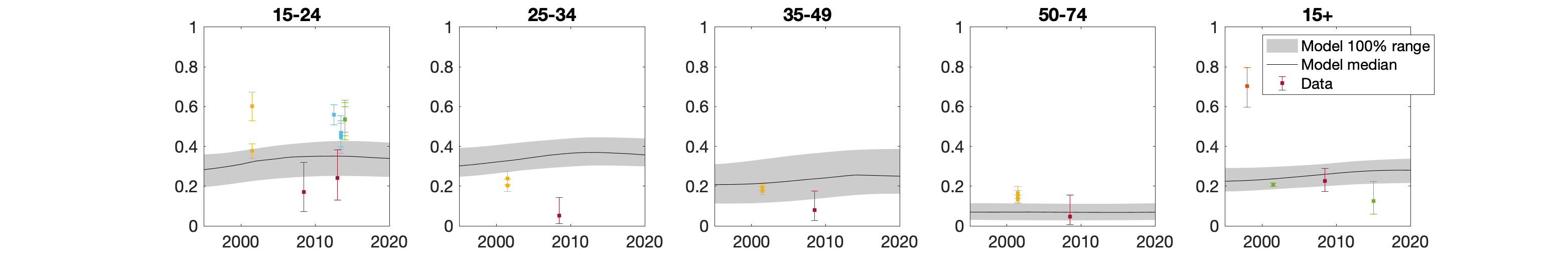


Figure 8.3 J) Modeled HR-HPV prevalence in HIV-negative women over time. Model outputs validated against varied data. (75,76,81–87)


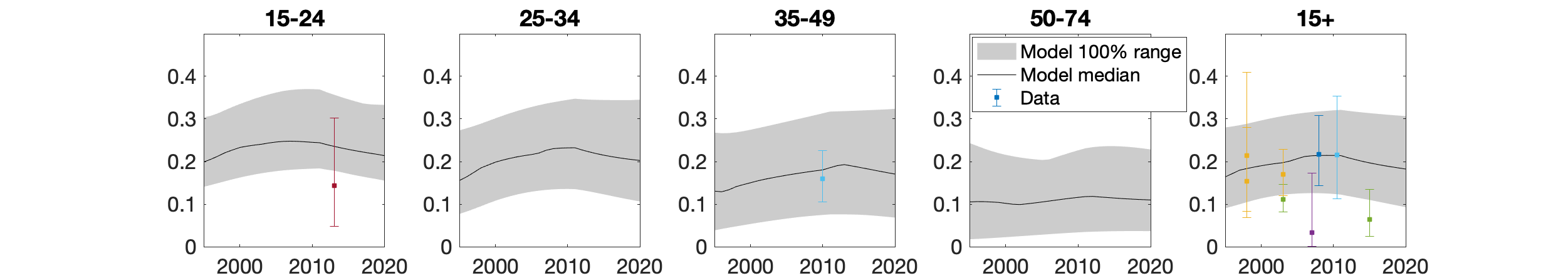


Figure 8.3 K) Modeled HPV 16/18 prevalence in women living with HIV over time. Model outputs validated against varied data. (77,78,85,87,89,91–94)


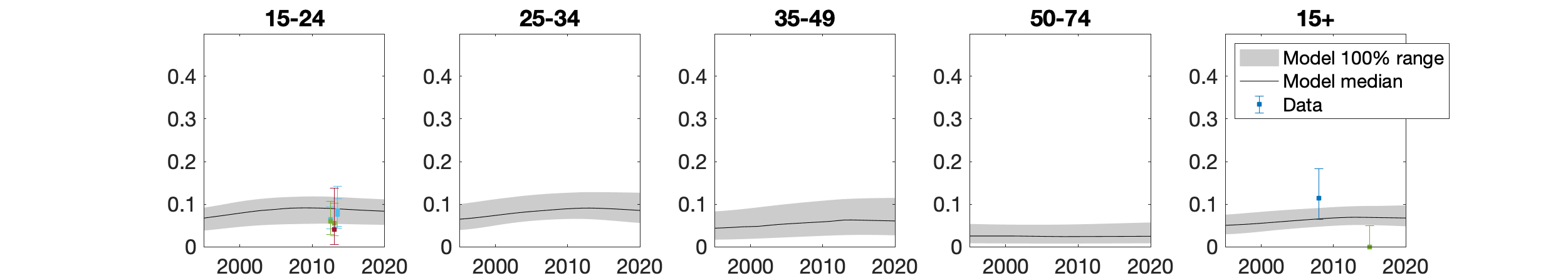


Figure 8.3 L) Modeled HPV 16/18 prevalence in HIV-negative women over time. Model outputs validated against varied data. (76,83,85,87,90,91)


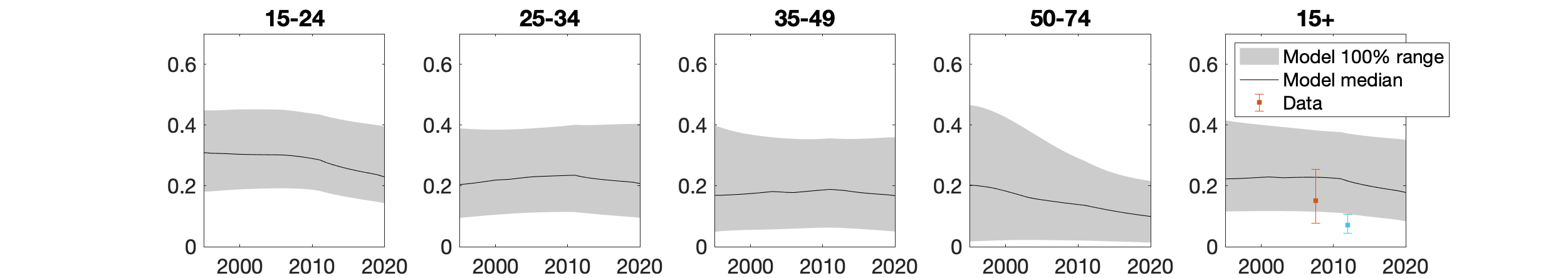


Figure 8.3 M) Modeled HPV 16/18 prevalence in men living with HIV over time. Model outputs validated against varied data.(95,96) Note the y-axis between figures M and N are different.


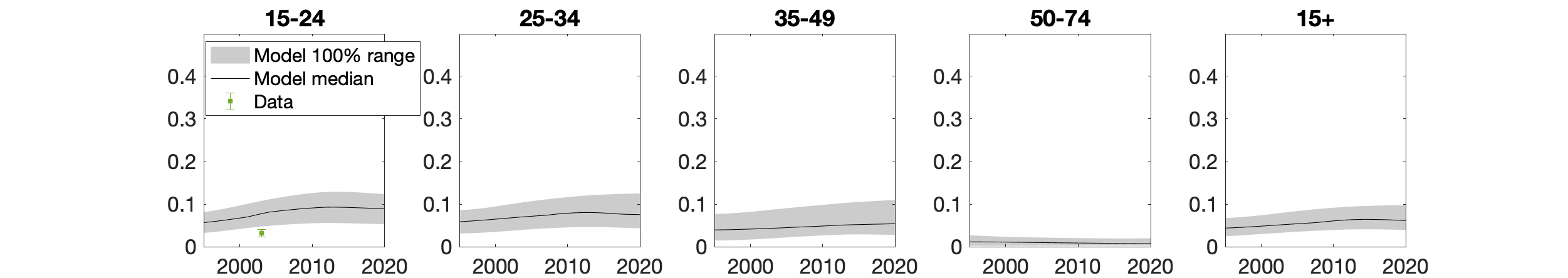


Figure 8.3 N) Modeled HPV 16/18 prevalence in HIV-negative men over time. Model outputs validated against varied data.(97) Note the y-axis between figures M and N are different.

CIN2+ prevalence in women


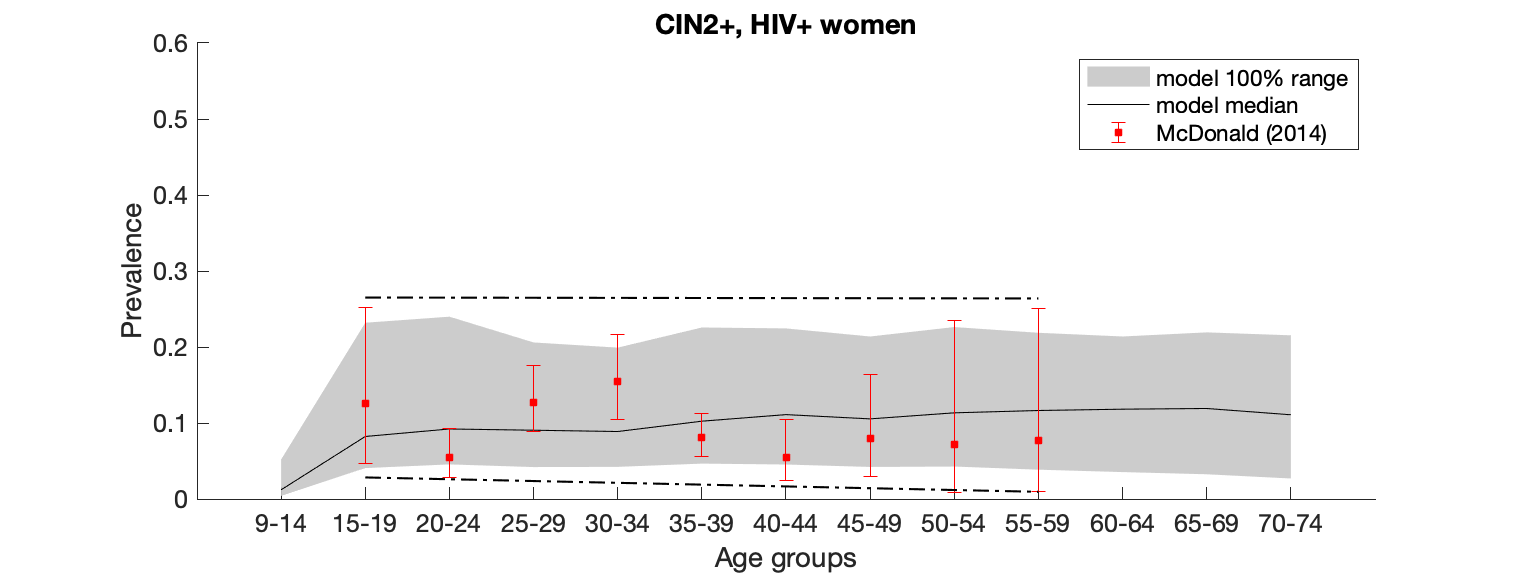


Figure 8.4 A) Predicted CIN2+ prevalence in women living with HIV by age in 2001(74) calibration. Dashed lines represent calibration target ranges.

N.B. First age group in McDonald is 17-19 years old, and we allowed the lower minimum prevalence range for 15-19 age group to reflect 15-16 years potentially having a lower prevalence.


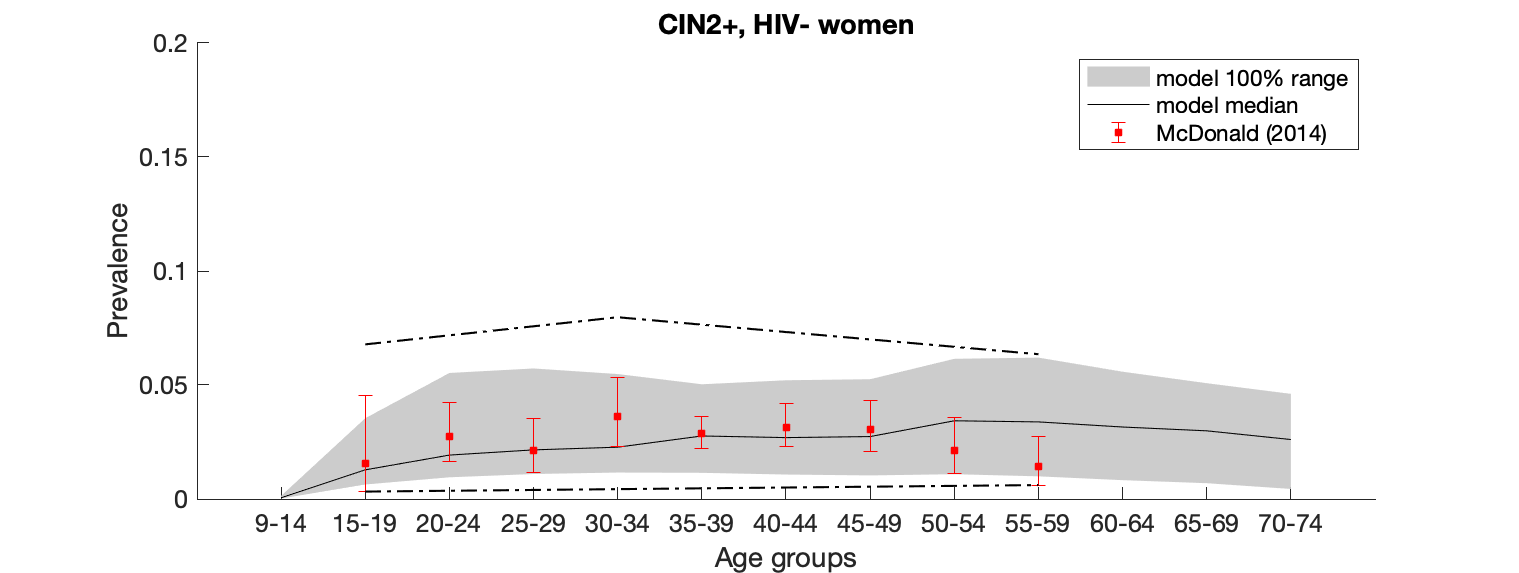


Figure 8.4 B) Predicted CIN2+ prevalence in women living with HIV by age in 2001(74) calibration. Dashed lines represent calibration target ranges. Note the axis range is different between A and B.

N.B. First age group in McDonald is 17-19 years old, and we allowed the lower minimum prevalence range for 15-19 age group to reflect 15-16 years potentially having a lower prevalence.

HPV type distribution


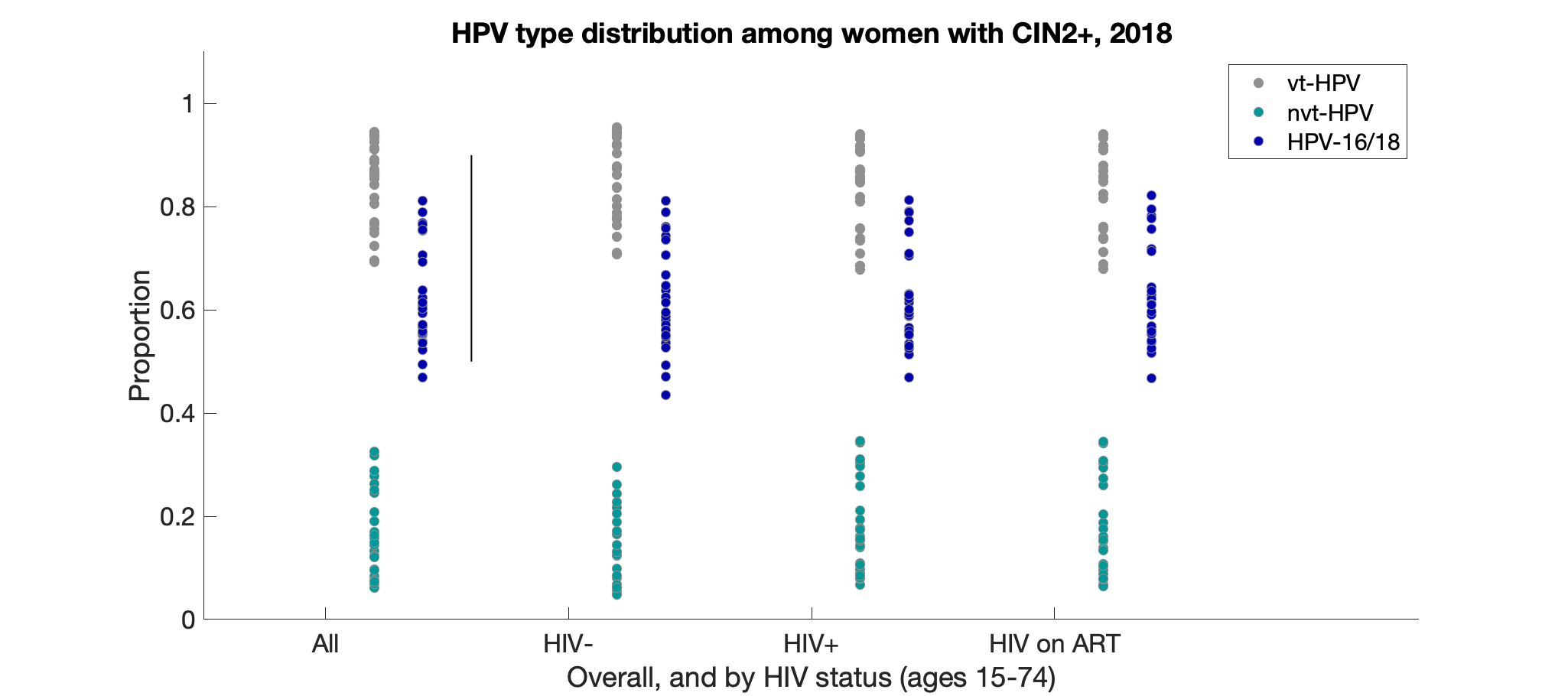


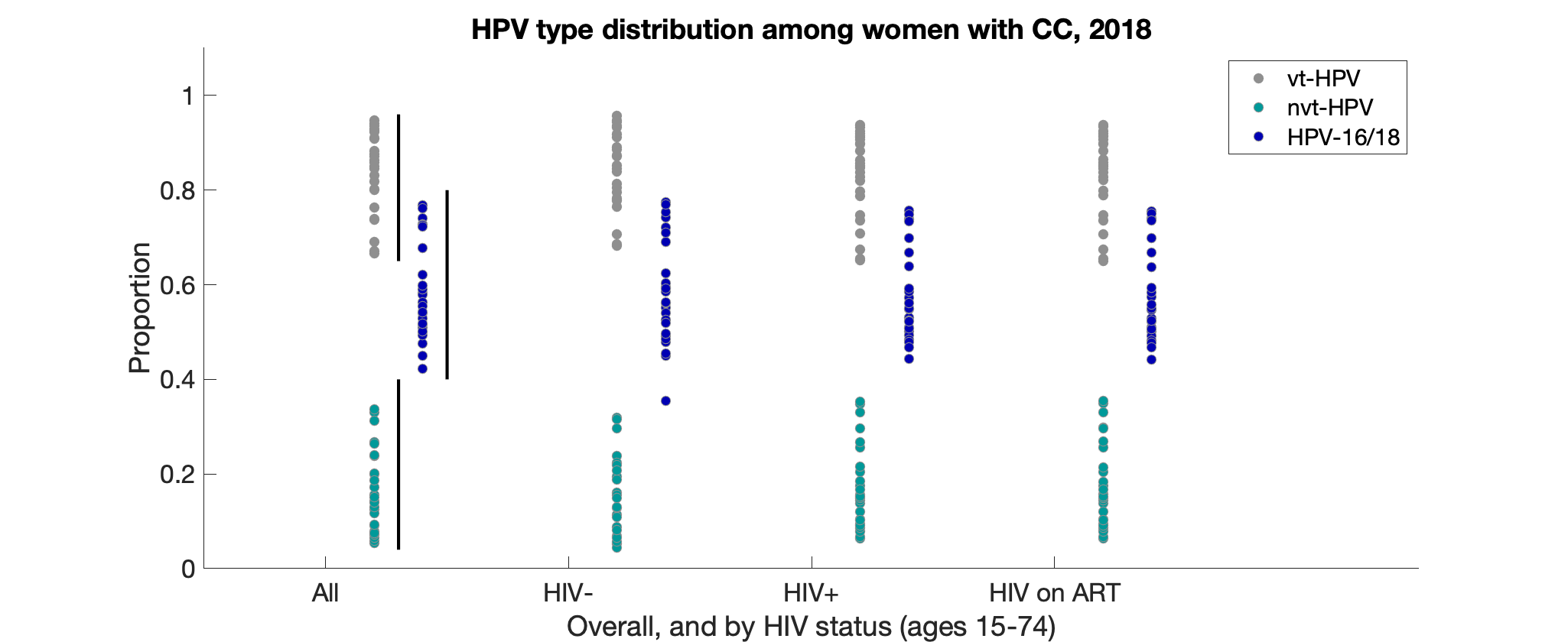
Figure 8.5 A) Modeled grouped HPV type distribution in women with CIN2+ . Vertical black line next to “all” reflects data ranges for vt-HPV (105)

Figure 8.5 B) Modeled grouped HPV type distribution in women with CC. Vertical black line next to “all” reflects data ranges for vt-HPV (105)


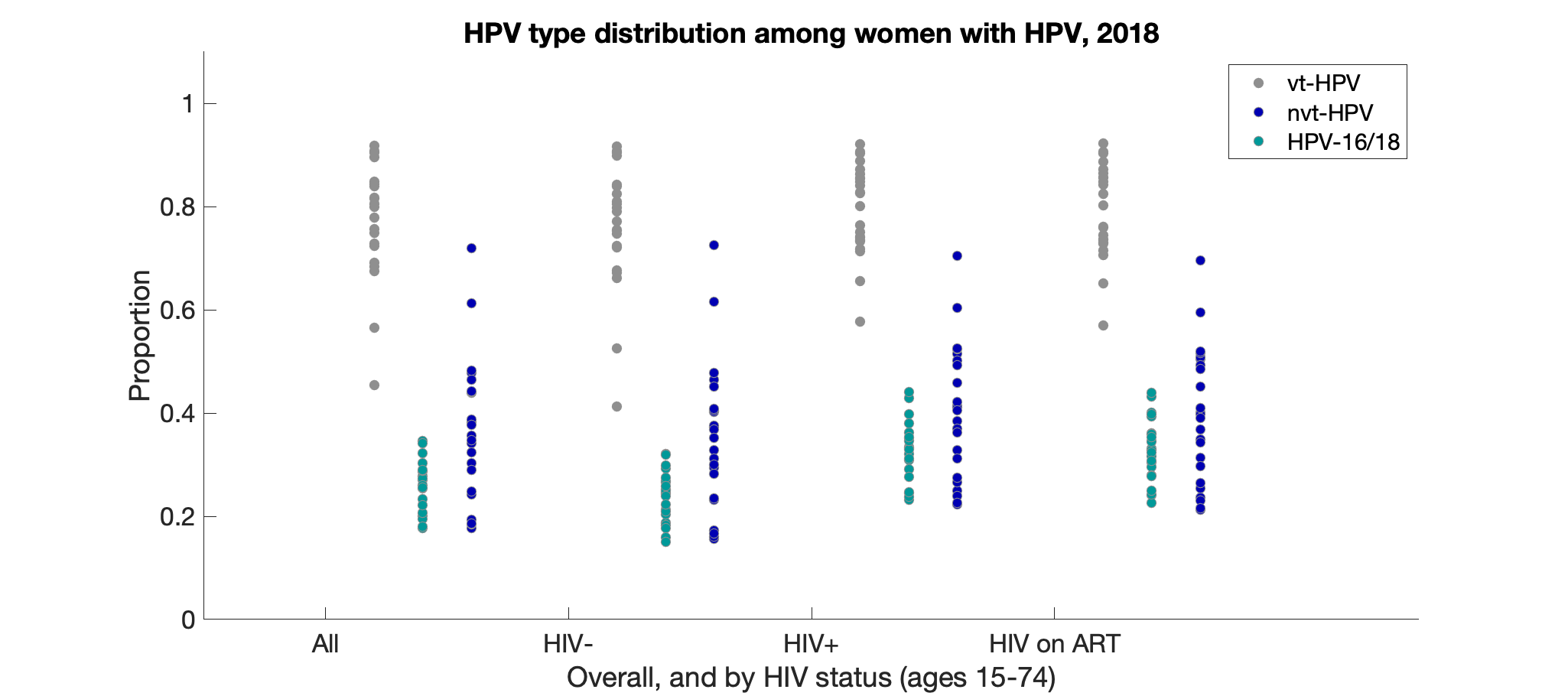


Figure 8.5 C) Modeled grouped HPV type distribution in women with HPV.

Cervical cancer incidence


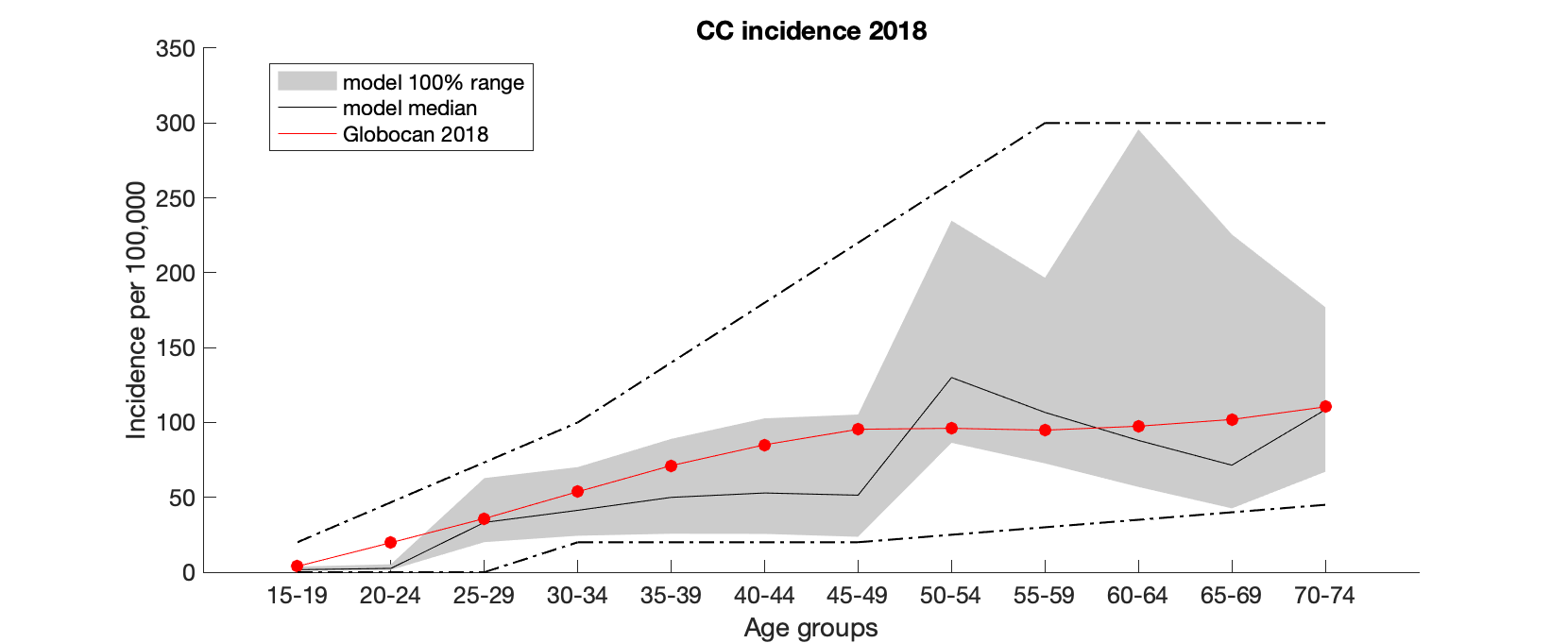


Figure 8.6.A) Modeled cervical cancer incidence in women in 2018, calibrated by age, target limits are presented by dashed lines. Data used are Globocan CC estimates for 2018


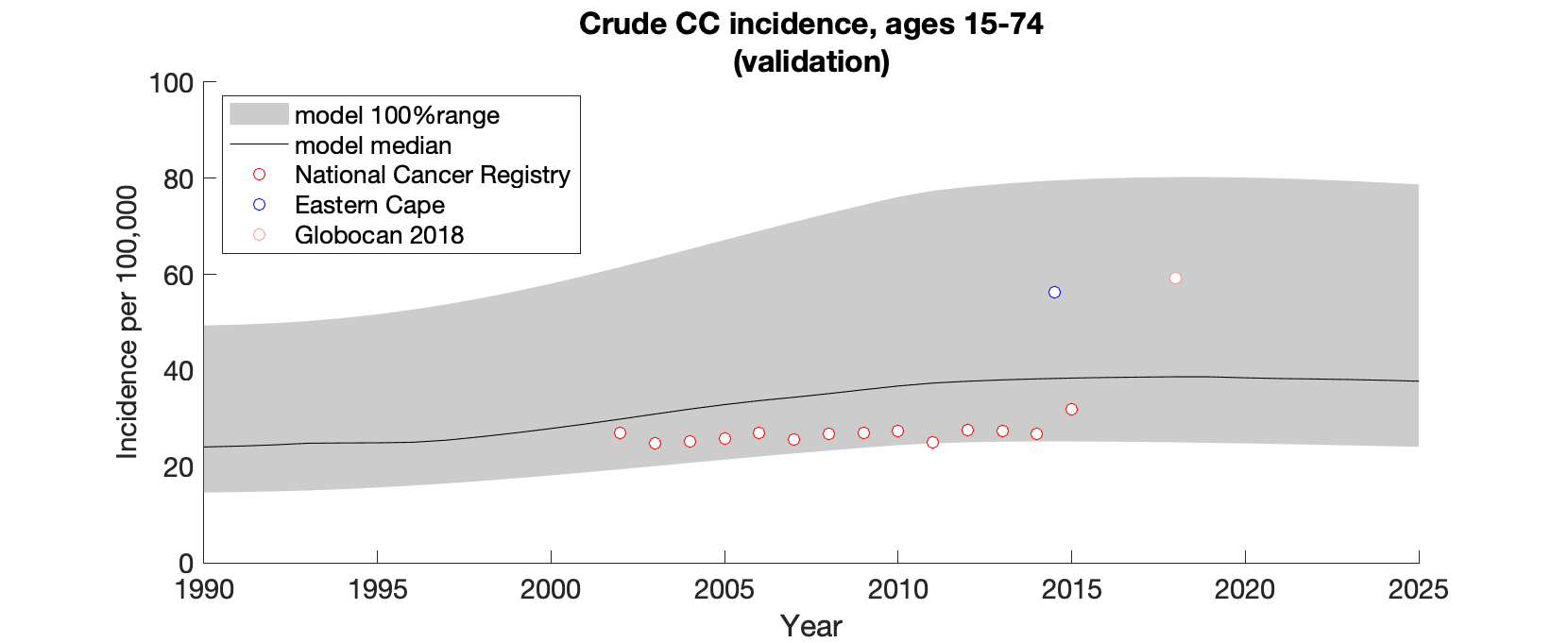


Figure 8.6.B) Modeled cervical cancer incidence over time. Validated against a range of estimates: National Cancer Registry estimates of CC diagnoses (assuming incidence should be higher than diagnoses), incidence estimates from Eastern Cape, and Globocan 2018 estimate. (101,102)

**9) Model equations**

Model equations are presented in the text in the relevant sections above.

**10) References**

1. United Nations. World Population Prospects [Internet]. [cited 2019 Apr 14]. Available from: https://population.un.org/wpp/

2. Human Sciences Research Council. South African National HIV Prevalence, HIV Incidence, Behaviour and Communication Survey (SABSSM) 2005 [Internet]. [cited 2022 Feb 17]. Available from: http://curation.hsrc.ac.za/index.php?module=pagesetter&type=user&func=hsrcdataset&ppnumber=PFAJLA&datasetno=6

3. Shisana O, Rehle T, Simbayi L, Parker W, Zuma K, Bhana A, et al. South African National HIV Prevalence, HIV Incidence, Behaviour and Communication Survey, 2005 – The Human Sciences Research Council (HSRC). 2005.

4. Stone J, Mukandavire C, Boily MC, Fraser H, Mishra S, Schwartz S, et al. Estimating the contribution of key populations towards HIV transmission in South Africa. J Int AIDS Soc. 2021 Jan 1;24(1).

5. Shisana O, Simbayi L, Council SAMR. South African National HIV Prevalence, HIV Incidence, Behaviour and Communication Survey, 2005. 2008. 204 p.

6. Garnett GP, Anderson RM. Balancing sexual partnerships in an age and activity stratified model of HIV transmission in heterosexual populations. IMA J Math Appl Med Biol. 1994/01/01. 1994;11(3):161–92.

7. Manhart LE, Koutsky LA. Do Condoms Prevent Genital HPV Infection, External Genital Warts, or Cervical Neoplasia ? Sex Transm Diseses. 2002;29(11):725–35.

8. Lam JUH, Rebolj M, Dugué PA, Bonde J, von Euler-Chelpin M, Lynge E. Condom use in prevention of human papillomavirus infections and cervical neoplasia: Systematic review of longitudinal studies. J Med Screen. 2014;21(1):38–50.

9. Ferrand RA, Corbett EL, Wood R, Hargrove J, Chiratidzo E. Europe PMC Funders Group Europe PMC Funders Author Manuscripts AIDS among older children and adolescents in Southern Africa : projecting the time course and magnitude of the epidemic. 2012;23(15):2039–46.

10. Brisson M. Technical Apendix HPV-ADVISE. 2012;1–82.

11. Van de Velde N, Brisson M, Boily M-C. Modeling Human Papillomavirus Vaccine Effectiveness: Quantifying the Impact of Parameter Uncertainty. Am J Epidemiol. 2007 Feb 19;165(7):762–75.

12. Campos NG, Burger E a, Sy S, Sharma M, Schiffman M, Rodriguez AC, et al. An Updated Natural History Model of Cervical Cancer: Derivation of Model Parameters. Am J Epidemiol. 2014 Sep 1;180(5):545–55.

13. Kelly H, Weiss HA, Benavente Y, de Sanjose S, Mayaud P, Qiao Y, et al. Association of antiretroviral therapy with high-risk human papillomavirus, cervical intraepithelial neoplasia, and invasive cervical cancer in women living with HIV: a systematic review and meta-analysis. Lancet HIV. 2018 Jan;5(1):e45–58.

14. Liu G, Sharma M, Tan N, Barnabas R. HIV-positive women have higher risk of HPV infection, precancerous lesions, and cervical cancer: a systematic review and meta-analysis. AIDS. 2018 Jan 24;1.

15. Looker KJ, Rönn MM, Brock PM, Brisson M, Drolet M, Mayaud P, et al. Evidence of synergistic relationships between HIV and Human Papillomavirus (HPV): systematic reviews and meta‐analyses of longitudinal studies of HPV acquisition and clearance by HIV status, and of HIV acquisition by HPV status. J Int AIDS Soc. 2018;21(6).

16. Weller SC, Davis-Beaty K. Condom effectiveness in reducing heterosexual HIV transmission. Cochrane Database Syst Rev. 2002 Jan 21;(1).

17. Mags B, Phumla N, Zonke M, Jenni S, Bongiwe Z, Lungile P, et al. Twenty years of the female condom programme in South Africa : past, present, and future. South African Heal Rev. 2017;2017(1):147–56.

18. Siegfried N, Muller M, Deeks JJ, Volmink J. Male circumcision for prevention of heterosexual acquisition of HIV in men. Cochrane Database Syst Rev. 2009 Apr 15;(2):CD003362.

19. World Health Organization. Progress in scaling up voluntary medical male circumcision for HIV prevention in East and Southern Africa: January - December 2012. World Heal Organ. 2012;(December):1–25.

20. Shisana O, CSimbayi L. Nelson Mandela/HSRC study of HIV/AIDS: South African national HIV prevalence, behavioural risks and mass media: household survey 2002.

21. Wilcken A, Keil T, Dick B. Traditional male circumcision in eastern and southern Africa: a systematic review of prevalence and complications. Bull World Health Organ. 2010 Dec 1;88(12):907–14.

22. Nordling L. A New Era for HIV. Nature. 2016 Jul 13;535(7611):214–7.

23. World Bank. Antiretroviral therapy coverage (% of people living with HIV) - South Africa [Internet]. [cited 2022 Feb 10]. Available from: https://data.worldbank.org/indicator/SH.HIV.ARTC.ZS?locations=ZA

24. South Africa | UNAIDS [Internet]. Available from: http://www.unaids.org/en/regionscountries/countries/southafrica

25. South African Department of Health. National Guideline for Cervical Cancer Screening Programme. 2000.

26. Bruni L, Albero G, Serrano B, Mena M, Collado J, Gómez D, et al. Human Papillomavirus and Related Diseases in South Africa. Summary Report 22 October 2021. [Internet]. ICO/IARC Information Centre on HPV and Cancer (HPV Information Centre). [cited 2022 Feb 15]. Available from: www.hpvcentre.net

27. Phaswana-Mafuya N, Peltzer K. Breast and Cervical Cancer Screening Prevalence and Associated Factors among Women in the South African General Population. Asian Pac J Cancer Prev. 2018 Jun 1;19(6):1465.

28. Human Sciences Research Council. South African National HIV Prevalence, Incidence and Behaviour Survey, 2012 [Internet]. [cited 2020 Jul 2]. Available from: https://www.hsrcpress.ac.za/books/south-african-national-hiv-prevalence-incidence-and-behaviour-survey-2012

29. Khozaim K, Orang’O E, Christoffersen-Deb A, Itsura P, Oguda J, Muliro H, et al. Successes and challenges of establishing a cervical cancer screening and treatment program in western Kenya. Int J Gynaecol Obstet. 2014;124(1):12–8.

30. Coleman JS, Cespedes MS, Cu-Uvin S, Kosgei RJ, Maloba M, Anderson J, et al. An Insight into Cervical Cancer Screening and Treatment Capacity in Sub-Saharan Africa HHS Public Access. J Low Genit Tract Dis. 2016;20(1):31–7.

31. Debeaudrap P, Sobngwi J, Tebeu PM, Clifford GM. Residual or Recurrent Precancerous Lesions After Treatment of Cervical Lesions in Human Immunodeficiency Virus-infected Women: A Systematic Review and Meta-analysis of Treatment Failure. Clin Infect Dis. 2019 Oct 15;69(9):1555–65.

32. Hoffman SR, Le T, Lockhart A, Sanusi A, Dal Santo L, Davis M, et al. Patterns of persistent HPV infection after treatment for cervical intraepithelial neoplasia (CIN): A systematic review. Int J cancer. 2017 Jul 1;141(1):8–23.

33. Batra P, Kuhn L, Denny L. Utilisation and outcomes of cervical cancer prevention services among HIV-infected women in Cape Town. South African Med J. 2010;100(1):39–44.

34. S J, P M, K R, C S, J B. A Review of Cervical Cancer in South Africa: Previous, Current and Future. Heal Care Curr Rev. 2016;04(04).

35. Sankaranarayanan R, Gaffikin L, Jacob M, Sellors J, Robles S. A critical assessment of screening methods for cervical neoplasia. Int J Gynaecol Obstet. 2005;89 Suppl 2(SUPPL. 2).

36. Firnhaber C, Mayisela N, Mao L, Williams S, Swarts A, Faesen M, et al. Validation of Cervical Cancer Screening Methods in HIV Positive Women from Johannesburg South Africa. PLoS One. 2013 Jan 17;8(1):e53494.

37. Taylor S, Kuhn L, Dupree W, Denny L, De Souza M, Wright TC. Direct comparison of liquid-based and conventional cytology in a South African screening trial. Int J cancer. 2006 Feb 15;118(4):957–62.

38. Nattrass N, Maughan-Brown B, Seekings J, Whiteside A. Poverty, sexual behaviour, gender and HIV infection among young black men and women in Cape Town, South Africa. African J AIDS Res. 2012;11(4):307–17.

39. Quaife M, Eakle R, Cabrera M, Vickerman P, Tsepe M, Cianci F, et al. Preferences for ARV-based HIV prevention methods among men and women, adolescent girls and female sex workers in Gauteng Province, South Africa: A protocol for a discrete choice experiment. BMJ Open. 2016 Jun 1;6(6).

40. Carael M, Slaymaker E, Lyerla R, Sarkar S. Clients of sex workers in different regions of the world: Hard to count. Sex Transm Infect. 2006 Jun;82(SUPPL. III).

41. Shisana. South African National HIV Prevalence, Incidence, and Behavior Survey: Early Data Resease. 2017.

42. S.P.Reddy SJ, R.Sewpaul SS, A.Ellahebokus, N.S.Kambaran RGO. Department of the Human Sciences Research Council South Africa, 2011. Umthente Uhlaba Usamila - The 3rd South African National Survey, Youth Risk Behaviour 2011, [Internet]. [cited 2021 May 20]. Available from: http://www.hsrc.ac.za/en/research-data/view/6874

43. South African Health Monitoring Survey. SAHMS Final Report Survey on female sex workers in South Africa. 2014;

44. Rao A, Baral S, Phaswana-Mafuya N, Lambert A, Kose Z, Mcingana M, et al. Pregnancy intentions and safer pregnancy knowledge among female sex workers in Port Elizabeth, South Africa. Obstet Gynecol. 2016 Jul 1;128(1):15–21.

45. Peltzer K, Mashego TA, Mabeba M. Attitudes and practices of doctors toward domestic violence victims in South Africa. Health Care Women Int. 2003;24(2):149–57.

46. Quaife M, Eakle R, Cabrera M, Vickerman P, Tsepe M, Cianci F, et al. Preferences for ARV-based HIV prevention methods among men and women, adolescent girls and female sex workers in Gauteng Province, South Africa: a protocol for a discrete choice experiment. BMJ Open. 2016 Jun 27;6(6):e010682.

47. Owen BN, Elmes J, Silhol R, Dang Q, McGowan I, Shacklett B, et al. How common and frequent is heterosexual anal intercourse among South Africans? A systematic review and meta-analysis. J Int AIDS Soc. 2017;19(1):21162.

48. Owen BN, Baggaley RF, Elmes J, Harvey A, Shubber Z, Butler AR, et al. What Proportion of Female Sex Workers Practise anal Intercourse and How Frequently? A Systematic Review and Meta-analysis. AIDS and Behavior. 2019.

49. Boily MC, Baggaley RF, Wang L, Masse B, White RG, Hayes RJ, et al. Heterosexual risk of HIV-1 infection per sexual act: systematic review and meta-analysis of observational studies. Lancet Infect Dis. 2009/01/31. 2009;9(2):118–29.

50. Baggaley RF, Owen BN, Silhol R, Elmes J, Anton P, McGowan I, et al. Does per-act HIV-1 transmission risk through anal sex vary by gender? An updated systematic review and meta-analysis. Am J Reprod Immunol. 2018 Nov;80(5):e13039.

51. Houben RMGJ, Menzies NA, Sumner T, Huynh GH, Arinaminpathy N, Goldhaber-Fiebert JD, et al. Feasibility of achieving the 2025 WHO global tuberculosis targets in South Africa, China, and India: a combined analysis of 11 mathematical models. Lancet Glob Heal. 2016 Nov 1;4(11):e806–15.

52. Hollingsworth TD, Anderson RM, Fraser C. HIV-1 transmission, by stage of infection. J Infect Dis. 2008;198(5):687–93.

53. Morgan D, Mahe C, Mayanja B, Okongo JM, Lubega R, Whitworth J a G. HIV-1 infection in rural Africa: is there a difference in median time to AIDS and survival compared with that in industrialized countries? AIDS. 2002;16(4):597–603.

54. HIV-CAUSAL Collaboration, Ray M, Logan R, Sterne JAC, Hernández-Díaz S, Robins JM, et al. The effect of combined antiretroviral therapy on the overall mortality of HIV-infected individuals. AIDS. 2010 Jan 2;24(1):123–37.

55. Maheu-Giroux M, Vesga JF, Diabaté S, Alary M, Baral S, Diouf D, et al. Changing Dynamics of HIV Transmission in Côte dʼIvoire. JAIDS J Acquir Immune Defic Syndr. 2017 Aug 15;75(5):517–27.

56. Johnson LF, Mossong J, Dorrington RE, Schomaker M, Hoffmann CJ, Keiser O, et al. Life expectancies of South African adults starting antiretroviral treatment: collaborative analysis of cohort studies. PLoS Med. 2013;10(4):e1001418.

57. Johnson LF, Chiu C, Myer L, Davies M-A, Dorrington RE, Bekker L-G, et al. Prospects for HIV control in South Africa: a model-based analysis. Glob Health Action. 2016 Dec 8;9(1):30314.

58. Donnell D, Baeten JM, Kiarie J, Thomas KK, Stevens W, Cohen CR, et al. Heterosexual HIV-1 transmission after initiation of antiretroviral therapy: a prospective cohort analysis. Lancet. 2010;375(9731):2092–8.

59. Attia S, Egger M, Müller M, Zwahlen M, Low N. Sexual transmission of HIV according to viral load and antiretroviral therapy: systematic review and meta-analysis. AIDS. 2009 Jul;23(11):1397–404.

60. Rosen S, Maskew M, Fox MP, Nyoni C, Mongwenyana C, Malete G, et al. Initiating Antiretroviral Therapy for HIV at a Patient’s First Clinic Visit: The RapIT Randomized Controlled Trial. Binagwaho A, editor. PLOS Med. 2016 May 10;13(5):e1002015.

61. Mberi MN, Kuonza LR, Dube NM, Nattey C, Manda S, Summers R. Determinants of loss to follow-up in patients on antiretroviral treatment, South Africa, 2004–2012: a cohort study. BMC Health Serv Res. 2015 Dec 4;15(1):259.

62. Eakle R, Gomez GB, Naicker N, Bothma R, Mbogua J, Cabrera Escobar MA, et al. HIV pre-exposure prophylaxis and early antiretroviral treatment among female sex workers in South Africa: Results from a prospective observational demonstration project. Bekker L-G, editor. PLOS Med. 2017 Nov 21;14(11):e1002444.

63. Menon S, Rossi R, Kariisa M, Acharya SD, Zdraveska N, Mahmood S, et al. Relationship between Highly Active Antiretroviral Therapy (HAART) and human papillomavirus type 16 (HPV 16) infection among women in Sub-Saharan Africa and public health implications: A systematic review. PLoS One. 2019 Mar 1;14(3):e0213086.

64. Viscidi RP, Ahdieh-Grant L, Schneider MF, Clayman B, Massad LS, Anastos KM, et al. Serum immunoglobulin A response to human papillomavirus type 16 virus-like particles in human immunodeficiency virus (HIV)-positive and high-risk HIV-negative women. J Infect Dis. 2003;188:1834–44.

65. O.Shisana, T.Rehle, L.C.Simbayi, K.Zuma, S.Jooste, N.Zungu, D.Labadarios DO. South African national HIV Prevalence, Incidence and Behaviour Survey. 2012. 1–198 p.

66. UNAIDS. AIDSinfo [Internet]. [cited 2019 Apr 14]. Available from: http://aidsinfo.unaids.org/

67. HSRC. South African National HIV Prevalence Behaviour and Communication Survey [Internet]. 2017 [cited 2019 Sep 23]. Available from: http://www.hsrc.ac.za/en/departments/saph/HAST_National_HIV_Survey

68. Ramjee G, Williams B, Gouws E, Van Dyck E, De Deken B, Karim SA. The impact of incident and prevalent herpes simplex virus-2 infection on the incidence of HIV-1 infection among commercial sex workers in South Africa. J Acquir Immune Defic Syndr. 2005;39(3):333–9.

69. K.L. D, M.E. B, V.H. R, R.C. B, Y. H, M.L. W. Risk factors for HIV infection among sex workers in Johannesburg, South Africa. Int J STD AIDS. 2005;16(3):256–61.

70. Schwartz S, Lambert A, Phaswana-Mafuya N, Kose Z, McIngana M, Holland C, et al. Engagement in the HIV care cascade and barriers to antiretroviral therapy uptake among female sex workers in Port Elizabeth, South Africa: Findings from a respondent-driven sampling study. Sex Transm Infect. 2017;93(4):290–6.

71. Williams B, Gilgen D, Taljaard D, Campbell C. The Natural History of HIV / AIDS in South Africa. Industrial Research. 2000. 1–218 p.

72. Olorunfemi G, Ndlovu N, Masukume G, Chikandiwa A, Pisa PT, Singh E. Temporal trends in the epidemiology of cervical cancer in South Africa (1994-2012). Int J Cancer. 2018 Nov 1;143(9):2238–49.

73. ANOVA, Wits RHI. South African Health Monitoring Survey (SAHMS): An Integrated Biological and Behavioural Survey among Female Sex Workers , South Africa 2013 – 2014 Final Report. 2016;(Cdc).

74. McDonald AC, Tergas AI, Kuhn L, Denny L, Wright TC. Distribution of Human Papillomavirus Genotypes among HIV-Positive and HIV-Negative Women in Cape Town, South Africa. Front Oncol. 2014;4(March):1–11.

75. Mbulawa ZZA, Coetzee D, Williamson A-L. Human papillomavirus prevalence in South African women and men according to age and human immunodeficiency virus status. BMC Infect Dis. 2015;15(1):459.

76. Mbulawa ZZA, Van Schalkwyk C, Hu NC, Meiring TL, Barnabas S, Dabee S, et al. High human papillomavirus (HPV) prevalence in South African adolescents and young women encourages expanded HPV vaccination campaigns. PLoS One. 2018;13(1):1–15.

77. Denny L, Boa R, Williamson AL, Allan B, Hardie D, Stan R, et al. Human papillomavirus infection and cervical disease in human immunodeficiency virus-1-infected women. Obstet Gynecol. 2008;111(6):1380–7.

78. Dols JAM, Reid G, Brown JM, Tempelman H, Bontekoe TR, Quint WG V., et al. HPV Type Distribution and Cervical Cytology among HIV-Positive Tanzanian and South African Women. ISRN Obstet Gynecol. 2012;2012:1–5.

79. Firnhaber C, Swarts A, Goeieman B, Rakhombe N, Mulongo M, Williamson AL, et al. Cryotherapy reduces progression of cervical intraepithelial neoplasia grade 1 in South African HIV-infected women: A randomized, controlled trial. J Acquir Immune Defic Syndr. 2017;76(5):532–8.

80. Ngou J, Magooa MP, Gilham C, Djigma F, Didelot MN, Kelly H, et al. Comparison of careHPV and hybrid capture 2 assays for detection of high-risk human Papillomavirus DNA in cervical samples from hiv-1-infected African women. J Clin Microbiol. 2013;51(12):4240–2.

81. McDonald AC, Tergas AI, Kuhn L, Denny L, Wright TC. Distribution of Human Papillomavirus Genotypes among HIV-Positive and HIV-Negative Women in Cape Town, South Africa. Front Oncol. 2014 Jan;4(March):48.

82. McDonald AC, Denny L, Wang C, Tsai W-Y, Wright TC, Kuhn L. Distribution of High-Risk Human Papillomavirus Genotypes among HIV-Negative Women with and without Cervical Intraepithelial Neoplasia in South Africa. PLoS One. 2012 Jan;7(9):e44332.

83. Giuliano AR, Botha MH, Zeier M, Abrahamsen ME, Glashoff RH, van der Laan LE, et al. High HIV, HPV, and STI Prevalence Among Young Western Cape, South African Women. JAIDS J Acquir Immune Defic Syndr. 2015;68(2):227–35.

84. Barnabas SL, Dabee S, Passmore JAS, Jaspan HB, Lewis DA, Jaumdally SZ, et al. Converging epidemics of sexually transmitted infections and bacterial vaginosis in southern African female adolescents at risk of HIV. Int J STD AIDS. 2018;29(6):531–9.

85. Adler DH, Wallace M, Bennie T, Mrubata M, Abar B, Meiring TL, et al. Cervical dysplasia and high-risk human papillomavirus infections among HIV-infected and HIV-uninfected adolescent females in South Africa. Infect Dis Obstet Gynecol. 2014;2014.

86. Auvert B, Marais D, Lissouba P, Zarca K, Ramjee G, Williamson AL. High-risk human papillomavirus is associated with HIV acquisition among South African female sex workers. Infect Dis Obstet Gynecol. 2011;2011.

87. Kriek JM, Jaumdally SZ, Masson L, Little F, Mbulawa Z, Gumbi PP, et al. Female genital tract inflammation, HIV co-infection and persistent mucosal Human Papillomavirus (HPV) infections. Virology. 2016;493:247–54.

88. Kelly H, Faust H, Chikandiwa A, Ngou J, Weiss HA, Segondy M, et al. Human papillomavirus serology among women living with HIV: Type-Specific seroprevalence, seroconversion, and risk of cervical reinfection. J Infect Dis. 2018;218(6):927–36.

89. Wang C, Wright TC, Denny L, Kuhn L. Rapid rise in detection of human papillomavirus (HPV) infection soon after incident HIV infection among south African women. J Infect Dis. 2011;203:479–86.

90. Sudenga SL, Torres BN, Botha MH, Zeier M, Abrahamsen ME, Glashoff RH, et al. Cervical HPV Natural History Among Young Western Cape, South African Women: The Randomized Control EVRI Trial. J Infect. 2015 Oct 14;

91. Vuyst H De, Ndirangu G, Moodley M, Tenet V, Estambale B, Meijer CJLM, et al. Prevalence of human papillomavirus in women with invasive cervical carcinoma by HIV status in Kenya and South Africa. Int J Cancer. 2012;131(4):949–55.

92. Marais DJ, Vardas E, Ramjee G, Allan B, Kay P, Rose RC, et al. The impact of Human Immunodeficiency Virus type 1 status on Human Papillomavirus (HPV) prevalence and HPV antibodies in serum and cervical secretions. J Infect Dis. 2000;182(4):1239–42.

93. Firnhaber C, Evans D, Friedman-Khalili R, Willliams S, Michelow P, Matlhagela K, et al. Seroprevalence of HPV vaccine types 6, 11, 16 and 18 in HIV-infected women from South Africa, Brazil and Botswana. J Clin Virol. 2011;52(3):265–8.

94. Richter KL, Van Rensburg EJ, Van Heerden WFP, Boy SC. Human papilloma virus types in the oral and cervical mucosa of HIV-positive South African women prior to antiretroviral therapy. J Oral Pathol Med. 2008;37(9):555–9.

95. Firnhaber C, Sello M, Maskew M, Williams S, Schulze D, Williamson AL, et al. Human papillomavirus types in HIV seropositive men with penile warts in Johannesburg, South Africa. Int J STD AIDS. 2011;22(2):107–9.

96. Chikandiwa A, Chimoyi L, Pisa PT, Chersich MF, Muller EE, Michelow P, et al. Prevalence of anogenital HPV infection, related disease and risk factors among HIV-infected men in inner-city Johannesburg, South Africa: Baseline findings from a cohort study. BMC Public Health. 2017;17(Suppl 3).

97. Auvert B, Lissouba P, Cutler E, Zarca K, Puren A, Taljaard D. Association of oncogenic and nononcogenic human papillomavirus with HIV incidence. J Acquir Immune Defic Syndr. 2010;53(1):111–6.

98. Mbulawa ZZA, Van Schalkwyk C, Hu NC, Meiring TL, Barnabas S, Dabee S, et al. High human papillomavirus (HPV) prevalence in South African adolescents and young women encourages expanded HPV vaccination campaigns. PLoS One. 2018 Jan 1;13(1).

99. McDonald AC, Denny L, Wang C, Tsai W-Y, Wright TC, Kuhn L, et al. Distribution of High-Risk Human Papillomavirus Genotypes among HIV-Negative Women with and without Cervical Intraepithelial Neoplasia in South Africa. de Sanjose S, editor. PLoS One. 2012 Sep 6;7(9):e44332.

100. Mbulawa ZZ a, Johnson LF, Marais DJ, Coetzee D, Williamson A-L. The impact of human immunodeficiency virus on human papillomavirus transmission in heterosexually active couples. J Infect. 2013;67(1):51–8.

101. National Institute For Communicable Diseases. National Cancer Registry [Internet]. [cited 2022 Feb 25]. Available from: https://www.nicd.ac.za/centres/national-cancer-registry/

102. International Association of Cancer Registries. Table by cancers [Internet]. [cited 2022 Feb 25]. Available from: https://ci5.iarc.fr/CI5-XI/Pages/summary_table_pop_sel.aspx

103. World Health Organization, International Agency for Research on Cancer. Global Cancer Observatory [Internet]. [cited 2019 Apr 14]. Available from: https://gco.iarc.fr/

104. National Institute for Communicable Diseases. National Cancer Registry [Internet]. [cited 2019 Apr 13]. Available from: http://www.nicd.ac.za/centres/national-cancer-registry/

105. Data Query | ICO Information Centre on HPV and Cancer [Internet]. [cited 2017 Jan 13]. Available from: http://www.hpvcentre.net/dataquery.php
