# Supplementary material for: Estimating the effect of HIV on cervical cancer elimination in South Africa: Comparative modelling of the impact of vaccination and screening
Source: eClinicalMedicine. 2022 Nov 17;54:101754. doi: 10.1016/j.eclinm.2022.101754 (PMC9793279; doi:10.1016/j.eclinm.2022.101754)
Supplement: Model specific _Technical Appendix _T2 [file mmc8.docx]

**Model-Specific Technical Appendix T2:** Technical material for the *MicroCOSM-HPV* model

*(National level)*

**Table of content**

Model overview………………………………………………………………………….…………….Page 1

Technical description of the *MicroCOSM-HPV* model (South Africa) ……………………………… Page 2

1. Demography, sexual behaviour and transmission…………………………………………………Page 2
2. Natural history of infection and disease progression…………………………………………….. Page 2
3. Baseline interventions – for HIV……………………………………………………………….....Page 4
4. Baseline interventions - for HPV and Cervical cancer…………………………………………... Page 5
5. Model calibration page……………………………………………………………………………Page 7
6. Parameter estimates and fitting/validations outcomes and data sources……………………….....Page 8
7. Description of analysis……………………………………………………………………………Page 14
8. Fitting and validation results……………………………………………………………………...Page 15
9. Model equations…………………………………………………………………………………..Page 21
10. References ………………………………………………………………………………………..Page 22

**Model overview**

*MicroCOSM-HPV* is a model for South Africa at the national level. Briefly, *MicroCOSM-HPV* is an individual-based stochastic model representing 1 million individuals in 1985. The model simulates HIV and HPV transmission (13 independent high-risk types: vaccine types 16, 18, 31, 33, 45, 52, 58, and non-vaccine types: 35, 39, 51, 56, 59, 68) and HPV-induced cervical carcinogenesis associated with each type among heterosexual individuals by sex, single age cohorts, and sexual risk. Each health state represents the underlying true health state of each simulated individual (as opposed to a diagnosed state) such as active infection, latent infection and natural immunity status, cervical intraepithelial neoplasia (CIN1, 2, 3), and 8 cancer stages (4 stages undiagnosed and 4 diagnosed) (see model flowchart below). The model assumes that HIV increases the risk of persistent HPV infections and disease progression to cervical cancer among women with HIV, whereas ART reduces the risk of HPV and disease progression compared to women with HIV not on ART but not completely compared to HIV negative women. Condom use and ART are also effective against HPV infections and/or disease progression. Male circumcision is assumed to have no effect on HPV acquisition for men or transmission from men to women. HIV infection does not influence the duration of natural immunity, but reactivation of a latent infection depends on the stage of HIV infection, and ART status. No effect of HPV on HIV is assumed (Table T3).

The model accounts for changes in the levels of HIV interventions such as condom use and HIV treatment over time since the beginning of the HIV epidemic in 1990. The model also represents baseline cervical cancer screening and treatment through a detailed screening and diagnosis algorithm (starting in 2000).

Model transitions between health states are governed by probabilities that depend on sex, age, HPV type, and HIV/ART status. The probabilities of HPV or HIV transmission among susceptible individuals depend on their sexual activity, the prevalence of infection among partners, and level of interventions (condom use, ART status). The per sex-act transmission probability for HPV is independent of HIV status (of the individual or their partner), and vice versa.

The model structures of HIV and HPV infection are summarised in figure T2.1. We provide a summary of the natural histories of both infections and their co-infection dynamics below. Detailed descriptions of the model structure, parameters, calibration methods and the data used can be found in the main text and supplementary materials of Van Schalkwyk et al. (1) and Johnson and Geffen (2).

The model was parameterised and fitted using multivariate calibration process based on detailed data on sexual behaviour, HIV and HPV infections and cervical cancer epidemiology over time representative of the South African context. The data sources used at this stage are described below.

**Technical description of the *MicroCOSM-HPV*** **model (South Africa)**

1. **Demography, sexual behaviour and transmission:**

*MicroCOSM-HPV* simulates an open and growing heterosexual population of all ages representative of the South African population (3–5). Since this is an individual-based model, only a fraction of the population is simulated to limit the use of computing resources. This model was not developed to simulate detailed demographic changes (e.g., international migration is not considered) and HIV prevention is limited to changes in condom use and ART coverage. For these reasons, we reweight the population totals in our model using the projected population demographics (age, sex, HIV and ART status) of the *Thembisa* model, on the assumption that the *Thembisa* model estimates future HIV and demographic trends more realistically. This model is used by UNAIDS as the official source of HIV estimates for South Africa and is used to inform investment in HIV prevention by the National Department of Health (6–8).

At birth, or in 1985, an individual is randomly assigned the static status of high-risk or low-risk based on the propensity for concurrent partnerships or commercial sex. All individuals become sexually active between ages 10 and 30. At each time step (weekly), individuals looking for sexual partners are matched to other individuals looking for partners (random events based on sexual mixing probabilities by sex, age, risk group). When low-risk individuals are single, they will be looking for one partner. High-risk individuals may be looking for a primary partner, secondary partner or, if male, a contact with a female sex worker (a maximum of two concurrent partnerships). All relationships start as short-term (at rates determined by age, sex, and risk group), which may after an average of 6 months dissolve or become a long-term relationship (rates determined by age and sex). Rates at which sexual behaviour related events occur (sexual debut, partnership formation/dissolution, coital frequencies, etc) have been estimated in previous publications (2,9) and details regarding the wide variety of data sources and the calibration methods to estimate the rates are documented in these publications.

**HIV force of infection:** HIV transmission from an infected individual to a susceptible individual is modelled based on a probability of transmission per sex act (2). These probabilities depend on the relationship type (short-term, long-term, client-sex worker, Table T2.1), sex, age (young females are more susceptible), condom use (90% effective to prevent transmission), stage of HIV infection (acute, latent, late) and ART status. The reduction in infectiousness of those on ART increases over time (up to 2020) to account for increasing levels of viral suppression (due to improvements in drug regimens over time). Those who have interrupted treatment are as infectious as untreated individuals in the latent stage of HIV infection. Condom use reduces the per sex act HIV transmission probability by 90%. We assume that HPV status has no effect on the susceptibility to HIV or infectiousness of HIV. Transmission occurs through heterosexual relationships, or from mother to child during birth or during the first six months of life.

**HPV force of infection:** HPV transmission from an infected individual (in HPV positive or cervical pre-cancer stages in Figure T2.1) to a susceptible individual is modelled based on a probability of transmission per sex act. These probabilities depend on the HPV type and sex (Table T2.3). Individuals who are latently infected or have natural immunity are not infectious, and we assume that women with cervical cancer are not sexually active. Infection with one HPV type does not influence the transmission probability of another type and probabilities are independent of cervical disease stage. HIV status does not influence the per sex act transmission probability for HPV. Condom use is 70% effective against transmission for males and females (10) and male circumcision is not protective against infection with HPV (11).

1. **Natural history of infection and disease progression**

The model represents HIV and HPV transmission, disease progression, interaction between HIV and HPV, and the impact of prevention and treatment interventions on disease outcomes.

*
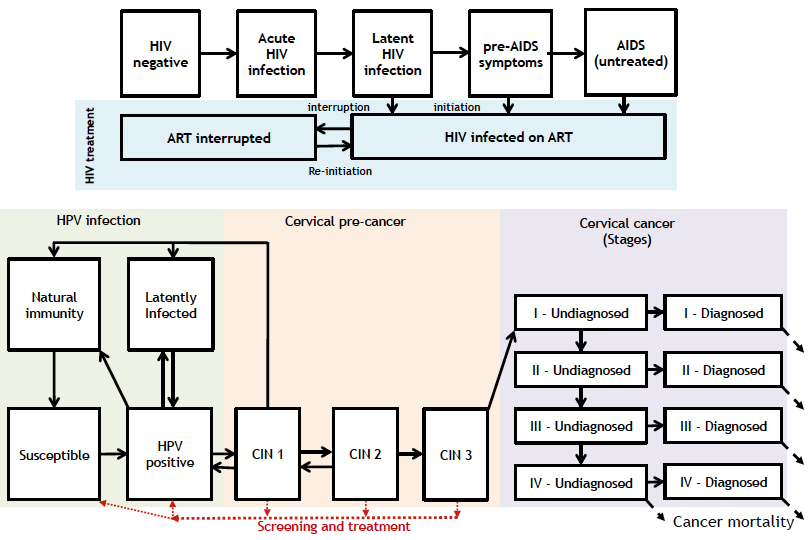
*

**Figure T2.1 –** The natural histories of HIV and HPV infection in MicroCOSM-HPV.

**HIV natural history:** HIV infection is introduced to the population in 1990, through infection in a small number of high-risk individuals (2,12). A newly infected individual will transition through the acute phase (average duration 3 months), the asymptomatic latent phase (average duration ~5 years), the symptomatic pre-AIDS phase (average duration ~5 years) and the AIDS phase (average duration 2 years) (Figure T2.1). The individual can initiate ART during any stage after the acute stage and can interrupt and restart treatment. ART initiation rates depend on time, sex and HIV stage, and ART interruption rates depend on time. Age and time dependent rates of fertility and mortality are influenced by HIV stage. Excess mortality for those on ART depends on the stage at ART initiation, and the duration on treatment.

**HPV and cervical cancer natural history:** Starting conditions for stage of HPV infections (all stages excluding cervical cancer stages in Figure T2.1) in 1985 are derived through an iterative process. Initially starting with crude estimates of prevalence and parameters, the model is run for a period of 40 years, assuming no changes in sexual behaviour and no HIV epidemic. The starting conditions are then updated with the prevalence in each stage at the end of the 40-year period. This process is repeated several times after each calibration step until equilibrium conditions are achieved.

Thirteen independent HR-HPV types are simulated individually (infection with one type does not influence infection, regression, or progression with any other type). A newly HPV infected woman with any HPV type will progress through stages of HPV infection (no cervical disease), three stages of pre-cancer (CIN1-3) and four stages of cervical cancer (Figure T2.1). HPV infections can spontaneously clear, and the individual can develop waning natural immunity or remain latently infected (which may reactivate at a later stage). It is possible to move between latent and active infection multiple times, and latent infections cannot progress to pre-cancer or contribute to transmission.

Early stages of pre-cancer can regress and upon regression some individuals will remain HPV infected, while others will acquire natural immunity or remain latently infected. HPV infected individuals are infectious through all stages of cervical disease. However, once one HPV type has progressed to cancer, progression or regression of other types do not occur. This implies that a woman will get cervical cancer only once and that this cancer is attributable to only one HPV type, even if multiple types are present. Women with cervical cancer can be diagnosed during any of the four cancer stages, and survival probabilities (derived from data of women diagnosed and treated at Groote Schuur Hospital in Cape Town) depend on the stage of diagnosis. Women who are not diagnosed, move through the stages of cancer, and die from Stage IV cancer after an average of 6 months in this stage. Parameters for the duration of cancer stages are shown in table T2.2. Survivors of cervical cancer are removed from the model simulation. Men move through the stages of susceptible to infection, active HPV infection, naturally immune and latently infected.

**Co-infections and effect of ART on HPV and diseases:** HPV infection and cervical disease has no impact on the natural history of HIV (5). However, HIV infection influences progression/regression rates of HPV infection and cervical disease: HPV durations are longer, rates of reactivation are higher, rates of progression to cancer are higher and rates of regression are lower (Table T2.3). ART use reduces risk of progression (and increases regression) compared to those not on ART, but not compared to HIV negative women. Women who start ART during the late stages of HIV experience the same rates of regression/progression as women not on ART for the first two years, and thereafter experience the same rates as women who started ART during the latent phases (Table T2.3).

1. **Baseline interventions – for HIV**

**Condom use:** Condom usage and frequency of sex acts depend on age, sex, and relationship type (short-term, long-term and sex worker-client). The proportion of sex acts protected by condoms increased as HIV prevalence increased and declined again in short-term relationships after the scale-up of ART.


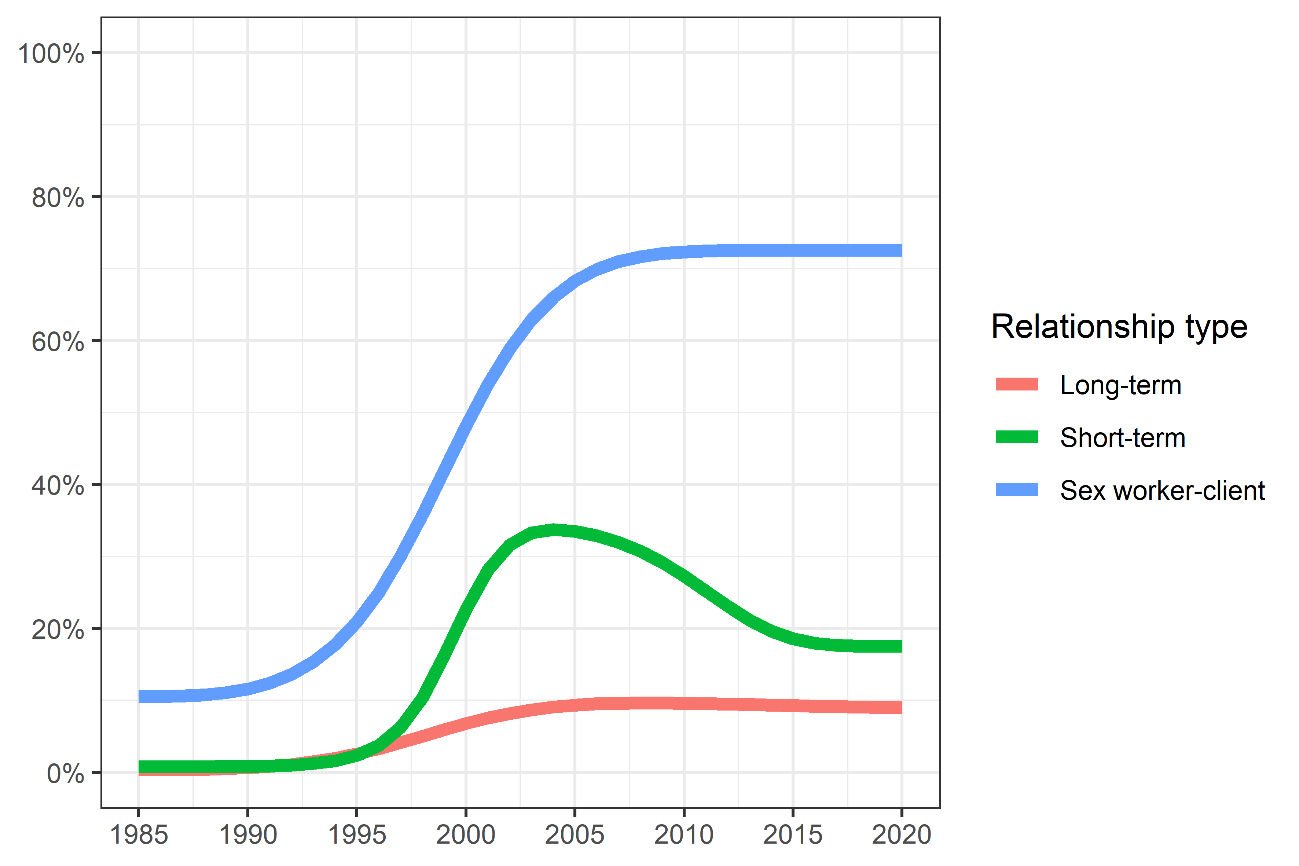


**Figure T2.2** – The proportion of sex acts protected by condoms among females aged 20-24 over time.

**Male circumcision:** *MicroCOSM-HPV* does not simulate male circumcision. Since traditional male circumcision has stayed constant at around 35% of males aged 15-49 over the years, the per-sex act transmission probability implicitly takes this into account. However, since around 2010 medical male circumcision has substantially increased leading to total circumcision prevalence of ~60% among 15-49-year-olds in 2020 (6). Although *MicroCOSM-HPV* matched HIV prevalence estimates well up to 2017 (Figures T2.4 and T2.6), this limitation may impact long-term predictions of HIV prevalence and in turn, cervical cancer incidence. We address this limitation by re-weighting the *MicroCOSM-HPV* population according to the population demographics of the *Thembisa* model, by age, sex, HIV and ART status (6). The *Thembisa* model simulates HIV prevention (including medical male circumcision) in much greater detail than *MicroCOSM-HPV* and provides more reliable long-term demographic estimates.

**HIV treatment:** ART initiation in the model is possible since 2000, stratified by sex and stage of infection. Rates of ART initiation are increased to reflect changes in South African HIV prevention policy – from 2004 people with CD4 count of ≤200 cells/μ could initiate treatment in the public sector. In 2011 the threshold was updated to ≤350 cells/μ and to ≤500 cells/μ in 2015. Universal access to treatment is available since 2016, but in the model, initiation rates among late-stage HIV remain higher than in the latent phase, and higher for females than males. Rates of ART initiation by sex and HIV stage (latent/late) were fitted to match ART coverage of the *Thembisa* model, which simulates HIV testing and treatment initiation in detail (13). Rates of ART interruption and resumption are based on a review of local data (6).

**4) Baseline interventions - for HPV and Cervical cancer**

**Cervical cancer screening:** *MicroCOSM-HPV* *basecase* scenario assumes that routine screening for cervical pre-cancer starts in 2000 (coinciding with the release of the first national screening policy (14)). Each woman has an age- and ART status-specific probability of entering the screening programme. This probability increases over time and stabilises in different years depending on ART status (women with HIV but not on ART has the same probabilities as HIV negative women, Figure T2.4). After the initial Pap smear, time to the next screen or treatment is drawn from Weibull distributions depending on the cytological result. To inform the probabilities of entering screening, we developed a separate, simpler simulation model and fitted this model to screening frequencies in the Western Cape (WC) province of South Africa, using individual-level Pap smear data from the Provincial Health Data Centre (PHDC) (15). Although the national screening policy states that HIV-negative women should be screened every 10 years (at ages 30, 40 and 50), and HIV-positive women should be screened 3 yearly after diagnosis (16), this schedule is not adhered to, and HIV-negative women are screened before age 30 and well after age 60. HIV-negative women younger than 30 have a small probability of entering the screening algorithm in the model, and all routine screening stops after the age of 60. Realistic distributions of time between visits (routine screening, follow-up after inadequate smear, follow-up after abnormal smear) were also estimated using the WC-PHDC data. We assume that Pap smear is the only screening method, and LLETZ treatment follows a positive colposcopy diagnosis. The screening algorithm in the model is illustrated in Figure T2.3. Pap smear has a 54% sensitivity of diagnosing CIN2+ correctly, and 98% specificity (17–19). Women with a cytological diagnosis of HSIL, or a second LSIL, are referred to colposcopy. The fraction who accesses colposcopy - and therefore pre-cancer treatment - depends on time and HIV/ART status. Around 54% of HIV-negative women who required colposcopy in 2017 visited these facilities within 2 years, and 44% of HIV-positive women (based on WC-PHDC data). All women with CIN1+ on colposcopy are treated (91% sensitivity (20,21)) and we assume that 75% of HIV-negative and 40% of HIV-positive women will be cleared of all abnormal lesions following LLETZ treatment (weighted averages from (22–27)). In all scenarios we assume that 15% of those treated successfully will not clear the HPV infection (28–30). The *basecase* screening and treatment algorithm in the model are fully described in the supplementary material of Van Schalkwyk et al. (1).

**HPV vaccination:** We assume that vaccinated women receive lifelong full protection against acquisition of vaccine HPV types, regardless of HIV or ART status at or after vaccination. If vaccines are administered to sexually active women who are not HPV-naïve, it does not influence the natural history of the HPV infections already present. Although South Africa has rolled-out a national vaccination programme in 2014, with consistent 2-dose coverage of around 60% among 9-year-old girls, our *basecase* assumption for this analysis is that there is no vaccination programme in place. This is to be consistent with previous analyses of the Cervical Cancer Elimination Modelling Consortium (31).


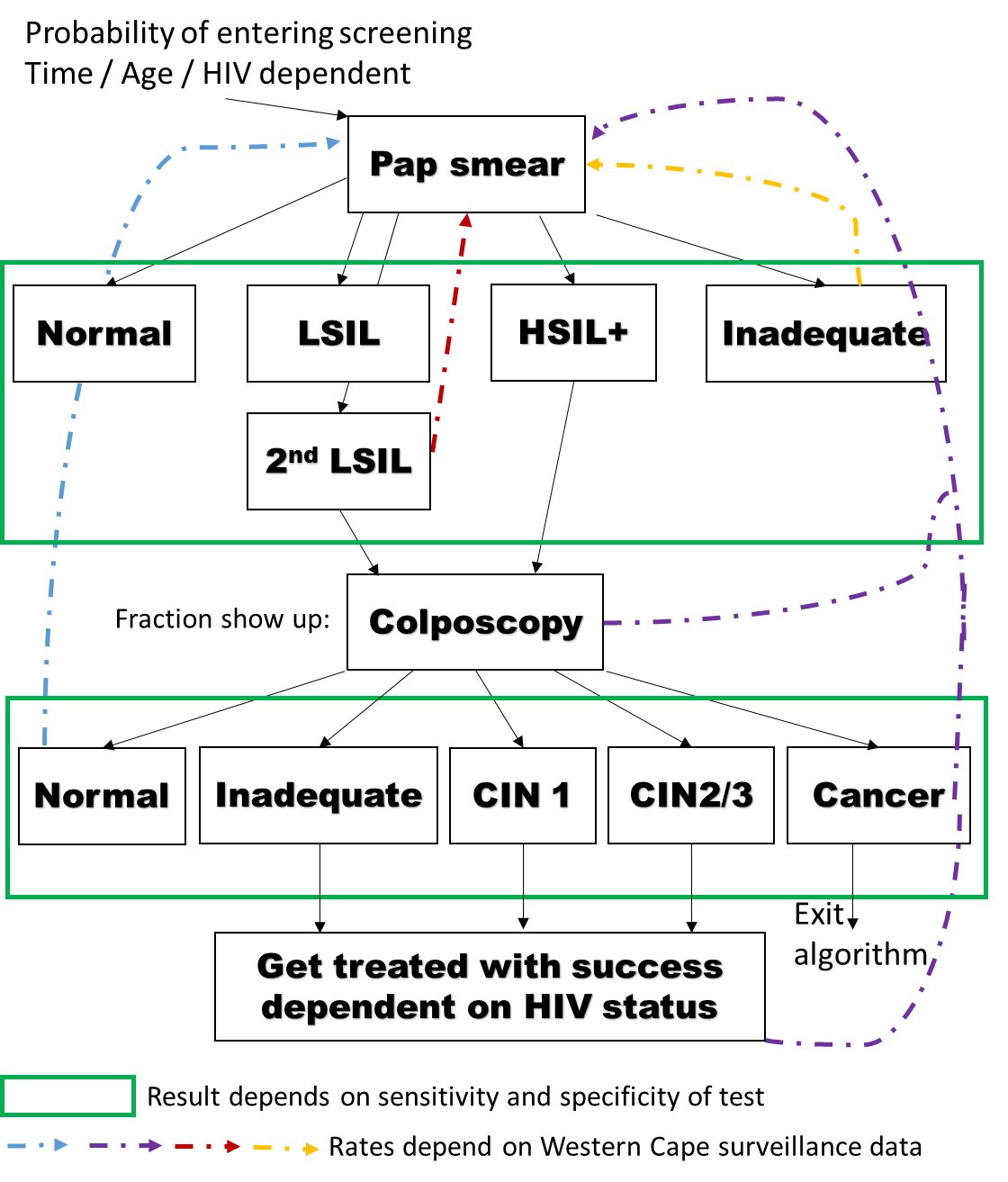


**Figure T2.3** – The screening algorithm in *MicroCOSM-HPV*


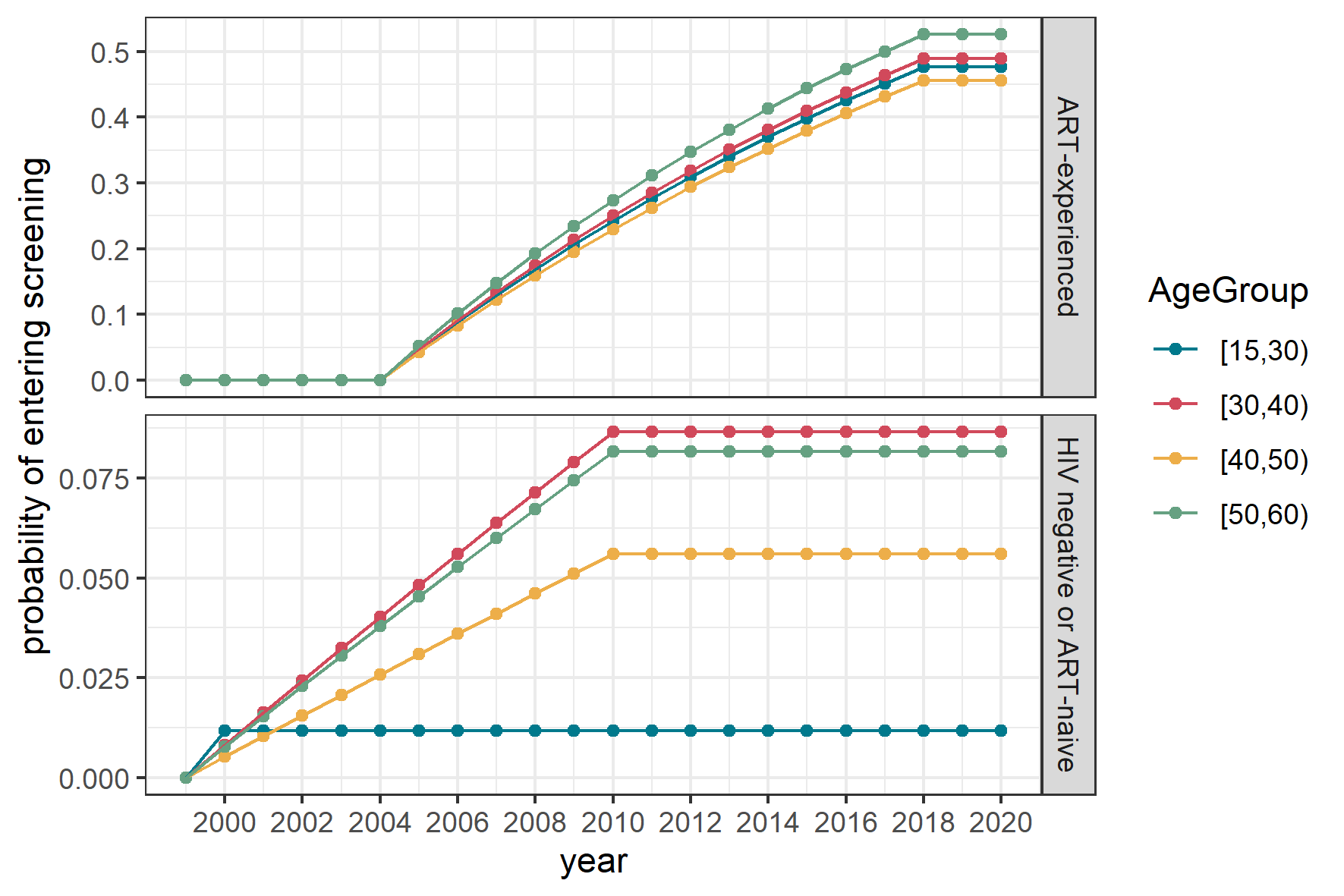


**Figure T2.4** – Yearly probabilities of entering the screening programme, by age and ART status.

**5) Model calibration**

In *MicroCOSM-HPV*, we use the medians of best-fitting parameter combinations for sexual behaviour and HIV parameters that were estimated in previous studies (2,9). We calibrate the HPV infection, cervical pre-cancer, and cervical cancer components in three steps, using the Bayesian sampling-importance-resampling approach (32).

First, all stages of cervical disease were collapsed into an ‘HPV infected’ stage and the main parameters that determine type-specific HPV prevalence were varied (HPV transmission probability per sex-act; durations of HPV infections, natural immunity, and latency; HIV/ART multipliers). Type-and sex- specific parameters were calibrated to HPV prevalence data from South African studies (table T2.5). In the second and third calibration steps, the parameters of all 13 HR-HPV types are fixed at the medians of the posterior distributions (4,5).

In the second step, we calibrate the parameters that determine pre-cancer disease progression (progression to and regression from CIN 1 and 2) using histological results obtained from biopsies performed during studies performed in South Africa (table T2.6), as well as the HPV prevalence data in table T2.5. We fit progression and regression rates for HPV16, HPV18 and all 11 other HR-HPV types combined, and some parameters are assumed to have the same values for all types (Table T2.4). In the third step, these parameters are kept fixed at the medians of the 100 best-fitting parameter combinations.

In the third step, we calibrate the parameters that determine progression to cervical cancer and diagnosis by using age-specific cervical cancer incidence as reported to the National Cancer Registry (NCR) between 2000 and 2016 (Table T2.8), as well as data on the fractions of women diagnosed in each stage of cancer in South Africa (Table T2.7). The NCR reports only on pathologically confirmed cancer, and we calibrate the parameters assuming different fractions of cases that receive no pathological confirmation. The assumption that 10% of cervical cancer cases receive only a clinical diagnosis resulted in the best fit to data, and this is our base case assumption.

Prior distributions for all parameters were derived from published literature (South African studies where possible) and other modelling studies. Likelihood functions account for the heterogeneity between prevalence estimates, since none of the studies were performed among nationally representative participants. A full description of the calibration methods, data sources used, and choices of prior distributions are given in the supplementary material of Van Schalkwyk et al. (1).

**6) Parameter estimates and fitting/validations outcomes and data sources**

**Table T2.1** - Best-fitting HIV model parameter values (2)

| **Parameter** | **Median (IQR)** |
| --- | --- |
| **Transmission probability per sex act** |  |
| **M-to-F, non-spousal** | 0.81% (0.69-0.93%) |
| **F-to-M, non-spousal** | 0.36% (0.31-0.4%) |
| **M-to-F, spousal** | 0.19% (0.14-0.24%) |
| **F-to-M, spousal** | 0.17% (0.13-0.21%) |
| **Relative infectiousness, acute HIV** | 19.3 (14.9-23.1) |
| **Relative infectiousness, AIDS** | 6.9 (5.71-8.15) |
| **Initial prevalence (1990) in high-risk women** | 2.31% (1.76-2.6%) |
| **Bias in self-reported condom use*** | 0.638 (0.537-0.773) |

* Parameter to allow for bias in reporting of condoms across all ages and relationship types (1 = no bias and values <1 indicated over-reporting)

**Table T2.2** – CC natural history parameters that were used as fixed values (not calibrated to data).

| **Parameter** | **Estimate** | **Source** |
| --- | --- | --- |
| **Proportion that clears HPV infection during CIN1 regression** | 0.9 | (33,34) |
| **Natural progression of cervical cancer (rate per year*)** |  | (35) |
| **Stage I to Stage II** | 0.225 |  |
| **Stage II to Stage III** | 0.3 |  |
| **Stage III to Stage IV** | 0.45 |  |
| **Weibull parameters for survival probability after cervical cancer diagnosis. Shape(scale)** |  | Data from Groote Schuur Hospital |
| **Stage I** | 0.61 (126.5) |  |
| **Stage II** | 0.67 (16.28) |  |
| **Stage III** | 0.56 (3.91) |  |
| **Stage IV** | 0.78 (0.53) |  |

* Annual rates converted to weekly probabilities in the simulation

**Table T2.3**- Type specific medians of the posterior distributions of the HPV infection and transmission parameters in the model

|  | **16** | **18** | **31** | **33** | **35** | **39** | **45** | **51** | **52** | **56** | **58** | **59** | **68** |
| --- | --- | --- | --- | --- | --- | --- | --- | --- | --- | --- | --- | --- | --- |
| **Transmission probability (per sex act)** | | | | | | | | | | | | | |
| **Male to Female** | 0.60 | 0.40 | 0.54 | 0.18 | 0.48 | 0.44 | 0.19 | 0.53 | 0.46 | 0.15 | 0.54 | 0.46 | 0.37 |
| **Female to Male** | 0.09 | 0.38 | 0.28 | 0.31 | 0.20 | 0.23 | 0.24 | 0.33 | 0.41 | 0.56 | 0.32 | 0.28 | 0.57 |
| **Average duration of HPV infection (in months) if HIV-negative (priors based on** (37–45) **for women and** (46) **for men)** | | | | | | | | | | | | | |
| **Males** | 12.1 | 5.6 | 3.3 | 3.3 | 5.2 | 4.6 | 5.3 | 8.0 | 6.8 | 3.2 | 4.7 | 7.6 | 9.6 |
| **Females** | 11.4 | 9.7 | 7.8 | 7.0 | 10.0 | 8.5 | 10.5 | 9.3 | 8.5 | 7.3 | 11.5 | 6.8 | 7.5 |
| **Relative HPV duration in HIV infection (priors based on** (35,36)**)** | | | | | | | | | | | | | |
| **Latent HIV vs HIV negative** | 1.3 | 1.3 | 1.3 | 1.3 | 1.4 | 1.3 | 1.4 | 1.3 | 1.4 | 1.4 | 1.3 | 1.4 | 1.4 |
| **Acute HIV/late HIV/recent ART* vs HIV negative** | 2.2 | 2.1 | 1.6 | 2 | 2 | 1.8 | 2.1 | 1.9 | 2.1 | 2.3 | 1.8 | 1.8 | 2.1 |
| **Average time to reactivation (in years) if HIV-negative** | | | | | | | | | | | | | |
| **Males** | 17.5 | 16.5 | 18.8 | 16.2 | 12.4 | 13.7 | 13.9 | 14.4 | 15.7 | 20.1 | 13.9 | 13.8 | 17.2 |
| **Females** | 19.4 | 18.5 | 20.8 | 17.7 | 19.8 | 17.2 | 17.9 | 17.6 | 18.8 | 18.5 | 19.1 | 18.2 | 19.5 |
| **Proportion who become latently infected after clearance** | 0.56 | 0.65 | 0.3 | 0.51 | 0.5 | 0.66 | 0.73 | 0.58 | 0.44 | 0.54 | 0.62 | 0.51 | 0.36 |
| **Relative HPV reactivation rate** | | | | | | | | | | | | | |
| **Latent HIV vs HIV-negative** | 2.4 | 2.1 | 2.3 | 2.2 | 2.4 | 1.8 | 2.5 | 2.0 | 2.5 | 2.7 | 2.0 | 1.9 | 2.7 |
| **Acute HIV/late HIV/recent ART* vs latent HIV** | 2.1 | 1.9 | 1.7 | 1.9 | 2.0 | 1.8 | 2.0 | 1.9 | 2.0 | 1.9 | 2.0 | 1.9 | 2.0 |
| **Average duration of immunity (in years)** | | | | | | | | | | | | | |
| **Males** | 16.6 | 10.5 | 9.1 | 9.0 | 6.3 | 9.5 | 10.1 | 9.9 | 11.5 | 11.2 | 8.0 | 8.4 | 11.9 |
| **Females** | 15.7 | 17.5 | 16.5 | 16.1 | 17.3 | 18.8 | 17.7 | 18.1 | 16.2 | 16.5 | 17.4 | 19.6 | 19.0 |

*Recent ART is defined as ART initiation within last 2 years. The same parameters are used for people who have been on ART for longer than two years and HIV negative people.

**Table T2.4** – Medians and 95% percentile intervals for 100 best fitting parameter combinations

| **Cervical pre-cancer parameters** | **Type 16** | **Type 18** | **Other HR-HPV** | **Source for prior distributions** |
| --- | --- | --- | --- | --- |
| **Multiplier for duration of HPV among women^+^**  **(applies to all type-specific durations in Table T2.3)** | 0.53  (0.23-0.94) | 0.64  (0.28-1.0) | 0.65  (0.43-0.95) |  |
| **Proportion that will progress from HPV infected to CIN1** | 0.23  (0.11-0.34) | 0.17  (0.08-0.3) | 0.15  (0.08-0.27) | (36,37) |
| **γ_1_ : Progression rate from CIN1 to CIN2 in HIV negative women*** | 0.15  (0.1-0.24) | 0.07 (0-0.17) | 0.11  (0.05-0.16) | (38) |
| **α_1_: Regression rate from CIN1 to Normal in HIV negative women *** | 0.66  (0.31-1.01) | 0.91  (0.43-1.64) | 1.02  (0.51-1.66) | (38) |
| **α_2_: Regression rate from CIN2 to CIN1 (<=30) in HIV negative women *** | 0.44  (0.38-0.52) | 0.67  (0.46-0.89) | 0.61  (0.43-0.9) | (39) |
| **Multiplier for regression (α_1_ and α_2_) in women older than 30** | 0.59 (0.46-0.73) | | | (39) |
| **m_1_: HIV multiplier for CIN1 progression (γ_1_)** | 2.54 (2.01-4.79) | | | (40) |
| **m_2_: ART multiplier for HIV progression multiplier (m_1_)** | 0.72 (0.57-0.88) | | | (41) |
| **m_3_: HIV multiplier for rate of CIN1/2 regression in HIV negative women (α_1_ and α_2_)** | 0.76 (0.61-0.82) | | | (40) |
| **m_4_: ART multiplier for HIV multiplier of rate of CIN1/2 regression (m_3_)** | 1.0 (0.97-1.0) | | | (41) |
| **Cervical cancer parameters** | **Type 16** | **Type 18** | **Other HR-HPV** | **Source for prior distributions** |
| **γ_2_: Progression rate from CIN2 to CIN3 (<=30)*** | 0.041 (0.024-0.072) | 0.015 (0.001-0.047) | 0.008 (0.005-0.013)) | (39) |
| **m_5_: Multiplier of γ_2_ for women aged 30-50** | 2.48 (2.04-2.93) | | |  |
| **m_6_: Multiplier of γ_2_ for women aged 50+** | 3.77 (2.5-5.38) | | | (42–45) |
| **HIV: multiplier for CIN2 (γ_2_) and cancer progression (Table T2.2)** | 1.21 (1.12-1.42) | | | (40) |
| **CIN3 duration: Weibull scale parameter (years)** | 16.45 (11.8-19.5) | | |  |
| **CIN3 duration: Weibull shape parameter** | 2.55 (2.1-2.9) | | |  |
| **Yearly probability of getting diagnosed in Stage I** | 0.023 (0.002-0.049) | | |  |
| **Yearly probability of getting diagnosed in Stage II** | 0.12 (0.06-0.19) | | |  |
| **Yearly probability of getting diagnosed in Stage III** | 0.61 (0.41-0.79) | | |  |
| **Yearly probability of getting diagnosed in Stage IV** | 0.93 (0.86-1) | | |  |

+HPV duration in Table T2.3 includes pre-cancer and cancer stages. During the second calibration step we separate HPV infection from pre-cancer and cancer, and total infection duration gets reduced to duration without pre-cancer.

*Annual rates are converted to weekly probabilities

**Table T2.5** - Type specific HPV prevalence used in fitting the HPV and cervical pre-cancer parameters.

|  | Study | HIV status | Date | Location | Sample  size | Prevalence | | | | | | | | | | | | |
| --- | --- | --- | --- | --- | --- | --- | --- | --- | --- | --- | --- | --- | --- | --- | --- | --- | --- | --- |
|  |  |  |  |  |  | **16** | **18** | **31** | **33** | **35** | **39** | **45** | **51** | **52** | **56** | **58** | **59** | **68** |
|  | General population data - females | | | | | | | | | | | | | | | | | |
| 1 | McDonald (46) | negative | 2000* | Khayelitsha | 8050 | 2.7% | 1.5% | 1.3% | 1.3% | 2.9% | 0.8% | 1.8% | 1.3% | 1.6% | 0.9% | 1.9% | 1.0% | 1.3% |
| 2 | McDonald (46) | infected | 2000* | Khayelitsha | 1371 | 8.2% | 6.2% | 4.1% | 4.3% | 8.5% | 3.7% | 5.7% | 5.1% | 5.4% | 3.7% | 7.9% | 3.3% | 6.2% |
| 3 | Giuliano (47) | negative | 2012 | Kraaifontein | 391 | 14.1% | 6.4% | 4.9% | 1.8% | 9.0% | 4.4% | 6.1% | 8.7% | 11.3% | 3.3% | 10.0% | 7.2% | 6.4% |
| 4 | Snyman (48) | not tested | 2011 | Tshwane | 253 | 5.7% | 4.9% |  |  |  |  |  |  |  |  |  |  |  |
| 5 | Snyman (49) | not tested | 2012 | Tshwane | 160 | 4.4% | 5.7% |  |  |  |  |  |  |  |  |  |  |  |
| 6 | Adler (50) | negative | 2013 | Masiphumelele | 50 | 6.0% | 4.0% | 0.0% | 0.0% | 2.0% | 4.0% | 0.0% | 6.0% | 4.0% | 0.0% | 2.0% | 2.0% | 6.0% |
| 7 | Adler (50) | infected | 2013 | Masiphumelele | 35 | 20.0% | 14.3% | 2.9% | 2.9% | 14.3% | 5.7% | 25.7% | 8.6% | 11.4% | 5.7% | 2.9% | 2.9% | 20.0% |
| 8 | Mbulawa (51) | negative | 2014 | Masiphumelele | 148 | 10.8% | 6.8% | 6.1% | 1.4% | 6.1% | 4.1% | 6.8% | 10.8% | 6.1% | 4.1% | 13.5% | 6.1% | 8.1% |
| 9 | Mbulawa (51) | negative | 2014 | Soweto | 143 | 12.6% | 8.4% | 2.1% | 1.4% | 7.7% | 4.9% | 1.4% | 7.0% | 6.3% | 1.4% | 7.0% | 7.0% | 6.3% |
| 10 | Mbulawa (52) | infected | 2006 | Gugulethu | 277 | 11.2% | 8.7% | 4.0% | 4.3% | 7.6% | 5.1% | 9.7% | 7.2% | 11.2% | 4.7% | 10.5% | 5.1% | 7.2% |
| 11 | Mbulawa (52) | negative | 2006 | Gugulethu | 207 | 3.4% | 2.4% | 2.9% | 1.4% | 4.8% | 2.9% | 0.5% | 1.4% | 3.9% | 0.5% | 4.3% | 2.4% | 2.4% |
| 12 | Denny (53) | infected | 2002 | Cape Town | 311 | 16.3% | 8.9% | 5.6% | 5.6% | 11.5% | 6.7% | 7.0% | 7.8% | 13.3% | 8.1% | 10.0% | 8.9% | 8.5% |
| 13 | Liebenberg (54) | negative | 2007 | KZN | 779 | 10.8% | 7.1% | 6.2% | 6.8% | 9.4% | 5.4% | 5.5% | 9.8% | 6.0% | 3.0% | 9.0% | 6.2% | 5.3% |
|  | General population data - males | | | | | | | | | | | | | | | | | |
| 14 | Vardas (55) | negative | 2005 | Soweto | 538 | 4.4% | 4.4% | 1.4% | 1.6% | 3.1% | 2.1% | 2.9% | 4.3% | 5.2% | 4.1% | 3.5% | 3.3% |  |
| 15 | Mbulawa (52) | infected | 2006 | Gugulethu | 277 | 13.3% | 7.0% | 3.8% | 3.2% | 9.5% | 7.0% | 15.2% | 9.5% | 7.6% | 2.5% | 10.1% | 11.4% | 9.5% |
| 16 | Mbulawa (52) | negative | 2006 | Gugulethu | 207 | 5.8% | 3.8% | 1.9% | 1.3% | 1.9% | 3.5% | 3.5% | 5.4% | 5.1% | 1.3% | 2.9% | 4.2% | 4.8% |
| 17 | Chikandiwa (56) | infected | 2015 | Johannesburg | 283 | 13.0% | 7.0% | 2.0% | 5.0% | 13.0% | 5.0% | 7.0% | 10.0% | 7.0% | 5.0% | 7.0% | 9.0% | 8.0% |
| ART clinics (initiating ART) | | |  |  |  |  |  |  |  |  |  |  |  |  |  |  |  |  |
| 18 | Moodley (57) | infected | 2007 | Cape Town | 109 | 13.8% | 15.6% | 5.5% | 8.3% | 4.6% | 10.1% | 15.6% | 12.8% | 9.2% | 5.5% | 17.4% | 7.3% | 11.0% |
| 19 | Firnhaber (58) | infected | 2009 | Johannesburg | 147 | 29.9% | 18.4% | 7.5% | 8.2% | 19.7% | 8.8% | 16.3% | 13.6% | 13.6% | 15.0% | 9.5% | 10.9% | 8.2% |
|  | Family planning clinics | | | | | | | | | | | | | | | | | |
| 20 | Mbulawa (59) | not tested | 2015 | 5 provinces | 330 | 7.0% | 6.1% | 2.1% | 1.2% | 4.8% | 6.7% | 7.6% | 6.7% | 3.0% | 3.0% | 7.6% | 4.8% | 3.0% |

*This is a combination of three studies performed in 1998-99, 2000-02 and 2004-06. The 2000-02 study enrolled the greatest proportion of the overall number of participants, so 2000 was assumed to best represent the timing of the combined prevalence estimate.

**Table T2.6** - Prevalence of cervical pre-cancer used in fitting pre-cancer parameters

| **Study** | **HIV status** | **Year** | **Location** | **Outcome** | **Age range** | **N** | **Prevalence** |
| --- | --- | --- | --- | --- | --- | --- | --- |
| **McDonald** (46) | infected | 2000 | Khayelitsha | CIN2+ | 17-30 | 512 | 9.6% |
| **McDonald** | infected | 2000 | Khayelitsha | CIN2+ | 30-40 | 582 | 10.3% |
| **McDonald** | infected | 2000 | Khayelitsha | CIN2+ | 40-65 | 277 | 6.5% |
| **McDonald** | negative | 2000 | Khayelitsha | CIN2+ | 17-25 | 884 | 2.5% |
| **McDonald** | negative | 2000 | Khayelitsha | CIN2+ | 25-30 | 662 | 2.1% |
| **McDonald** | negative | 2000 | Khayelitsha | CIN2+ | 30-35 | 666 | 3.6% |
| **McDonald** | negative | 2000 | Khayelitsha | CIN2+ | 35-40 | 2272 | 2.9% |
| **McDonald** | negative | 2000 | Khayelitsha | CIN2+ | 40-45 | 1400 | 3.1% |
| **McDonald** | negative | 2000 | Khayelitsha | CIN2+ | 45-50 | 982 | 3.1% |
| **McDonald** | negative | 2000 | Khayelitsha | CIN2+ | 50-55 | 617 | 2.1% |
| **McDonald** | negative | 2000 | Khayelitsha | CIN2+ | 55-65 | 567 | 1.4% |
| **Cronje** (60) | not tested | 2001 | Free State | CIN1+ | 21-65 | 1093 | 34.9% |
| **Cronje** | not tested | 2001 | Free State | CIN2+\|CIN1+* | 21-65 | 382 | 23.6% |
| **Denny** (61) | not tested | 1996 | Khayelitsha | CIN1+ | 35-65 | 2922 | 6.1% |
| **Denny** | not tested | 1996 | Khayelitsha | CIN2+\|CIN1+ | 35-65 | 178 | 46.6% |
| **Kuhn** (62) | negative | 2015 | Cape Town | CIN1+ | 30-65 | 378 | 12.7% |
| **Kuhn** | negative | 2015 | Cape Town | CIN2+\|CIN1+ | 30-65 | 48 | 41.7% |
| **Kuhn** | not on ART | 2015 | Cape Town | CIN1+ | 30-65 | 67 | 37.3% |
| **Kuhn** | not on ART | 2015 | Cape Town | CIN2+\|CIN1+ | 30-65 | 25 | 56.0% |
| **Kuhn** | on ART | 2015 | Cape Town | CIN1+ | 30-65 | 263 | 28.9% |
| **Kuhn** | on ART | 2015 | Cape Town | CIN2+\|CIN1+ | 30-65 | 76 | 55.3% |

*CIN2 or worse given any abnormality (CIN1+)

**Table T2.7** - The proportion of women diagnosed in each stage of cervical cancer (used for fitting cervical cancer parameters).

|  | **Year** | **N** | **Stage I** | **Stage II** | **Stage III** | **Stage IV** | **Sample** |
| --- | --- | --- | --- | --- | --- | --- | --- |
| **Groote Schuur Hospital**  **(GSH)** | 2000-2004 | 741 | 18.5% | 23.5% | 44.5% | 13.5% | All women treated in GSH in 2000-04 |
| **GSH** | 2005-2009 | 839 | 19.2% | 19.3% | 48.6% | 12.9% | All women treated in GSH in 2005-09 |
| **GSH** | 2010-2013 | 747 | 24.8% | 24.2% | 39.2% | 11.8% | All women treated in GSH in 2010-13 |
| **Lomalisa** (63) | 2000 | 836 | 8.4% | 35.6% | 41.0% | 15.0% | All women treated in Johannesburg hospital 1997-8 |
| **Mbodi** (64) | 2013 | 104 | 8.4% | 22.1% | 59.7% | 9.6% | Sample of women treated in Chris Hani Baragwanath hospital 2013 |
| **Snyman** (65) | 2011 | 85 | 7.0% | 17.6% | 58.9% | 16.5% | Sample of women treated in Kalafong hospital in 2011 |
| **Sabulei** (66) | 2015 | 153 | 5.2% | 44.4% | 49.7% | 0.7% | All women treated in an academic hospital in Gauteng in 2017 |

**Table T2.8** – Age-specific pathology diagnosed cervical cancer incidence (per 100,000 women) reported by the National Cancer Registry (67) (used in calibration of cervical cancer parameters). To calculate these incidence estimates, we used the number of cases reported by NCR as numerators and the *Thembisa* mid-year population estimates as denominators (6). During calibration, these numbers were inflated by 10% to account for the fraction of cases that do not receive a pathological confirmation of cancer.

| **Age** | **2000** | **2001** | **2002** | **2003** | **2004** | **2005** | **2006** | **2007** | **2008** | **2009** | **2010** | **2011** | **2012** | **2013** | **2014** | **2015** | **2016** |
| --- | --- | --- | --- | --- | --- | --- | --- | --- | --- | --- | --- | --- | --- | --- | --- | --- | --- |
| **20-24** | 1.2 | 1.3 | 1.1 | 1.0 | 0.7 | 0.7 | 1.1 | 0.9 | 1.0 | 1.1 | 1.0 | 0.3 | 0.2 | 0.7 | 0.5 | 1.1 | 1.0 |
| **25-29** | 5.5 | 5.4 | 4.0 | 5.0 | 4.7 | 5.1 | 5.6 | 4.5 | 5.7 | 6.6 | 6.2 | 4.8 | 3.1 | 5.7 | 4.3 | 5.2 | 4.9 |
| **30-34** | 14.1 | 14.6 | 14.8 | 11.5 | 12.8 | 13.5 | 17.8 | 16.3 | 16.8 | 17.5 | 19.7 | 15.2 | 13.6 | 17.3 | 16.0 | 18.7 | 20.2 |
| **35-39** | 26.8 | 28.0 | 25.4 | 23.9 | 26.9 | 26.8 | 29.6 | 26.9 | 32.4 | 30.7 | 31.2 | 30.4 | 28.6 | 34.6 | 34.9 | 37.0 | 42.9 |
| **40-44** | 43.9 | 40.5 | 43.0 | 36.4 | 37.8 | 39.4 | 41.9 | 40.2 | 41.3 | 41.6 | 44.4 | 40.9 | 45.2 | 41.4 | 43.7 | 54.6 | 58.0 |
| **45-49** | 51.7 | 56.5 | 51.1 | 54.8 | 48.9 | 49.1 | 48.1 | 47.1 | 48.6 | 50.6 | 49.4 | 48.6 | 52.2 | 49.8 | 49.1 | 59.9 | 61.5 |
| **50-54** | 68.6 | 71.5 | 68.0 | 59.5 | 59.7 | 63.0 | 61.6 | 59.4 | 55.3 | 57.9 | 57.2 | 50.4 | 59.9 | 55.1 | 53.3 | 60.9 | 73.5 |
| **55-59** | 77.4 | 71.2 | 73.0 | 67.1 | 67.3 | 74.4 | 69.0 | 66.1 | 63.9 | 64.8 | 64.0 | 56.5 | 67.6 | 62.7 | 59.0 | 69.6 | 71.8 |
| **60-64** | 85.7 | 90.5 | 91.2 | 79.2 | 82.0 | 72.4 | 77.7 | 71.1 | 73.4 | 68.1 | 67.8 | 56.6 | 72.6 | 61.1 | 62.6 | 70.2 | 71.2 |
| **65-69** | 81.7 | 81.9 | 77.8 | 74.3 | 70.3 | 66.8 | 70.4 | 71.4 | 75.7 | 76.6 | 67.4 | 65.2 | 66.6 | 63.4 | 60.2 | 74.9 | 80.7 |
| **70-74** | 82.1 | 74.7 | 78.4 | 61.8 | 75.6 | 78.3 | 75.4 | 66.7 | 70.6 | 58.2 | 64.3 | 63.5 | 78.7 | 69.3 | 58.5 | 71.1 | 72.3 |
| **75+** | 58.1 | 47.5 | 58.7 | 52.2 | 57.3 | 53.8 | 52.3 | 54.8 | 56.5 | 59.0 | 57.5 | 38.6 | 66.5 | 54.9 | 50.2 | 60.0 | 58.0 |

**7) Description of analysis**

We project cervical cancer incidence for the next century assuming that sexual behaviour parameters will stay constant, and that fertility and mortality rates will decline to reach an equilibrium (6). *MicroCOSM-HPV*’s age-, HIV- and ART- specific cervical cancer incidence rates are reweighted according to the average age-, HIV- and ART- specific demographics of the *Thembisa* model. This model simulates the population more realistically (international migration is considered) and simulates the HIV epidemic and prevention of HIV in more detail. In the *basecase* and other scale up scenario (unless specified in sensitivity analysis), we assume that the UNAIDS 90-90-90 targets will be met by 2030, which involved decreasing rates of ART interruption to increase the fraction of people living with HIV who are on ART to 90% by 2030. The default assumptions result in more than 90% of PLHIV diagnosed, more than 90% viral suppression and male circumcision prevalence of more than 70% by 2030. We calculate age-standardised cervical cancer incidence over time since 2020 using UNDP 2015 standard population for each of the 100 best-fitting cervical cancer parameter combinations and calculate medians.

In our *basecase* screening algorithm, women can be screened between the ages of 15 and 30 (at very low rates) and up to the ages of 60 (Figure T2.4). To switch to the once/twice in a lifetime screening after 2020 (scale-up scenarios), we had to phase out baseline screening to avoid seeing initial increases in cancer among those age groups not receiving Pap smear screening any more, and not receiving HPV-DNA based screening. Instead of screening once at ages 35 and 45 exactly, we assume one screen between the ages of 30 and 40, and one between 40 and 50 according to the following steps:

**Once/twice in a lifetime screening with HPV DNA test:** The once in a lifetime scenario suggests one screen for all women aged 35 (and one screen for all women aged 45 in the case of two lifetime screens). We assume that one of these two HPV-DNA screens can happen at any time between ages 30 and 40 and the other one at any time between ages 40 and 50. Women younger than 30 can still enrol in Pap smear screening at the same rates as in the *basecase* scenario but will not receive any Pap smears after they get to 30 years old. Women aged 50-60 can still receive Pap smear screening at the same rate as in the *basecase* scenario, but this rate linearly decreases to zero over ten years, by which time all women who had the opportunity to receive an HPV-DNA test will be aged 50-60. For the scenarios with only one lifetime screen aged 30-40, women older than 40 will be screened with Pap at rates that eventually reduce to zero over time. Initial rates of HPV-DNA screening (at and after implementation in 2020) are chosen such that coverage of screening among women aged 30-50 (by ART status) does not reduce after 2020, and these rates linearly increase to 70% coverage in 2030 and 90% in 2045.

**Extra screening for women with HIV:** These scenarios suggest one screen for HIV negative women aged 35; one screen for HIV negative women aged 45 and three-yearly screens for women with HIV aged 25 to 50. Our assumptions for HIV negative women are the same as above. Women with HIV aged between 15 and 25 can receive Pap smear screening at the same rates as the *basecase* scenario but will not receive any Pap smears after 25. women with HIV aged 50-60 can still receive Pap smear screening at the same rate as the *basecase* scenario, but this rate linearly decreases to zero over ten years, by which time all women who had the opportunity to receive an HPV-DNA test will be aged 50-60.

**8) Fitting and validation results**

8.1) HIV epidemiology


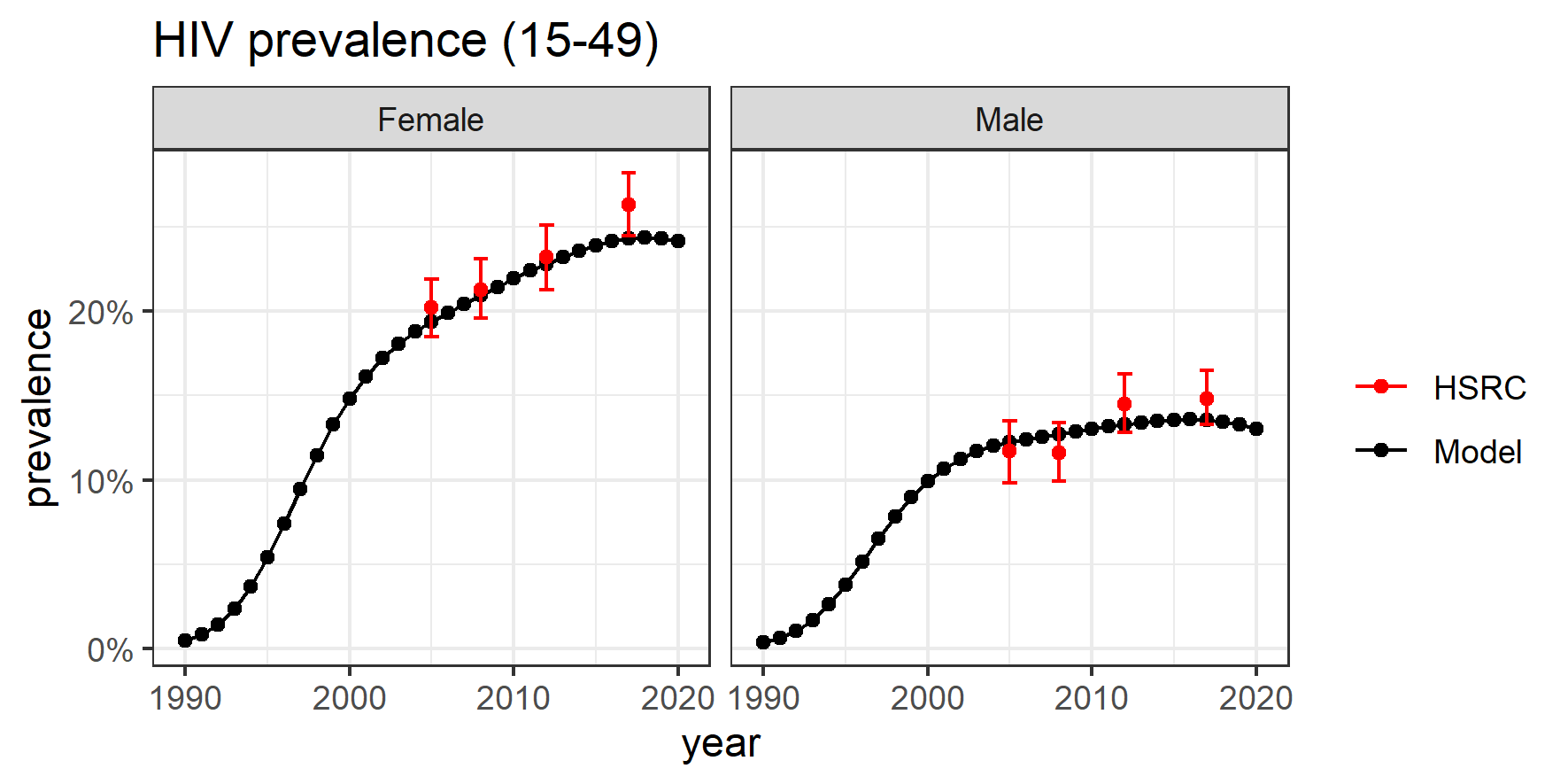


**Figure T2.5**: HIV prevalence by sex for adults aged 15-49. Data from nationally representative studies performed by the Human Sciences Research Council (HSRC) in red, and model estimates in black.


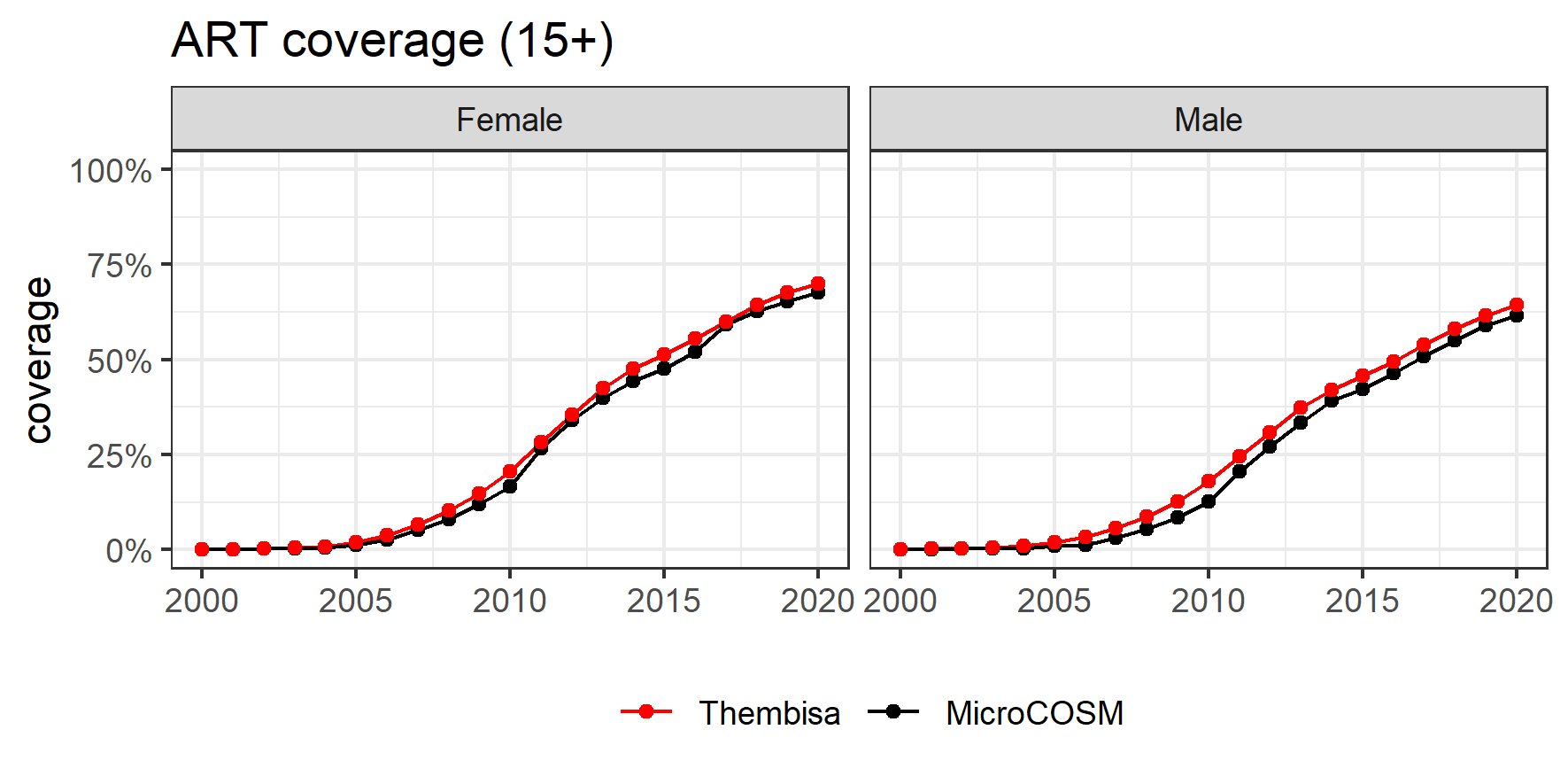


**Figure T2.6**: ART coverage among adults, by sex. Estimates from the *Thembisa* model in red, model estimates in black.


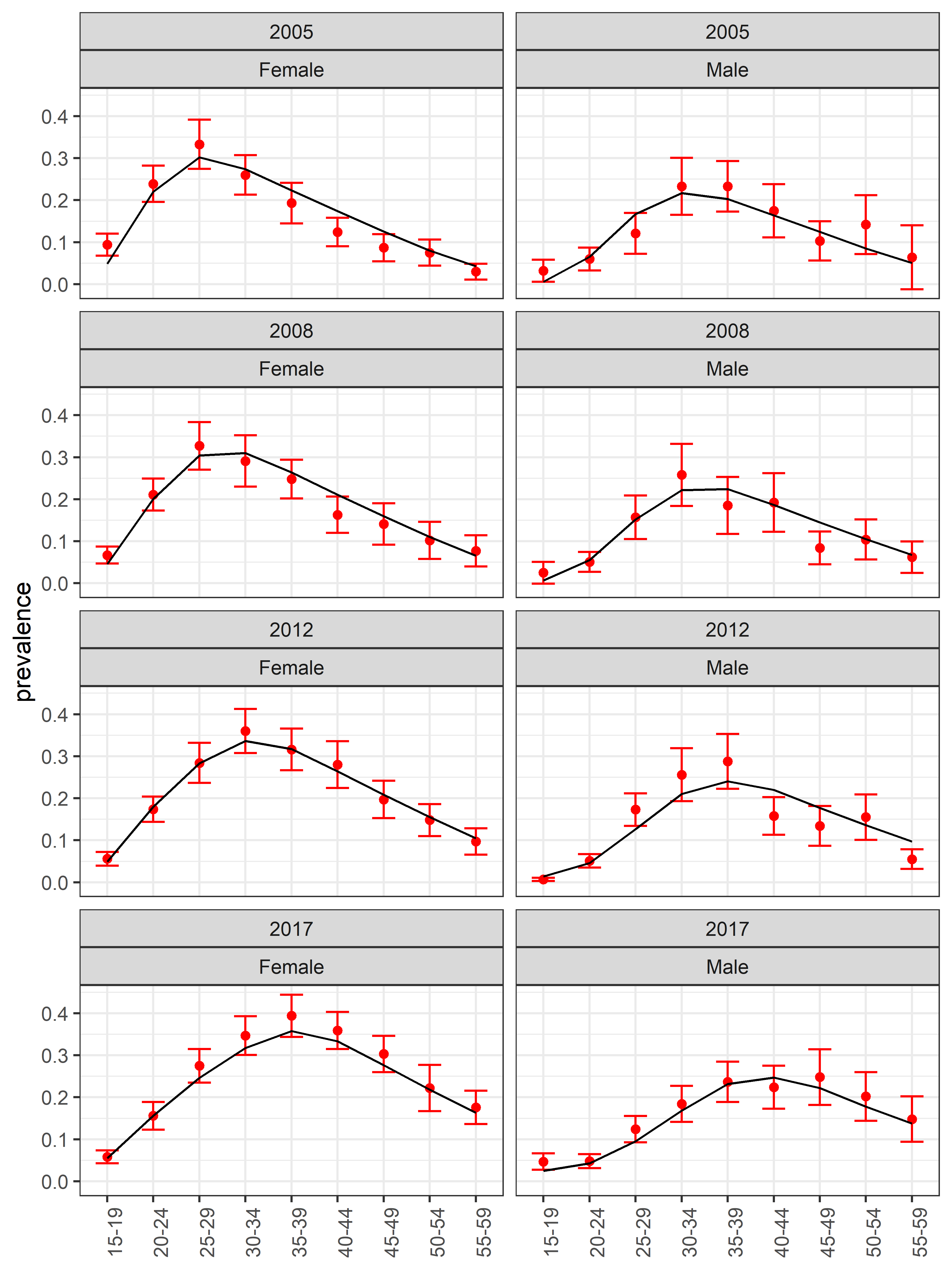


**Figure T2.7**: HIV prevalence by age, sex, and time. HSRC estimates in red, model estimates in black.

8.2) HPV and cervical cancer epidemiology


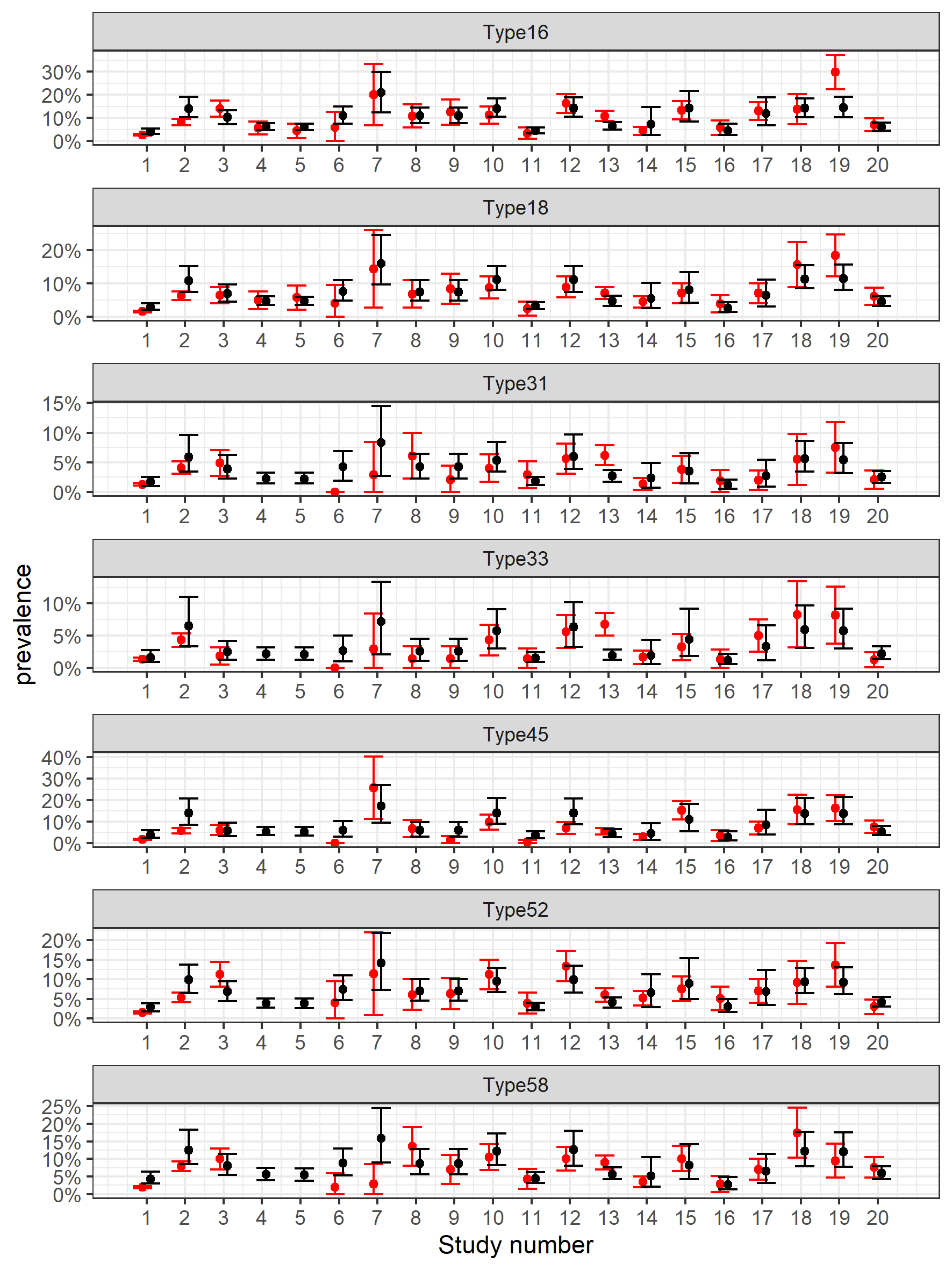


**Figure T2.8**: Vaccine type HPV prevalence. Each study number corresponds to the number in the first column of Table T2.5. The data is shown in red, and the mean and 95% percentile interval of the model estimates are shown in black.


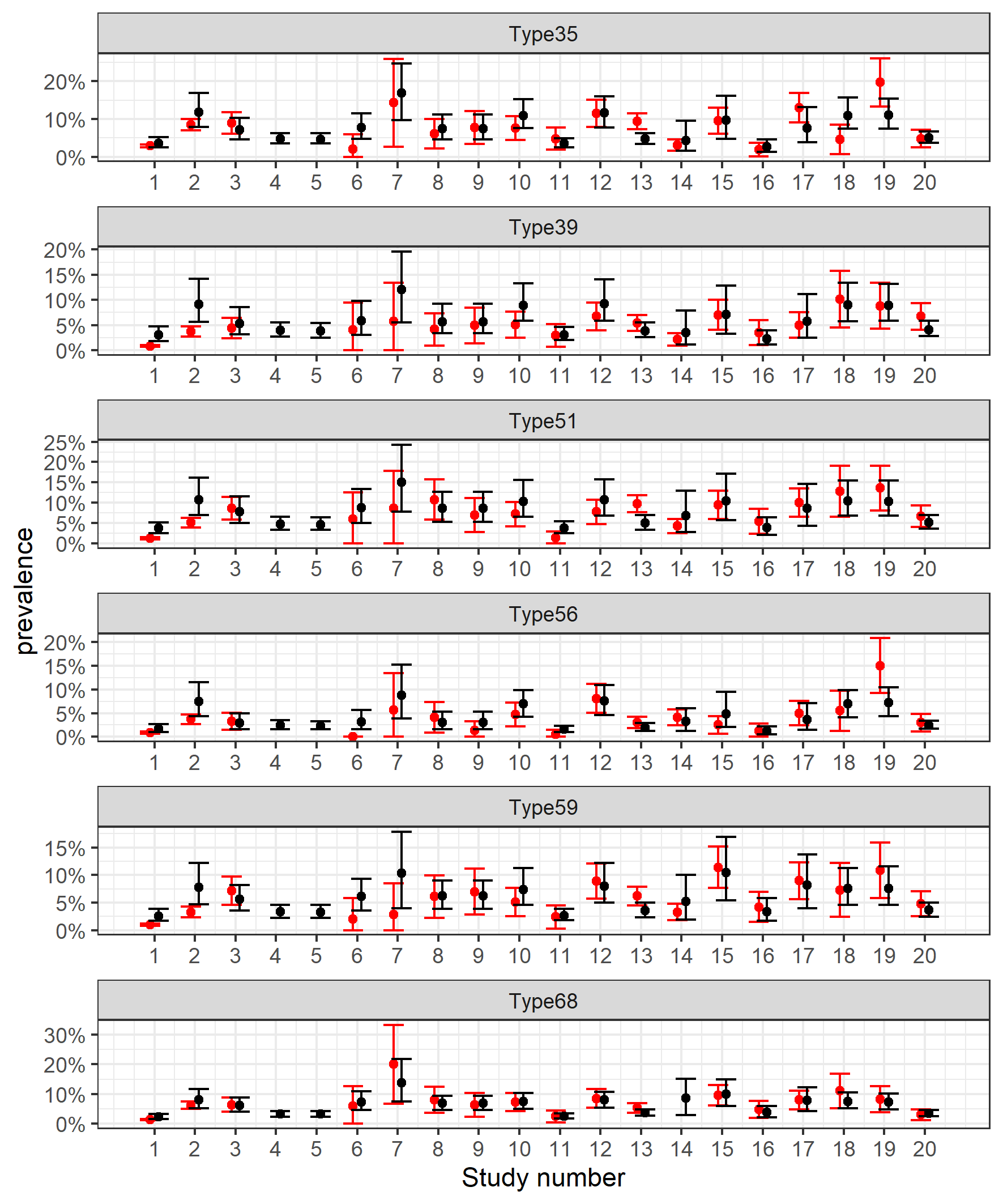


**Figure T2.9** -- Non-vaccine type HPV prevalence. Each study number corresponds to the number in the first column of Table T2.5. The data is shown in red, and the mean and 95% percentile interval of the model estimates are shown in black.

**Table T2.9** – Model fits to cervical pre-cancer data (Table T2.6)

| **Study** | **HIV** | **Measure** | **Ages** | **Sample size** | **Observed prevalence** | **Model prevalence** |
| --- | --- | --- | --- | --- | --- | --- |
| Cronje (60) | Not tested | CIN1+ | 21-65 | 1093 | 34.9 (32.1-37.8) | 15.2 (12.2-18.2) |
| Cronje | Not tested | CIN2+\|CIN1+* | 21-65 | 382 | 23.6 (19.3-27.8) | 27.6 (23.2-35.5) |
| Denny (61) | Not tested | CIN1+ | 35-65 | 2922 | 6.1 (5.2-7) | 11.2 (8.9-13.4) |
| Denny | Not tested | CIN2+\|CIN1+ | 35-65 | 178 | 46.6 (39.3-54) | 24.3 (19.5-32.1) |
| Kuhn (62) | Negative | CIN1+ | 30-65 | 378 | 12.7 (9.3-16.1) | 8.5 (6.5-10.6) |
| Kuhn | Negative | CIN2+\|CIN1+ | 30-65 | 48 | 41.7 (27.7-55.6) | 34.1 (27.2-40.7) |
| Kuhn | No ART | CIN1+ | 30-65 | 67 | 37.3 (25.7-48.9) | 41.1 (32.7-49) |
| Kuhn | No ART | CIN2+\|CIN1+ | 30-65 | 25 | 56 (36.5-75.5) | 39.8 (33.5-47) |
| Kuhn | On ART | CIN1+ | 30-65 | 263 | 29 (23.4-34.4) | 34.5 (26.7-43.8) |
| Kuhn | On ART | CIN2+\|CIN1+ | 30-65 | 76 | 55 (44.1-66.5) | 43.7 (35.7-51.6) |

*CIN2 or worse given any abnormality (CIN1+)


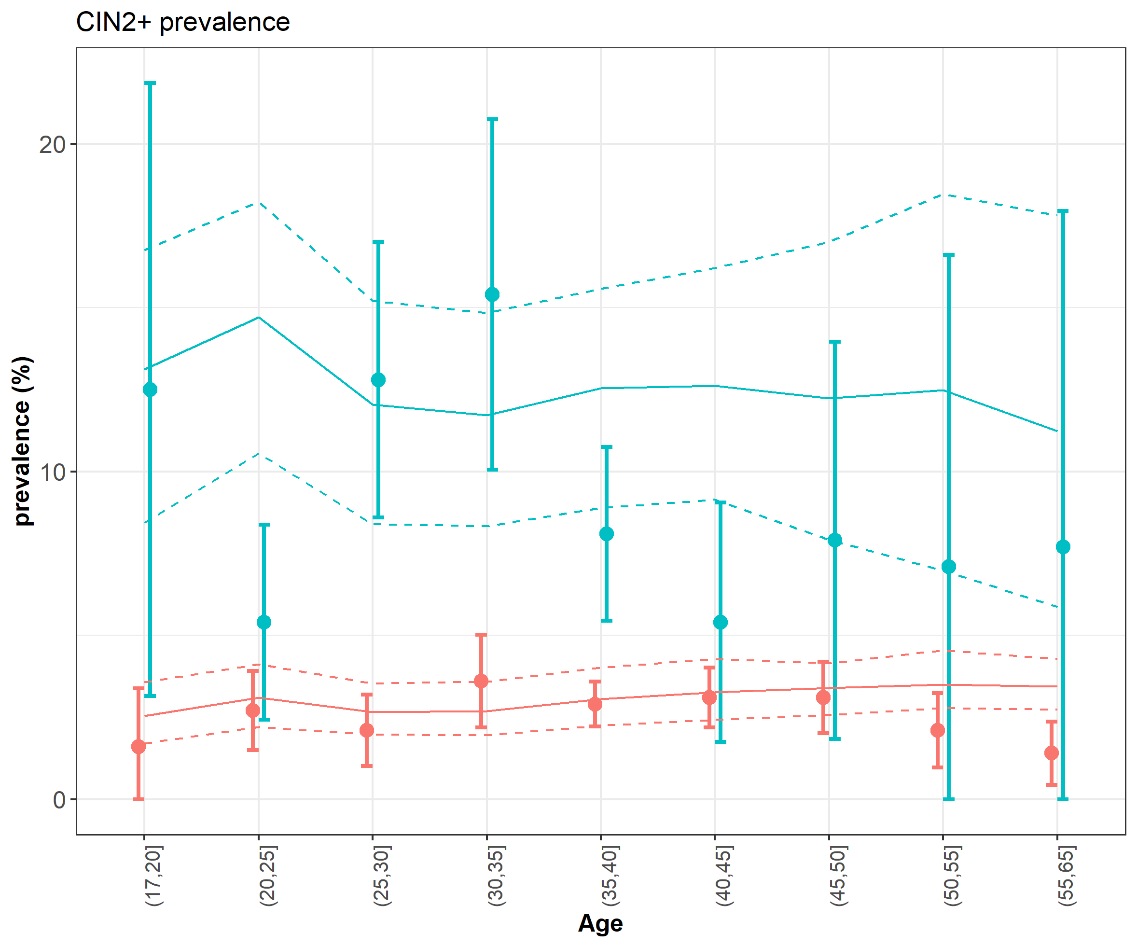


**Figure T2.10** – CIN2+ prevalence by age and HIV status from McDonald et al. (46) (Table T2.6) (red=HIV negative and blue=women with HIV; point and error bars=data, lines=mean model estimates and 95% percentile intervals)


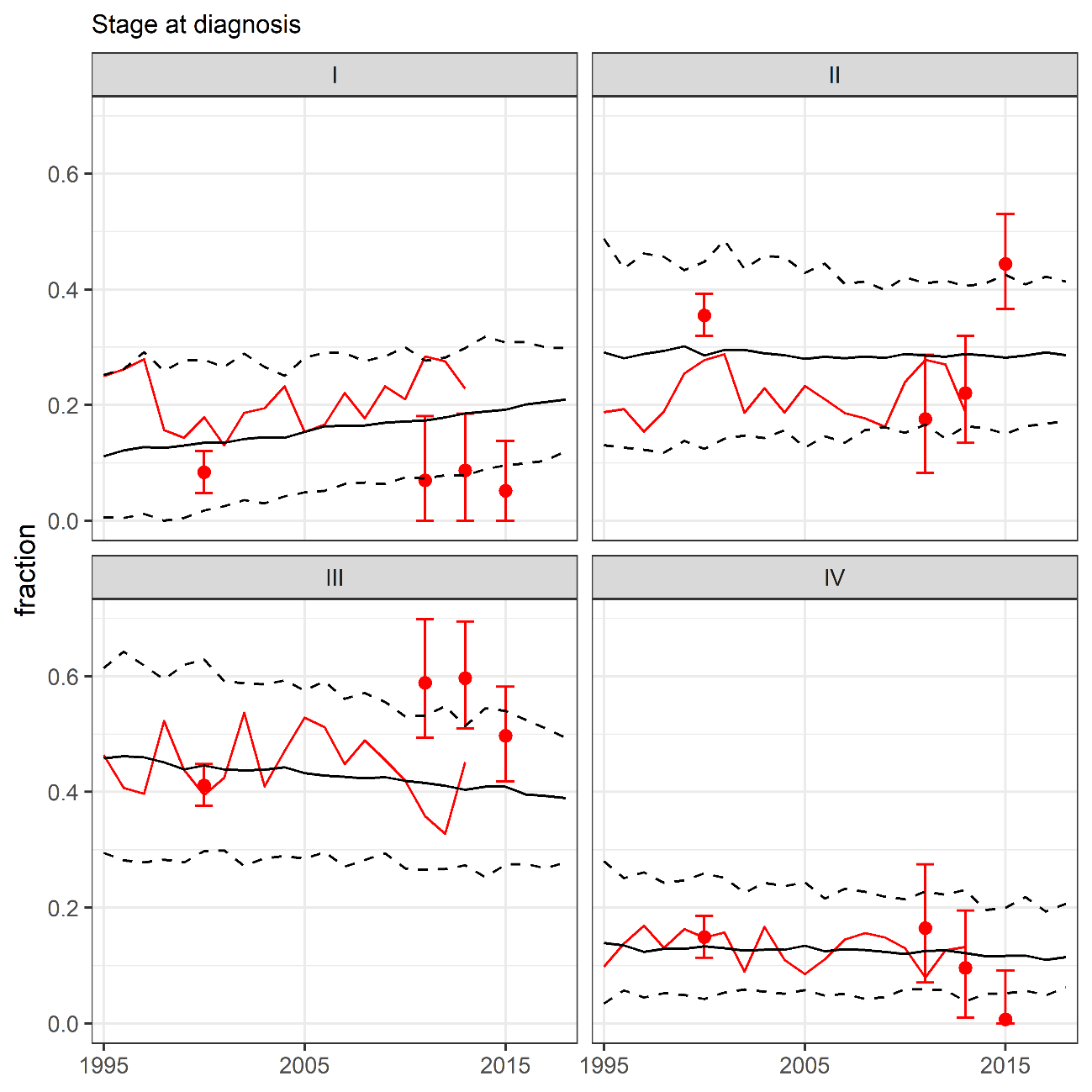


**Figure T2.11** – The fraction of cervical cancer cases diagnosed in each stage of the disease (Table T2.7). Estimates are from Groote Schuur Hospital (red lines), studies (red dots, (64–66,68)) and the 100 best fitting parameter combinations (black lines show mean of 100 estimates, dashed lines show 95% percentile intervals).


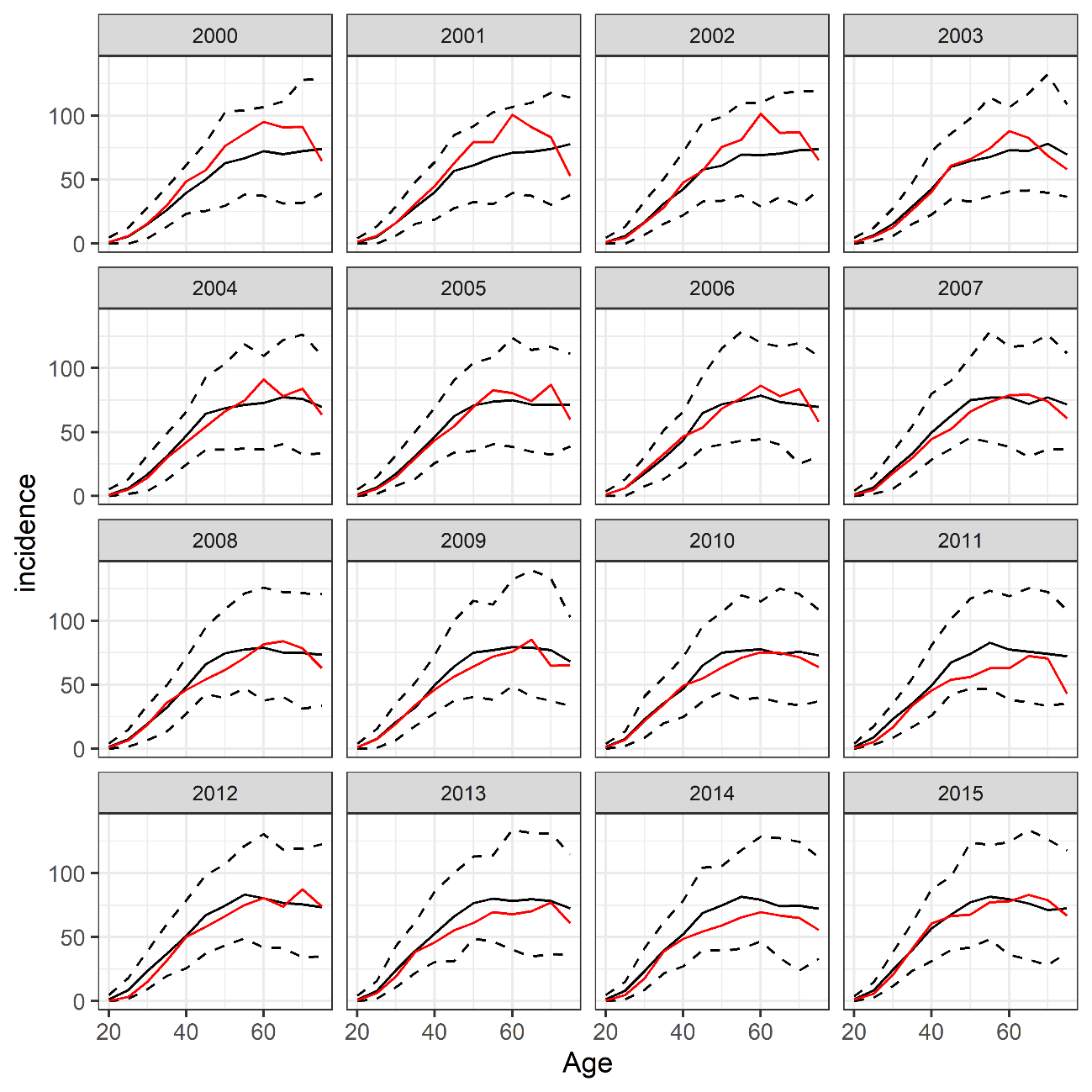


**Figure T2.11** – Age specific diagnosed cervical cancer incidence per 100,000 women as calculated from NCR data (red lines, Table T2.8) and the 100 best fitting parameter combinations (black lines show mean of 100 estimates, dashed lines show 95% percentile intervals).

**9) Model equations**

**See reference 1:** van Schalkwyk C, Moodley J, Welte A, Johnson LF. Modelling the impact of prevention strategies on cervical cancer incidence in South Africa. Int J cancer. 2021 Oct 15;149(8):1564–75.

**10) References**

1. van Schalkwyk C, Moodley J, Welte A, Johnson LF. Modelling the impact of prevention strategies on cervical cancer incidence in South Africa. Int J cancer. 2021 Oct 15;149(8):1564–75.

2. Johnson LF, Geffen N. A Comparison of Two Mathematical Modeling Frameworks for Evaluating Sexually Transmitted Infection Epidemiology. Sex Transm Dis. 2016 Mar;43(3):139–46.

3. Johnson LF, Geffen N. A comparison of microsimulation and deterministic approaches to modelling of sexually transmitted infection dynamics. In: STI and AIDS World Congress. Vienna, Austria; 2013.

4. Van Schalkwyk C, Moodley J, Welte A, Johnson LF. Estimated impact of human papillomavirus vaccines on infection burden: The effect of structural assumptions. Vaccine [Internet]. 2019 Aug;37(36):5460–5. Available from: https://doi.org/10.1016/j.vaccine.2019.06.013

5. Van Schalkwyk C, Moodley J, Welte A, Johnson LF. Are associations between HIV and human papillomavirus transmission due to behavioural confounding or biological effects? Sex Transm Infect. 2019;95(2).

6. Johnson L, Dorrington R. Thembisa version 4.2: A model for evaluating the impact of HIV / AIDS in South Africa [Internet]. 2019 [cited 2020 Jul 20]. Available from: www.thembisa.org

7. South African Department of Health, South African National AIDS Council. South African HIV and TB Investment Case - Summary Report Phase 1 [Internet]. 2016 [cited 2020 Jul 2]. Available from: http://www.heroza.org/wp-content/uploads/2016/03/SA-HIV_TB-Investment-Case-Full-Report-Low-Res.pdf

8. Marsh K, Eaton JW, Mahy M, Sabin K, Autenrieth CS, Wanyeki I, et al. Global, regional and country-level 90-90-90 estimates for 2018: Assessing progress towards the 2020 target. AIDS. 2019;33(April 2019):S213–26.

9. Johnson LF, Dorrington RE, Bradshaw D, Pillay-Van Wyk V, Rehle TM. Sexual behaviour patterns in South Africa and their association with the spread of HIV: Insights from a mathematical model. Demogr Res. 2009;21:289–340.

10. Winer RL, Hughes JP, Feng Q, O’Reilly S, Kiviat NB, Holmes KK, et al. Condom use and the risk of genital human papillomavirus infection in young women. N Engl J Med. 2006;354:2645–54.

11. Albero G, Castellsagué X, Giuliano AR, Bosch FX. Male circumcision and genital human papillomavirus: A systematic review and meta-analysis. Sex Transm Dis. 2012;39(2):104–13.

12. Johnson L, Kubjane M, Moolla H. MicroCOSM: a model of social and structural drivers of HIV and interventions to reduce HIV incidence in high-risk populations in South Africa. bioRxiv. 2018;310763.

13. Johnson LF, Rehle TM, Jooste S, Bekker L-G. Rates of HIV testing and diagnosis in South Africa: successes and challenges. AIDS. 2015 Jul;29(11):1401–9.

14. South African Department of Health. National Guideline for Cervical Cancer Screening Programme [Internet]. 2000 [cited 2019 Nov 27]. Available from: http://www.kznhealth.gov.za/cervicalcancer.pdf

15. Boulle A, Heekes A, Tiffin N, Smith M, Mutemaringa T, Zinyakatira N, et al. Data Centre Profile: The Provincial Health Data Centre of the Western Cape Province, South Africa. Int J Popul data Sci. 2019 Nov 20;4(2):1143.

16. South African Department of Health. Clinical guidelines for the management of HIV and AIDS in adults and adolescents. 2010;42.

17. Denny L, Kuhn L, Pollack A, Wainwright H, Wright TC. Evaluation of Alternative Methods of Cervical Cancer Screening for Resource-Poor Settings. Am Cancer Soc. 2000;89(4):826–33.

18. Wright TC, Denny L, Kuhn L, Pollack A, Lorincz A. HPV DNA testing of self-collected vaginal samples compared with cytologic screening to detect cervical cancer. JAMA. 2000;283(1):81–6.

19. Taylor S, Kuhn L, Dupree W, Denny L, De Souza M, Wright TC. Direct comparison of liquid-based and conventional cytology in a South African screening trial. Int J Cancer. 2006;962(118):957–62.

20. Mitchell MF, Schottenfeld D, Tortolero-Luna G, Cantor SB, Richards-Kortum R. Colposcopy for the diagnosis of squamous intraepithelial lesions: A meta-analysis. Vol. 91, Obstetrics and Gynecology. 1998. p. 626–31.

21. Cantor SB, Cárdenas-Turanzas M, Cox DD, Atkinson EN, Nogueras-Gonzalez GM, Beck JR, et al. Accuracy of colposcopy in the diagnostic setting compared with the screening setting. Obstet Gynecol. 2008;111(1):7–14.

22. Adam Y, van Gelderen CJ, de Bruyn G, McIntyre J a, Turton D a, Martinson N a. Predictors of persistent cytologic abnormalities after treatment of cervical intraepithelial neoplasia in Soweto, South Africa: a cohort study in a HIV high prevalence population. BMC Cancer. 2008;8:211.

23. Zeier MD, Botha MH, van der Merwe FH, Eshun-Wilson I, van Schalkwyk M, la Grange M, et al. Progression and Persistence of Low-Grade Cervical Squamous Intraepithelial Lesions in Women Living With Human Immunodeficiency Virus. Vol. 16, Journal of Lower Genital Tract Disease. 2012. p. 243–50.

24. Batra P, Kuhn L, Denny L. Utilisation and outcomes of cervical cancer prevention services among HIV-infected women in Cape Town. South African Med J. 2010;100(1).

25. Noël CJ. Excision margins in Human Immunodeficiency Virus seropositive women undergoing Large Loop Excision of the Transformation Zone for cervical dysplasia. University of the Witwatersrand; 2015.

26. Kabir F, Gelderen C Van, McIntyre J, Michelow P, Turton D, Adam Y. Cervical intra-epithelial neoplasia in HIV-positive women after excision of the transformation zone – does the grade change ? South African Med J. 2012;102(9):757–60.

27. Smith JS, Sanusi B, Swarts A, Faesen M, Levin S, Goeieman B, et al. A randomized clinical trial comparing cervical dysplasia treatment with cryotherapy vs loop electrosurgical excision precedure in HIV-seropositive women from Johannesburg, South Africa. Am J Obstet Gynecol. 2017;217(2):183.e1-183.e11.

28. Kreimer AR, Katki H a., Schiffman M, Wheeler CM, Castle PE. Viral determinants of human papillomavirus persistence following loop electrical excision procedure treatment for cervical intraepithelial neoplasia grade 2 or 3. Cancer Epidemiol Biomarkers Prev. 2007;16(January):11–6.

29. Kocken M, Helmerhorst TJM, Berkhof J, Louwers JA, Bais AG, Hogewoning CJA, et al. Risk of recurrent high-grade cervical intraepithelial neoplasia after successful treatment: a long-term multi-cohort study. Lancet Oncol. 2011;12:441–50.

30. Paraskevaidis E, Arbyn M, Sotiriadis A, Diakomanolis E, Martin-hirsch P, Koliopoulos G, et al. The role of HPV DNA testing in the follow-up period after treatment for CIN: a systematic review of the literature. Cancer Treat Rev. 2004;30:205–11.

31. Brisson M, Kim JJ, Canfell K, Drolet M, Gingras G, Burger EA, et al. Impact of HPV vaccination and cervical screening on cervical cancer elimination: a comparative modelling analysis in 78 low-income and lower-middle-income countries. Lancet. 2020;395(10224):575–90.

32. Smith AFM, Gelfand AE. Bayesian statistics without tears: a sampling–resampling perspective. Am Stat. 1992;46(2):84–8.

33. Nobbenhuis MAE, Helmerhorst TJM, Van Den Brule AJC, Rozendaal L, Voorhorst FJ, Bezemer PD, et al. Cytological regression and clearance of high-risk human papillomavirus in women with an abnormal cervical smear. Lancet. 2001;358(9295):1782–3.

34. Schiffman M, Wheeler CM, Castle PE. Human Papillomavirus DNA Remains Detectable Longer than Related Cervical Cytologic Abnormalities. J Infect Dis. 2002;186(8):1169–72.

35. Myers ER, McCrory DC, Nanda K, Bastian L, Matchar DB. Mathematical model for the natural history of human papillomavirus infection and cervical carcinogenesis. Am J Epidemiol. 2000 Jun 15;151(12):1158–71.

36. Insinga RP, Dasbach EJ, Elbasha EH, Liaw K-L, Barr E. Incidence and duration of cervical human papillomavirus 6, 11, 16, and 18 infections in young women: an evaluation from multiple analytic perspectives. Cancer Epidemiol biomarkers Prev. 2007 Apr;16(4):709–15.

37. Insinga RP, Perez G, Wheeler CM, Koutsky LA, Garland SM, Leodolter S, et al. Incident Cervical HPV Infections in Young Women: Transition Probabilities for CIN and Infection Clearance. Cancer Epidemiol biomarkers Prev. 2011;(11):287–97.

38. Liu M, Yan X, Zhang M, Li X, Li S, Jing M. Influence of Human Papillomavirus Infection on the Natural History of Cervical Intraepithelial Neoplasia 1: A Meta-Analysis. Biomed Res Int. 2017;2017:1–9.

39. Tainio K, Athanasiou A, Tikkinen KAO, Aaltonen R, Cárdenas J, Hernándes, et al. Clinical course of untreated cervical intraepithelial neoplasia grade 2 under active surveillance: systematic review and meta-analysis. BMJ. 2018 Feb 27;360:k499.

40. Liu G, Sharma M, Tan N, Barnabas R V. HIV-positive women have higher risk of human papilloma virus infection, precancerous lesions, and cervical cancer. AIDS. 2018 Mar 27;32(6):795–808.

41. Kelly H, Weiss HA, Benavente Y, de Sanjose S, Mayaud P, Qiao Y lin, et al. Association of antiretroviral therapy with high-risk human papillomavirus, cervical intraepithelial neoplasia, and invasive cervical cancer in women living with HIV: a systematic review and meta-analysis. Lancet HIV. 2018;5(1):e45–58.

42. Roura E, Travier N, Waterboer T, de Sanjosé S, Bosch FX, Pawlita M, et al. The Influence of Hormonal Factors on the Risk of Developing Cervical Cancer and Pre-Cancer: Results from the EPIC Cohort. PLoS One. 2016;11(1):e0147029.

43. McCredie MRE, Sharples KJ, Paul C, Baranyai J, Medley G, Jones RW, et al. Natural history of cervical neoplasia and risk of invasive cancer in women with cervical intraepithelial neoplasia 3: a retrospective cohort study. Lancet Oncol. 2008 May;9(5):425–34.

44. Canfell K, Barnabas R, Patnick J, Beral V. The predicted effect of changes in cervical screening practice in the UK: Results from a modelling study. Br J Cancer. 2004;91(3):530–6.

45. Tan N, Sharma M, Winer R, Galloway D, Rees H, Barnabas R V. Model-estimated effectiveness of single dose 9-valent HPV vaccination for HIV-positive and HIV-negative females in South Africa. Vaccine. 2018;36(32):4830–6.

46. McDonald AC, Tergas AI, Kuhn L, Denny L, Wright TC. Distribution of Human Papillomavirus Genotypes among HIV-Positive and HIV-Negative Women in Cape Town, South Africa. Front Oncol. 2014;4(March):48.

47. Giuliano AR, Botha MH, Zeier M, Abrahamsen ME, Glashoff RH, van der Laan LE, et al. High HIV, HPV, and STI prevalence among young Western Cape, South African women: EVRI HIV prevention preparedness trial. J Acquir Immune Defic Syndr. 2015;68(2):227–35.

48. Snyman LC, Dreyer G, Botha MH, van der Merwe FH, Becker PJ. The Vaccine and Cervical Cancer Screen (VACCS) project: Linking cervical cancer screening to HPV vaccination in the South-West District of Tshwane, Gauteng, South Africa. South African Med J. 2015 Jan 6;105(2):115–20.

49. Snyman LC, Dreyer G, Visser C, Botha MH, Van der Merwe FH. The Vaccine and Cervical Cancer Screen project 2 (VACCS 2): Linking cervical cancer screening to a two-dose HPV vaccination schedule in the South-West District of Tshwane, Gauteng, South Africa. South African Med J. 2015;105(3):191.

50. Adler DH, Wallace M, Bennie T, Mrubata M, Abar B, Meiring TL, et al. Cervical dysplasia and high-risk human papillomavirus infections among HIV-infected and HIV-uninfected adolescent females in South Africa. Infect Dis Obstet Gynecol. 2014;2014:498048.

51. Mbulawa ZZA, van Schalkwyk C, Hu N-C, Meiring TL, Barnabas S, Dabee S, et al. High human papillomavirus (HPV) prevalence in South African adolescents and young women encourages expanded HPV vaccination campaigns. PLoS One [Internet]. 2018;13(1):e0190166. Available from: http://www.ncbi.nlm.nih.gov/pubmed/29293566

52. Mbulawa ZZA, Marais DJ, Johnson LF, Boulle A, Coetzee D, Williamson A-L. Influence of human immunodeficiency virus and CD4 count on the prevalence of human papillomavirus in heterosexual couples. J Gen Virol. 2010 Dec;91(Pt 12):3023–31.

53. Denny L, Boa R, Williamson A-L, Allan B, Hardie D, Stan R, et al. Human papillomavirus infection and cervical disease in human immunodeficiency virus-1-infected women. Obstet Gynecol. 2008 Jun;111(6):1380–7.

54. Liebenberg LJP, McKinnon LR, Yende-Zuma N, Garrett N, Baxter C, Kharsany ABM, et al. HPV infection and the genital cytokine milieu in women at high risk of HIV acquisition. Nat Commun. 2019;10(1):1–12.

55. Vardas E, Giuliano AR, Goldstone S, Palefsky JM, Moreira ED, Penny ME, et al. External genital human papillomavirus prevalence and associated factors among heterosexual men on 5 continents. J Infect Dis. 2011 Jan 1;203(1):58–65.

56. Chikandiwa A, Chimoyi L, Pisa PT, Chersich MF, Muller EE, Michelow P, et al. Prevalence of anogenital HPV infection, related disease and risk factors among HIV-infected men in inner-city Johannesburg, South Africa: baseline findings from a cohort study. BMC Public Health. 2017 Jul 4;17(Suppl 3):425.

57. Moodley JR, Constant D, Hoffman M, Salimo A, Allan B, Rybicki E, et al. Human papillomavirus prevalence, viral load and pre-cancerous lesions of the cervix in women initiating highly active antiretroviral therapy in South Africa: a cross-sectional study. BMC Cancer. 2009 Jan;9:275.

58. Firnhaber C, Zungu K, Levin S, Michelow P, Montaner LJ, McPhail P, et al. Diverse and high prevalence of human papillomavirus associated with a significant high rate of cervical dysplasia in human immunodeficiency virus-infected women in Johannesburg, South Africa. Acta Cytol. 2009;53(1):10–7.

59. Mbulawa ZZA, Hu NC, Kufa-Chakezha T, Kularatne R, Williamson A-L. Sentinel surveillance of human papillomavirus genotypes among patients attending public healthcare facilities in South Africa, 2014-2016 133 [Internet]. Vol. 14, Communicable Diseases Surveillance Bulletin. 2016. 133–136 p. Available from: http://nicd.ac.za/assets/files/Sentinel surveillance of HPV.pdf

60. Cronjé HS, Parham GP, Cooreman BF, de Beer A, Divall P, Bam RH. A comparison of four screening methods for cervical neoplasia in a developing country. Am J Obstet Gynecol. 2003;188(2):395–400.

61. Denny L, Kuhn L, Pollack A, Wainwright H, Wright TC. Evaluation of alternative methods of cervical cancer screening for resource-poor settings. Cancer. 2000;89(4):826–33.

62. Kuhn L, Saidu R, Boa R, Tergas A, Moodley J, Persing D, et al. Clinical evaluation of modifications to a human papillomavirus assay to optimise its utility for cervical cancer screening in low-resource settings: a diagnostic accuracy study. Lancet Glob Heal. 2020;8(2):e296–304.

63. Lomalisa P, Smith T, Guidozzi F. Human Immunodeficiency Virus Infection and Invasive Cervical Cancer in South Africa. Gynecol Oncol. 2000;77:460–3.

64. Mbodi L, Adam Y. Reasons Why Women present with late stages of Cervical Cancer at Chris Hani Baragwanath Academic Hospital. University of the Witwatersrand; 2016.

65. Snyman L, Herbst U. Reasons why unscreened patients with cervical cancer present with advanced stage disease. South African J Gynaecol Oncol. 2013;5(1):16–20.

66. Sabulei C, Maree J. An exploration into the quality of life of women treated for cervical cancer. Curationis. 2019;42(1):1–9.

67. National Cancer Registry, National Institute for Occupational Health, National Health Laboratory Service. Cancer in South Africa 2017 Full Report [Internet]. 2020 [cited 2020 Jul 16]. Available from: https://www.nicd.ac.za/centres/national-cancer-registry/

68. Lomalisa P, Smith T, Guidozzi F. Human immunodeficiency virus infection and invasive cervical cancer in South Africa. Gynecol Oncol. 2000;77(3):460–3.
