# Supplementary material for: Estimating the effect of HIV on cervical cancer elimination in South Africa: Comparative modelling of the impact of vaccination and screening
Source: eClinicalMedicine. 2022 Nov 17;54:101754. doi: 10.1016/j.eclinm.2022.101754 (PMC9793279; doi:10.1016/j.eclinm.2022.101754)
Supplement: Model specific _Technical Appendix _T3 [file mmc9.docx]

Technical Appendix T3-Technical material for the *DRIVE* model

**Model-Specific Technical Appendix T3:** Technical material for the *DRIVE* model

*(Provincial level)*

**Table of contents**

Model overview………………………………………………………………………….……………Page 1

Technical description of the *DRIVE* model (KwaZulu-Natal) ………………………………………..Page 2

1. Demography, sexual behaviour and transmission………………………………………………..Page 2
2. Natural history of infection and disease progression……………………………………………..Page 3
3. Baseline interventions – for HIV………………………………………………………………....Page 4
4. Baseline interventions – for HPV and Cervical cancer…………………………………………...Page 5
5. Model calibration…………………………………………………………………………………Page 7
6. Parameter estimates, fitting/validation outcomes and data sources…..………….…………….....Page 9
7. Description of analysis……………………………………………………………………………Page 20
8. Fitting and validation results……………………………………………………………………...Page 22
9. Model equations…………………………………………………………………………………..Page 33
10. References….……………………………………………………………………………………..Page 44

**Model overview**

DRIVE is a model for the province of KwaZulu-Natal. Briefly, DRIVE is a deterministic transmission dynamic model that simulates HIV and HPV transmission (2 groups of high-risk (hr) genotypes: HPV16/18/31/33/45/52/58, non-vaccine high-risk HPV types), HIV disease progression and HPV-induced cervical carcinogenesis associated with each type group in a heterosexual population that is stratified by gender, 5-year age groups, and sexual activity. Each health state aims to represent the underlying true health state of the simulated individuals (as opposed to a diagnosed state) such as infection status, grade of cervical intraepithelial neoplasia (CIN1, 2, 3), and cancer stage (Figure 1). The model takes into account the multiple interactions between HIV and HPV infection and disease progression, namely it is assumed that HIV increases the risk of HPV acquisition and disease progression to cervical cancer. WLHIV on ART and virally suppressed are assumed to experience rates of HPV acquisition and disease progression comparable to HIV negative women, but their HPV clearance rate, disease regression rates, and cervical cancer-associated mortality are only partially restored (and are assumed similar to untreated WLHIV with high (>500) CD4 count). The model also assumes that condom use is effective against HPV acquisition. Male circumcision is assumed to have no effect on HPV.

The model represents variation in the levels of HIV interventions such as condom use, male circumcision and HIV treatment (ART) of people living with HIV (PLHIV) over time since the beginning of the HIV epidemic in 1980. The model assumes that all individuals treated with ART are virally suppressed. The model also represents HPV vaccination (starting in 2020) and *basecase* cervical cancer screening and treatment (starting in 2000).

The risk of HPV acquisition among susceptible individuals depends on their sexual activity, the prevalence of infection among partners, HIV and ART status, and level of condom use and HPV vaccination interventions. The transition rates between HPV health states depend on gender, age, HPV type group, and HIV/ART status. The risk of HIV acquisition depends on sexual activity, the prevalence of infection and viral load among partners, and level of HIV interventions. The transition rates between HIV health states depend on gender and age.

The model was parameterised and fitted using multivariate calibration process based on detailed data on sexual behaviour, HIV and HPV infections and cervical cancer epidemiology over time representative of the KwaZulu-Natal context. The data sources used at this stage are described below.

Model development and simulation were facilitated through the use of advanced computational, storage, and networking infrastructure provided by the Hyak supercomputer system at the University of Washington.

**Technical description of the *DRIVE* model (KwaZulu-Natal)**

**1) Demography, sexual behaviour and transmission:**

The DRIVE (Data-driven Recommendations for Interventions against Viral infEction) model represents the open and growing heterosexual population aged 0-79 in KwaZulu-Natal (KZN), South Africa. The model was adapted from a previously-published model calibrated to this context ([1](#_ENREF_1)). Model dynamics are governed by a system of differential equations evaluated at two-month time intervals. Beginning in 1925, the model initiates a population stratified by gender, sixteen 5-year age groups, and three sexual risk groups (low, moderate, and high risk) distinguished by the number of sexual partners each person is expected to have per year. The population distribution by gender and age in 1925 (Table 1) was projected backward from observed data on the total South African population and proportion residing in KwaZulu-Natal beginning in 1950, assuming exponential growth between 1925 and 1950 ([2](#_ENREF_2), [3](#_ENREF_3)).

Total fertility rates determine how many newborns enter the population annually. We model a 56% linear decline in fertility from 1960 to 2000, followed by a ten-year stabilization until 2010, and then a 24% linear decrease from 2010 values by 2020 consistent with literature, United Nations Population Division total fertility estimates, and KwaZulu-Natal population estimates ([2-5](#_ENREF_2)). Fertility rates are applied to women aged 15-49 and vary by age, CD4+ T-cell (CD4) count in women with HIV, and over time (Table 2) ([6](#_ENREF_6), [7](#_ENREF_7)). The proportion of births from mothers with HIV that result in perinatal transmission decreases linearly from 2004 to 2008, capturing improvements in services for pregnant women living with HIV (Table 3) ([8-10](#_ENREF_8)). We assume a 1:1 gender ratio at birth and that infected newborns have acute HIV. To age the population, one-fifth of each compartment moves to the next age group annually.

Persons leave the population due to death or aging past age 79. Background mortality rates are applied separately from HIV or cervical cancer disease-specific mortality and vary by gender, age, and time (Table 4). Before 1950, we use United Nations life tables for South Africa from the period of 1950 to 1955([2](#_ENREF_2)). We then assume that background mortality decreases linearly until 1985, the start of the generalized HIV epidemic. Background mortality changes linearly between 1985 and 2000 and between 2000 and 2020 according to mortality estimates from the IHME Global Burden of Disease Study ([11](#_ENREF_11)).

Sexual activity begins in the 10-14 age group. Each sexual risk group has an age-specific number of sexual partners (Table 6) and number of sexual acts per partnership per year (Table 7). Higher-risk groups are assumed to have fewer acts per partnership due to shorter partnership durations. The distribution of individuals in each sexual risk group by age (Table 5) was derived from Africa Centre cohort partnership data for KwaZulu-Natal ([12](#_ENREF_12)), calculated as the percentage of the cohort in age groups 15-19 through 45-49 who reported 0-1 partners (low risk), 2-4 recent partners (moderate risk), or 5 or more recent partners (high risk). Individuals aged 10-14 are assumed to be predominately low-risk, and the risk distribution for ages 45-49 is extrapolated up to age 79. Upon aging to the next five-year group, individuals are re-distributed into the closest unfilled sexual risk group. The risk distribution derived from male partner data is used for both men and women for simplicity. However, the number of partners and number of sexual acts per partnership within risk and age groups are calibrated and allowed to vary by gender to account for uncertainty in reported estimates due to reporting biases and the effects of concurrency on increasing transmission. Among men, we assume those aged 10-19 have the same number of acts per partnership as women of the same ages, whereas the number of acts among men aged 20-79 matches the number of acts among women of the next lowest age group, reflecting age discrepancies in relationships. The degree of assortativity in patterns of sexual mixing by sexual risk group is informed by literature, while the degree of assortativity in patterns of sexual mixing by age are calibrated (Table 8). The mixing matrix by age is biased such that women tend to form partnerships with men of the next oldest 5-year age group ([13-15](#_ENREF_13)).

Because our model assumes purely heterosexual contact, the modelled number of partnerships expected for men must match the number expected for women. The observed data used to inform these parameters are subject to selection and response biases that may result in imbalances, and our calibration procedure may also result in imbalanced parameters. We thus adjust contact rates such that the number of partnerships among men in a given age and risk group equals the number of partnerships that women have with men of that same age and risk group. We assume that this adjusted contact rate is equally driven by estimated rates for men and women due to lack of data to assume otherwise.

***HIV force of infection****:* The per-capita rate of HIV infection of susceptible individuals is a function of the number of partnerships, the prevalence of HIV infection among partners, patterns of sexual mixing by sexual activity and age, and per-partnership transmission probabilities. Per-partnership transmission probabilities are calculated as the cumulative risk of acquiring HIV from all sexual acts within the partnership (per-partnership transmission = $1-\left( 1-p \right)^{a}$, where $a$ is the number of acts and $p$ is the probability of transmission per act). The probability of transmission per sex act depends on the viral load of the partner with HIV (Tables 9-10). Risk of HIV transmission is highest during the initial acute stage of infection, decreases during the asymptomatic phase, and then gradually rises as an individual progresses to pre-AIDS symptomatic and AIDS stages ([16-19](#_ENREF_16)). As a proxy for decreased sexual activity due to pain and sickness during late-stage HIV, we reduce HIV per-act transmission to 10% of the rate during the AIDS stage and, similarly, reduce HPV per-act transmission by 90%([19](#_ENREF_19)). We assume equal probabilities of male-to-female and female-to-male HIV transmission across all viral load stages.

Condom use decreases the per-capita rate of HIV infection among all susceptible individuals. The degree of reduction is calculated as population-level condom use (defined as the average proportion of the population ever using condoms multiplied by the percent of sexual acts for which those persons use a condom) multiplied by the proportional reduction in HIV acquisition with condom use. Men without HIV who receive voluntary medical male circumcision (VMMC) also experience a reduced per-capita rate of HIV infection.

***HPV force of infection****:* The per-capita rate of HPV infection of susceptible individuals is a function of the number of partnerships, the prevalence of HPV infection among partners, patterns of sexual mixing by sexual activity and age, and per-partnership transmission probabilities calculated as the cumulative risk of acquiring HPV from all sexual acts within the partnership. The probability of transmission per sex act is assumed to be equivalent for vaccine-type and non-vaccine-type hrHPV and across all stages of precancer and cervical cancer (Table 11). We assume women with regional or distant cervical cancer (FIGO stages 2-4) also decrease their sexual activity due to pain, reducing HIV and HPV transmission by 50%.

Condom use decreases the per-capita rate of HPV infection among all susceptible individuals. The degree of reduction is calculated as population-level condom use (defined as the average proportion of the population ever using condoms multiplied by the proportion of sexual acts for which those persons use a condom) multiplied by the proportional reduction in HPV acquisition with condom use. Additionally, women who clear HPV develop temporary natural immunity that partially protects them against re-infection with the same HPV type. We assume HPV vaccination provides life-long protection against vaccine-type HPV infection (varied in sensitivity analysis). By comparison, the per-capita rate of HPV infection is increased among HPV-susceptible individuals with HIV who have progressed past the acute stage of infection. Persons with HIV who are treated with viral suppression are assumed to experience rates of HPV acquisition comparable to individuals without HIV.

**2) Natural history of infection and disease progression**

The model represents HIV and HPV transmission, disease progression, interaction between HIV and HPV, and the impact of prevention and treatment interventions on disease outcomes.

***HIV natural history****:* HIV is introduced to the model in 1980 and infection occurs either through heterosexual transmission or mother to child transmission. The natural history of HIV infection is then modelled in stages defined by CD4 count and viral load (Tables 12-13). When a person acquires HIV, they enter the acute stage characterized by a short duration and high probability of onward HIV transmission. The person then progresses through stages of decreasing CD4 count and increasing viral load, with transition rates stratified by gender and age ([17](#_ENREF_17), [20-22](#_ENREF_20)). HIV-associated mortality rates depend on CD4 cell count and age (Table 14) ([23-27](#_ENREF_23)). As a result of these combination of disease progression and mortality rates, women have a longer average expected survival than men, and persons aged 5-49 live longer than young children and older adults.

***HPV and cervical cancer natural history****:* HPV is introduced to the model in 1925 to allow HPV transmission dynamics and cervical cancer incidence to equilibrate prior to the introduction of HIV infection in 1980. HPV infections are grouped as either nonavalent vaccine-type hrHPV (types 16, 18, 31, 33, 45, 52, and 58) or non-vaccine type hrHPV (all other oncogenic types). The distribution of these type groups by stage of infection is reflective of observed data (Table 29) ([28-35](#_ENREF_28)). Oncogenic HPV infection in women can progress to precancerous lesions (categorized as cervical intraepithelial neoplasia (CIN) grades 1, 2, or 3) and cervical cancer (categorized as local, regional, or distant) (Table 15). We assume that HPV infection persists throughout all stages of CIN and cervical cancer disease. CIN1,2,3 can regress and HPV infection (without lesion) can clear naturally. HPV progression and regression rates vary by CIN stage, and progression, regression, and clearance rates vary by HPV type (Table 16). All women who clear HPV develop partial natural immunity against reinfection with the same HPV type group that also wanes over time (Table 15). In men, we model HPV infection, with clearance resulting in a transition back to the susceptible state with no natural immunity ([36](#_ENREF_36)). Men clear HPV faster than women (Table 15) ([37](#_ENREF_37)).

***Co-infections and effect of ART on HPV and disease:*** Persons with HIV who have progressed past the acute stage of HIV experience higher rates of HPV acquisition, immunity waning, and disease progression, and lower rates of HPV clearance and disease regression relative to individuals without HIV ([38](#_ENREF_38)). CD4 cell count is inversely correlated with the development of cervical cancer. HIV coinfection increases cervical cancer-associated mortality, but this effect decreases as cervical cancer progresses. Beginning in 2004, modelled individuals with HIV may achieve viral suppression with initiation of ART. We only model the effect of ART among individuals who achieve viral suppression: persons with HIV who initiate treatment without achieving viral suppression are assumed to have no benefit from treatment and are not tracked in our model. Treated person living with HIV who achieve viral suppression are assumed to experience rates of HPV acquisition comparable to individuals without HIV, but HPV clearance, disease progression rates, disease regression rates, and cervical cancer-associated mortality are equivalent to untreated individuals with high (>500) CD4 count.

**3) Baseline interventions – for HIV**

**Condom use**: Population-level condom use is defined as the average proportion of the population ever using condoms multiplied by the proportion of sexual acts for which those persons use a condom. Overall condom use is initiated in the model in 1995, scales up linearly from 1995 to 2000, and then remains constant. We assume condom use reduces HIV acquisition in both men and women by 80% ([39](#_ENREF_39)). We reduce the force of HIV infection applied to all susceptible individuals by a factor calculated as population-level condom use multiplied by protection afforded by condom use against HIV acquisition.

**Male circumcision**: Prior to the initiation of the South Africa National voluntary medical male circumcision (VMMC) program in 2010, circumcision was primarily targeted to young adult men as a rite of passage ([40](#_ENREF_40)). In addition to accounting for the historical practice of traditional circumcision ([41](#_ENREF_41), [42](#_ENREF_42)), modelling circumcision beginning in 1960 among youth results in circumcision prevalence among men aged 50 and older in 2012 corresponding to observed estimates ([43](#_ENREF_43)).We assume coverage of circumcision (either traditional or medical) increases linearly from 1960 to 2000 and between 2000 and 2008 to match coverage levels estimated from SABSSM data ([40](#_ENREF_40), [44](#_ENREF_44)). Following initiation of the national VMMC program in 2010, we model scale-up of circumcision for all men aged 15 or older at levels extrapolated backwards from 2012-2017 SABSSM and DHS data ([43](#_ENREF_43), [45](#_ENREF_45), [46](#_ENREF_46)). We assume country-level VMMC coverage data is reflective of that in KwaZulu-Natal as the overall proportion of men who received VMMC is similar in South Africa and KwaZulu-Natal ([43](#_ENREF_43), [45](#_ENREF_45)), and province-level estimates are not provided by age. We assume KwaZulu-Natal achieves 70% coverage for men aged 15-79 by 2030, with coverage increasing linearly between 2020 and 2030.

We assume that men without HIV who receive circumcision have decreased risk of HIV acquisition. Data suggest that men circumcised by a medical professional have a 60% lower risk of acquiring HIV compared to uncircumcised men ([47](#_ENREF_47), [48](#_ENREF_48)), and we reduce the force of infection applied to circumcised men without HIV by an equal amount. We assume no effect of circumcision for men with HIV ([49](#_ENREF_49)). We additionally assume that circumcision does not reduce the risk of HIV transmission to female partners([50](#_ENREF_50), [51](#_ENREF_51)).

**HIV treatment**: Beginning in 2004, modelled individuals may achieve viral suppression with initiation of ART. We only model the effect of ART among individuals who achieve viral suppression: persons with HIV who initiate treatment without achieving viral suppression are assumed to have no benefit from treatment and are not tracked in our model. Persons on treatment with viral suppression are assumed to have zero probability of transmitting HIV ([52](#_ENREF_52), [53](#_ENREF_53)), and to have the same fertility rates as women without HIV. HIV-associated mortality is reduced among treated persons compared to untreated individuals. Additionally, HIV-associated mortality rates applied to the population living with HIV and on treatment decrease over time, corresponding to an increase in the average CD4 count at ART initiation and improved regimens ([54-57](#_ENREF_54)).

We assume individuals aged 10-14 or older are eligible for ART. The scale-up of ART over time depends on CD4 count and gender. Reflecting changes in policy for treatment eligibility in South Africa ([58](#_ENREF_58)), ART becomes available in 2004 to persons with a CD4 count ≤200 cells/µL. The initiation threshold is subsequently raised to CD4 ≤350 cells/µL in 2011 and to CD4 ≤500 cells/µL in 2015. In 2016, ART becomes available to all individuals with HIV regardless of their CD4 cell count, including those with acute infection.

Informed by empirical data on ART coverage and viral suppression over time in South Africa and KwaZulu-Natal specifically ([45](#_ENREF_45), [59-62](#_ENREF_59)), we model higher rates of viral suppression in women than men. In all modelled scenarios (unless specified in sensitivity analysis) we assume KwaZulu-Natal achieves the UNAIDS 90-90-90 targets (90% of persons with HIV are diagnosed, 90% of persons diagnosed receive ART, and 90% of persons on ART have viral suppression([63](#_ENREF_63))) by 2030: the percentage of persons with HIV who are treated with viral suppression is assumed to increase linearly from 2017 levels (the latest year for which data were available) to 72.9% among both men and women by 2030.

Within gender and CD4 cell count categories, the probability of ART initiation is uniform by age or risk group. However, we do not model the process of discontinuation of ART and resulting loss of viral suppression, such that the cumulative probability of being on ART increases with age. This results in proportions with viral suppression that are lower in younger ages and higher in older ages compared to observed data. To control this age differential, we apply a minimum bound of 0.85*(target population-level viral suppression) and a maximum bound of 1.04*(target population-level viral suppression) within each age group; the proportion of the age-group on ART with viral suppression is checked at each time step, and eligible individuals are initiated on ART with viral suppression if coverage falls below the minimum for a given age group, and we discontinue treatment for some individuals if coverage exceeds the maximum within an age group. These age-specific minimum and maximum limits ensure that viral suppression is distributed more appropriately in all age groups while also matching the population-wide levels of viral suppression by gender in the observed data.

**4) Baseline interventions – for HPV and cervical cancer**

**Condom use**: Population-level condom use is defined as the average proportion of the population ever using condoms multiplied by the proportion of sexual acts for which those persons use a condom. Overall condom use is initiated in the model in 1995, scales up linearly from 1995 to 2000, and then remains constant. We assumed condoms reduce HPV acquisition among women by 70% and HPV acquisition among men by 46% ([64](#_ENREF_64), [65](#_ENREF_65)). We reduce the force of HPV infection applied to all susceptible individuals by a factor calculated as population-level condom use multiplied by protection afforded by condom use against HPV acquisition.

**Male circumcision:** We assume that circumcision does not reduce the risk of HPV acquisition or transmission ([66-69](#_ENREF_66)).

**Cervical cancer screening**: Beginning in 2000, we model once-per-lifetime cervical cancer screening for women in the age range of 35-39. Although South African policy during this time frame ([70](#_ENREF_70)) recommended screening every 10 years beginning at age 30, observed data suggest low compliance with this schedule ([71](#_ENREF_71), [72](#_ENREF_72)). Beginning in 2011, screening was recommended every 3 years in women with HIV with low CD4 count, and for all women with HIV from 2016 onwards, yet data do not show the expected increases in screening uptake corresponding with these changes ([73](#_ENREF_73)). Informed by consultation with in-country experts, we conservatively assume only one lifetime screen in the age range of 35-39 in the *basecase* scenario. We assumed that screening coverage increases linearly from 0% in 2000 to 18% of women aged 35-39 by 2003, and to 48% of women aged 35-39 by 2016 ([46](#_ENREF_46), [74](#_ENREF_74)). We applied screening coverage uniformly across the 5-year age group so that the expected proportion of women aging into the next age group had a history of cervical cancer screening.

Our *basecase* scenario for cervical cancer screening and treatment corresponds to current guidelines and practice in South Africa (see Table 23 for treatment cascade) ([75](#_ENREF_75)). We assume that women are screened using cytology (Papanicolaou smear) and that 57% of CIN2+ and cervical cancer cases are correctly identified([76](#_ENREF_76)). Of the women who test positive, 72% return for triage using colposcopic biopsy ([77](#_ENREF_77)). Fifty-one percent of women with confirmed CIN2+ are treated with the loop electrosurgical excision procedure (LEEP, also known as large loop excision of the transformation zone, or LLETZ) ([77](#_ENREF_77)). Although cryotherapy is recommended for lesions that can be treated with ablative therapy, the availability of cryotherapy is limited in South Africa, such that we make the simplifying assumption of treatment with LEEP only ([75](#_ENREF_75)). Among women confirmed to have cervical cancer after triage, 40% return for hysterectomy treatment ([77](#_ENREF_77)). These rates reflect challenges with follow-up and retention for cervical cancer prevention and treatment, particularly with a multi-visit screening and treatment approach.

Based on data from a recent meta-analysis, we assume that LEEP successfully treats CIN2+ lesions 91% of the time in women without HIV and 77% of the time in women with HIV ([78](#_ENREF_78)). Additionally, evidence suggests that 28% of women treated with LEEP have persistent HPV infection, including cases with residual or recurrent CIN ([79](#_ENREF_79)). Because this estimate is drawn from studies of primarily women without HIV, we used the estimate of 9% treatment failure stated above (also measured among women without HIV) to calculate the percent of women who would have persistent HPV with successfully treated lesions (18.5%). Lacking data on differential HPV persistence by HIV status, we assume that HPV persistence after lesion clearance is the same among women with HIV as women without HIV. Women with a successfully treated lesion who also clear HPV are assumed to develop temporary, partial immunity against reinfection with the same modelled HPV type group(s) present at the time of treatment (vaccine-type vs. other hrHPV).

We assume that hysterectomy is 100% effective at treating cervical cancer. We do not account for treatment or recurrence of late-stage cancers that have spread to other organ systems. After hysterectomy, women remain in the model population but are assumed to be infertile, unable to acquire or transmit HPV, and to have no increase in mortality due to their prior cancer status. Because the focus of our analyses is on cervical cancer incidence, we do not model symptomatic diagnosis of cervical cancer or treatment with other methods such as radiation or chemotherapy. We assume cervical cancer screening and treatment to be equally effective for both vaccine-type and non-vaccine type hrHPV. If a woman is coinfected, her most advanced HPV type group determines her disease status.

**HPV vaccination**: In most scenarios, we assume that nonavalent HPV vaccination provides complete, lifelong protection against the seven oncogenic HPV types included in the nonavalent vaccine (types 16, 18, 31, 33, 45, 52, and 58) and 0% protection against other hrHPV types. The vaccine is ineffective for persons with an active nonavalent vaccine-type HPV infection at the time of vaccination. The dynamic nature of our transmission model captures population-level effects such as herd immunity.


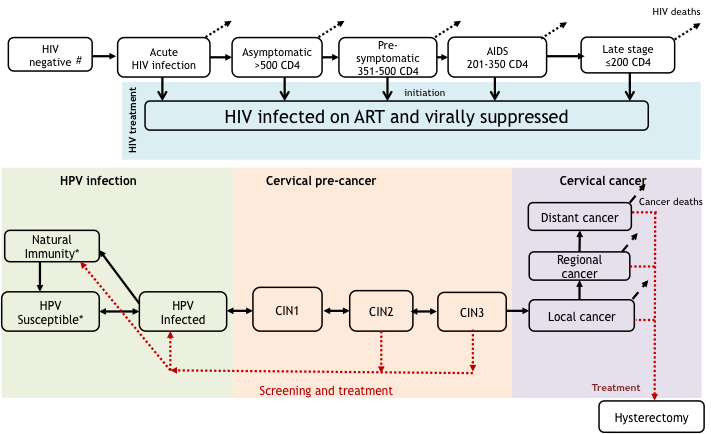


Figure 1. *Flowchart of the model representing the HIV and HPV/CC health states represented in the model. Arrows represent flows between health states. ^*^HPV vaccination is effective only for individuals in these stages. Once received, vaccination status is tracked through subsequent transitions. ^#^HIV susceptible men are stratified by circumcision status, with circumcision decreasing the risk of HIV acquisition.*

**5) Model calibration**

We took a phased approach to model calibration, summarised and then described in greater detail below:

Phase 0: We used hand-calibration to explore the sensitivity of model outcomes to individual parameters. This exploratory step informed our decision to divide the formal Bayesian calibration into two additional phases.

Phase 1: Beginning with a uniform prior distribution for each parameter, we first used Approximate Bayesian Computation-Sequential Monte Carlo (ABC-SMC) with the summed-log-likelihood as our overall fit metric to calibrate 22 sexual behaviour and HIV natural history parameters to observed demographic and HIV prevalence data. We sampled a total of 91,980 parameter sets and selected the 50 best-fitting sets which we carried into Phase 2.

Phase 2: Randomly resampling from the 50 best-fitting parameter sets of Phase 1, we then used the same ABC-SMC algorithm and summed-log-likelihood metric to fit 23 HPV natural history parameters to observed data on HPV prevalence, CIN prevalence, cervical cancer incidence, and type distribution. After sampling a total of 25,760 parameter sets, the range of individual parameters among the 25 best-fitting sets of Phase 2 conditional on the Phase 1 sampled values capture the parameter uncertainty in our model predictions.

*Additional details*:

In phase 0, we first identified parameters for which empirical evidence was limited. We assumed a uniform prior distribution for each parameter, and we used published estimates and expert opinion to assign upper and lower bounds. By systematically varying parameters within the specified ranges, we verified that the parameters included in the calibration and their prior ranges could plausibly produce model results fitting the observed data. We also assessed the relative impact of individual parameters on different model outcomes. From this exploratory process, we determined that HIV outcomes are more sensitive to sexual behaviour parameters than HPV-related outcomes, that HIV outcomes are tightly linked to demographic outcomes, and that pre-cancer and cancer outcomes are primarily driven by the HPV natural history parameters. These findings allowed us to narrow prior ranges and divide our formal Bayesian calibration into two subsequent phases, thereby increasing efficiency. To further increase efficiency, we fixed parameters with negligible impact on all model outcomes to the most robust estimates from literature.

For Phases 1 and 2, we used the Approximate Bayesian Computation-Sequential Monte Carlo (ABC-SMC) algorithm to efficiently explore the multidimensional parameter space and identify parameter sets that generated model outcomes that best fit observed data ([80-82](#_ENREF_80)). The ABC-SMC approach focuses sampling in the highest likelihood areas and avoids resampling identical parameter sets. Because a Bayesian calibration approach frames parameters as probabilistic and unknown, this allowed us to systematically estimate the range of outcomes given uncertainty in the underlying parameters. At each iteration, we retain only a proportion (alpha) of the best-fitting sets, re-sample from those sets, and then use a Gaussian kernel to perturb the values, therefore focusing sampling in the areas of highest likelihood while avoiding re-sampling the exact same sets. Convergence is achieved when less than a set proportion (pacc_min) of accepted particles have a likelihood greater than epsilon, or the smallest likelihood of the accepted particles from the last iteration. To balance quality with computational time, we choose intermediate values for alpha of 0.6 (phase 1) or 0.4 (phase 2), and set pacc_min to 0.1.

We used the summed-log-likelihood as our overall metric of model fit to combine outcomes, and maximum likelihood estimation (MLE) to evaluate the probability that potential parameter sets produced observed data. The point in multidimensional parameter space that maximizes the likelihood function is the most likely to have produced the observed trends in transmission of infection and development of disease. We defined likelihood equations for each target. By assuming that all outputs followed independent normal distributions, we were able to use the same equation form for all targets. For prevalence data, we assumed a normal approximation of the binomial where $\mu$ = prevalence proportion (p) and variance = (p(1-p))/N for a sample size N. For incidence data, we assumed a normal approximation of a Poisson distribution where $\mu$= λ and variance = λ, where λ = the annual cervical cancer incidence rate. We assumed a normal distribution for the total population size of KwaZulu-Natal and that the 2019 estimate had the same variance as the 2011 estimate. In most cases, mean values were derived from a single study. However, the proportions of vaccine and non-vaccine type HPV by disease stage were averaged across several studies. Therefore, we assumed a normal approximation of the binomial and calculated the variance of an average as = (1/N)^2 * (sum of the individual variances). When observed values were estimated from a multi-year sample, we compared to modelled results from the year at the middle of the time interval.

First, in Phase 1, we began with a uniform prior distribution for each parameter and used ABC-SMC to fit 22 sexual behaviour and HIV natural history parameters to observed demographic and HIV prevalence data (Tables 24-25). We sampled a total of 91,980 parameter sets, from which we selected the 50 best-fitting sets which we carried into Phase 2.

Second, in phase 2, we used ABC-SMC to fit 23 HPV natural history parameters to observed data on HPV prevalence, CIN prevalence, cervical cancer incidence, and type distribution (Tables 26-29). For each Phase 2 simulation, we randomly resampled the sexual behaviour and HIV natural history parameters from the 50 best-fitting parameter sets of Phase 1. After sampling a total of 25,760 parameter sets, the range of individual parameters among the 25 best-fitting Phase 2 sets and their corresponding Phase 1 sampled values defined parameter uncertainty (see Figures 4-13 for calibration results).

To verify our ability to accurately predict future outcomes, we cross-validated our HIV natural history module by comparing model HIV prevalence and HIV incidence predictions to additional time points and data sources (Tables 30-31). More specifically, we compared our predicted HIV prevalence to empirical estimates from 2010 to 2016 by gender and age from the same reference as our calibration data set ([12](#_ENREF_12)). We also compared our modelled HIV prevalence for both genders combined in broad age groups to observed data for KwaZulu-Natal from the 2017 SABSSM survey ([45](#_ENREF_45)). Finally, we compared HIV incidence, a non-calibrated output, to observed data from the Africa Centre cohort in KwaZulu-Natal, the same cohort captured by our prevalence data ([83](#_ENREF_83)). Alignment of our model to these external data supports the robustness of our predicted HIV dynamics (Figures 14-20). Ideally, we would also validate our HPV module to additional data on HPV prevalence or cervical cancer incidence. However, reliable data are sparse for these outcomes, and we used the best data available for calibration.

**6) Parameter estimates, fitting/validation outcomes and data sources**

Table 1. Initial population size in 1925.

| Age group | Initial population size | | References |
| --- | --- | --- | --- |
|  | Men | Women |  |
| 0 – 4 | 232193 | 234484 | ([2](#_ENREF_2), [3](#_ENREF_3)) |
| 5 – 9 | 159981 | 163174 |  |
| 10 – 14 | 130604 | 133156 |  |
| 15 – 19 | 116664 | 114322 |  |
| 20 – 24 | 105385 | 99000 |  |
| 25 – 29 | 95487 | 87233 |  |
| 30 – 39 | 88296 | 80039 |  |
| 35 – 39 | 81236 | 71998 |  |
| 40 – 44 | 73180 | 65881 |  |
| 45 – 49 | 62270 | 56376 |  |
| 50 – 54 | 49095 | 47787 |  |
| 55 – 59 | 38061 | 38751 |  |
| 60 – 64 | 27479 | 32112 |  |
| 65 – 69 | 18576 | 23810 |  |
| 70 – 74 | 12335 | 15545 |  |
| 75 – 79 | 6595 | 7570 |  |
| TOTAL | 1297437 | 1271238 |  |

Table 2. Baseline annual fertility rates by age and HIV status.

| Age group | Annual fertility rates before 1960 (births per capita per year) | | | | | | References |
| --- | --- | --- | --- | --- | --- | --- | --- |
|  | HIV uninfected or WLHIV on ART | Acute | CD4 >500 | CD4 350-500 | CD4 200-350 | CD4 <200 |  |
| 0 – 14 | 0 | 0 | 0 | 0 | 0 | 0 | ([6](#_ENREF_6), [7](#_ENREF_7)) |
| 15 – 19 | 0.1555 | 0.1555 | 0.1555 | 0.0902 | 0.0902 | 0.0638 |  |
| 20 – 24 | 0.3058 | 0.3058 | 0.3058 | 0.1774 | 0.1774 | 0.1254 |  |
| 25 – 29 | 0.3120 | 0.3120 | 0.3120 | 0.1809 | 0.1809 | 0.1279 |  |
| 30 – 34 | 0.2323 | 0.2323 | 0.2323 | 0.1347 | 0.1347 | 0.0953 |  |
| 35 – 39 | 0.1483 | 0.1483 | 0.1483 | 0.0860 | 0.0860 | 0.0608 |  |
| 40 – 44 | 0.0596 | 0.0596 | 0.0596 | 0.0346 | 0.0346 | 0.0244 |  |
| 45 – 49 | 0.0194 | 0.0194 | 0.0194 | 0.0112 | 0.0112 | 0.0079 |  |
| 50 – 79 | 0 | 0 | 0 | 0 | 0 | 0 |  |

Table 3. Proportion of births from women with HIV that result in mother-to-child transmission (MTCT).

| Year | Proportion of births with MTCT | References |
| --- | --- | --- |
| Before 2004 | 0.340 | ([8-10](#_ENREF_8)) |
| By 2005 | 0.292 |  |
| After 2008 | 0.071 |  |

Table 4. Annual background mortality rates.

|  | **Annual background mortality rates (per capita deaths per year)** | | | | | | | | **References** |
| --- | --- | --- | --- | --- | --- | --- | --- | --- | --- |
| **Age group** | Before 1950 | | By 1985 | | By 2000 | | By 2020 | |  |
|  | Men | Women | Men | Women | Men | Women | Men | Women |  |
| **0 – 4** | 0.1901 | 0.1628 | 0.0570 | 0.0474 | 0.0144 | 0.0118 | 0.0054 | 0.0046 | ([2](#_ENREF_2), [11](#_ENREF_11)) |
| **5 – 9** | 0.0087 | 0.0074 | 0.0025 | 0.0019 | 0.0013 | 0.0010 | 0.0004 | 0.0003 |  |
| **10 – 14** | 0.0048 | 0.0044 | 0.0016 | 0.0012 | 0.0010 | 0.0008 | 0.0002 | 0.0001 |  |
| **15 – 19** | 0.0060 | 0.0049 | 0.0025 | 0.0017 | 0.0022 | 0.0018 | 0.0007 | 0.0005 |  |
| **20 – 24** | 0.0085 | 0.0056 | 0.0038 | 0.0024 | 0.0044 | 0.0050 | 0.0021 | 0.0010 |  |
| **25 – 29** | 0.0094 | 0.0065 | 0.00438 | 0.0031 | 0.0079 | 0.0087 | 0.0030 | 0.0018 |  |
| **30 – 34** | 0.0105 | 0.0073 | 0.0052 | 0.0038 | 0.0110 | 0.0093 | 0.0040 | 0.0026 |  |
| **35 – 39** | 0.0122 | 0.0083 | 0.0064 | 0.0046 | 0.0131 | 0.0095 | 0.0049 | 0.0030 |  |
| **40 – 44** | 0.0144 | 0.0096 | 0.0080 | 0.0057 | 0.0141 | 0.0086 | 0.0055 | 0.0033 |  |
| **45 – 49** | 0.0166 | 0.0105 | 0.0101 | 0.0067 | 0.0172 | 0.0094 | 0.0074 | 0.0045 |  |
| **50 – 54** | 0.0206 | 0.0136 | 0.0140 | 0.0090 | 0.0227 | 0.0120 | 0.0108 | 0.0059 |  |
| **55 – 59** | 0.0255 | 0.0180 | 0.0186 | 0.0120 | 0.0282 | 0.0153 | 0.0161 | 0.0086 |  |
| **60 - 64** | 0.0352 | 0.0270 | 0.0278 | 0.0185 | 0.0378 | 0.0218 | 0.0230 | 0.0117 |  |
| **65- 69** | 0.0506 | 0.0420 | 0.0428 | 0.0304 | 0.0466 | 0.0303 | 0.0321 | 0.0165 |  |
| **70 - 74** | 0.0771 | 0.0674 | 0.0685 | 0.0517 | 0.0680 | 0.0452 | 0.0405 | 0.0231 |  |
| **75 - 79** | 0.1190 | 0.1070 | 0.1120 | 0.0881 | 0.0839 | 0.0559 | 0.0570 | 0.0353 |  |

Table 5. Sexual risk distribution by age.

| **Age group** | **Risk distribution** | | | **References** |
| --- | --- | --- | --- | --- |
|  | Low risk | Moderate risk | High risk |  |
| **10 – 14** | 0.980 | 0.015 | 0.005 | ([12](#_ENREF_12)) |
| **15 – 19** | 0.509 | 0.408 | 0.083 |  |
| **20 – 24** | 0.472 | 0.443 | 0.085 |  |
| **25 – 29** | 0.510 | 0.412 | 0.078 |  |
| **30 – 34** | 0.605 | 0.342 | 0.054 |  |
| **35 – 39** | 0.766 | 0.203 | 0.031 |  |
| **40 – 44** | 0.818 | 0.168 | 0.014 |  |
| **45 – 79** | 0.851 | 0.148 | 0.001 |  |

Table 6. Annual number of sexual partnerships by gender, age, and risk.

| **Sexual risk group** | **Age group** | **Annual number of sexual partnerships**  **Mean [Uncertainty Range]** | | **References** |
| --- | --- | --- | --- | --- |
|  |  | Men | Women |  |
| **High** | 10-14 | 0.5x calibrated annual partnerships in the 15-19 age group | | Calibrated in Phase 1. Values presented are the mean and range of the randomly sampled Phase 1 values corresponding to the 25 best-fitting parameter sets after Phase 2. Prior bounds were defined around observed data on male and female partnerships ([12](#_ENREF_12)), with ranges informed by Phase 0. |
|  | 15-19 | 2.23  [0.27, 7.50] | 10.07  [5.92, 13.34] |  |
|  | 20-24 | 12.33  [5.02, 17.32] | 25.84  [13.18, 37.52] |  |
|  | 25-29 | 21.28  [8.99, 35.22] | 10.70  [5.21, 26.69] |  |
|  | 30-44 | 20.32  [7.89, 34.78] | 8.58  [5.31, 14.51] |  |
|  | 45-79 | 13.85  [5.24, 17.83] | 9.33  [5.65, 14.89] |  |
| **Moderate risk multiplier on high risk** | 15-79 | 0.16  [0.11, 0.29] | 0.47  [0.18, 0.86] | Calibrated in Phase 1. Values presented are the mean and range of the randomly sampled Phase 1 values corresponding to the 25 best-fitting parameter sets after Phase 2. Prior range informed by observed estimates and Phase 0. |
| **Low risk multiplier on moderate risk** | 15-79 | 0.24  [0.16, 0.52] | 0.49  [0.24, 0.73] |  |

Table 7. Annual sexual acts per partnership among low-risk women by age. We assume acts per partnership for moderate- and high-risk individuals are 0.60x acts in the next lowest risk group. Phase 0 demonstrated that the model’s fit to observed HPV and CIN2/3 prevalence by age was sensitive to age trends in female acts. Among men, we assume those aged 10-19 have the same number of acts per partnership as women of the same age, whereas men aged 20-79 have equal acts to women of the next lowest age group, reflecting age disparities in relationships.

| **Age group** | **Annual acts per partnership**  **Mean [Uncertainty Range]** | **References** |
| --- | --- | --- |
| **10-14** | 0.5x calibrated annual acts per partnership in the 15-19 age group | Calibrated in Phase 1. Values presented are the mean and range of the randomly sampled Phase 1 values corresponding to the 25 best-fitting parameter sets after Phase 2. Prior range informed by Phase 0. |
| **15-19** | 65.71 [58.17, 76.37] |  |
| **20-24** | 72.94 [54.24, 84.97] |  |
| **25-29** | 55.39 [47.20, 62.32] |  |
| **30-44** | 49.22 [41.04, 59.50] |  |
| **45-79** | 54.02 [42.06, 64.40] |  |

Table 8. Sexual mixing by sexual risk and age group. (0 < $\epsilon_{a/r}$ < 1), where ($\epsilon_{a/r}=0)$ indicates completely assortative mixing, and ($\epsilon_{a/r}=1)$ indicates completely random mixing

|  | **Mixing parameters**  **Fixed value, or**  **Mean [Uncertainty Range]** | **References** |
| --- | --- | --- |
| **By age group** | 0.43 [0.13, 0.56] | Calibrated in Phase 1. Values presented are the mean and range of the randomly sampled Phase 1 values corresponding to the 25 best-fitting parameter sets after Phase 2. Prior set according to expert opinion (Ruanne Barnabas, personal communications) and published literature ([84](#_ENREF_84), [85](#_ENREF_85)) indicating that mixing by age is not random but reflects that women tend to prefer older men, as suggested by data. |
| **By risk group** | 0.3 | ([13](#_ENREF_13)) |

Table 9: HIV transmission rate per act.

| **HIV transmission rate per act**  **Mean [Uncertainty Range]** | **References** |
| --- | --- |
| 0.0009  [0.0007, 0.0013] | Calibrated in Phase 1. Values presented are the mean and range of the randomly sampled Phase 1 values corresponding to the 25 best-fitting parameter sets after Phase 2. Prior set according to data on female-to-male and male-to-female transmission in developed and developing countries ([86](#_ENREF_86)), and narrowed in Phase 0 to exclude values inconsistent with observed cohort data. |

Table 10. Risk multipliers for HIV transmission by viral load. The probability of HIV transmission per act depends on the viral load of the HIV-positive partner.

| Risk multiplier for HIV transmission | | | | | **References** |
| --- | --- | --- | --- | --- | --- |
| Acute | Asymptomatic | Pre-AIDS symptomatic | AIDS | Late-stage |  |
| 9.0 | 1.0 | 2.5 | 7.0 | 0.7 | ([16-19](#_ENREF_16), [86](#_ENREF_86)) |

Table 11. HPV transmission rate per act.

| HPV transmission rate per act  Mean [Uncertainty Range] | References |
| --- | --- |
| 0.008 [0.003, 0.016] | Calibrated. Values presented are the mean and range of the 25 best-fitting parameter sets after Phase 2. Prior based on observed data ([87](#_ENREF_87)), and narrowed in Phase 0 to exclude values inconsistent with observed cohort data. |

Table 12. The duration of time in each CD4 stage by gender and age.

| Age group | Duration of time spent in CD4 stage with untreated infection (years) | | | | | References |
| --- | --- | --- | --- | --- | --- | --- |
|  | Acute | CD4 ≥500 | CD4 350-500 | CD4 200-350 | CD4 ≤200 |  |
|  | Men | | | | | ([20](#_ENREF_20), [22](#_ENREF_22)) |
| 0 – 4 | 0.25 | 0.25 | 3.56 | 4.67 | 2.13 |  |
| 5 – 49 | 0.25 | 0.25 | 3.56 | 4.67 | 3.70 |  |
| 50 – 79 | 0.25 | 0.25 | 2.85 | 4.51 | 1.85 |  |
|  | Women | | | | |  |
| 0 – 4 | 0.25 | 0.93 | 3.71 | 4.68 | 2.13 |  |
| 5 – 49 | 0.25 | 0.93 | 3.71 | 4.68 | 3.70 |  |
| 50 – 79 | 0.25 | 0.29 | 3.34 | 4.23 | 1.85 |  |

Table 13. The duration of time in each viral load stage by gender and age.

| Age group | Duration of time spent in viral load stage (years) | | | | | References |
| --- | --- | --- | --- | --- | --- | --- |
|  | Acute | Asymptomatic | Pre-AIDS symptomatic | AIDS | Late-stage |  |
|  | Men | | | | | ([17](#_ENREF_17), [21](#_ENREF_21)) |
| 0 – 4 | 0.25 | 5.03 | 4.00 | 0.75 | 0.83 |  |
| 5 – 49 | 0.25 | 6.60 | 4.00 | 0.75 | 0.83 |  |
| 50 – 79 | 0.25 | 3.88 | 4.00 | 0.75 | 0.83 |  |
|  | Women | | | | |  |
| 0 – 4 | 0.25 | 5.87 | 4.00 | 0.75 | 0.83 |  |
| 5 – 49 | 0.25 | 7.44 | 4.00 | 0.75 | 0.83 |  |
| 50 – 79 | 0.25 | 4.13 | 4.00 | 0.75 | 0.83 |  |

Table 14. HIV-associated mortality.

| Age group | Annual HIV-associated mortality rates (per capita deaths per year) | | | | | References |
| --- | --- | --- | --- | --- | --- | --- |
|  | Acute | CD4 ≥500 | CD4 350-500 | CD4 200-350 | CD4 ≤200 |  |
| 0 – 4 | 0 | 0.4700 | 0.4700 | 0.4700 | 0.4700 | ([23-27](#_ENREF_23)) |
| 5 – 49 | 0 | 0.0035 | 0.0255 | 0.0455 | 0.2655 |  |
| 50 – 79 | 0 | 0.0071 | 0.0511 | 0.0911 | 0.5311 |  |

Table 15. Annual HPV natural immunity clearance rate among women, multiplier on transition from HPV infection to susceptible among men, and cervical cancer progression rates.

| Transition rate or multiplier | | Fixed value, or Mean [Uncertainty Range] | References |
| --- | --- | --- | --- |
| Multiplier on force of HPV infection for extent of type-specific natural immunity protection following recent clearance of this type | | 0.7 [0.5, 1.0] | Calibrated. Values presented are the mean and range of the 25 best-fitting parameter sets after Phase 2. Prior lower bound based on observed data ([88](#_ENREF_88)). |
| Per capita annual rate of waning from immune to susceptible among women | | 0.024 | ([89](#_ENREF_89)) |
| Multiplier on annual transition rate from HPV infection to susceptible, applied to men only | | 2.4 [1.2, 3.4] | Calibrated. Values presented are the mean and range of the 25 best-fitting parameter sets after Phase 2. Data suggests decreased HPV persistence among men ([37](#_ENREF_37)). This parameter also allows flexibility around assumptions that female-to-male and male-to-female transmission are equal, and that men have no natural immunity after HPV infection clearance. |
| Annual cervical cancer progression rates | Local to Regional | 0.020 | ([90](#_ENREF_90)) |
|  | Regional to Distant | 0.025 |  |

Table 16. Annual HPV clearance and precancer progression and regression rates.

| Progression rates  Mean [Uncertainty Range] | | | | References |
| --- | --- | --- | --- | --- |
| Transition rate (per capita per year) | Age group | Vaccine-type | Non-vaccine-type | Calibrated. Calculated as (baseline transition rate) * (Mean [Uncertainty Range] of calibrated rate multiplier). Prior ranges of rate multipliers were based on exploration of a broad range of values in Phase 0. Baseline values were derived from Cancer Council New South Wales estimates previously described by Tan et al. (2018) in Supplement Table S25 ([1](#_ENREF_1)). This set of estimates defines rates for HPV 16, 18, and other high-risk types, and includes additional state transitions. We therefore reweighted types and consolidated transitions to better match our HPV type grouping and HPV natural history model structure. Varying rates by age are used to approximate the increased risks associated with HPV persistence since the model does not track infection duration within individuals, and rates are scaled up linearly across the age groupings. |
| HPV infection to CIN1 | 10-29 | 0.2065  [0.1114, 0.3612] | 0.1746  [0.0680, 0.3263] |  |
|  | 30-49 | 0.2168  [0.1170, 0.3793] | 0.1833  [0.0714,0.3427] |  |
|  | 50-79 | 0.2271  [0.1226, 0.3974] | 0.1921  [0.0748, 0.3590] |  |
| CIN1 to CIN2 | 10-29 | 0.2476  [0.0902, 0.5084] | 0.1197  [0.0320, 0.2421] |  |
|  | 30-49 | 0.2897  [0.1055, 0.5948] | 0.1400  [0.0374, 0.2833] |  |
|  | 50-79 | 0.3516  [0.1280, 0.7219] | 0.1699  0.0454, 0.3438] |  |
| CIN2 to CIN3 | 10-29 | 0.1989  [0.0603, 0.3377] | 0.1305  [0.0482, 0.2016] |  |
|  | 30-49 | 0.3182  [0.0965, 0.5403] | 0.2088  [0.0772, 0.3225] |  |
|  | 50-79 | 0.3978  [0.1206, 0.6754] | 0.2610  [0.0965, 0.4032] |  |
| CIN3 to cervical cancer | 10-29 | 0.0127  [0.0056, 0.0191] | 0.0028  [0.0012, 0.0038] |  |
|  | 30-49 | 0.0318  [0.0140, 0.0479] | 0.0071  [0.0030, 0.0094] |  |
|  | 50-79 | 0.1146  [0.0503, 0.1723] | 0.0256  [0.0108, 0.0338] |  |
| Regression and clearance rates  Mean [Uncertainty Range] | | | |  |
| Transition rate (per capita per year) | Age group | Vaccine-type | Non-vaccine-type |  |
| HPV infection to naturally immune (women) or susceptible (men) | 10-29 | 1.2496  [0.7678, 1.7061] | 1.2617  [0.7745, 1.6579] |  |
|  | 30-49 | 1.2496  [0.7678, 1.7061] | 1.2617  [0.7745, 1.6579] |  |
|  | 50-79 | 0.6373  [0.3950, 0.8456] | 0.6435  [0.3950, 0.8456] |  |
| CIN1 to HPV infection | 10-29 | 0.4553  [0.2235, 0.7188] | 0.3171  [0.0979, 0.4448] |  |
|  | 30-49 | 0.4553  [0.2235, 0.7188] | 0.3171  [0.0979, 0.4448] |  |
|  | 50-79 | 0.4553  [0.2235, 0.7188] | 0.3171  [0.0979, 0.4448] |  |
| CIN2 to CIN1 | 10-29 | 0.5490  [0.3280, 0.7518] | 0.4772  [0.0878, 0.7241] |  |
|  | 30-49 | 0.5490  [0.3280, 0.7518] | 0.4772  [0.0878, 0.7241] |  |
|  | 50-79 | 0.5490  [0.3280, 0.7518] | 0.4772  [0.0878, 0.7241] |  |
| CIN3 to CIN2 | 10-29 | 0.0809  [0.0108, 1398] | 0.1160  [0.0538, 0.1841] |  |
|  | 30-49 | 0.0647  [0.0086, 0.1118] | 0.0928  [0.0430, 0.1473] |  |
|  | 50-79 | 0.0485  [0.0065, 0.0839] | 0.0696  [0.0323, 0.1104] |  |

Table 17. Multipliers on HPV acquisition, clearance, immunity waning, progression, and regression rates for persons with HIV by CD4 count and treatment status. Persons on ART are assumed to be virally suppressed.

| Multiplier for persons with HIV | CD4 Stage  Fixed value, or Mean [Uncertainty Range] | | | | | References |
| --- | --- | --- | --- | --- | --- | --- |
|  | On ART | CD4 ≥500 | CD4 350-500 | CD4 200-350 | CD4 ≤200 |  |
| HPV acquisition | 1.00 | 1.78 | 1.99 | 2.12 | 2.32 | ([1](#_ENREF_1)) |
| HPV infection to naturally immune (women) or susceptible (men) | 0.60 | 0.60 | 0.55 | 0.45 | 0.30 |  |
| Natural immunity waning | 1.42 | 1.42 | 1.57 | 1.97 | 2.83 |  |
| CIN1 to CIN2 progression | 1.00 | 1.00 | 2.20  [1.53, 2.76] | 2.77  [1.93, 3.49] | 3.12  [2.17, 3.92] | Calibrated. Calculated as (baseline multiplier) * (Mean [Uncertainty Range] of calibrated multiplier). Prior ranges of multipliers were based on Phase 0. Baseline multipliers based on Liu et al. (2018) and adjusted to the KwaZulu-Natal setting by Tan et al. (2018) ([1](#_ENREF_1), [38](#_ENREF_38)). |
| CIN2 to CIN3 progression | 1.00 | 1.00 | 1.41  [1.01, 1.97] | 1.91  [1.36, 2.66] | 2.47  [1.76, 3.44] |  |
| CIN2 to CIN1 regression | 0.65  [0.46, 0.81] | 0.65  [0.46, 0.81] | 0.59  [0.43, 0.74] | 0.49  [0.35, 0.61] | 0.32  [0.23, 0.41] |  |
| CIN3 to CIN2 regression | 0.89  [0.75, 0.99] | 0.89  [0.75, 0.99] | 0.82  [0.69, 0.91] | 0.67  [0.56, 0.75] | 0.45  [0.38, 0.50] |  |

Table 18. Cervical cancer-associated mortality by cancer disease stage and CD4 count among women with HIV.

|  | **Annual cervical cancer-associated mortality rates**  **(per capita deaths per year)** | | | **References** |
| --- | --- | --- | --- | --- |
|  | Local | Regional | Distant |  |
| **HIV uninfected** | 0.0624 | 0.1502 | 0.5207 | ([91](#_ENREF_91), [92](#_ENREF_92)) |
| **HIV-positive on ART** | 0.2071 | 0.2418 | 0.5207 |  |
| **CD4 ≥500** | 0.2071 | 0.2418 | 0.5207 |  |
| **CD4 350-500** | 0.2515 | 0.2936 | 0.5207 |  |
| **CD4 250-350** | 0.3052 | 0.3563 | 0.5207 |  |
| **CD4 ≤250** | 0.4489 | 0.5240 | 0.5207 |  |

Table 19. Condom use.

| **Condom use in 2000 (x% of people * y% of the time)**  **Mean [Uncertainty Range]** | **References** |
| --- | --- |
| 0.28 [0.16, 0.40] | Calibrated. Values presented are the mean and range of the randomly sampled Phase 1 values corresponding to the 25 best-fitting parameter sets after Phase 2. Prior bounds were informed by data on self-reported condom use for contraception ([46](#_ENREF_46)). The lower bound uses data from married women, and the upper bound uses data from women aged 20-24, who were the most sexually active. |

Table 20. Proportion of men without HIV who receive voluntary male medical circumcision (VMMC).

| VMMC coverage among men without HIV over time by age group | | | | | **References** |
| --- | --- | --- | --- | --- | --- |
| Year | Age group | | | |  |
|  | 15-19 | 20-24 | 25-49 | 50-79 |  |
| 1960 | 0.040 | 0.060 | * | * | ([40](#_ENREF_40), [43-46](#_ENREF_43)) |
| 2000 | 0.100 | 0.130 | * | * |  |
| 2008 | 0.114 | 0.161 | * | * |  |
| 2010 | 0.143 | 0.201 | 0.140 | 0.120 |  |
| 2012 | 0.172 | 0.242 | 0.191 | 0.143 |  |
| 2017 | 0.459 | 0.420 | 0.318 | 0.204 |  |
| 2020 | 0.515 | 0.485 | 0.406 | 0.319 |  |
| 2030 | 0.700 | 0.700 | 0.700 | 0.700 |  |


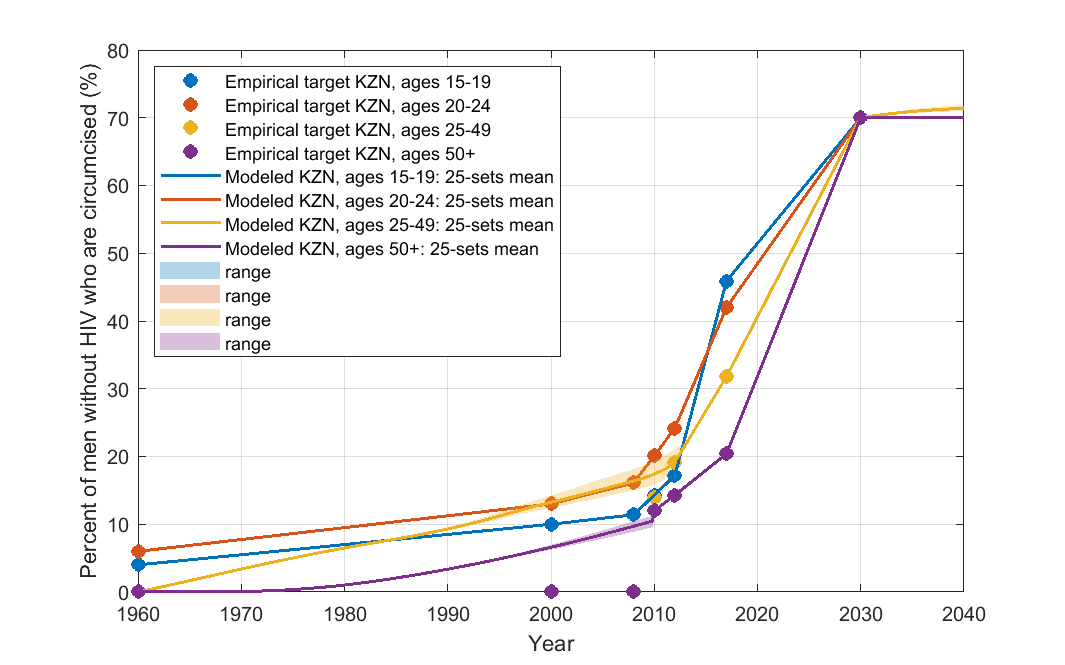
Figure 2. Percentage of men without HIV circumcised over time. Shaded regions represent the range of estimates using the 25 best-fitting model parameter sets.

Table 21. Proportion of persons with HIV and aged 10-14 or older who are on ART and virally suppressed in KwaZulu-Natal.

| Proportion virally suppressed over time by gender | | | **References** |
| --- | --- | --- | --- |
| Year | Men | Women |  |
| 2004 | 0 | 0 | ([45](#_ENREF_45), [59-62](#_ENREF_59)) |
| 2005 | 0.0057 | 0.0077 |  |
| 2006 | 0.0218 | 0.0294 |  |
| 2007 | 0.0476 | 0.0643 |  |
| 2008 | 0.0820 | 0.1108 |  |
| 2009 | 0.1152 | 0.1557 |  |
| 2010 | 0.1416 | 0.1913 |  |
| 2011 | 0.1760 | 0.2378 |  |
| 2017 | 0.4421 | 0.5975 |  |
| 2020 | 0.5083 | 0.6278 |  |
| 2030 | 0.7290 | 0.7290 | ([63](#_ENREF_63)) |


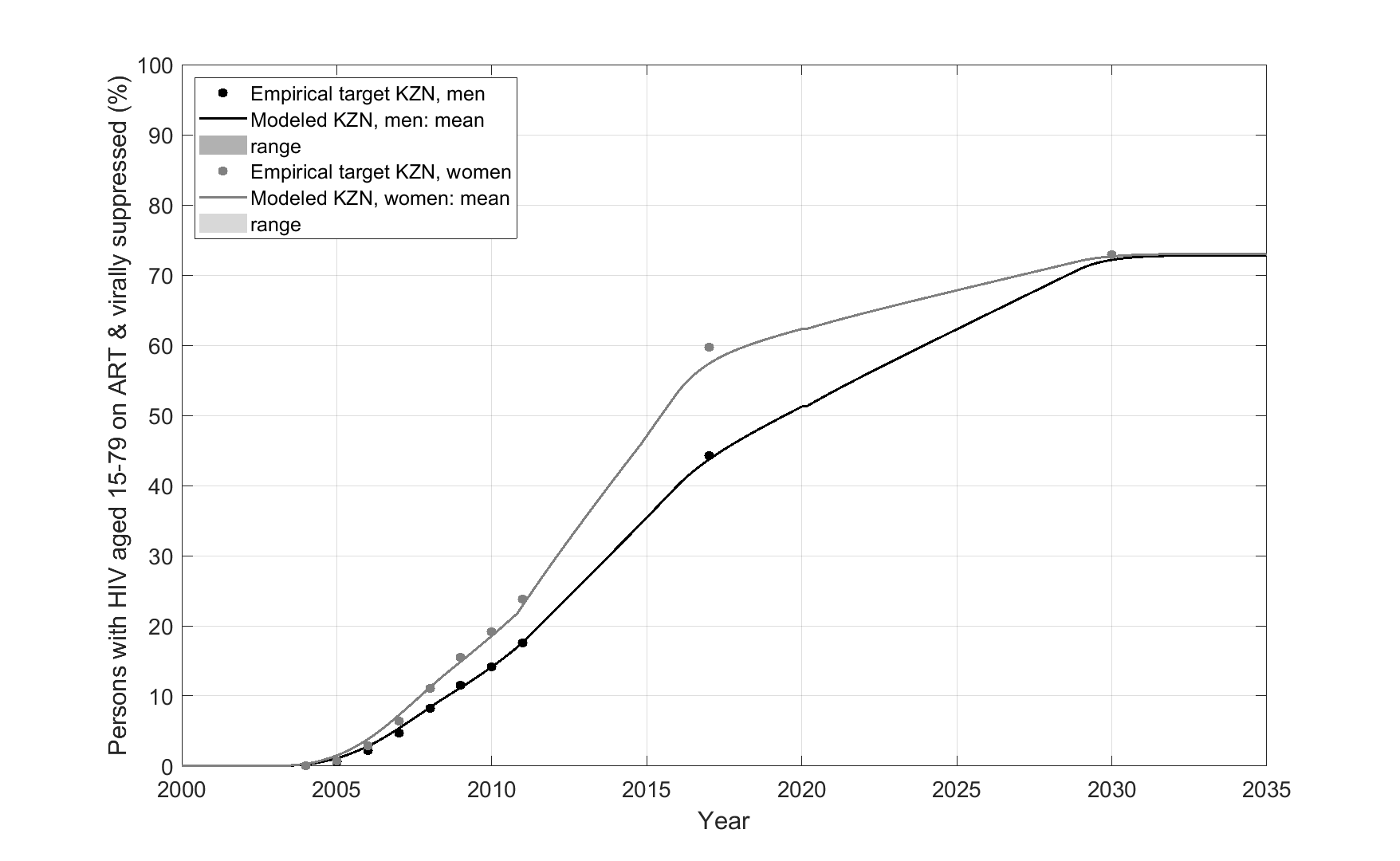


Figure 3. Proportion of persons with HIV on ART and virally suppressed by gender over time. Shaded regions represent the range of estimates using the 25 best-fitting model parameter sets.

Table 22. HIV-associated mortality after ART initiation. HIV-associated mortality among persons with viral suppression is assumed to decrease over time corresponding to earlier ART initiation and improved regimens. Excess HIV mortality among treated persons with HIV is defined relative to the background mortality (Table 4), such that, for instance, virally suppressed individuals from 2004-2011 have 1.5 times the all-cause risk of death relative to individuals without HIV.

| HIV-associated mortality among individuals with HIV viral suppression due to ART | | **References** |
| --- | --- | --- |
| Time period | Multiplier on background mortality rates listed in Table 4 |  |
| 2004 to 2011 | 0.5 | ([54-57](#_ENREF_54)) |
| 2011 to 2015 | 0.4 |  |
| 2015 to 2016 | 0.25 |  |
| After 2016 | 0.15 |  |

Table 23. Treatment cascade for women with CIN2+ or cervical cancer following screening in the *basecase* scenario. Numbers represent the proportion of women with true CIN2+ or cervical cancer who continue from the previous step to the designated step of the treatment cascade.

| Cascade step | CIN2+ | | Cervical cancer | References |
| --- | --- | --- | --- | --- |
|  | Women without HIV | Women with HIV |  |  |
| Correctly identified using cytology | 0.57 | 0.57 | 0.57 | ([2](#_ENREF_2), [3](#_ENREF_3), [76](#_ENREF_76)) |
| Retention to triage with colposcopic biopsy | 0.72 | 0.72 | 0.72 | ([77](#_ENREF_77)) |
| Proportion correctly identified with colposcopic biopsy | 1.0 | 1.0 | 1.0 | Assumed |
| Retention to treatment | 0.51 | 0.51 | 0.40 | ([77](#_ENREF_77)) |
| Treatment success | 0.91 | 0.77 | 1.0 | ([78](#_ENREF_78)) |
| HPV persistence after successful treatment | 0.185 | 0.185 | 0.0 | ([79](#_ENREF_79)) |

Table 24. Demography calibration targets. Calibrated in Phase 1.

| **Criteria** | **Gender** | **Year** | **Age group** | **Targets** | **Reference** |
| --- | --- | --- | --- | --- | --- |
| **Total population size** | Men and women combined | 2001, 2011, 2019 | 0-79 | Source provides number occurrences and N; used to calculate mean and variance | Statistics South Africa Census 2001 Post-enumeration survey, Table 2.7: Adjusted total population- full universe, for year 2001 ([93](#_ENREF_93)). Table 14: Adjusted total population- full universe, for years 2011 and 2019 ([94](#_ENREF_94)) |
| **Population age distribution** | Men and women combined | 1996, 2011, 2019 | By 5-year age group: 0-4 … 75-79 | Source provides number occurrences and N; used to calculate mean and variance | Statistics South Africa Primary tables Census '96 and 2001 compared, Table 4.1: KwaZulu-Natal, for year 1996 ([95](#_ENREF_95)). Statistics South Africa Statistical Release P0301 Community Survey 2016, Table 2.1: Census 2011 and Community Survey 2016, for year 2011 ([96](#_ENREF_96)). Statistics South Africa Statistical Release P0302 2019 Mid-year population estimates, Table 6 and Table 11, for year 2019 ([3](#_ENREF_3)). |

Table 25. HIV prevalence calibration targets. Calibrated in Phase 1.

| **Criteria** | **Gender** | **Year** | **Age group** | **Targets** | **Reference** |
| --- | --- | --- | --- | --- | --- |
| **HIV Prevalence among Men** | Men | 2003, 2005- 2009 | By 5-year age group: 15-19 … 45-49 | Source provides number cases and N; used to calculate mean and variance | ([12](#_ENREF_12)) |
| **HIV Prevalence among Women** | Women | 2003, 2005-2009 | By 5-year age group: 15-19 … 45-49 | Source provides number cases and N; used to calculate mean and variance | ([12](#_ENREF_12)) |

Table 26. HPV prevalence calibration targets. Calibrated in Phase 2.

| **Criteria** | **HIV status** | **Year** | **Age group** | **Targets** | **Reference** |
| --- | --- | --- | --- | --- | --- |
| **HPV Prevalence (including CIN) among Women** | Women with HIV, Women without HIV | 2002 | By 5-year age group except where specified: 17-19 , 20-24 … 50-54 , 55-65 | Source provides number cases and N; used to calculate mean and variance | ([97](#_ENREF_97)) |
| **HPV Prevalence among Men** | Men with HIV, Men without HIV | 2008 | 18-25, 26-35, 36-45, 46-66 | Source provides number cases and N; used to calculate mean and variance | ([98](#_ENREF_98)) |

Table 27. CIN prevalence calibration targets. Calibrated in Phase 2.

| **Criteria** | **HIV status** | **Year** | **Age group** | **Targets** | **Reference** |
| --- | --- | --- | --- | --- | --- |
| **CIN2/CIN3 Prevalence** | Women with HIV, Women without HIV | 2002 | By 5-year age group except where specified: 17-19 , 20-24 … 55-59, 60-65 | Source provides number cases and N; used to calculate mean and variance | ([97](#_ENREF_97)) |
| **CIN1 Prevalence** | Women with HIV, Women without HIV | 2015 | 30-65 | Source provides number cases and N; used to calculate mean and variance | ([99](#_ENREF_99)) |
| **CIN2 Prevalence** | Women with HIV, Women without HIV | 2015 | 30-65 | Source provides number cases and N; used to calculate mean and variance |  |
| **CIN3 Prevalence** | Women with HIV, Women without HIV | 2015 | 30-65 | Source provides number cases and N; used to calculate mean and variance |  |

Table 28. Cervical cancer incidence calibration targets. Calibrated in Phase 2.

| **Criteria** | **Year** | **Age group** | **Targets** | **Reference** |
| --- | --- | --- | --- | --- |
| **Cervical cancer incidence per 100K women** | 2018 | By 5-year age group: 15-19 … 70-74 | Source provides mean values. To approximate cervical cancer incidence in KwaZulu-Natal rather than South Africa nationally, we adjusted the Globocan 2018 rates by age to take into account higher HIV prevalence in KwaZulu-Natal. We assumed women with HIV have four times increased risk of cervical cancer ([38](#_ENREF_38)) and then reweighted the overall cervical cancer incidence rate according to the HIV prevalence in KwaZulu-Natal compared to South Africa nationally. HIV prevalence data in older ages are sparse, so we assumed prevalence is increased by the same proportion in women aged 50+. | ([38](#_ENREF_38), [100](#_ENREF_100)) |
|  |  |  |  |  |
|  |  |  |  |  |
|  |  |  |  |  |
|  |  |  |  |  |
|  |  |  |  |  |
|  |  |  |  |  |
|  |  |  |  |  |
|  |  |  |  |  |
|  |  |  |  |  |
|  |  |  |  |  |
|  |  |  |  |  |

Table 29. HPV type distribution calibration targets. Calibrated in Phase 2. Used to guide calibration of progression, regression, and clearance parameters for nonavalent-vaccine-type and non-vaccine-type infections. The type distribution for CIN2 was calculated as the average of the distributions for CIN1 and CIN3.

| **Criteria** | **HPV type** | **Year** | **Age group** | **Targets** | **Reference** |
| --- | --- | --- | --- | --- | --- |
| **HPV** | Vaccine-type HPV | 2011-2015 | 0-79 | Proportions of nonavalent vaccine-type HPV and non-vaccine-type HPV were averaged across several studies. | ([28](#_ENREF_28), [35](#_ENREF_35)) |
|  | Non-vaccine-type HPV |  |  |  |  |
| **CIN1** | Vaccine-type HPV | 2011-2015 | 0-79 |  | ([28](#_ENREF_28), [34](#_ENREF_34)) |
|  | Non-vaccine-type HPV |  |  |  |  |
| **CIN3** | Vaccine-type HPV | 2011-2015 | 0-79 |  | ([28](#_ENREF_28), [32](#_ENREF_32), [33](#_ENREF_33)) |
|  | Non-vaccine-type HPV |  |  |  |  |
| **Cervical cancer** | Vaccine-type HPV | 2011-2015 | 0-79 |  | ([28-31](#_ENREF_28)) |
|  | Non-vaccine-type HPV |  |  |  |  |

Table 30. HIV prevalence validation targets.

| **Criteria** | **Gender** | **Year** | **Age group** | **Targets** | **References** |
| --- | --- | --- | --- | --- | --- |
| **HIV Prevalence among Men** | Men | 2010-2016 | By 5-year age group: 15-19 … 45-49 | Source provides number cases and N; used to calculate mean and variance | ([12](#_ENREF_12)) |
| **HIV Prevalence among Women** | Women | 2010-2016 | By 5-year age group: 15-19 … 45-49 | Source provides number cases and N; used to calculate mean and variance | ([12](#_ENREF_12)) |
| **HIV Prevalence, ages 15-49** | Men and women combined | 2010-2016 | 15-49 | Calculated from the number cases and N provided by 5-year age groups | ([12](#_ENREF_12)) |
| **HIV Prevalence, ages 15-49** | Men and women combined | 2002, 2005, 2008, 2012, 2017 | 15-49 | Source provides mean and 95% confidence interval | ([45](#_ENREF_45)) |
| **HIV Prevalence, ages 25+** | Men and women combined | 2002, 2005, 2008, 2012, 2017 | 25+ | Source provides mean and 95% confidence interval | ([45](#_ENREF_45)) |
| **HIV Prevalence, ages 50+** | Men and women combined | 2002, 2005, 2008, 2012, 2017 | 50+ | Source provides mean and 95% confidence interval | ([45](#_ENREF_45)) |

Table 31. HIV incidence validation targets.

| **Criteria** | **Year** | **Age group** | **Targets** | **References** |
| --- | --- | --- | --- | --- |
| **Men aged 15-29,**  **HIV incidence** | 2005-2017 | 15-29 | Source provides mean and 95% confidence interval | ([83](#_ENREF_83)) |
| **Men aged 30-54,**  **HIV incidence** | 2005-2017 | 30-54 | Source provides mean and 95% confidence interval |  |
| **Men aged 15-54,**  **HIV incidence** | 2005-2017 | 15-54 | Source provides mean and 95% confidence interval |  |
| **Women aged 15-29,**  **HIV incidence** | 2005-2017 | 15-29 | Source provides mean and 95% confidence interval |  |
| **Women aged 30-49,**  **HIV incidence** | 2005-2017 | 30-49 | Source provides mean and 95% confidence interval |  |
| **Women aged 15-49, HIV incidence** | 2005-2017 | 15-49 | Source provides mean and 95% confidence interval |  |

**7) Description of analysis**

We projected cervical cancer incidence over the next century with the assumption that sexual behaviour parameters will stay constant. We model a linear decline in fertility rates by 50% from 2020 to 2035 to match projected United Nations Population Division estimates for population size, age distribution, and total fertility (4,5). We assumed that KwaZulu-Natal achieves the UNAIDS 90-90-90 targets (90% of persons with HIV diagnosed, 90% of persons diagnosed receive ART, and 90% of persons on ART have viral suppression([63](#_ENREF_63))) by 2030. To achieve this, the percentage of persons with HIV who are treated with viral suppression was assumed to increase linearly from 2020 levels to 72.9% among both men and women by 2030. The proportion of men aged 15-79 who receive VMMC also increased linearly from 2020 coverage levels to 70% by 2030.

We calculated the median cervical cancer incidence over time by age group from simulations using the 25 best-fitting model parameter sets. We then used these crude median age group-specific rates to calculate age-standardied incidence, using the 2015 World Standard Population aged 0-99. In a similar process, we obtained the 5th and 95th percentiles of cervical cancer incidence over time by age group from simulations using the 25 best-fitting model parameter sets, which we used to calculate lower and upper bounds for the uncertainty interval of age-standardised cervical cancer incidence over time. We calculated the percent reduction in age-standardised cervical cancer incidence over time by taking the difference between the simulated estimates for each scenario and the corresponding estimates from the *basecase* scenario run, dividing by the *basecase* estimates, and multiplying by 100. We carried all 25 best-fitting parameter sets through the age-standardised percent reduction calculation and then calculated the median, 5th, and 95th percentiles of the percent reduction to capture uncertainty in our predictions.

In scenarios with routine vaccination (targeting girls aged 9-14), we vaccinated the number of girls necessary to reach the specified coverage level in these ages, and vaccination status was tracked as girls aged through the model. In scenarios with catch-up vaccination of women with HIV, we modelled vaccination of young women aged 15-24 to reach the given coverage level. Most catch-up vaccinations therefore occurred in the first ten years of the simulation, before girls who received routine vaccination aged into the age groups designated for catch-up.

Beginning in 2000, we modelled once-per-lifetime cervical cancer screening for women in the age range of 35-39, screening the number of women necessary in the age group to reach the specified coverage level. We applied screening coverage uniformly across the 5-year age group so that the expected proportion of women aging into the next age group had a history of cervical cancer screening. This effectually accelerated and overestimated the impact of the screening intervention by four years relative to a scenario in which screening is targeted to women at a single age. Another effect of our full age-group screening approach was that in scenarios with a more efficacious treatment cascade beginning in 2020, previously-screened women who had experienced a less effective treatment cascade needed to cycle through the age group for the intervention to have a full effect. In scenarios with twice-per-lifetime screening beginning in 2020, the full impact of the screening and treatment cascade was observed immediately for women aged 45-49, as women were not previously screened in this age group, and the model had no memory of screening history once a women aged out of an actively screened age group. This resulted in a sharp decrease in simulated cervical cancer incidence from 2020 to 2021. In scenarios with screening every three years for women with HIV, we applied the given screening coverage to each 5-year age group containing an age recommended for screening (i.e., women aged 25-29, 30-34, 35-39, 40-44, 45-49). Therefore, as a result of our model structure with 5-year age groups, we model screening every 5 years in scenarios with enhanced screening for women with HIV. We observed the most dramatic impact on cervical cancer incidence in year-one of these scenarios as multiple age groups were newly screened and treated.

**8) Fitting and validation results**

*8.1) Demography*


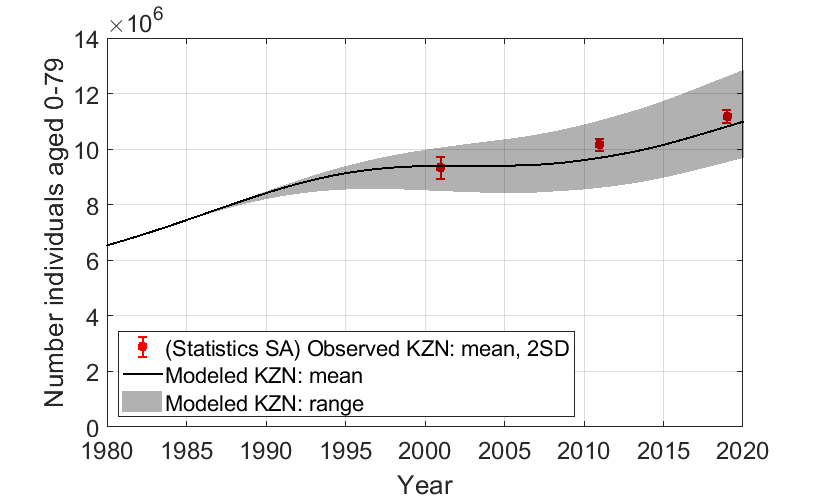


Figure 4. Model fit to observed total population size data over time. Standard deviation of the observed data calculated from the given absolute error assuming that the total population size follows a normal distribution. Shaded region represents the range of estimates using the 25 best-fitting model parameter sets.


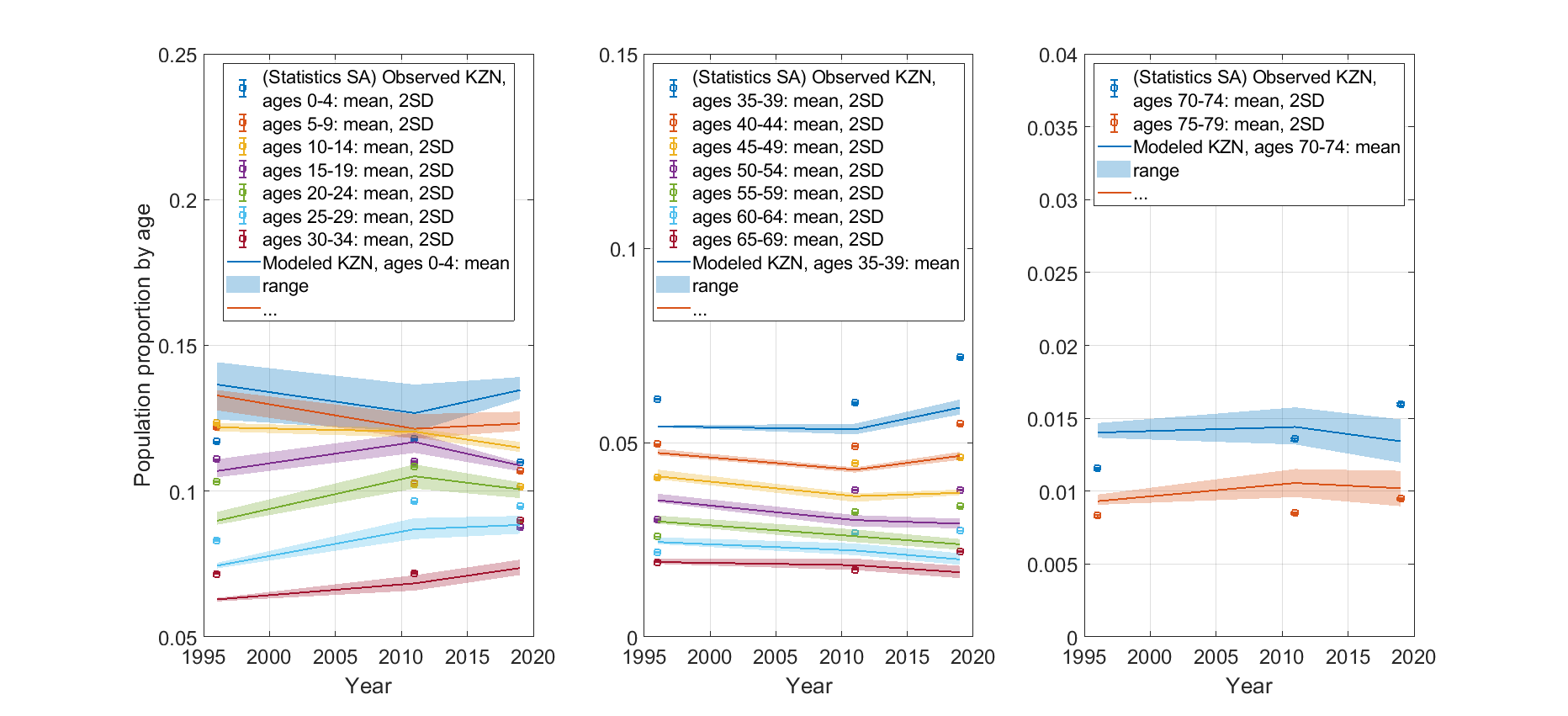


Figure 5. Model fit to observed population age distribution data over time. Standard deviation of the observed data calculated assuming that population proportions follow a normal approximation of the binomial distribution. Shaded regions represent the range of estimates using the 25 best-fitting model parameter sets.

8.2) HIV epidemiology


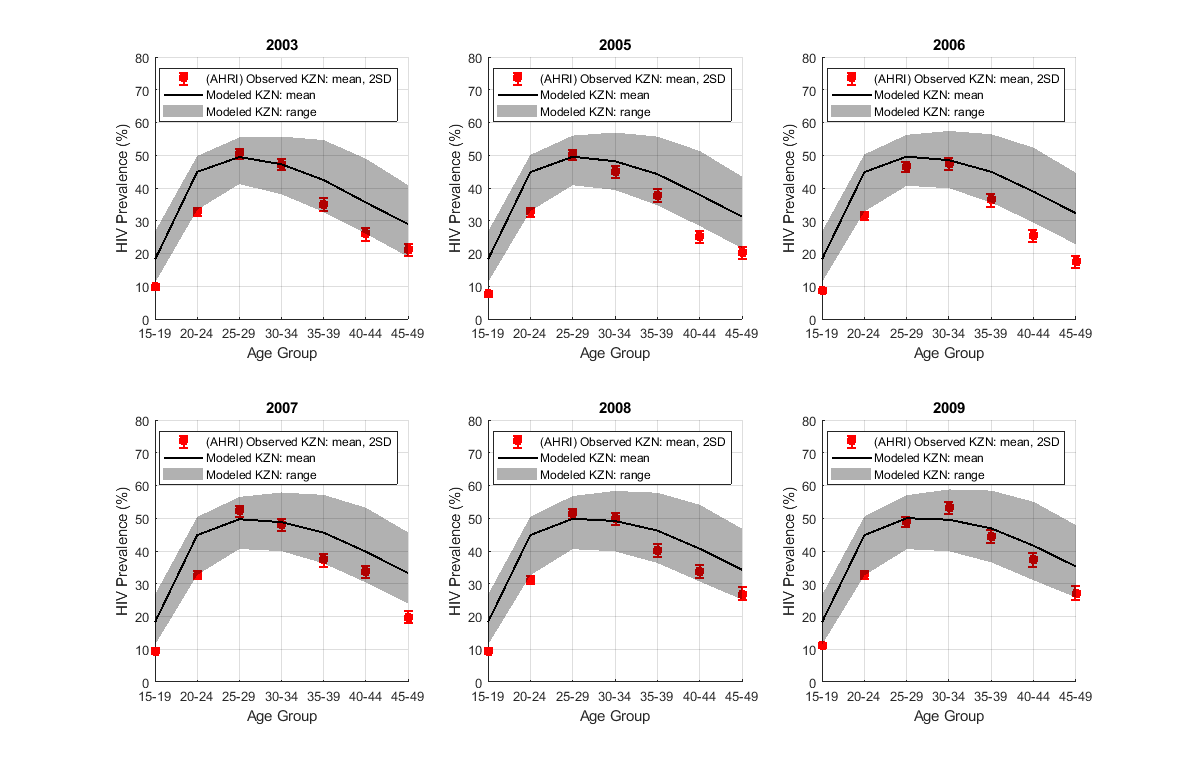


Figure 6. Model fit to observed HIV prevalence data in women by age over time. Standard deviation of the observed data calculated assuming that prevalence proportions follow a normal approximation of the binomial distribution. Shaded regions represent the range of estimates using the 25 best-fitting model parameter sets.


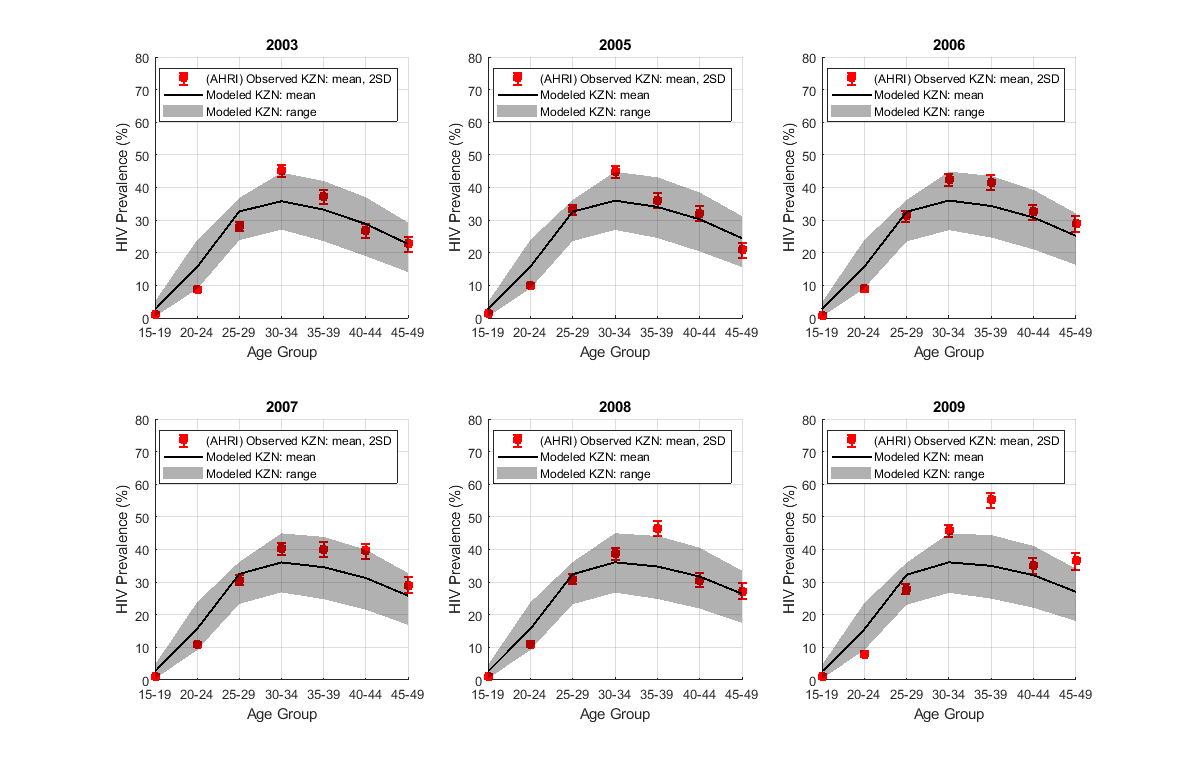


Figure 7. Model fit to observed HIV prevalence data in men by age over time. Standard deviation of the observed data calculated assuming that prevalence proportions follow a normal approximation of the binomial distribution. Shaded regions represent the range of estimates using the 25 best-fitting model parameter sets.

*8.3) HPV and Cervical cancer epidemiology*


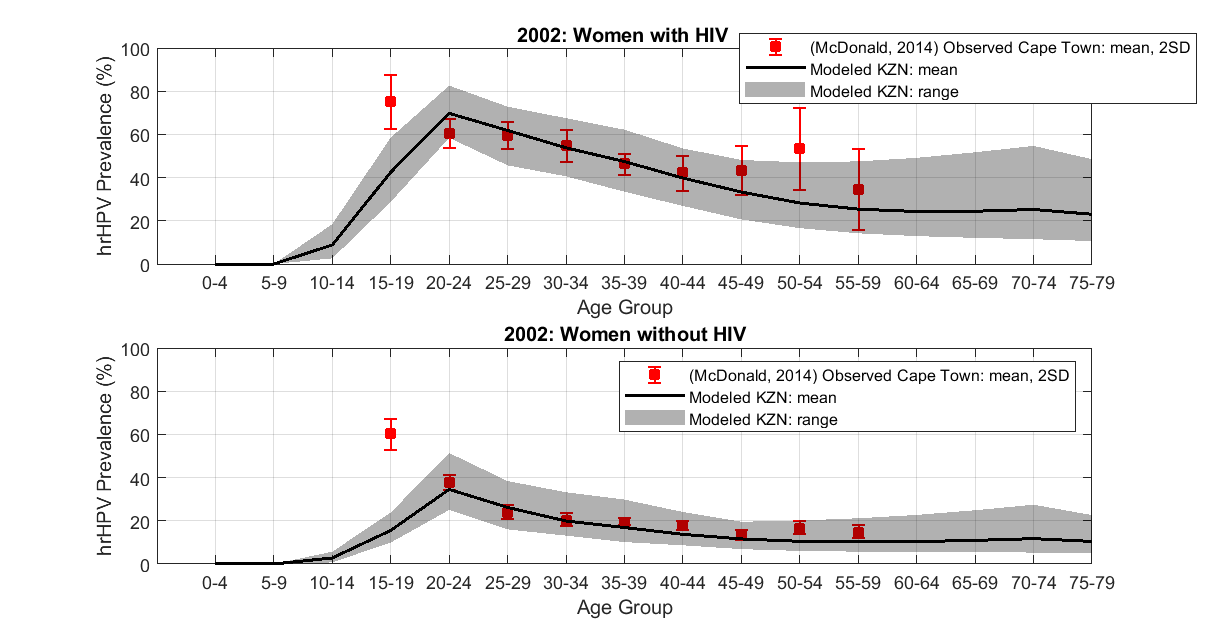
Figure 8. Model fit to observed HPV prevalence data in women by age and HIV status in 2002. Standard deviation of the observed data calculated assuming that prevalence proportions follow a normal approximation of the binomial distribution. Shaded regions represent the range of estimates using the 25 best-fitting model parameter sets.


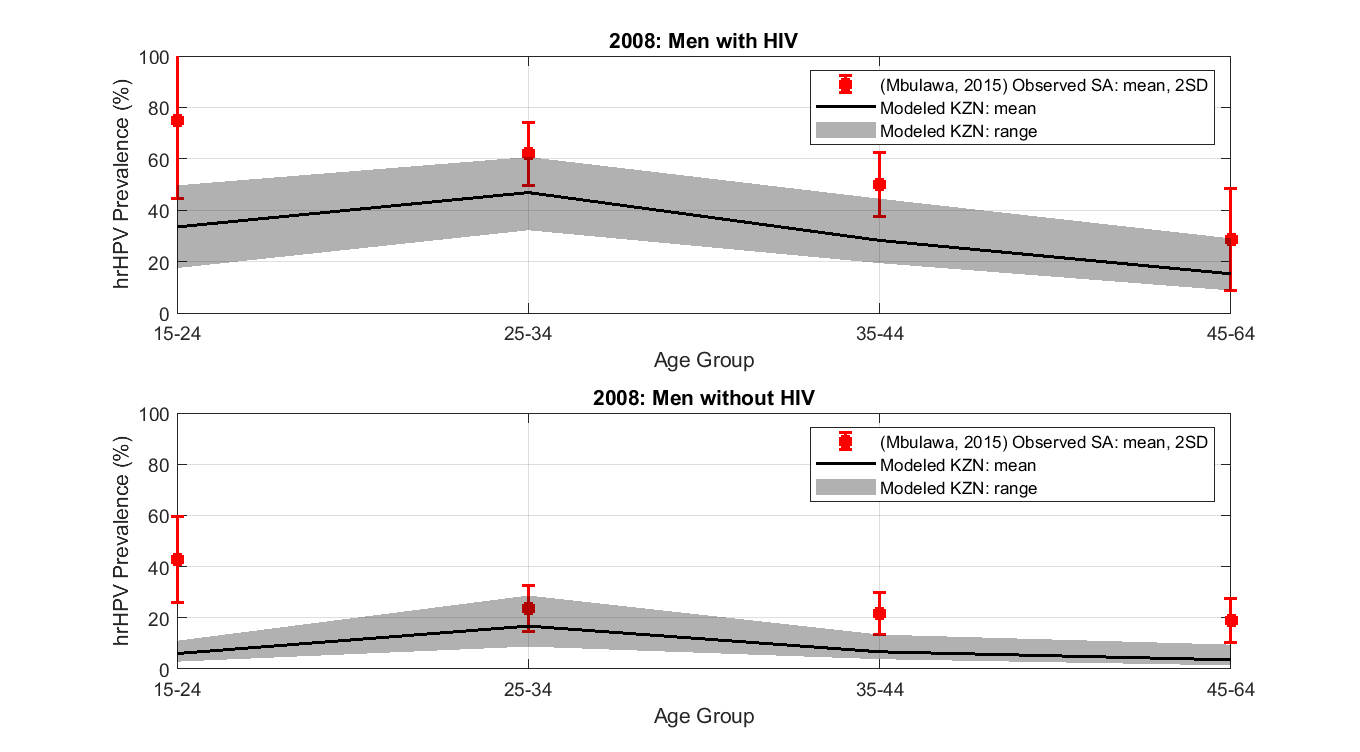
Figure 9. Model fit to observed HPV prevalence data in men by age and HIV status in 2008. Standard deviation of the observed data calculated assuming that prevalence proportions follow a normal approximation of the binomial distribution. Shaded regions represent the range of estimates using the 25 best-fitting model parameter sets.


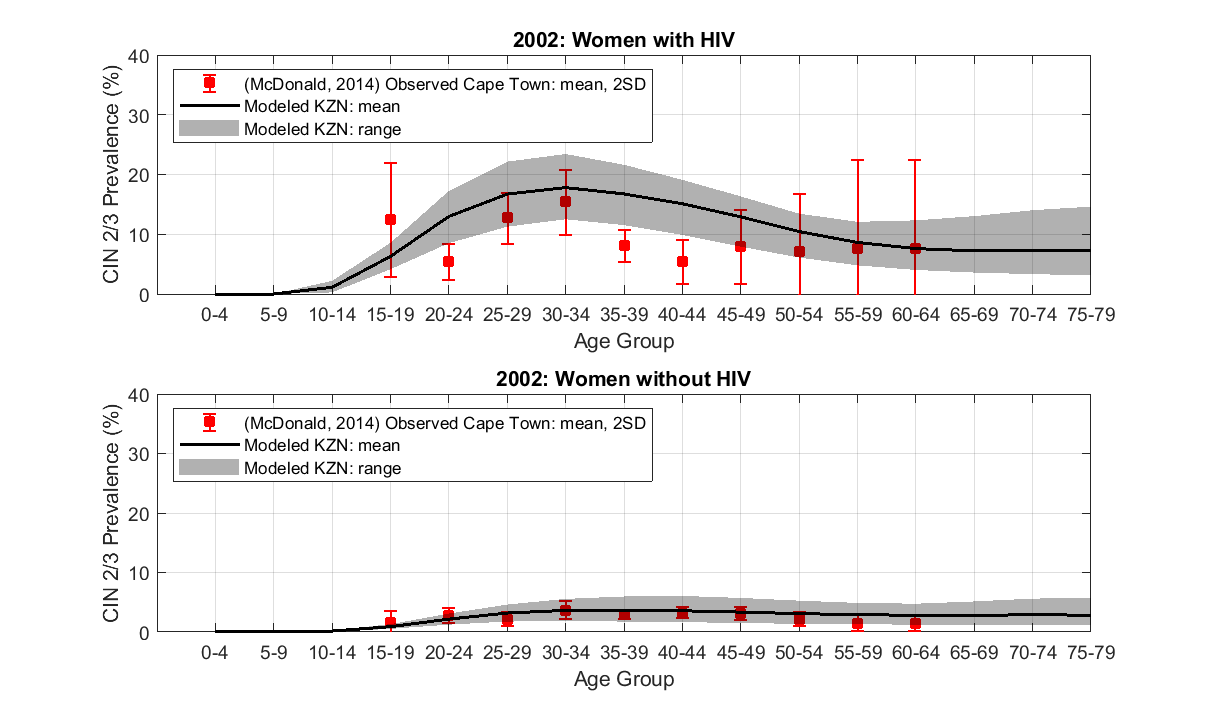


Figure 10. Model fit to observed CIN2/3 prevalence data by age and HIV status in 2002. Standard deviation of the observed data calculated assuming that prevalence proportions follow a normal approximation of the binomial distribution. Shaded regions represent the range of estimates using the 25 best-fitting model parameter sets.


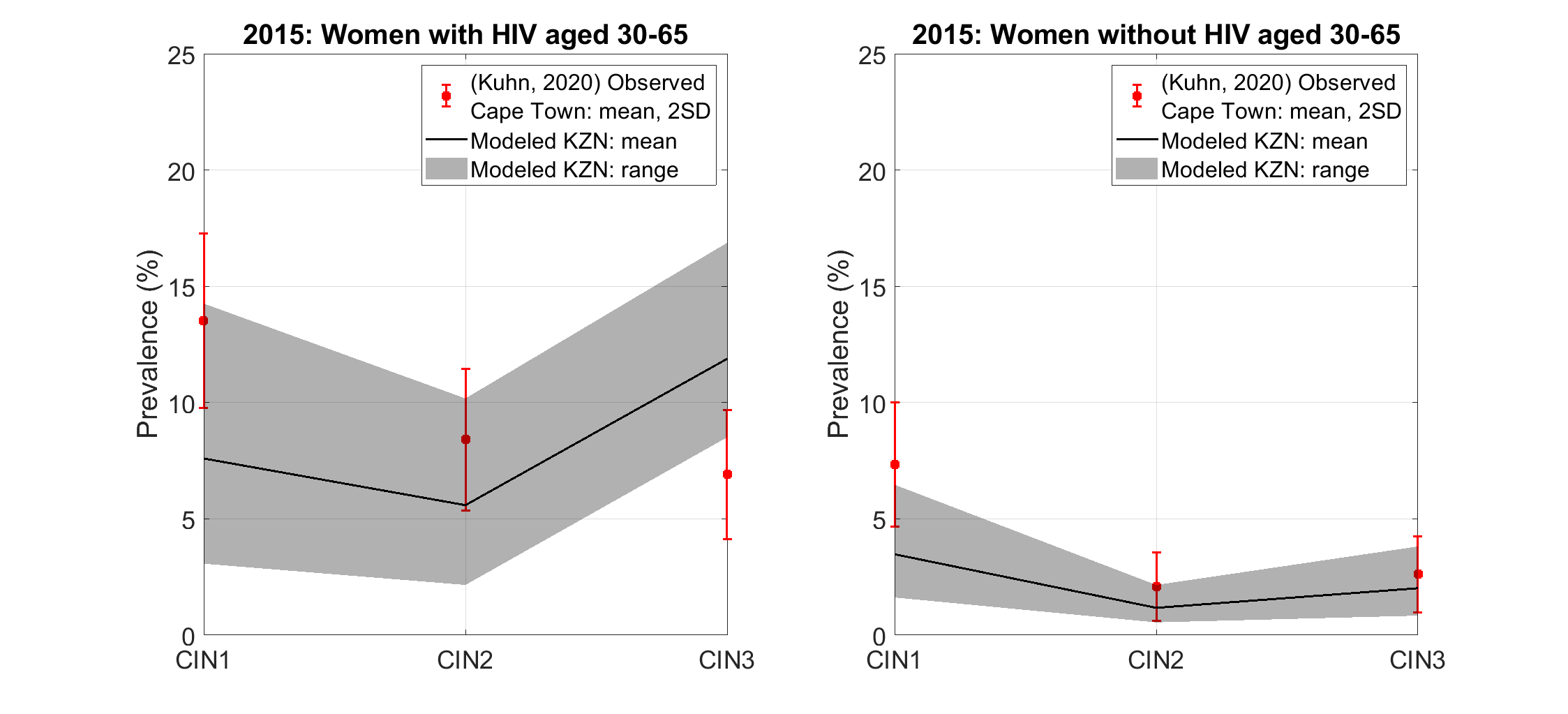
Figure 11. Model fit to observed prevalence data by CIN stage and HIV status in 2015. Standard deviation of the observed data calculated assuming that prevalence proportions follow a normal approximation of the binomial distribution. Shaded regions represent the range of estimates using the 25 best-fitting model parameter sets.


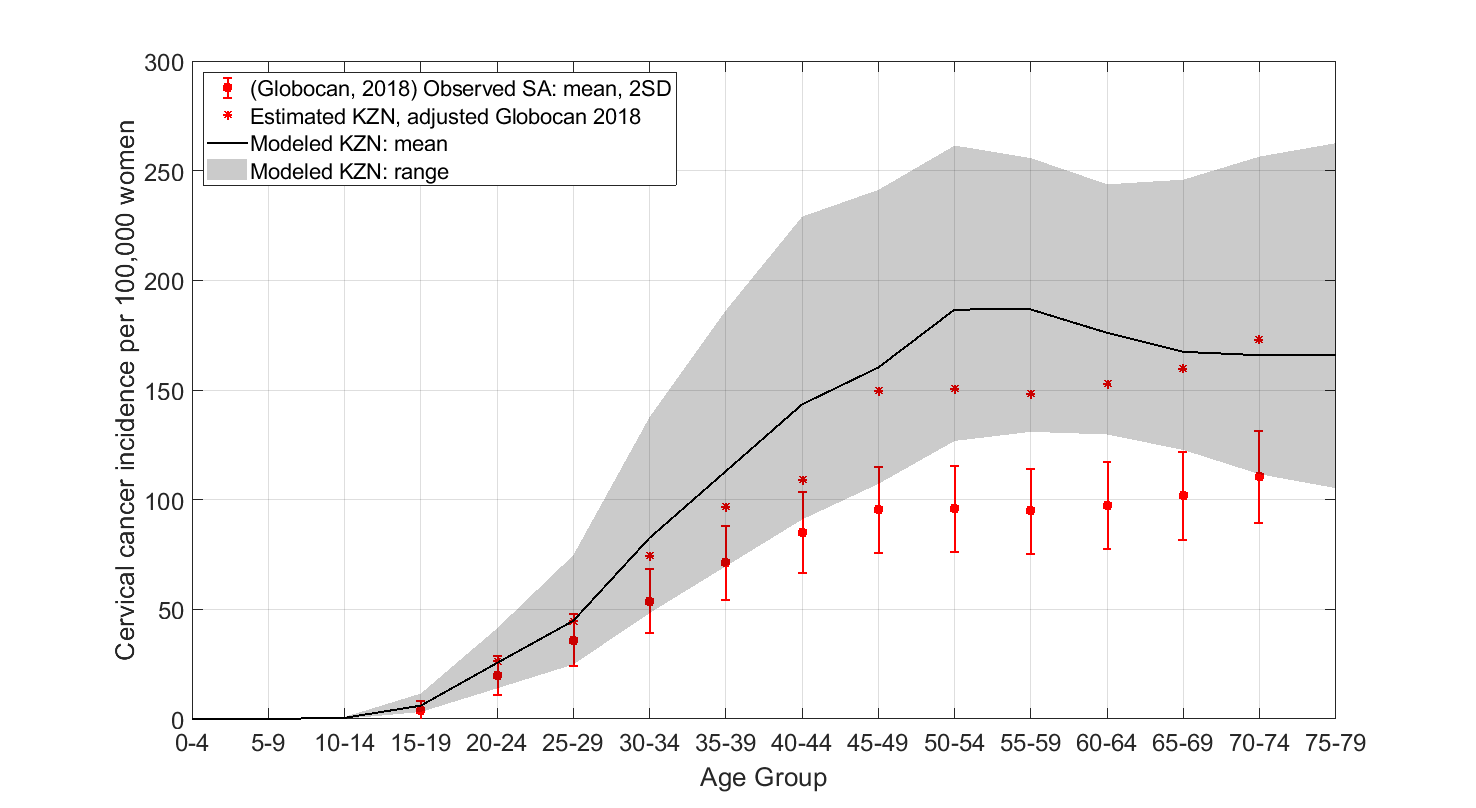


Figure 12. Model fit to observed cervical cancer incidence data by age in 2018. Asterisks represent South Africa Globocan 2018 rates adjusted to take into account higher HIV prevalence in KwaZulu-Natal. Standard deviation of the observed data calculated assuming that incidence follows a normal approximation of the Poisson distribution. Shaded region represents the range of estimates using the 25 best-fitting model parameter sets.


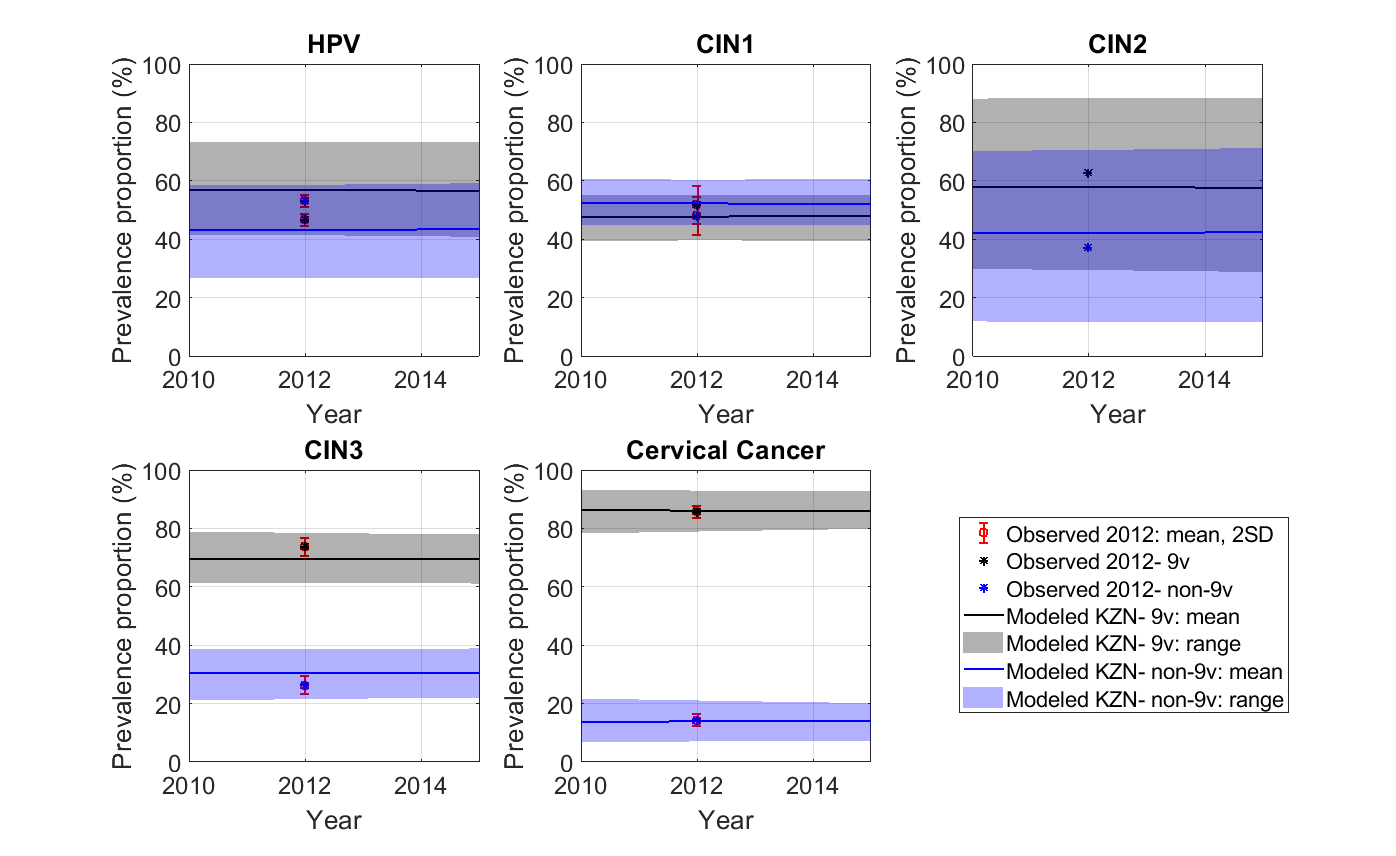


Figure 13. Model fit to observed type distribution data by state in 2012 among all ages. Vaccine-type (9v) and non-vaccine type (non-9v) HPV coinfections are classified as vaccine-type HPV. The type distribution for CIN2 was not fit to observed data, but is displayed relative to the average of observed CIN1 and CIN3 data. Standard deviation of the observed data calculated assuming that prevalence proportions follow a normal approximation of the binomial distribution. Shaded regions represent the range of estimates using the 25 best-fitting model parameter sets.

*8.4) Validation results*

To verify our ability to accurately predict future outcomes, we cross-validated our HIV natural history module by comparing model HIV prevalence and HIV incidence predictions to additional time points and data sources.


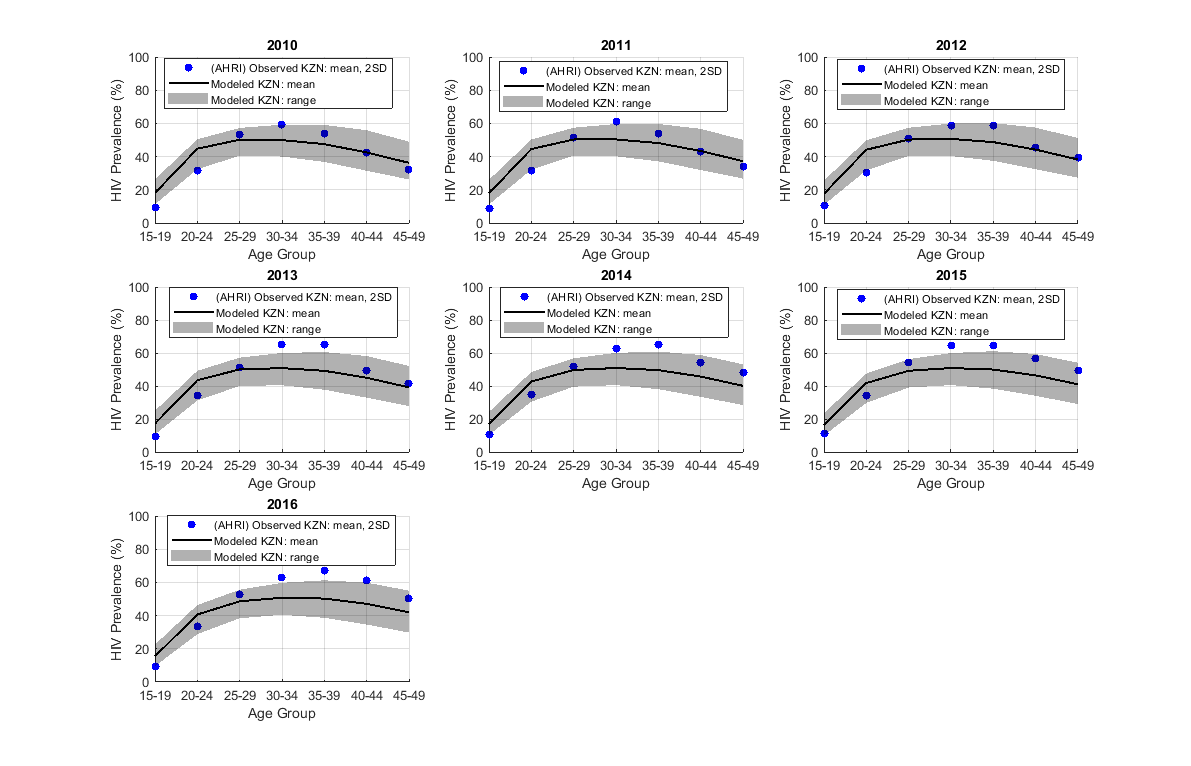


Figure 14. Model validation to observed HIV prevalence data in women by age over time. Red indicates calibration data for reference, and blue indicates validation data. Shaded regions represent the range of estimates using the 25 best-fitting model parameter sets.


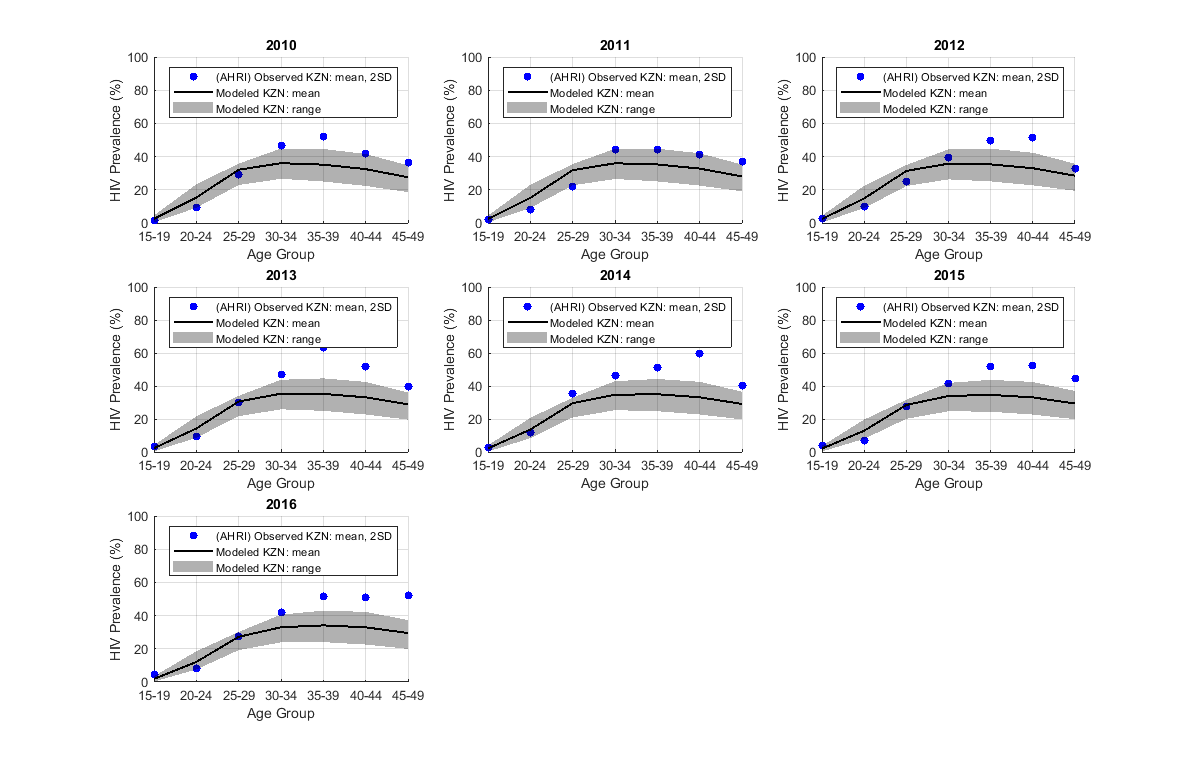


Figure 15. Model validation to observed HIV prevalence data in men by age over time. Red indicates calibration data for reference, and blue indicates validation data. Shaded regions represent the range of estimates using the 25 best-fitting model parameter sets.


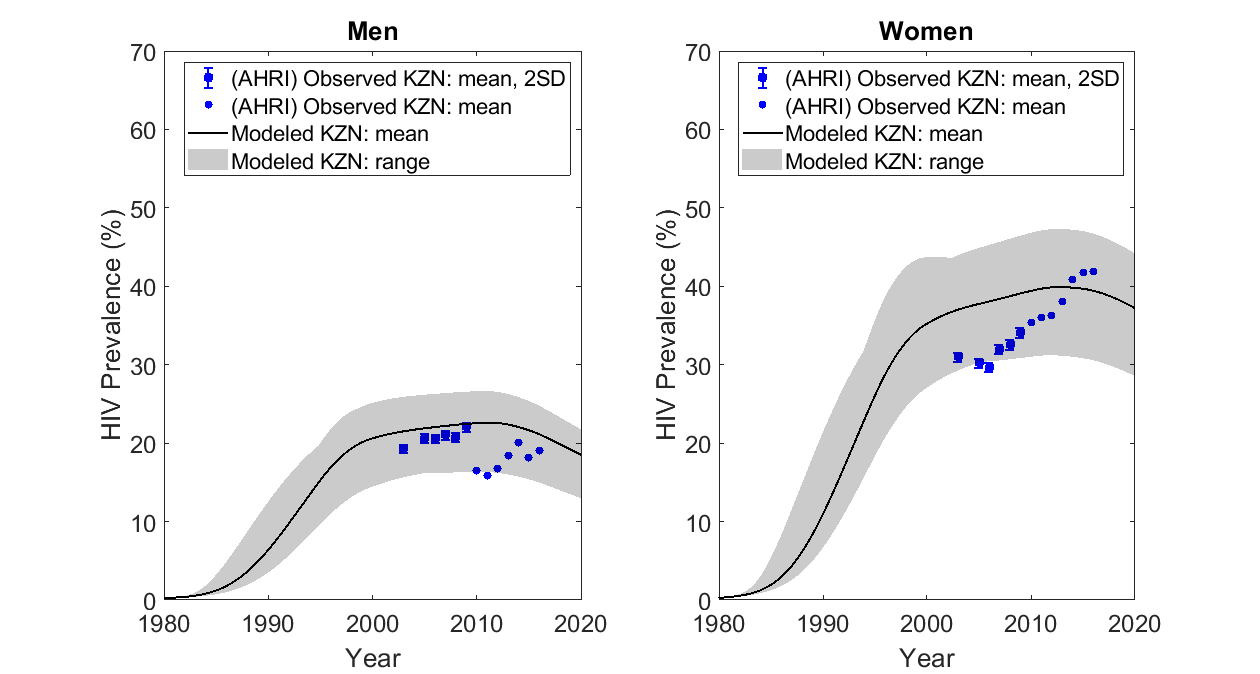


Figure 16. Model validation to observed HIV prevalence data by gender over time. Blue error bars from 2003-2009 recalculate HIV prevalence for ages 15-49 combined from the calibration dataset, while blue data from 2010-2016 represents later timepoints used only for validation. Shaded regions represent the range of estimates using the 25 best-fitting model parameter sets.


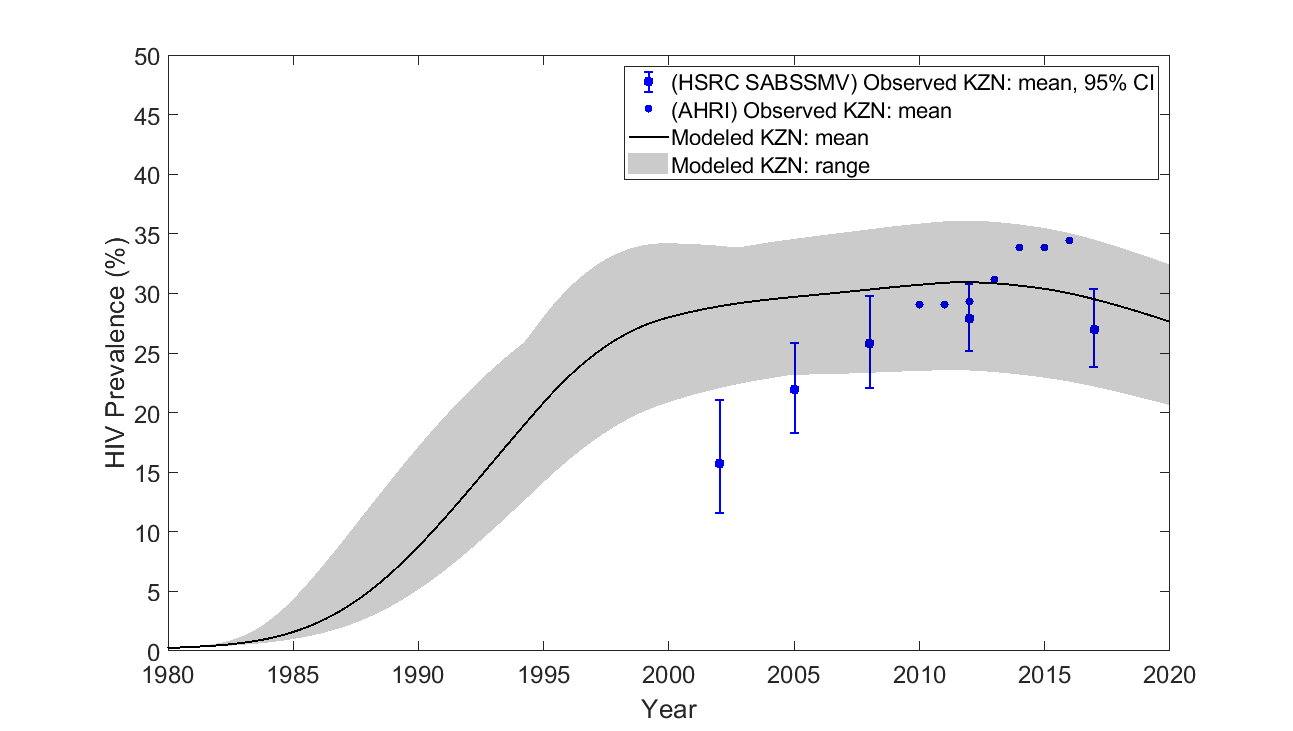


Figure 17. Model validation to observed HIV prevalence data among ages 15-49 over time. Shaded region represents the range of estimates using the 25 best-fitting model parameter sets.


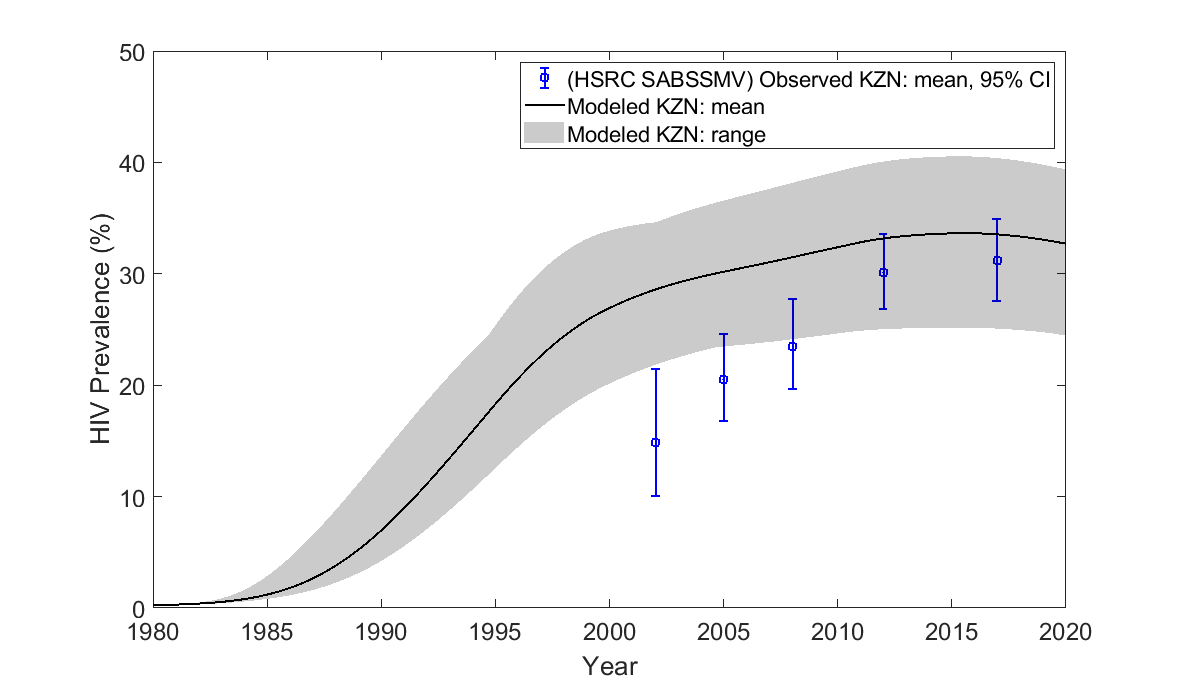


Figure 18. Model validation to observed HIV prevalence data among ages 25+ over time. Shaded region represents the range of estimates using the 25 best-fitting model parameter sets.


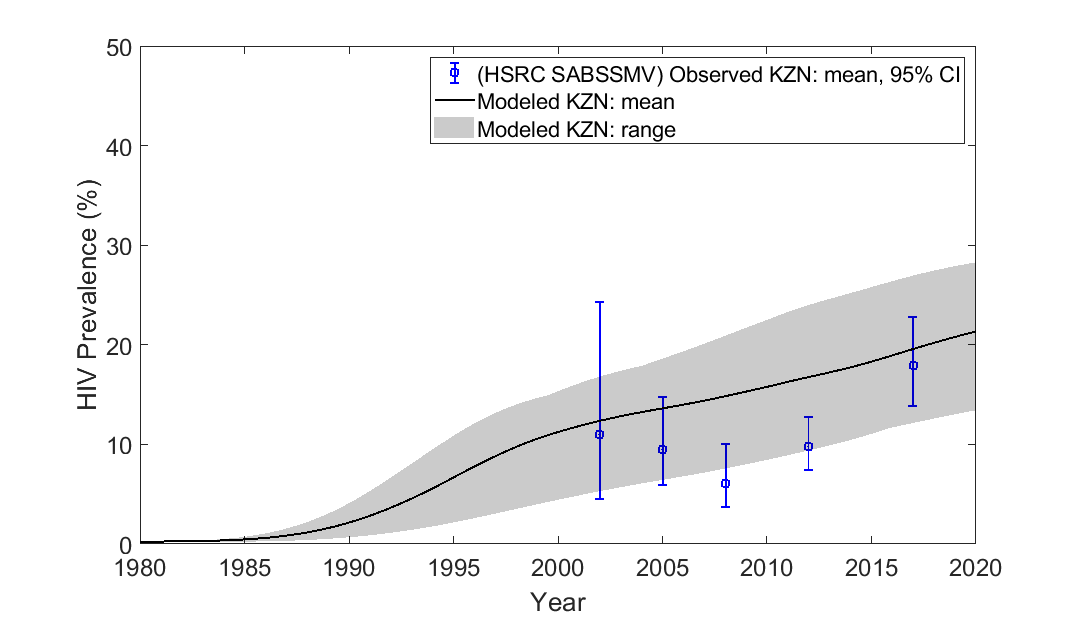


Figure 19. Model validation to observed HIV prevalence data among ages 50+ over time. Shaded region represents the range of estimates using the 25 best-fitting model parameter sets.

 
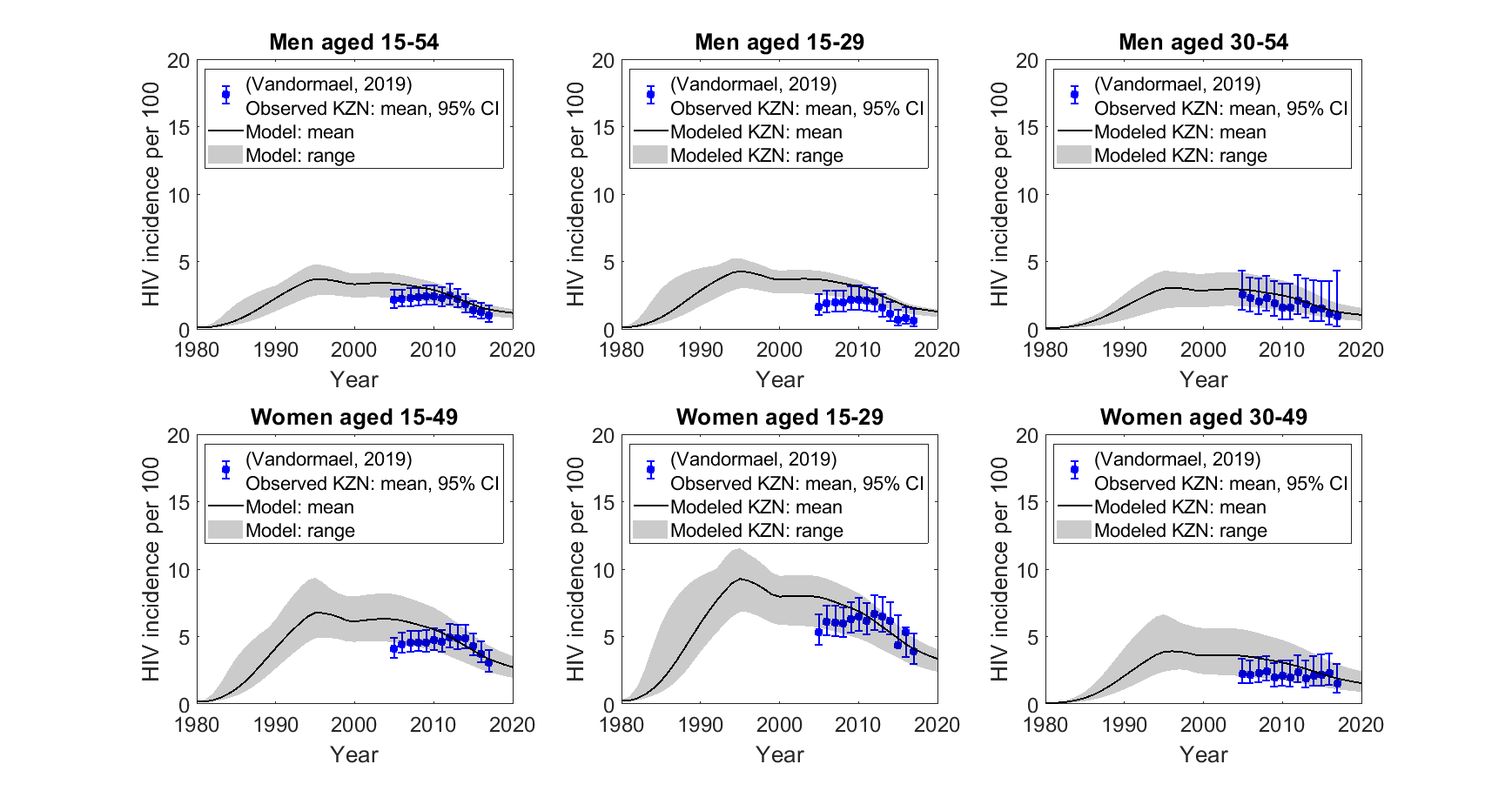


Figure 20. Model validation to observed HIV incidence data by gender and age over time. Shaded regions represent the range of estimates using the 25 best-fitting model parameter sets.

**9) Model equations**

We use a system of differential equations to estimate changes in population and infection dynamics over each time step. We split the full system of equations into topic-based modules and solve each iteratively using a 4^th^-order Runge-Kutta numerical solver in MATLAB. The order is largely historical and based on the work of (Tan et al., 2018)([1](#_ENREF_1)). The modules are:

1. HPV natural history
   1. Progression and clearance of HPV
   2. Progression and regression of precancerous lesions
   3. Development and progression of cervical cancer
   4. Cervical cancer- associated mortality
2. Cervical cancer screening and treatment
   1. Screening
   2. Treatment
3. HPV and HIV transmission
   1. Heterosexual mixing by gender, age, and risk group
   2. Partnership adjustment
   3. HPV infection by type
   4. HIV infection
4. HIV natural history and treatment
   1. CD4 progression
   2. Viral load progression
   3. ART initiation, discontinuation, and scale-up by CD4 count
   4. HIV-associated mortality
5. Demography
   1. Births
   2. Mother-to-child HIV transmission
   3. Aging and risk-group redistribution
   4. Natural deaths
6. Voluntary male medical circumcision
7. HPV vaccination
   1. School-based regimen
   2. Catch-up regimen

Ideally, each module would be completely independent from the others. This may not be the case here, but a comparative analysis revealed no significant difference in the results of split or combined approaches. We therefore chose the split approach for efficiency as it allows us to use a larger time step.

Throughout each simulation, we track population demographics and the number of persons with infection, with progressed disease, or with preventative or therapeutic treatment. We describe these states $X_{g,a,r}^{d,v,h,s,x,p}(t)$ with the following indices (using 1-based indexing):

| **Index** | **Description** | **Values** |
| --- | --- | --- |
| *d* | HIV disease state, CD4 count, circumcision status, and ART status | 1. HIV-negative, uncircumcised 2. HIV-negative, circumcised 3. HIV-positive, acute infection 4. HIV-positive, CD4 > 500 cells/µL 5. HIV-positive, CD4 350-500 cells/µL 6. HIV-positive, CD4 200-350 cells/µL 7. HIV-positive, CD4 ≤ 200 cells/µL 8. HIV-positive, on ART |
| *v* | HIV viral load | 1. If ( 2 ˂ *d* ˂ 8 ), Acute infection; if ( *d* = 1,2 ), HIV-negative: VL = 0.0 2. Asymptomatic: VL = 3.0-4.5 log_10_ 3. Pre-AIDS symptomatic: VL = 4.0-5.5 log_10_ 4. AIDS: VL = 5.5-7.0 log_10_ 5. Late-stage 6. On ART and virally suppressed: VL = 0.0 |
| *h* | Vaccine-type HPV precancer or disease state | 1. Susceptible 2. Infected 3. CIN1 4. CIN2 5. CIN3 6. Cervical cancer or hysterectomy 7. Immune |
| *s* | Non-vaccine type HPV precancer or disease state | 1. Susceptible 2. Infected 3. CIN1 4. CIN2 5. CIN3 6. Cervical cancer or hysterectomy 7. Immune |
| *x* | Cervical cancer or hysterectomy status | 1. If ( *h* = 6 or *s* = 6 ), Cervical cancer, local; else, no cancer or hysterectomy 2. Cervical cancer, regional 3. Cervical cancer, distant 4. Hysterectomy |
| *p* | Vaccination and screening history | 1. Non-vaccinated, non-screened 2. Vaccinated 3. Screened 4. Vaccinated and screened |
| *g* | Gender | 1. Men 2. Women |
| *a* | Age | 1. 0-4 2. 5-9 3. 10-14 4. 15-19 5. 20-24 6. 25-29 7. 30-34 8. 35-39 9. 40-44 10. 45-49 11. 50-54 12. 55-59 13. 60-64 14. 65-69 15. 70-74 16. 75-79 |
| *r* | Risk | 1. Low risk 2. Moderate risk 3. High risk |

*9.1 Demography*

| **Equation variables** | |
| --- | --- |
| $\gamma_{a}^{d}$(t) | The annual fertility rate for women by age *a* and HIV disease stage *d*.  Women aged 15-49 bear children. |
| $\eta\left( t \right)$ | The proportion of births from women living with HIV that result in vertical transmission. |
| $b\_s\left( t \right)$ | Number of births by HIV-negative women and women on ART. |
| $b\_i\left( t \right)$ | Number of births by women living with HIV. |
| $b_{g,a,r}^{d,1,h,s,x,p}(t)$ | Number of infant births of HIV disease stage *d* and gender *g.*  We assume an equal gender ratio at birth of 1:1, that all newborns are born as low risk, no vertical transmission of HPV, and that if HIV is vertically transmitted, that infected newborns are born into the acute stage of HIV. |
| $\phi_{g,a,r}$ | Distribution of sexual risk *r* by gender *g* and age *a.* (Currently, the risk distribution derived from male partner data is used for both men and women for simplicity.) |
| ${\mu\_bkrd}_{g,a}(t)$ | Annual background mortality rate by gender *g* and age *a.* |

***Fertility***

The number of births by HIV status of the mother are calculated as:

*HIV-negative women and women on ART*

$$b\_s\left( t \right)=\sum_{h=1}^{7} \sum_{s=1}^{7} \sum_{x=1}^{3} \sum_{p=1}^{4} \sum_{a=4}^{10} \sum_{r=1}^{3} \left[ \gamma_{a}^{1}\left( t \right)\cdot X_{g,a,r}^{1,1,h,s,x,p}\left( t \right)+\gamma_{a}^{8}\left( t \right)\cdot X_{g,a,r}^{8,6,h,s,x,p}\left( t \right) \right]$$

*Women living with HIV*

$$b\_i\left( t \right)=\sum_{d=3}^{7} \sum_{v=1}^{5} \sum_{h=1}^{7} \sum_{s=1}^{7} \sum_{x=1}^{3} \sum_{p=1}^{4} \sum_{a=4}^{10} \sum_{r=1}^{3} \left[ \gamma_{a}^{d}\left( t \right)\cdot X_{g,a,r}^{d,v,h,s,x,p}(t) \right]$$

We then compute the number of births by gender and HIV status of the infant as:

*HIV-negative, uncircumcised births*

*For h = s = x = p = a = r = 1,*

$$b_{g,a,r}^{1,1,h,s,x,p}\left( t \right)=0.5 \left( b\_s\left( t \right)+\left( 1-\eta\left( t \right) \right)b\_i\left( t \right) \right)$$

else,

$$b_{g,a,r}^{1,1,h,s,x,p}\left( t \right)=0$$

*HIV-positive births*

*For h = s = x = p = a = r = 1,*

$$b_{g,a,r}^{3,1,h,s,x,p}\left( t \right)=0.5 \eta\left( t \right)\cdot b\_i(t)$$

else,

$$b_{g,a,r}^{3,1,h,s,x,p}\left( t \right)=0$$

***Aging***

To age the population, one-fifth of each compartment moves to the next age group while maintaining the same gender, disease state, and sexual risk distribution $\phi_{g,a,r}$:

$$\frac{dX_{g,1,r}^{d,v,h,s,x,p}(t)}{dt}=-\frac{1}{5}\sum_{r=1}^{3} X_{g,1,r}^{d,v,h,s,x,p}\left( t \right) \cdot\phi_{g,a,r} (for a=1)$$

$$\frac{dX_{g,a,r}^{d,v,h,s,x,p}(t)}{dt}=-\frac{1}{5}\sum_{r=1}^{3} X_{g,a,r}^{d,v,h,s,x,p}\left( t \right) \cdot\phi_{g,a,r}+\frac{1}{5}\sum_{r=1}^{3} X_{g,a-1,r}^{d,v,h,s,x,p}\left( t \right) \cdot\phi_{g,a-1,r} (for a\neq1)$$

Upon aging to the next five-year group, individuals are re-distributed into the closest unfilled risk group to match observed data on the age distribution of low, moderate, and high-risk individuals.

*Mortality*

We compute the number of deaths due to background mortality as:

$$\frac{dX_{g,a,r}^{d,v,h,s,x,p}(t)}{dt}=-{\mu\_bkrd}_{g,a}\left( t \right)\cdot X_{g,a,r}^{d,v,h,s,x,p}\left( t \right)$$

*9.2 Sexual Behaviour*

***Mixing matrix***

| **Equation variables** | |
| --- | --- |
| $c_{g,a,r}$ | Number of partners a person has per year of gender *g,* age *a,* and sexual-risk group *r* (ie. the partner exchange rate, or contact rate). |
| $\epsilon_{a}$ | Mixing parameter by age *a.*  We assume a mixing pattern that is partially random and partially off-diagonal (0 < $\epsilon_{a}$ < 1), where ($\epsilon_{a}=0)$ indicates completely off-diagonal mixing, and ($\epsilon_{a}=1)$ indicates completely random mixing. |
| $\epsilon_{r}$ | Mixing parameter by sexual-risk group *r.*  We assume a mixing pattern that is partially random and partially on-diagonal (0 < $\epsilon_{r}$ < 1), where ($\epsilon_{r}=0)$ indicates completely on-diagonal mixing, and ($\epsilon_{r}=1)$ indicates completely random mixing. |
| $\delta_{g,a,a^{'}}$ | Mixing pattern by age.  In completely non-random mixing by age, women are most likely to form partnerships with men of the next oldest age group*.* We represent this pattern using on off-diagonal matrix.  For men (*g* = 1) of age *a* mixing with women of age *a’*:  = 0.3 if ($a=a^{'}$)  = 0.7 if ($a=a^{'}+1$)  except for the following (correct for no sexual activity before age group 3):  = 0.0 if ($a=a^{'}=1$)  = 0.0 if ($a=$ 2) and (*a’* = 1)  = 0.0 if ($a=$2) and (*a’* = 2)  = 0.0 if ($a=$ 3) and (*a’* = 2)  For women (*g* = 2) of age *a* mixing with men of age *a’*:  = 0.3 if ($a=a^{'}$)  = 0.7 if ($a=a^{'}-1$)  except for the following (correct for no sexual activity before age group 3):  = 0.0 if ($a=a^{'}=$1)  = 0.0 if ($a=$1) and (*a’* = 2)  = 0.0 if ($a=a^{'}=$2)  = 0.0 if ($a=$2) and (*a’* = 3) |
| $\delta_{r,r^{'}}$ | Mixing pattern by risk.  Completely non-random mixing by risk confines sexual encounters to individuals within the same risk group. We represent this pattern using an identity matrix.  = 1.0 if ($r=r^{'}$)  = 0.0 if ($r\neq r^{'}$) |

For a person of gender *g,* age *a,* and sexual-risk group *r,* we use the mixing matrix $\rho_{g,a,a^{'},r,r^{'}}(t)$ to describe the proportion of sexual partners that come from age group *a’* and sexual-risk group *r’*. We assume that mixing is partially random and partially designated by a mixing pattern $\delta_{g,a,a^{'}}$ or $\delta_{r,r^{'}}$. The overall mixing matrix is therefore a weighted average of random mixing proportional to the number of available partnerships of each group, and mixing among groups with similar characteristics. Although an off-diagonal mixing pattern results in the first and last ages groups (ages 10-14 and 75-79) having fewer than 100% of their partnerships, these age groups have relatively few partnerships and contribute marginally to overall infection transmission.

$$\rho_{g,a,a^{'},r,r^{'}}\left( t \right)=\left( \epsilon_{a}\cdot\frac{\sum_{r^{'}=1}^{3} \left( c_{g^{'},a^{'},r^{'}}\cdot\sum_{d^{'}=1}^{8} \sum_{v^{'}=1}^{6} \sum_{h^{'}=1}^{7} \sum_{s^{'}=1}^{7} \sum_{x^{'}=1}^{4} \sum_{p^{'}=1}^{4} X_{g',a',r'}^{d',v',h',s',x',p'}\left( t \right) \right)}{\sum_{a^{'}=1}^{16} \sum_{r^{'}=1}^{3} \left( c_{g^{'},a^{'},r^{'}}\cdot\sum_{d^{'}=1}^{8} \sum_{v^{'}=1}^{6} \sum_{h^{'}=1}^{7} \sum_{s^{'}=1}^{7} \sum_{x^{'}=1}^{4} \sum_{p^{'}=1}^{4} X_{g',a',r'}^{d',v',h',s',x',p'}\left( t \right) \right)}+\left( 1-\epsilon_{a} \right)\delta_{g,a,a^{'}} \right)\cdot\left( \epsilon_{r}\cdot\frac{\left( c_{g^{'},a^{'},r^{'}}\cdot\sum_{d^{'}=1}^{8} \sum_{v^{'}=1}^{6} \sum_{h^{'}=1}^{7} \sum_{s^{'}=1}^{7} \sum_{x^{'}=1}^{4} \sum_{p^{'}=1}^{4} X_{g',a',r'}^{d',v',h',s',x',p'}\left( t \right) \right)}{\sum_{r^{'}=1}^{3} \left( c_{g^{'},a^{'},r^{'}}\cdot\sum_{d^{'}=1}^{8} \sum_{v^{'}=1}^{6} \sum_{h^{'}=1}^{7} \sum_{s^{'}=1}^{7} \sum_{x^{'}=1}^{4} \sum_{p^{'}=1}^{4} X_{g',a',r'}^{d',v',h',s',x',p'}\left( t \right) \right)}+\left( 1-\epsilon_{r} \right)\delta_{g,r,r^{'}} \right)$$

***Rate of partner change***

| **Equation variables** | |
| --- | --- |
| $c_{g,a,r}$ | Number of partners a person has per year of gender *g,* age *a,* and sexual-risk group *r* (ie. the partner exchange rate, or contact rate).  We assume zero partnerships for individuals below the age of sexual debut (age 10). |
| $\theta$ | Gender influence on contact rate adjustment.  We assume an adjusted contact rate equally driven by rates reported by men and women ($\theta$= 0.5), where ($\theta$ = 0) when completely female-driven, and ($\theta$ = 1) when completely male-driven. |
| $\rho_{g,a,a^{'},r,r^{'}}\left( t \right)$ | Mixing matrix for a person of gender *g,* age *a,* and sexual-risk group *r* that describes the proportion of sexual partners that come from age group *a’* and sexual-risk group *r’.*  We assume a solely heterosexual population and therefore that all contacts are with the opposite gender. |

Bias in observed data leads to contact rates $c_{g,a,r}$ that, when assuming solely heterosexual contact, are inconsistent between men and women. We account for this variability by using an adjusted contact rate $c_{g,a,a^{'},r,r^{'}}^{*}(t)$ that ensures that the number of partnerships of men of age *a* and risk group *r* with women of age *a’* and risk group *r’* equals the number of partnerships of women of age *a* and risk group *r* with men of age *a’* and risk group *r’*.

We first calculate the discrepancy between reported contacts among men and women as:

$$B_{a,a^{'},r,r^{'}}\left( t \right)=\frac{c_{1,a,r}\cdot\rho_{1,a,a^{'},r,r^{'}}\left( t \right)\cdot\sum_{d^{'}=1}^{8} \sum_{v^{'}=1}^{6} \sum_{h^{'}=1}^{7} \sum_{s^{'}=1}^{7} \sum_{x^{'}=1}^{4} \sum_{p^{'}=1}^{4} X_{1,a',r'}^{d',v',h',s',x',p'}\left( t \right)}{c_{2,a,r}\cdot\rho_{2,a,a^{'},r,r^{'}}\left( t \right)\cdot\sum_{d=1}^{8} \sum_{v=1}^{6} \sum_{h=1}^{7} \sum_{s=1}^{7} \sum_{x=1}^{4} \sum_{p=1}^{4} X_{2,a,r}^{d,v,h,s,x,p}\left( t \right)}$$

We then compute the adjusted contact rate for women as:

$$c_{2,a,a^{'},r,r^{'}}^{*}\left( t \right)=c_{2,a,r}\cdot\rho_{2,a,a^{'},r,r^{'}}\left( t \right)\cdot{B_{a,a^{'},r,r^{'}}\left( t \right)}^{\theta}\cdot\left( \frac{\sum_{d^{'}=1}^{8} \sum_{v^{'}=1}^{6} \sum_{h^{'}=1}^{7} \sum_{s^{'}=1}^{7} \sum_{x^{'}=1}^{4} \sum_{p^{'}=1}^{4} X_{1,a',r'}^{d',v',h',s',x',p'}\left( t \right)}{\sum_{d=1}^{8} \sum_{v=1}^{6} \sum_{h=1}^{7} \sum_{s=1}^{7} \sum_{x=1}^{4} \sum_{p=1}^{4} X_{2,a,r}^{d,v,h,s,x,p}\left( t \right)} \right)^{-\left( 1-\theta\right)}$$

and for men, an adjusted contact rate of:

$$c_{1,a,a^{'},r,r^{'}}^{*}(t)=c_{1,a,r}\cdot\rho_{1,a,a^{'},r,r^{'}}\left( t \right)\cdot{B_{a,a^{'},r,r^{'}}\left( t \right)}^{-\left( 1-\theta\right)}\cdot\left( \frac{\sum_{d^{'}=1}^{8} \sum_{v^{'}=1}^{6} \sum_{h^{'}=1}^{7} \sum_{s^{'}=1}^{7} \sum_{x^{'}=1}^{4} \sum_{p^{'}=1}^{4} X_{1,a',r'}^{d',v',h',s',x',p'}\left( t \right)}{\sum_{d=1}^{8} \sum_{v=1}^{6} \sum_{h=1}^{7} \sum_{s=1}^{7} \sum_{x=1}^{4} \sum_{p=1}^{4} X_{2,a,r}^{d,v,h,s,x,p}\left( t \right)} \right)^{\theta}$$

*9.3 Transmission Probabilities*

***Per-partnership probability of transmission***

| **Equation variables** | |
| --- | --- |
| $A_{g,a,r}$ | Number of acts a person has per partnership of gender *g,* age *a,* and sexual-risk group *r*.  We assume zero acts for individuals below the age of sexual debut (age 10). |
| ${\chi\_HIV}_{g}^{v^{'},x^{'}}$ | Per-act probability of HIV transmission to a person of gender *g* based on the viral load *v’* of the partner living with HIV*.*  We assume the probability of female-to-male HIV transmission is equal to the probability of male-to-female transmission across all viral load stages (${\chi\_HIV}_{1}^{v^{'}}= {\chi\_HIV}_{2}^{v^{'}}$). We reduce HIV per-act transmission as a proxy for decreased sexual activity during late-stage HIV (*v’* = 5), regional or distant cervical cancer (*x’* = 2 or *x’* = 3), or hysterectomy (*x’* = 4). |
| ${\chi\_HPV}_{g}^{v^{'},x^{'}}$ | Per-act probability of HPV transmission to a person of gender *g* by an HPV-positive partner.  We assume the per-act probability of HPV transmission is the same for vaccine-type and non-vaccine-type HPV and across all stages of pre-cancer or cervical cancer. We reduce HPV per-act transmission as a proxy for decreased sexual activity during late-stage HIV (*v’* = 5) or regional or distant cervical cancer (*x’* = 2 or *x’* = 3). We assume no HPV transmission after hysterectomy (*x’* = 4). |

The per-partnership probability of HIV transmission ${\beta\_HIV}_{g,a,r}^{v^{'},x^{'}}$ is the cumulative risk of acquiring HIV from all sexual acts with a partner. This quantity depends on the per-act probability of HIV transmission and the number of acts per partnership.

We calculate the per-partnership probability of HIV transmission to a male partner:

$${\beta\_HIV}_{1,a,r}^{v^{'},x^{'}}=1-{(1-{\chi\_HIV}_{1}^{v^{'},x^{'}})}^{A_{1,a,r}}$$

Similarly, the per-partnership probability of HIV transmission to a female partner:

$${\beta\_HIV}_{2,a,r}^{v^{'},x^{'}}=1-{(1-{\chi\_HIV}_{2}^{v^{'},x^{'}})}^{A_{2,a,r}}$$

Likewise, the per-partnership probability of HPV transmission ${\beta\_HPV}_{g,a,r}^{v^{'},x^{'}}$ depends on the per-act probability of HPV transmission and the number of acts per partnership.

We calculate the per-partnership probability of HPV transmission to a male partner:

$${\beta\_HPV}_{1,a,r}^{v^{'},x^{'}}=1-{(1-{\chi\_HPV}_{1}^{v^{'},x^{'}})}^{A_{1,a,r}}$$

Similarly, the per-partnership probability of HPV transmission to a female partner:

$${\beta\_HPV}_{2,a,r}^{v^{'},x^{'}}=1-{(1-{\chi\_HPV}_{2}^{v^{'},x^{'}})}^{A_{2,a,r}}$$

***Force of infection***

| **Equation variables** | |
| --- | --- |
| $c_{g,a,a^{'},r,r^{'}}^{*}(t)$ | Adjusted yearly contact rate for persons of gender *g,* age *a,* and risk group *r,* with persons of the opposite gender, age *a’,* and risk group *r’.* |
| ${\beta\_HIV}_{g,a,r}^{v^{'},x^{'}}$ | Annual per-partnership probability of HIV transmission from a person with HIV with viral load *v’* and cervical cancer stage *x’* to a HIV-susceptible partner with gender *g,* age *a,* and risk group *r.* |
| ${\beta\_HPV}_{g,a,r}^{v^{'},x^{'}}$ | Annual per-partnership probability of HPV transmission from a HPV-infected person with viral load *v’* and cervical cancer stage *x’* to a HPV-susceptible partner with gender *g,* age *a,* and risk group *r.* |

The force of infection represents the cumulative risk of acquiring HIV or HPV from all possible partners, and depends on the adjusted contact rate, the per-partnership probability of transmission, and the proportion of sexually active persons who are HIV- or HPV-infected.

The force of infection ${\lambda\_HIV}_{g,a,r}(t)$ determines HIV disease transmission:

$${\lambda\_HIV}_{g,a,r}(t)=\sum_{a^{'}=1}^{16} \sum_{r^{'}=1}^{3} \left( c_{g,a,a^{'},r,r^{'}}^{*}(t)\cdot\frac{-\sum_{v^{'}=1}^{6} \sum_{x^{'}=1}^{4} ln(1-{\beta\_HIV}_{g,a,r}^{v^{'},x^{'}})\cdot\sum_{d^{'}=3}^{8} \sum_{h^{'}=1}^{7} \sum_{s^{'}=1}^{7} \sum_{p^{'}=1}^{4} X_{g',a',r'}^{d',v',h',s',x',p'}\left( t \right)}{\sum_{d^{'}=1}^{8} \sum_{v^{'}=1}^{6} \sum_{h^{'}=1}^{7} \sum_{s^{'}=1}^{7} \sum_{x^{'}=1}^{4} \sum_{p^{'}=1}^{4} X_{g',a',r'}^{d',v',h',s',x',p'}\left( t \right)} \right)$$

Similarly, the force of infection ${\lambda\_vHPV}_{g,a,r}(t)$ determines vaccine-type HPV transmission:

$${\lambda\_vHPV}_{g,a,r}(t)=\sum_{a^{'}=1}^{16} \sum_{r^{'}=1}^{3} \left( c_{g,a,a^{'},r,r^{'}}^{*}(t)\cdot\frac{-\sum_{v^{'}=1}^{6} \sum_{x^{'}=1}^{4} ln(1-{\beta\_HPV}_{g,a,r}^{v^{'},x^{'}})\cdot\sum_{d^{'}=1}^{8} \sum_{h^{'}=2}^{6} \sum_{s^{'}=1}^{7} \sum_{p^{'}=1}^{4} X_{g',a',r'}^{d',v',h',s',x',p'}\left( t \right)}{\sum_{d^{'}=1}^{8} \sum_{v^{'}=1}^{6} \sum_{h^{'}=1}^{7} \sum_{s^{'}=1}^{7} \sum_{x^{'}=1}^{4} \sum_{p^{'}=1}^{4} X_{g',a',r'}^{d',v',h',s',x',p'}\left( t \right)} \right)$$

and $\lambda{\_nvHPV}_{g,a,r}(t)$ defines non-vaccine-type HPV transmission:

$$\lambda{\_nvHPV}_{g,a,r}\left( t \right)=\sum_{a^{'}=1}^{16} \sum_{r^{'}=1}^{3} \left( c_{g,a,a^{'},r,r^{'}}^{*}(t)\cdot\frac{-\sum_{v^{'}=1}^{6} \sum_{x^{'}=1}^{4} ln(1-{\beta\_HPV}_{g,a,r}^{v^{'},x^{'}})\cdot\sum_{d^{'}=1}^{8} \sum_{h^{'}=1}^{7} \sum_{s^{'}=2}^{6} \sum_{p^{'}=1}^{4} X_{g',a',r'}^{d',v',h',s',x',p'}\left( t \right)}{\sum_{d^{'}=1}^{8} \sum_{v^{'}=1}^{6} \sum_{h^{'}=1}^{7} \sum_{s^{'}=1}^{7} \sum_{x^{'}=1}^{4} \sum_{p^{'}=1}^{4} X_{g',a',r'}^{d',v',h',s',x',p'}\left( t \right)} \right)$$

*9.4 Natural History and Interventions*

***HIV***

| **Equation variables** | |
| --- | --- |
| ${\mu\_HIV}_{g,a}^{d}$ | Annual HIV-associated mortality rate by gender *g*, age *a*, and HIV disease stage *d* for (3 ≤ *d* ≤ 8)*.* |
| ${\lambda\_HIV}_{g,a,r}(t)$ | Force of HIV infection for HIV-negative persons by gender *g,* age *a,* and risk *r*. |
| ${\rho\_HIV}_{g}$ | Reduction in HIV acquisition due to circumcision by gender.  Only men receive circumcision (${\rho\_HIV}_{2}=1$). |
| ${\psi\_HIV}_{g}$ | Reduction in HIV acquisition due to population-level condom use by gender. |
| $\omega^{d}$ | The rate of progressing from HIV stage *d* to stage *d + 1,* for (3 ≤ *d* ≤ 7)*.* |
| $l^{d}$ | The rate of progressing from viral load stage *v* to *v + 1,* for (1 ≤ *v* ≤ 5). |
| $P_{g,a}(t)$ | The proportion of HIV-negative persons of gender *g* and age *a* that are circumcised.  Only men receive circumcision ($P_{2,a}(t)$ = 0). |
| $A_{g,a}^{d}(t)$ | The proportion of persons living with HIV of disease stage *d,* gender *g,* and age *a* that initiate ART. |
| $\sigma_{g,a,r}^{d,v}(t)$ | The proportion of persons who discontinue ART based on the recent distribution of persons initiating ART by gender *g*, age *a,* risk *r,* disease *d,* and viral *v*. |

We calculate changes in HIV status and HIV stage defined by CD4 count, viral load, and treatment status. The HIV-negative population can acquire HIV after sexual debut with a force of infection reduced by circumcision among men and condom use by either gender. We only track circumcision among HIV-negative men. Individuals with HIV infection experience HIV-associated mortality, CD4 and viral load stage progression, and ART initiation and discontinuation. CD4 and viral load stage are not tracked among persons on treatment.

*HIV-negative, uncircumcised*

$$\frac{dX_{g,a,r}^{1,1,h,s,x,p}\left( t \right)}{dt}=-\left( {\psi\_HIV}_{g}{\cdot\lambda\_HIV}_{g,a,r}\left( t \right) +P_{g,a}\left( t \right) \right)X_{g,a,r}^{1,1,h,s,x,p}\left( t \right)$$

*HIV-negative, circumcised*

$$\frac{dX_{g,a,r}^{2,1,h,s,x,p}\left( t \right)}{dt}=P_{g,a}\left( t \right) \cdot X_{g,a,r}^{1,1,h,s,x,p}\left( t \right)$$

$$- \left( {\psi\_HIV}_{g}\cdot{\rho\_HIV}_{g}\cdot{\lambda\_HIV}_{g,a,r}(t) \right) X_{g,a,r}^{2,1,h,s,x,p}\left( t \right)$$

*HIV-positive, acute infection*

$$\frac{dX_{g,a,r}^{3,1,h,s,x,p}\left( t \right)}{dt}={\psi\_HIV}_{g}{\cdot\lambda\_HIV}_{g,a,r}(t)\cdot X_{g,a,r}^{1,1,h,s,x,p}\left( t \right)$$

$$+ {\psi\_HIV}_{g}\cdot{\rho\_HIV}_{g}\cdot{\lambda\_HIV}_{g,a,r}\left( t \right)\cdot X_{g,a,r}^{2,1,h,s,x,p}\left( t \right)+\sigma_{g,a,r}^{3,1}(t){\cdot X}_{g,a,r}^{8,6,h,s,x,p}\left( t \right) -\left( {\mu\_HIV}_{g,a}^{3}+\omega^{3}+A_{g,a}^{3}(t) \right)X_{g,a,r}^{3,1,h,s,x,p}\left( t \right)$$

*HIV-positive, CD4 > 500 cells/µL*

$$\frac{dX_{g,a,r}^{4,v,h,s,x,p}\left( t \right)}{dt}= \omega^{3}{\cdot X}_{g,a,r}^{3,v,h,s,x,p}\left( t \right)+ l^{v-1}{\cdot X}_{g,a,r}^{4,v-1,h,s,x,p}\left( t \right) +\sigma_{g,a,r}^{4,v}(t)\cdot X_{g,a,r}^{8,6,h,s,x,p}\left( t \right)-\left( {\mu\_HIV}_{g,a}^{4}+\omega^{4}+l^{v}+A_{g,a}^{4}(t) \right)X_{g,a,r}^{4,v,h,s,x,p}\left( t \right)$$

*HIV-positive, CD4 350-500 cells/µL*

$$\frac{dX_{g,a,r}^{5,v,h,s,x,p}\left( t \right)}{dt}= \omega^{4}\cdot X_{g,a,r}^{4,v,h,s,x,p}\left( t \right)+ l^{v-1}\cdot X_{g,a,r}^{5,v-1,h,s,x,p}\left( t \right) +\sigma_{g,a,r}^{5,v}(t){\cdot X}_{g,a,r}^{8,6,h,s,x,p}\left( t \right)-\left( {\mu\_HIV}_{g,a}^{5}+\omega^{5}+l^{v}+A_{g,a}^{5}(t) \right)X_{g,a,r}^{5,v,h,s,x,p}\left( t \right)$$

*HIV-positive, CD4 200-350 cells/µL*

$$\frac{dX_{g,a,r}^{6,v,h,s,x,p}\left( t \right)}{dt}= \omega^{5}{\cdot X}_{g,a,r}^{5,v,h,s,x,p}\left( t \right)+ l^{v-1}\cdot X_{g,a,r}^{6,v-1,h,s,x,p}\left( t \right) +\sigma_{g,a,r}^{6,v}(t){\cdot X}_{g,a,r}^{8,6,h,s,x,p}\left( t \right)-\left( {\mu\_HIV}_{g,a}^{6}+\omega^{6}+l^{v}+A_{g,a}^{6}(t) \right)X_{g,a,r}^{6,v,h,s,x,p}\left( t \right)$$

*HIV-positive, CD4 ≤ 200 cells/µL*

$$\frac{dX_{g,a,r}^{7,v,h,s,x,p}\left( t \right)}{dt}= \omega^{6}{\cdot X}_{g,a,r}^{6,v,h,s,x,p}\left( t \right)+ l^{v-1}{\cdot X}_{g,a,r}^{7,v-1,h,s,x,p}\left( t \right) +\sigma_{g,a,r}^{7,v}(t)\cdot X_{g,a,r}^{8,6,h,s,x,p}\left( t \right)-\left( {\mu\_HIV}_{g,a}^{7}+\omega^{7}+l^{v}+A_{g,a}^{7}(t) \right)X_{g,a,r}^{7,v,h,s,x,p}\left( t \right)$$

*HIV-positive, on ART*

$$\frac{dX_{g,a,r}^{8,6,h,s,x,p}\left( t \right)}{dt}= \sum_{d=3}^{7} \sum_{v=1}^{5} \left( A_{g,a}^{d}\left( t \right)\cdot X_{g,a,r}^{d,v,h,s,x,p}\left( t \right)-\sigma_{g,a,r}^{d,v}{\cdot X}_{g,a,r}^{8,6,h,s,x,p}\left( t \right) \right)$$

***HPV***

| **Equation variables** | |
| --- | --- |
| ${\mu\_HPV}_{g}^{d,h,s,x}$ | Annual cervical cancer-associated mortality rate by gender *g*, HIV disease stage *d*, vaccine-type HPV stage *h,* non-vaccine-type HPV stage *s,* and cervical cancer stage *x* for (1 ≤ *x* ≤ 3).  Only women have cervical cancer-associated mortality (${\mu\_HPV}_{1}^{d,h,s,x}$ = 0) and only when (*h* = 6 or *s* = 6). |
| ${\lambda\_vHPV}_{g,a,r}(t)$ | Force of vaccine-type HPV infection for susceptible persons of gender *g,* age *a,* and risk *r*. |
| ${\lambda\_nvHPV}_{g,a,r}(t)$ | Force of non-vaccine-type HPV infection for susceptible persons of gender *g,* age *a,* and risk *r*. |
| $ж_{d}$ | HPV acquisition risk multiplier for individuals with HIV with CD4 count (4 ≤ *d* ≤ 7)*.* |
| ${\psi\_HPV}_{g}$ | HPV acquisition reduction multiplier due to population-level condom use by gender. |
| $\xi_{g,a}$ | HPV acquisition reduction multiplier by gender and age for individuals with type-specific natural immunity.  Only women temporarily develop partial natural immunity ($\xi_{1,a}=0$). Older women develop stronger natural immunity than young girls. |
| $\phi_{a}$ | Vaccine-type HPV acquisition reduction multiplier by age for vaccinated individuals.  We assume life-long protection with vaccination ($\phi_{a}$ is equivalent for all vaccinated ages). |
| ${k\_v}_{g,a}^{h,h^{'}}$ | Transition rate of progressing or regressing from vaccine-type HPV precancer or disease stage *h* to stage *h’*.  Only women develop precancerous lesions and cervical cancer (${k\_v}_{1,a}^{h,h^{'}}$= 0 except for HPV clearance when *h* = 2 and *h’* = 1). |
| ${k\_nv}_{g,a}^{s,s^{'}}$ | Transition rate of progressing or regressing from non-vaccine-type HPV precancer or disease stage *s* to stage *s’*.  Only women develop precancerous lesions and cervical cancer (${k\_v}_{1,a}^{s,s^{'}}$= 0 except for HPV clearance when *s* = 2 and s*’* = 1). |
| $r_{g}$ | Rate of waning type-specific natural immunity.  Only women temporarily develop partial natural immunity ($r_{1}=0$). |
| $\varphi_{g}^{h,s,x,x^{'}}$ | Progression rate of cervical cancer from stage *x* to stage *x’*.  Only women develop cervical cancer ($\varphi_{1}^{h,s,x,x^{'}}=0$) and ($\varphi_{2}^{h,s,x,x^{'}}>0$ only when *h* or *s* = 6) |
| ${\zeta\_v}^{d,h,h^{'}}$ | Transition rate multiplier for individuals with HIV progressing or regressing from vaccine-type precancer or disease stage *h* to stage *h’* with CD4 count *d.*  Transition rate multipliers for individuals with HIV are the same for vaccine-type and non-vaccine-type HPV (${\zeta\_v}^{d,h,h^{'}}= {\zeta\_nv}^{d,s,s^{'}}$ when *h* = *s* and *h’* = *s’*)*.* |
| ${\zeta\_nv}^{d,s,s^{'}}$ | Transition rate multiplier for individuals with HIV progressing or regressing from non-vaccine-type precancer or disease stage *s* to stage *s’* with gender *g* and CD4 count *d.*  Transition rate multipliers for individuals with HIV are the same for vaccine-type and non-vaccine-type HPV (${\zeta\_v}^{d,h,h^{'}}= {\zeta\_nv}^{d,s,s^{'}}$ when *h* = *s* and *h’* = *s’*)*.* |
| $\mathcal{l}_{g}$ | Additional multiplier for clearance of vaccine or non-vaccine-type HPV infection.  Only applied to men ($\mathcal{l}_{2}=1$). |
| $V_{g,a}^{d}$ | The proportion of persons with HIV disease status *d,* gender *g,* and age *a* vaccinated*.* |

We calculate changes in HPV status and precancer or disease stage for women without hysterectomy (1 ≤ *x* ≤ 3). We track vaccine-type and non-vaccine type HPV independently, but only enumerate cancer incidence for the first infection to progress to local cervical cancer. CIN1,2,3 can regress and HPV infection can clear naturally. Women who clear HPV temporarily develop partial natural immunity against reinfection with the same HPV type group while men who clear HPV do not develop natural immunity. Persons susceptible to type-specific HPV infection or with temporary natural immunity can acquire HPV after sexual debut. Individuals with HIV experience higher rates of HPV acquisition and disease progression, and lower rates of HPV clearance, immunity waning, and disease regression. Cervical cancer mortality varies by cancer stage and HIV status, and affects individuals regardless of type-specific etiology. We assume that the nonavalent HPV vaccine provides lifelong protection against vaccine-type HPV and no protection against non-vaccine-type HPV. We assume the vaccine is ineffective for persons with current vaccine-type HPV infection, and the equations therefore only reflect vaccination of persons susceptible or immune to vaccine-type HPV. Vaccination does not depend on non-vaccine-type HPV infection status. Although not shown in the equations below, persons are screened according to their age *a* and lose their screened status upon aging out of the screened age group.

***Vaccine-type HPV and precancer equations***

*Men, susceptible*

$$\frac{dX_{1,a,r}^{d,v,1,s,1,1}\left( t \right)}{dt}= {\mathcal{l}_{1}\cdot\zeta\_v}^{d,2,1}\cdot{k\_v}_{1,a}^{2,1}\cdot X_{1,a,r}^{d,v,2,s,1,1}\left( t \right) -\left( ж_{d}\cdot{\psi\_HPV}_{1}{\cdot\lambda\_vHPV}_{1,a,r}(t)+V_{1,a}^{d} \right)X_{1,a,r}^{d,v,1,s,1,1}\left( t \right)$$

*Men, HPV-infected*

$$\frac{dX_{1,a,r}^{d,v,2,s,1,1}\left( t \right)}{dt}={ж_{d}\cdot{\psi\_HPV}_{1}{\cdot\lambda\_vHPV}_{1,a,r}(t)\cdot X}_{1,a,r}^{d,v,1,s,1,1}\left( t \right)$$

$$- {\mathcal{l}_{1}\cdot\zeta\_v}^{d,2,1}\cdot{k\_v}_{1,a}^{2,1}\cdot X_{1,a,r}^{d,v,2,s,1,1}\left( t \right)$$

*Men, susceptible, vaccinated*

$$\frac{dX_{1,a,r}^{d,v,1,s,1,2}\left( t \right)}{dt}= V_{1,a}^{d}\cdot X_{1,a,r}^{d,v,1,s,1,1}\left( t \right)+ {\mathcal{l}_{1}\cdot\zeta\_v}^{d,2,1}\cdot{k\_v}_{1,a}^{2,1}\cdot X_{1,a,r}^{d,v,2,s,1,2}\left( t \right) -{\phi_{a}\cdotж_{d}\cdot{\psi\_HPV}_{1}{\cdot\lambda\_vHPV}_{1,a,r}(t)\cdot X}_{1,a,r}^{d,v,1,s,1,2}\left( t \right)$$

*Men, HPV-infected, vaccinated*

$$\frac{dX_{1,a,r}^{d,v,2,s,1,2}\left( t \right)}{dt}={{\phi_{a}\cdotж}_{d}\cdot{\psi\_HPV}_{1}{\cdot\lambda\_vHPV}_{1,a,r}(t)\cdot X}_{1,a,r}^{d,v,1,s,1,2}\left( t \right)$$

$$- {\mathcal{l}_{1}\cdot\zeta\_v}^{d,2,1}\cdot{k\_v}_{1,a}^{2,1}\cdot X_{1,a,r}^{d,v,2,s,1,2}\left( t \right)$$

*Women, susceptible*

$$\frac{dX_{2,a,r}^{d,v,1,s,x,[1,3]}\left( t \right)}{dt}= {\zeta\_v}^{d,7,1}\cdot r_{2}\cdot X_{2,a,r}^{d,v,7,s,x,[1,3]}\left( t \right) -\left( ж_{d}\cdot{\psi\_HPV}_{2}{\cdot\lambda\_vHPV}_{2,a,r}(t)+V_{2,a}^{d}+{\mu\_HPV}_{2}^{d,1,s,x} \right)X_{2,a,r}^{d,v,1,s,x,[1,3]}\left( t \right)$$

*Women, immune*

$$\frac{dX_{2,a,r}^{d,v,7,s,x,[1,3]}\left( t \right)}{dt}= V_{2,a}^{d}\cdot X_{2,a,r}^{d,v,7,s,x,[1,3]}\left( t \right)+{\zeta\_v}^{d,2,7}\cdot{k\_v}_{2,a}^{2,7}\cdot X_{2,a,r}^{d,v,2,s,x,[1,3]}\left( t \right) -\left( {\zeta\_v}^{d,7,1}\cdot{k\_v}_{2,a}^{7,1}+\xi_{2,a}{\cdotж}_{d}\cdot{\psi\_HPV}_{2}{\cdot\lambda\_vHPV}_{2,a,r}(t)+V_{2,a}^{d}+{\mu\_HPV}_{2}^{d,7,s,x} \right)X_{2,a,r}^{d,v,7,s,x,[1,3]}\left( t \right)$$

*Women, HPV-infected*

$$\frac{dX_{2,a,r}^{d,v,2,s,x,[1,3]}\left( t \right)}{dt}= {\zeta\_v}^{d,3,2}\cdot{k\_v}_{2,a}^{3,2}\cdot X_{2,a,r}^{d,v,3,s,x,\left[ 1,3 \right]}\left( t \right) +ж_{d}\cdot{\psi\_HPV}_{2}{\cdot\lambda\_vHPV}_{2,a,r}\left( t \right)\cdot X_{2,a,r}^{d,v,1,s,x,\left[ 1,3 \right]}\left( t \right)+\xi_{2,a}{\cdotж}_{d}\cdot{\psi\_HPV}_{2}{\cdot\lambda\_vHPV}_{2,a,r}\left( t \right)\cdot X_{2,a,r}^{d,v,7,s,x,\left[ 1,3 \right]}\left( t \right)-\left( {\zeta\_v}^{d,2,7}\cdot{k\_v}_{2,a}^{2,7}+{\zeta\_v}^{d,2,3}\cdot{k\_v}_{2,a}^{2,3}+{\mu\_HPV}_{2}^{d,2,s,x} \right)X_{2,a,r}^{d,v,2,s,x,[1,3]}\left( t \right)$$

*Women, susceptible, vaccinated*

$$\frac{dX_{2,a,r}^{d,v,1,s,x,[2,4]}\left( t \right)}{dt}= {\zeta\_v}^{d,7,1}\cdot r_{2}\cdot X_{2,a,r}^{d,v,7,s,x,\left[ 2,4 \right]}\left( t \right)+V_{2,a}^{d}\cdot X_{2,a,r}^{d,v,1,s,x,[1,3]}\left( t \right)-\left( \phi_{a}\cdotж_{d}\cdot{\psi\_HPV}_{2}{\cdot\lambda\_vHPV}_{2,a,r}(t)+{\mu\_HPV}_{2}^{d,1,s,x} \right)X_{2,a,r}^{d,v,1,s,x,[2,4]}\left( t \right)$$

*Women, immune, vaccinated*

$$\frac{dX_{2,a,r}^{d,v,7,s,x,[2,4]}\left( t \right)}{dt}= {\zeta\_v}^{d,2,7}\cdot{k\_v}_{2,a}^{2,7}\cdot X_{2,a,r}^{d,v,2,s,x,\left[ 2,4 \right]}\left( t \right)+V_{2,a}^{d}\cdot X_{2,a,r}^{d,v,7,s,x,[1,3]}\left( t \right) -\left( {\zeta\_v}^{d,7,1}\cdot{k\_v}_{2,a}^{7,1}+{\phi_{a}\cdot\xi}_{2,a}{\cdotж}_{d}\cdot{\psi\_HPV}_{2}{\cdot\lambda\_vHPV}_{2,a,r}(t)+{\mu\_HPV}_{2}^{d,7,s,x} \right)X_{2,a,r}^{d,v,7,s,x,[2,4]}\left( t \right)$$

*Women, HPV-infected, vaccinated*

$$\frac{dX_{2,a,r}^{d,v,2,s,x,[2,4]}\left( t \right)}{dt}= {\zeta\_v}^{d,3,2}\cdot{k\_v}_{2,a}^{3,2}\cdot X_{2,a,r}^{d,v,3,s,x,\left[ 2,4 \right]}\left( t \right) +{\phi_{a}\cdotж}_{d}\cdot{\psi\_HPV}_{2}{\cdot\lambda\_vHPV}_{2,a,r}\left( t \right)\cdot X_{2,a,r}^{d,v,2,s,x,\left[ 2,4 \right]}\left( t \right)+{\phi_{a}\cdot\xi}_{2,a}{\cdotж}_{d}\cdot{\psi\_HPV}_{2}{\cdot\lambda\_vHPV}_{2,a,r}\left( t \right)\cdot X_{2,a,r}^{d,v,7,s,x,\left[ 2,4 \right]}\left( t \right)-\left( {\zeta\_v}^{d,2,7}\cdot{k\_v}_{2,a}^{2,7}+{\zeta\_v}^{d,2,3}\cdot{k\_v}_{2,a}^{2,3}+{\mu\_HPV}_{2}^{d,2,s,x} \right)X_{2,a,r}^{d,v,2,s,x,[2,4]}\left( t \right)$$

*Women, CIN1*

$$\frac{dX_{2,a,r}^{d,v,3,s,x,p}\left( t \right)}{dt}= {\zeta\_v}^{d,4,3}\cdot{k\_v}_{2,a}^{4,3}\cdot X_{2,a,r}^{d,v,4,s,x,p}\left( t \right) +{\zeta\_v}^{d,2,3}\cdot{k\_v}_{2,a}^{2,3}\cdot X_{2,a,r}^{d,v,2,s,x,p}\left( t \right)-\left( {\zeta\_v}^{d,3,4}\cdot{k\_v}_{2,a}^{3,4}+{\zeta\_v}^{d,3,2}\cdot{k\_v}_{2,a}^{3,2}+{\mu\_HPV}_{2}^{d,3,s,x} \right)X_{2,a,r}^{d,v,3,s,x,p}\left( t \right)$$

*Women, CIN2*

$$\frac{dX_{2,a,r}^{d,v,4,s,x,p}\left( t \right)}{dt}= {\zeta\_v}^{d,5,4}\cdot{k\_v}_{2,a}^{5,4}\cdot X_{2,a,r}^{d,v,5,s,x,p}\left( t \right) +{\zeta\_v}^{d,3,4}\cdot{k\_v}_{2,a}^{3,4}\cdot X_{2,a,r}^{d,v,3,s,x,p}\left( t \right)-\left( {\zeta\_v}^{d,4,5}\cdot{k\_v}_{2,a}^{4,5}+{\zeta\_v}^{d,4,3}\cdot{k\_v}_{2,a}^{4,3}+{\mu\_HPV}_{2}^{d,4,s,x} \right)X_{2,a,r}^{d,v,4,s,x,p}\left( t \right)$$

*Women, CIN3*

$$\frac{dX_{2,a,r}^{d,v,5,s,x,p}\left( t \right)}{dt}= {\zeta\_v}^{d,4,5}\cdot{k\_v}_{2,a}^{4,5}\cdot X_{2,a,r}^{d,v,4,s,x,p}\left( t \right)-\left( {\zeta\_v}^{d,5,6}\cdot{k\_v}_{2,a}^{5,6}+{\zeta\_v}^{d,5,4}\cdot{k\_v}_{2,a}^{5,4}+{\mu\_HPV}_{2}^{d,5,s,x} \right)X_{2,a,r}^{d,v,5,s,x,p}\left( t \right)$$

***Non-vaccine-type HPV and precancer equations***

The non-vaccine-type HPV and precancer equations follow the same pattern as the vaccine-type HPV equations with a few updates. All values of *s* equal the values of *h* in the vaccine-type equations, and *h* equals any value. The appropriate force of infection, transition rates, and transition rate multipliers for individuals living with HIV should be used. Vaccination does not depend on non-vaccine-type HPV infection status.

***Cervical cancer equations***

*Women, cervical cancer, local*

*(where h=6)*

$$\frac{dX_{2,a,r}^{d,v,6,s,x,p}\left( t \right)}{dt}= {\zeta\_v}^{d,5,6}\cdot{k\_v}_{2,a}^{5,6}\cdot X_{2,a,r}^{d,v,5,s,x,p}\left( t \right)$$

*(where s=6)*

$$\frac{dX_{2,a,r}^{d,v,h,6,x,p}\left( t \right)}{dt}= {\zeta\_v}^{d,5,6}\cdot{k\_v}_{2,a}^{5,6}\cdot X_{2,a,r}^{d,v,h,6,x,p}\left( t \right)$$

*(where h=6 or s=6)*

$$\frac{dX_{2,a,r}^{d,v,h,s,1,p}\left( t \right)}{dt}=-\left( \varphi_{2}^{h,s,1,2}+{\mu\_HPV}_{2}^{d,h,s,1} \right)X_{2,a,r}^{d,v,h,s,1,p}\left( t \right)$$

*Women, cervical cancer, regional (where h=6 or s=6)*

$$\frac{dX_{2,a,r}^{d,v,h,s,2,p}\left( t \right)}{dt}= \varphi_{2}^{h,s,1,2}\cdot X_{2,a,r}^{d,v,h,s,1,p}\left( t \right)-\left( \varphi_{2}^{h,s,2,3}+{\mu\_HPV}_{2}^{d,h,s,2} \right)X_{2,a,r}^{d,v,h,s,2,p}\left( t \right)$$

*Women, cervical cancer, distant (where h=6 or s=6)*

$$\frac{dX_{2,a,r}^{d,v,h,s,3,p}\left( t \right)}{dt}= \varphi_{2}^{h,s,2,3}\cdot X_{2,a,r}^{d,v,h,s,2,p}\left( t \right)-\left( {\mu\_HPV}_{2}^{d,h,s,3} \right)X_{2,a,r}^{d,v,h,s,3,p}\left( t \right)$$

**10) References**

1. Tan N, Sharma M, Winer R, Galloway D, Rees H, Barnabas RV. Model-estimated effectiveness of single dose 9-valent HPV vaccination for HIV-positive and HIV-negative females in South Africa. Vaccine. 2018;36(32 Pt A):4830-6.

2. Department of Economic and Social Affairs PD. World Population Prospects 2019. In: Nations U, editor. Rev. 1 ed. Online Edition2019.

3. Africa SS. Mid-year population estimates 2019. Statistical Release P0302. Pretoria, South Africa2019.

4. Moultrie TA, Hosegood V, McGrath N, Hill C, Herbst K, Newell ML. Refining the criteria for stalled fertility declines: an application to rural KwaZulu-Natal, South Africa, 1990-2005. Stud Fam Plann. 2008;39(1):39-48.

5. Moultrie TA, Timaeus IM. The South African fertility decline: Evidence from two censuses and a Demographic and Health Survey. Popul Stud (Camb). 2003;57(3):265-83.

6. Anderson RM, May RM, Ng TW, Rowley JT. Age-dependent choice of sexual partners and the transmission dynamics of HIV in Sub-Saharan Africa. Philos Trans R Soc Lond B Biol Sci. 1992;336(1277):135-55.

7. Ross A, Van der Paal L, Lubega R, Mayanja BN, Shafer LA, Whitworth J. HIV-1 disease progression and fertility: the incidence of recognized pregnancy and pregnancy outcome in Uganda. AIDS. 2004;18(5):799-804.

8. Bobat R, Coovadia H, Coutsoudis A, Moodley D. Determinants of mother-to-child transmission of human immunodeficiency virus type 1 infection in a cohort from Durban, South Africa. Pediatr Infect Dis J. 1996;15(7):604-10.

9. Horwood C, Vermaak K, Butler L, Haskins L, Phakathi S, Rollins N. Elimination of paediatric HIV in KwaZulu-Natal, South Africa: large-scale assessment of interventions for the prevention of mother-to-child transmission. Bull World Health Organ. 2012;90(3):168-75.

10. Rollins N, Little K, Mzolo S, Horwood C, Newell ML. Surveillance of mother-to-child transmission prevention programmes at immunization clinics: the case for universal screening. AIDS. 2007;21(10):1341-7.

11. Network GBoDC. Global Burden of Disease Study 2017 (GBD 2017) Results. In: (IHME) IfHMaE, editor. Seattle, United States2018.

12. Institute AHR. Africa Centre cohort data from KwaZulu-Natal, SA Surveillance data repository. Durban, South Africa.

13. Garnett GP, Gregson S. Monitoring the course of the HIV-1 epidemic: The influence of patterns of fertility on HIV-1 prevalence estimates. Mathematical Population Studies. 2000;8:251-77.

14. Ott MQ, Barnighausen T, Tanser F, Lurie MN, Newell ML. Age-gaps in sexual partnerships: seeing beyond 'sugar daddies'. AIDS. 2011;25(6):861-3.

15. de Oliveira T, Kharsany AB, Graf T, Cawood C, Khanyile D, Grobler A, et al. Transmission networks and risk of HIV infection in KwaZulu-Natal, South Africa: a community-wide phylogenetic study. The lancet HIV. 2017;4(1):e41-e50.

16. Quinn TC, Wawer MJ, Sewankambo N, Serwadda D, Li C, Wabwire-Mangen F, et al. Viral load and heterosexual transmission of human immunodeficiency virus type 1. Rakai Project Study Group. The New England journal of medicine. 2000;342(13):921-9.

17. Hubert JB, Burgard M, Dussaix E, Tamalet C, Deveau C, Le Chenadec J, et al. Natural history of serum HIV-1 RNA levels in 330 patients with a known date of infection. The SEROCO Study Group. AIDS. 2000;14(2):123-31.

18. Lingappa JR, Hughes JP, Wang RS, Baeten JM, Celum C, Gray GE, et al. Estimating the impact of plasma HIV-1 RNA reductions on heterosexual HIV-1 transmission risk. PLoS One. 2010;5(9):e12598.

19. Hollingsworth TD, Anderson RM, Fraser C. HIV-1 transmission, by stage of infection. The Journal of infectious diseases. 2008;198(5):687-93.

20. Lodi S, Phillips A, Touloumi G, Geskus R, Meyer L, Thiebaut R, et al. Time from human immunodeficiency virus seroconversion to reaching CD4+ cell count thresholds <200, <350, and <500 Cells/mm(3): assessment of need following changes in treatment guidelines. Clinical infectious diseases : an official publication of the Infectious Diseases Society of America. 2011;53(8):817-25.

21. Lyles RH, Munoz A, Yamashita TE, Bazmi H, Detels R, Rinaldo CR, et al. Natural history of human immunodeficiency virus type 1 viremia after seroconversion and proximal to AIDS in a large cohort of homosexual men. Multicenter AIDS Cohort Study. The Journal of infectious diseases. 2000;181(3):872-80.

22. Pantazis N, Morrison C, Amornkul PN, Lewden C, Salata RA, Minga A, et al. Differences in HIV natural history among African and non-African seroconverters in Europe and seroconverters in sub-Saharan Africa. PLoS One. 2012;7(3):e32369.

23. Badri M, Lawn SD, Wood R. Short-term risk of AIDS or death in people infected with HIV-1 before antiretroviral therapy in South Africa: a longitudinal study. Lancet (London, England). 2006;368(9543):1254-9.

24. Lewden C, Gabillard D, Minga A, Ekouevi DK, Avit D, Konate I, et al. CD4-specific mortality rates among HIV-infected adults with high CD4 counts and no antiretroviral treatment in West Africa. Journal of acquired immune deficiency syndromes (1999). 2012;59(2):213-9.

25. Maduna PH, Dolan M, Kondlo L, Mabuza H, Dlamini JN, Polis M, et al. Morbidity and mortality according to latest CD4+ cell count among HIV positive individuals in South Africa who enrolled in project Phidisa. PLoS One. 2015;10(4):e0121843.

26. Adler WH, Baskar PV, Chrest FJ, Dorsey-Cooper B, Winchurch RA, Nagel JE. HIV infection and aging: mechanisms to explain the accelerated rate of progression in the older patient. Mech Ageing Dev. 1997;96(1-3):137-55.

27. Newell ML, Coovadia H, Cortina-Borja M, Rollins N, Gaillard P, Dabis F, et al. Mortality of infected and uninfected infants born to HIV-infected mothers in Africa: a pooled analysis. Lancet (London, England). 2004;364(9441):1236-43.

28. Bruni L AG, Serrano B, Mena M, Gómez D, Muñoz J, Bosch FX, de Sanjosé S. Human Papillomavirus and Related Diseases in South Africa: Summary Report 17 June 2019.

29. de Sanjose S, Quint WG, Alemany L, Geraets DT, Klaustermeier JE, Lloveras B, et al. Human papillomavirus genotype attribution in invasive cervical cancer: a retrospective cross-sectional worldwide study. Lancet Oncol. 2010;11(11):1048-56.

30. Denny L, Adewole I, Anorlu R, Dreyer G, Moodley M, Smith T, et al. Human papillomavirus prevalence and type distribution in invasive cervical cancer in sub-Saharan Africa. International journal of cancer. 2014;134(6):1389-98.

31. van Aardt MC, Dreyer G, Pienaar HF, Karlsen F, Hovland S, Richter KL, et al. Unique human papillomavirus-type distribution in South African women with invasive cervical cancer and the effect of human immunodeficiency virus infection. International journal of gynecological cancer : official journal of the International Gynecological Cancer Society. 2015;25(5):919-25.

32. Dartell M, Rasch V, Kahesa C, Mwaiselage J, Ngoma T, Junge J, et al. Human papillomavirus prevalence and type distribution in 3603 HIV-positive and HIV-negative women in the general population of Tanzania: the PROTECT study. Sexually transmitted diseases. 2012;39(3):201-8.

33. Van Aardt MC, Dreyer G, Snyman LC, Richter KL, Becker P, Mojaki SM. Oncogenic and incidental HPV types associated with histologically confirmed cervical intraepithelial neoplasia in HIV-positive and HIV-negative South African women. S Afr Med J. 2016;106(6).

34. Clifford GM, Rana RK, Franceschi S, Smith JS, Gough G, Pimenta JM. Human papillomavirus genotype distribution in low-grade cervical lesions: comparison by geographic region and with cervical cancer. Cancer epidemiology, biomarkers & prevention : a publication of the American Association for Cancer Research, cosponsored by the American Society of Preventive Oncology. 2005;14(5):1157-64.

35. Van Aardt MC, Dreyer G, Richter KL, Becker P. Human papillomavirus-type distribution in South African women without cytological abnormalities: a peri-urban study. Southern African Journal of Gynaecological Oncology. 2013;5(sup1):S21-S7.

36. Beachler DC, Jenkins G, Safaeian M, Kreimer AR, Wentzensen N. Natural Acquired Immunity Against Subsequent Genital Human Papillomavirus Infection: A Systematic Review and Meta-analysis. The Journal of infectious diseases. 2016;213(9):1444-54.

37. Kong X, Wang MC, Gray R. Analysis of longitudinal multivariate outcome data from couples cohort studies: application to HPV transmission dynamics. J Am Stat Assoc. 2015;110(510):472-85.

38. Liu G, Sharma M, Tan N, Barnabas RV. HIV-positive women have higher risk of human papilloma virus infection, precancerous lesions, and cervical cancer. AIDS. 2018;32(6):795-808.

39. Weller S, Davis K. Condom effectiveness in reducing heterosexual HIV transmission. Cochrane Database Syst Rev. 2002(1):CD003255.

40. Connolly C, Simbayi LC, Shanmugam R, Nqeketo A. Male circumcision and its relationship to HIV infection in South Africa: results of a national survey in 2002. S Afr Med J. 2008;98(10):789-94.

41. Doyle D. Ritual male circumcision: a brief history. J R Coll Physicians Edinb. 2005;35(3):279-85.

42. HIV/AIDS WHOaJUNPo. Male circumcision: global trends and determinants of prevalence, safety and acceptability. Switzerland2007.

43. Shisana O RT, Simbayi LC, Zuma K, Jooste S, Zungu N, Labadarios D,, Onoya D ea. South African National HIV Prevalence, Incidence and Behaviour Survey, 2012. Cape Town; 2014.

44. Shisana O SL, Rehle T, Zungu NP, Zuma K, Ngogo N, Jooste S, PillayVan Wyk V, Parker W, Pezi S, Davids A, Nwanyanwu O, Dinh TH, SABSSM III Implementation, Team. South African National HIV Prevalence, Incidence, Behaviour and Communication Survey, 2008: The health of our children. Cape Town; 2010.

45. Simbayi LC ZK, Zungu N, Moyo S, Marinda E, Jooste S, Mabaso M, Ramlagan S, North A, van Zyl J, Mohlabane N, Dietrich C, Naidoo I, SABSSM V Team. The Fifth South African National HIV Prevalence, Incidence, Behaviour and Communications Survey, 2017. Cape Town; 2019.

46. National Department of Health (NDoH) SSASS, South African Medical Research, Council (SAMRC) I. South Africa Demographic and Health Survey 2016. Pretoria, South Africa, and Rockville, Maryland, USA; 2019.

47. Gray RH, Kigozi G, Serwadda D, Makumbi F, Watya S, Nalugoda F, et al. Male circumcision for HIV prevention in men in Rakai, Uganda: a randomised trial. Lancet (London, England). 2007;369(9562):657-66.

48. Weiss HA, Quigley MA, Hayes RJ. Male circumcision and risk of HIV infection in sub-Saharan Africa: a systematic review and meta-analysis. AIDS. 2000;14(15):2361-70.

49. Tobian AA, Kong X, Wawer MJ, Kigozi G, Gravitt PE, Serwadda D, et al. Circumcision of HIV-infected men and transmission of human papillomavirus to female partners: analyses of data from a randomised trial in Rakai, Uganda. The Lancet Infectious diseases. 2011;11(8):604-12.

50. Weiss HA, Hankins CA, Dickson K. Male circumcision and risk of HIV infection in women: a systematic review and meta-analysis. The Lancet Infectious diseases. 2009;9(11):669-77.

51. Lei JH, Liu LR, Wei Q, Yan SB, Yang L, Song TR, et al. Circumcision Status and Risk of HIV Acquisition during Heterosexual Intercourse for Both Males and Females: A Meta-Analysis. PLoS One. 2015;10(5):e0125436.

52. Rodger AJ, Cambiano V, Bruun T, Vernazza P, Collins S, van Lunzen J, et al. Sexual Activity Without Condoms and Risk of HIV Transmission in Serodifferent Couples When the HIV-Positive Partner Is Using Suppressive Antiretroviral Therapy. Jama. 2016;316(2):171-81.

53. Eisinger RW, Dieffenbach CW, Fauci AS. HIV Viral Load and Transmissibility of HIV Infection: Undetectable Equals Untransmittable. Jama. 2019;321(5):451-2.

54. Brinkhof MW, Boulle A, Weigel R, Messou E, Mathers C, Orrell C, et al. Mortality of HIV-infected patients starting antiretroviral therapy in sub-Saharan Africa: comparison with HIV-unrelated mortality. PLoS medicine. 2009;6(4):e1000066.

55. Cornell M, Johnson LF, Wood R, Tanser F, Fox MP, Prozesky H, et al. Twelve-year mortality in adults initiating antiretroviral therapy in South Africa. Journal of the International AIDS Society. 2017;20(1):21902.

56. de Coninck Z, Hussain-Alkhateeb L, Bratt G, Ekstrom AM, Gisslen M, Petzold M, et al. Non-AIDS Mortality Is Higher Among Successfully Treated People Living with HIV Compared with Matched HIV-Negative Control Persons: A 15-Year Follow-Up Cohort Study in Sweden. AIDS patient care and STDs. 2018;32(8):297-305.

57. Lilian RR, Rees K, Mabitsi M, McIntyre JA, Struthers HE, Peters RPH. Baseline CD4 and mortality trends in the South African human immunodeficiency virus programme: Analysis of routine data. South Afr J HIV Med. 2019;20(1):963.

58. Meyer-Rath G, Johnson LF, Pillay Y, Blecher M, Brennan AT, Long L, et al. Changing the South African national antiretroviral therapy guidelines: The role of cost modelling. PLoS One. 2017;12(10):e0186557.

59. UNAIDS. South Africa2020 [Available from: <https://www.unaids.org/en/regionscountries/countries/southafrica>.

60. Kharsany ABM, Cawood C, Lewis L, Yende-Zuma N, Khanyile D, Puren A, et al. Trends in HIV Prevention, Treatment, and Incidence in a Hyperendemic Area of KwaZulu-Natal, South Africa. JAMA Netw Open. 2019;2(11):e1914378.

61. Takuva S, Brown AE, Pillay Y, Delpech V, Puren AJ. The continuum of HIV care in South Africa: implications for achieving the second and third UNAIDS 90-90-90 targets. AIDS. 2017;31(4):545-52.

62. Zaidi J, Grapsa E, Tanser F, Newell ML, Barnighausen T. Dramatic increase in HIV prevalence after scale-up of antiretroviral treatment. AIDS. 2013;27(14):2301-5.

63. 90-90-90: AN AMBITIOUS TREATMENT TARGET TO HELP END THE AIDS EPIDEMIC: UNAIDS; [Available from: <https://www.unaids.org/en/resources/909090>.

64. Pierce Campbell CM, Lin HY, Fulp W, Papenfuss MR, Salmeron JJ, Quiterio MM, et al. Consistent condom use reduces the genital human papillomavirus burden among high-risk men: the HPV infection in men study. The Journal of infectious diseases. 2013;208(3):373-84.

65. Winer RL, Hughes JP, Feng Q, O'Reilly S, Kiviat NB, Holmes KK, et al. Condom use and the risk of genital human papillomavirus infection in young women. The New England journal of medicine. 2006;354(25):2645-54.

66. Albero G, Castellsague X, Lin HY, Fulp W, Villa LL, Lazcano-Ponce E, et al. Male circumcision and the incidence and clearance of genital human papillomavirus (HPV) infection in men: the HPV Infection in men (HIM) cohort study. BMC infectious diseases. 2014;14:75.

67. Albero G, Villa LL, Lazcano-Ponce E, Fulp W, Papenfuss MR, Nyitray AG, et al. Male circumcision and prevalence of genital human papillomavirus infection in men: a multinational study. BMC infectious diseases. 2013;13:18.

68. Vanbuskirk K, Winer RL, Hughes JP, Feng Q, Arima Y, Lee SK, et al. Circumcision and acquisition of human papillomavirus infection in young men. Sexually transmitted diseases. 2011;38(11):1074-81.

69. Tobian AA, Kong X, Gravitt PE, Eaton KP, Kigozi G, Serwadda D, et al. Male circumcision and anatomic sites of penile high-risk human papillomavirus in Rakai, Uganda. International journal of cancer. 2011;129(12):2970-5.

70. Denny L. Prevention of cervical cancer. Reprod Health Matters. 2008;16(32):18-31.

71. Godfrey MAL, Mathenjwa S, Mayat N. Rural Zulu women's knowledge of and attitudes towards Pap smears and adherence to cervical screening. Afr J Prim Health Care Fam Med. 2019;11(1):e1-e6.

72. Gakidou E, Nordhagen S, Obermeyer Z. Coverage of cervical cancer screening in 57 countries: low average levels and large inequalities. PLoS medicine. 2008;5(6):e132.

73. Jordaan S, P M, K R, Simoens C, Bogers J-P. A Review of Cervical Cancer in South Africa: Previous, Current and Future. Health Care : Current Reviews. 2016;04.

74. Hoque M, Hoque E, Kader SB. Evaluation of cervical cancer screening program at a rural community of South Africa. East Afr J Public Health. 2008;5(2):111-6.

75. Africa NDoHRoS. Cervical Cancer Prevention and Control Policy. 2017.

76. Arbyn M, Sankaranarayanan R, Muwonge R, Keita N, Dolo A, Mbalawa CG, et al. Pooled analysis of the accuracy of five cervical cancer screening tests assessed in eleven studies in Africa and India. International journal of cancer. 2008;123(1):153-60.

77. Khozaim K, Orang'o E, Christoffersen-Deb A, Itsura P, Oguda J, Muliro H, et al. Successes and challenges of establishing a cervical cancer screening and treatment program in western Kenya. International journal of gynaecology and obstetrics: the official organ of the International Federation of Gynaecology and Obstetrics. 2014;124(1):12-8.

78. Debeaudrap P, Sobngwi J, Tebeu PM, Clifford GM. Residual or recurrent precancerous lesions after treatment of cervical lesions in HIV-infected women: a systematic review and meta-analysis of treatment failure. Clinical infectious diseases : an official publication of the Infectious Diseases Society of America. 2019.

79. Hoffman SR, Le T, Lockhart A, Sanusi A, Dal Santo L, Davis M, et al. Patterns of persistent HPV infection after treatment for cervical intraepithelial neoplasia (CIN): A systematic review. International journal of cancer. 2017;141(1):8-23.

80. Filippi S, Barnes CP, Cornebise J, Stumpf MPH. On optimality of kernels for approximate Bayesian computation using sequential Monte Carlo. Stat Appl Genet Mol. 2013;12(1).

81. Lenormand M, Jabot F, Deffuant G. Adaptive approximate Bayesian computation for complex models. Computation Stat. 2013;28(6):2777-96.

82. Toni T, Welch D, Strelkowa N, Ipsen A, Stumpf MPH. Approximate Bayesian computation scheme for parameter inference and model selection in dynamical systems. J R Soc Interface. 2009;6(31):187-202.

83. Vandormael A, Akullian A, Siedner M, de Oliveira T, Barnighausen T, Tanser F. Declines in HIV incidence among men and women in a South African population-based cohort. Nat Commun. 2019;10(1):5482.

84. Beauclair R, Helleringer S, Hens N, Delva W. Age differences between sexual partners, behavioural and demographic correlates, and HIV infection on Likoma Island, Malawi. Sci Rep. 2016;6:36121.

85. Mabaso M, Mlangeni L, Makola L, Oladimeji O, Naidoo I, Naidoo Y, et al. Factors associated with age-disparate sexual partnerships among males and females in South Africa: a multinomial analysis of the 2012 national population-based household survey data. Emerg Themes Epidemiol. 2021;18(1):3.

86. Boily MC, Baggaley RF, Wang L, Masse B, White RG, Hayes RJ, et al. Heterosexual risk of HIV-1 infection per sexual act: systematic review and meta-analysis of observational studies. The Lancet Infectious diseases. 2009;9(2):118-29.

87. Burchell AN, Richardson H, Mahmud SM, Trottier H, Tellier PP, Hanley J, et al. Modeling the sexual transmissibility of human papillomavirus infection using stochastic computer simulation and empirical data from a cohort study of young women in Montreal, Canada. American journal of epidemiology. 2006;163(6):534-43.

88. Rosillon D, Baril L, Del Rosario-Raymundo MR, Wheeler CM, Skinner SR, Garland SM, et al. Risk of newly detected infections and cervical abnormalities in adult women seropositive or seronegative for naturally acquired HPV-16/18 antibodies. Cancer Med. 2019;8(10):4938-53.

89. Johnson HC, Elfstrom KM, Edmunds WJ. Inference of type-specific HPV transmissibility, progression and clearance rates: a mathematical modelling approach. PLoS One. 2012;7(11):e49614.

90. Campos NG, Burger EA, Sy S, Sharma M, Schiffman M, Rodriguez AC, et al. An updated natural history model of cervical cancer: derivation of model parameters. American journal of epidemiology. 2014;180(5):545-55.

91. Dryden-Peterson S, Bvochora-Nsingo M, Suneja G, Efstathiou JA, Grover S, Chiyapo S, et al. HIV Infection and Survival Among Women With Cervical Cancer. Journal of clinical oncology : official journal of the American Society of Clinical Oncology. 2016;34(31):3749-57.

92. Sankaranarayanan R, Swaminathan R, Brenner H, Chen K, Chia KS, Chen JG, et al. Cancer survival in Africa, Asia, and Central America: a population-based study. Lancet Oncol. 2010;11(2):165-73.

93. Africa SS. Census 2001: Post-enumeration survey: Results and methodology. Pretoria, South Africa2004.

94. Africa SS. Census 2011: Post-enumeration Survey: Results and methodology. Pretoria, South Africa2012.

95. Africa SS. Census 2001: Primary tables KwaZulu-Natal: 1996 and 2001 compared. Pretoria, South Africa2004.

96. Africa SS. Community Survey 2016. Statistical release P0301. Pretoria, South Africa2016.

97. McDonald AC, Tergas AI, Kuhn L, Denny L, Wright TC, Jr. Distribution of Human Papillomavirus Genotypes among HIV-Positive and HIV-Negative Women in Cape Town, South Africa. Front Oncol. 2014;4:48.

98. Mbulawa ZZ, Coetzee D, Williamson AL. Human papillomavirus prevalence in South African women and men according to age and human immunodeficiency virus status. BMC infectious diseases. 2015;15:459.

99. Kuhn L, Saidu R, Boa R, Tergas A, Moodley J, Persing D, et al. Clinical evaluation of modifications to a human papillomavirus assay to optimise its utility for cervical cancer screening in low-resource settings: a diagnostic accuracy study. The Lancet Global health. 2020;8(2):e296-e304.

100. Bray F, Ferlay J, Soerjomataram I, Siegel RL, Torre LA, Jemal A. Global cancer statistics 2018: GLOBOCAN estimates of incidence and mortality worldwide for 36 cancers in 185 countries. CA: A Cancer Journal for Clinicians. 2018;68(6):394-424.
