# Supplementary material for: Estimating the effect of HIV on cervical cancer elimination in South Africa: Comparative modelling of the impact of vaccination and screening
Source: eClinicalMedicine. 2022 Nov 17;54:101754. doi: 10.1016/j.eclinm.2022.101754 (PMC9793279; doi:10.1016/j.eclinm.2022.101754)
Supplement: Captions for Supplement material [file mmc10.docx]

**Captions for Supplement material**

**Supplement figure S1**

**Supplement figure S2**

**Supplement figures S3-S4**

**Supplement figures S4-S9**

**Supplement tables S1**

**Technical Appendix**

**Model specific _Technical Appendix _T1**

**Model specific _Technical Appendix _T2**

**Model specific _Technical Appendix _T3**
